# Hiding in plain sight: Optimizing topoisomerase IIα inhibitors into Hsp90β selective binders

**DOI:** 10.1016/j.ejmech.2024.116934

**Published:** 2024-10-05

**Authors:** Jaka Dernovšek, Tjaša Goričan, Marius Gedgaudas, Živa Zajec, Dunja Urbančič, Ana Jug, Žiga Skok, Caterina Sturtzel, Martin Distel, Simona Golič Grdadolnik, Kesavan Babu, Ashna Panchamatia, Timothy R. Stachowski, Marcus Fischer, Janez Ilaš, Asta Zubrienė, Daumantas Matulis, Nace Zidar, Tihomir Tomašič

**Affiliations:** a Faculty of Pharmacy, University of Ljubljana, Aškerčeva cesta 7, 1000, Ljubljana, Slovenia; b Laboratory for Molecular Structural Dynamics, Theory Department, National Institute of Chemistry, Hajdrihova 19, 1001, Ljubljana, Slovenia; c Department of Biothermodynamics and Drug Design, Institute of Biotechnology, Vilnius University, Saulėtekio al. 7 (C319), LT-10257, Vilnius, Lithuania; d St. Anna Children’s Cancer Research Institute, Zimmermannplatz 10, 1090, Vienna, Austria; e Department of Chemical Biology and Therapeutics, St. Jude Children’s Research Hospital, 262 Danny Thomas Place, Memphis, TN, 38105-3678, USA

**Keywords:** Cancer, Hsp90, Isoform selectivity, Topoisomerase II

## Abstract

Due to their impact on several oncogenic client proteins, the Hsp90 family of chaperones has been widely studied for the development of potential anticancer agents. Although several Hsp90 inhibitors have entered clinical trials, most were unsuccessful because they induced a heat shock response (HSR). This issue can be circumvented by using isoform-selective inhibitors, but the high similarity in the ATP-binding sites between the isoforms presents a challenge. Given that Hsp90 shares a conserved Bergerat fold with bacterial DNA gyrase B and human topoisomerase IIα, we repurposed our ATP-competitive inhibitors of these two proteins for Hsp90 inhibition. We virtually screened a library of in-house inhibitors and identified eleven hits for evaluation of Hsp90 binding. Among these, compound **11** displayed low micromolar affinity for Hsp90 and demonstrated a 12-fold selectivity for Hsp90β over its closest isoform, Hsp90α. Out of 29 prepared analogs, 16 showed a preference for Hsp90β over Hsp90α. Furthermore, eleven of these compounds inhibited the growth of several cancer cell lines *in vitro*. Notably, compound **24e** reduced intracellular levels of Hsp90 client proteins in MCF-7 cells, leading to cell cycle arrest in the G0/G1 phase without inducing HSR. This inhibitor exhibited at least a 27-fold preference for Hsp90β and was selective against topoisomerase IIα, a panel of 22 representative protein kinases, and proved to be non-toxic in a zebrafish larvae toxicology model. Finally, molecular modeling, corroborated by STD NMR studies, and the binding of **24e** to the S52A mutant of Hsp90α confirmed that the serine to alanine switch drives the selectivity between the two cytoplasmic isoforms.

## 1. Introduction

Molecular chaperones are key cellular proteins that ensure proteostasis. Among the chaperones, the Hsp90 protein family is the most abundant, accounting for 1–2% of total cellular protein load under normal conditions [1]. However, the expression of Hsp90 is usually increased more than two-fold when cells are exposed to stress factors such as oxygen and nutrient deprivation. Considering the harsh microenvironment of tumors, it is not surprising that Hsp90 is also overexpressed in many types of cancer cells [2,3], which indicates the importance of the Hsp90 family in cancer development and progression.

Indeed, all four isoforms of the Hsp90 family are involved in cancer, mainly by affecting their client proteins, which rely on Hsp90 to obtain their correct tertiary structure [4–6]. Several hundred clients of Hsp90 have been identified, many of which are relevant in various signaling pathways that malfunction in cancer, making Hsp90 indirectly involved in all hallmarks of cancer [1,2,7]. For example, the constitutive cytoplasmic isoform Hsp90β is responsible for the folding and maturation of CDK4/6 [8,9], c-IAP1 [9] and CXCR4 [10]. Some of the most distinct clients of the inducible cytosolic paralog Hsp90α are c-Src [11], Raf-1 [8] and survivin [11,12]. Glucose regulated protein 94 (Grp94) is the Hsp90 isoform localized in the endoplasmic reticulum and is responsible for client proteins specific to this organelle, such as insulin growth factors (IGF-1 and IGF-2) and integrins [13]. The most dissimilar isoform is mitochondria-localized TNF receptor associated protein 1 (TRAP-1), whose main function is to protect the mitochondria from oxidative stress [14].

Since the simultaneous reduction in cellular concentration of the above mentioned and other cancer-relevant Hsp90 clients (e.g. estrogen receptor α, Akt, etc.) could have potent synergistic effects in cancer therapy [15,16], it is not surprising that 22 N-terminal pan-Hsp90 inhibitors have been clinically investigated [17–19]. To date, only one agent has entered the market: Hsp90α/β-selective inhibitor pimitespib (TAS-116) was approved in Japan in 2022 for the treatment of gastrointestinal stromal tumors (GIST) [20]. Other Hsp90 inhibitors failed in clinical trials due to various toxicities, lack of efficacy and the induction of heat shock response (HSR), which spawns the overexpression of different heat shock proteins, directing the cell into a pro-survival state [18,21,22]. As a result, the Hsp90 scientific community has begun to explore different strategies of Hsp90 inhibition, as Hsp90 structure enables more than one potential modulation approach [2,23].

Hsp90 is a functional homodimer, and each monomeric unit consists of the ATP-binding and hydrolyzing N-terminal domain (NTD) [24]. The ATP-binding pocket is also the most common target of clinically evaluated pan-Hsp90 inhibitors [25]. The NTD is connected to the client- and cochaperone-binding middle domain (MD) via a charged linker. The MD transitions to the C-terminal domain (CTD), which is primarily responsible for dimerization and contains allosteric binding sites that can be exploited to develop Hsp90 CTD inhibitors that do not induce HSR [26,27]. Additionally, bypassing the HSR induction is also achieved by inhibitors of protein-protein interactions (PPI) between Hsp90 and its co-chaperones [28,29]. Finally, HSR can be circumvented by isoform-selective NTD inhibitors. These inhibitors utilize small differences in amino acid residues of the N-terminal ATP-binding pockets between members of the Hsp90 family to achieve an isoform-selective mode of action. Therefore, only a smaller group of client proteins is affected in such manner, resulting in a lower incidence of toxicities [23, 30,31]. For example, the hERG channel is an Hsp90α-dependent client responsible for potential cardiotoxicity of pan-Hsp90 inhibitors [32]. Together with the absence of HSR, isoform-selective Hsp90β inhibitors have therefore arguably superior therapeutic potential [8,9], but the development of such compounds is particularly challenging.

**Fig. 1.**
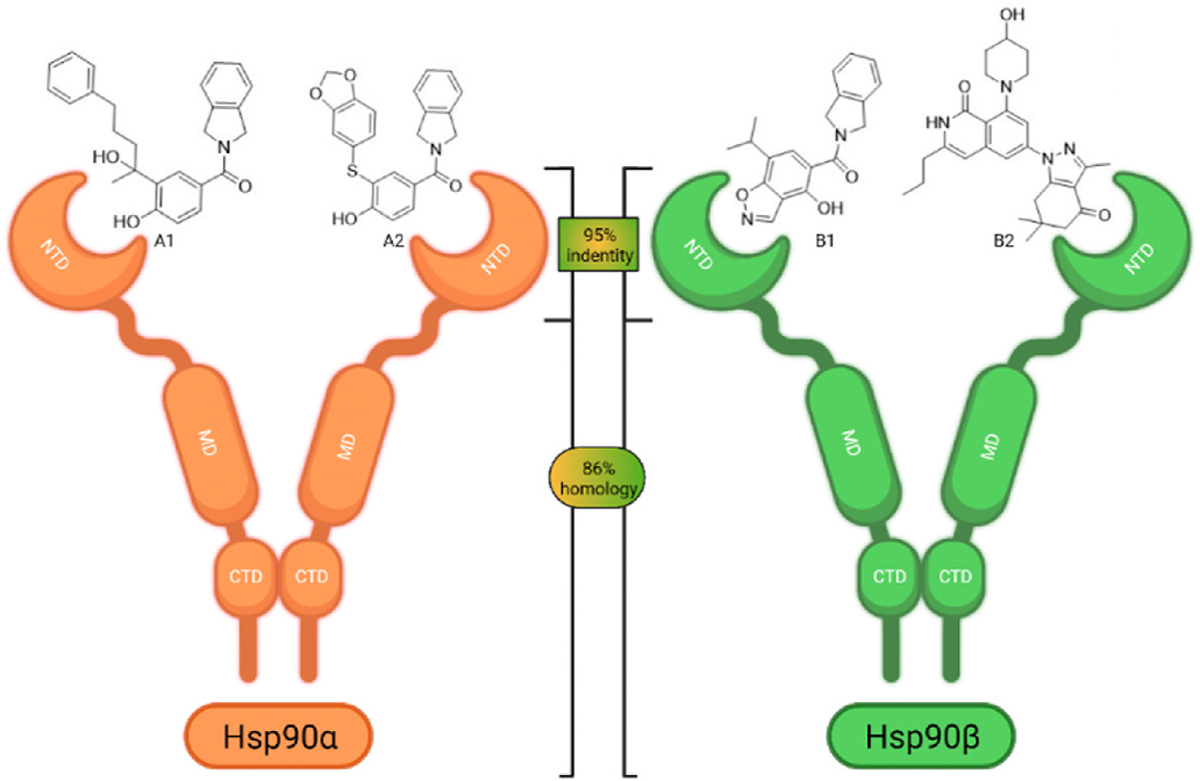
Representative Hsp90α selective (A1 and A2) and Hsp90β selective (B1 and B2) inhibitors that are able to bind selectively to the N-terminal domains of Hsp90α and Hsp90β with 95 % sequence identity.

All four Hsp90 isoforms are highly conserved; even the compartmental proteins TRAP-1 and Grp94 are approximately 34 % and 50 % identical to the cytoplasmic isoforms, respectively [33,34]. Focusing solely on the ATP-binding sites, the similarity is even higher – more than 85 % identity in the NTD within the Hsp90 family [9]. Furthermore, if one compares only Hsp90α and Hsp90β, they are 86 % homologous and, if we consider only the NTD ATP-binding pockets of these two isoforms 24 out of 26 amino acid residues are conserved – a 95 % sequence identity [8]. Therefore, only four compound classes of selective Hsp90α or Hsp90β inhibitors have been described to date (Fig. 1) [8,9,11,12,35]. Moreover, ATP-binding pocket conservation is not limited to the Hsp90 family; a similar binding site is also present within the GHKL protein family. DNA gyrase, histidine kinase, MutL, Hsp90, and topoisomerase II all share the unusual Bergerat ATP-binding fold, leading to similar nucleotide binding sites [36]. A closer look at the superimposed GHKL members Hsp90 and topoisomerase IIα (TopoIIα) revealed that although the amino acid sequence identity in the ATP-binding site is quite low (15.8 %), the similarity in residue properties is much higher [37]. In addition, a study comparing the positions of some of the major ATP-binding amino acid residues Asp93 and Asn51 in Hsp90α with Asn120 and Asn91 in TopoIIα, respectively, revealed their almost complete overlap, and a naturally occurring dual inhibitor of these two proteins quinacrine was identified [38].

To explore the possibility of repurposing our ATP-competitive DNA gyrase and TopoIIα inhibitors [39–42] for Hsp90 we set out to discover and optimize a new set of inhibitors. First, we identified a new class of Hsp90β selective inhibitors by virtual screening. Then, our hit optimization efforts, led to new inhibitors that have little to no affinity for TopoIIα and show a greater than 30-fold selectivity for Hsp90β compared to Hsp90α. Finally, our compounds inhibited cancer cell growth at low micromolar concentrations, similar to the clinically evaluated 17-DMAG, and our lead compound was able to reduce intracellular levels of Hsp90 dependent client proteins without significant HSR induction.

## 2. Results and discussion

### 2.1. Hit identification and design of optimized analogs

**Fig. 2.**
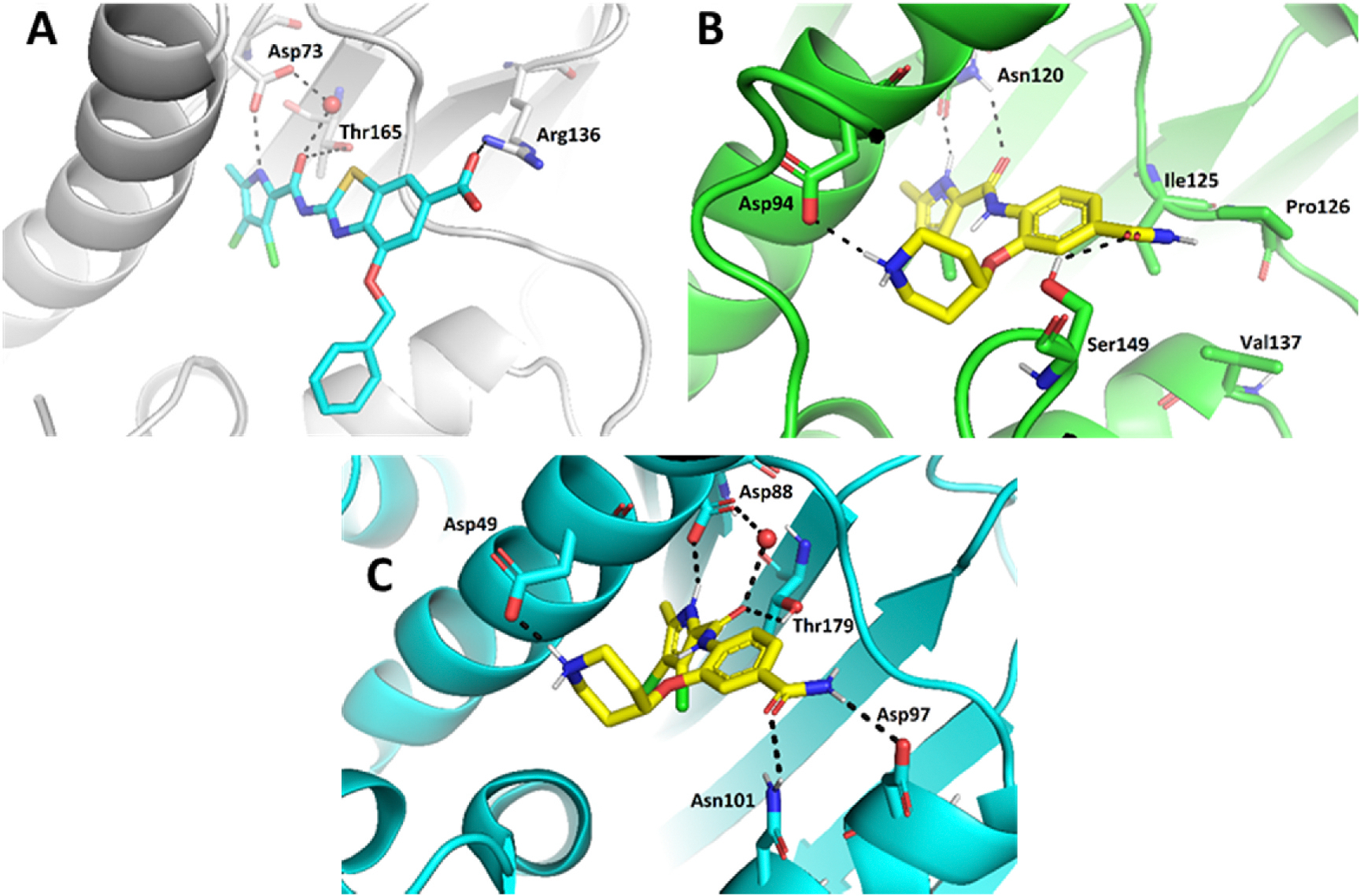
**A**) X-ray co-crystal structure binding mode of pyrrolamide-based inhibitor ULD2 (in cyan sticks) in the ATP-binding site of *Escherichia coli* DNA gyrase B (in grey cartoon, PDB entry: 7P2M); **B**) docking binding mode of compound **11** (in yellow sticks) in the ATP-binding site of human TopoIIα (in green cartoon, PDB entry: 4R1F); **C**) docking binding mode of compound **11** (in yellow sticks) in the ATP-binding site of Hsp90β (in cyan cartoon, PDB entry: 5UCJ). For clarity, only selected amino acid residues are presented as sticks. Hydrogen bonds are presented as black dashed lines.

We have previously designed and optimized several structural classes of inhibitors targeting the ATP-binding sites of GHKL superfamily proteins, such as bacterial DNA gyrase and topoisomerase IV [40–42], and human TopoIIα [39,43]. The latter were discovered by repurposing our DNA gyrase library of inhibitors [43] and then optimized to more potent TopoIIα inhibitors with improved anticancer activity [39]. This prompted us to perform a virtual screening to identify new Hsp90β selective inhibitors starting from our library of ATP-competitive inhibitors of DNA gyrase and TopoIIα. We docked an in-house library of 953 such inhibitors into the ATP-binding site of Hsp90β. The top-ranked compounds belonged to the *N-*phenylpyrrolamide class of TopoIIα inhibitors. In general, the pyrrolamide-based DNA gyrase and topoisomerase IV inhibitors were ranked lower than the human TopoIIα inhibitors. These results can be explained by comparing the ATP-binding sites of Hsp90β, DNA gyrase and TopoIIα (Fig. 2). The pyrrolamide-based DNA gyrase inhibitors mimic the binding of the adenine ring of ATP by forming hydrogen bonds with Asp73 (*Escherichia coli* amino acid numbering) and the structural water molecule. The affinity and selectivity for bacterial DNA gyrase is further enhanced by the formation of a salt bridge between the Arg136 side chain and inhibitor’s carboxylic acid or its bioisoster (Fig. 2A). In human TopoIIα, the pocket to which the pyrrolamide-based inhibitors bind is more hydrophobic and is composed of Ile125, Pro126 and Val137 (Fig. 2B). In contrast, in Hsp90β, the Asp97 residue is located at a similar position as the Arg136 in the DNA gyrase (Fig. 2C), resulting in a repulsion between the two negatively charged groups, thereby preventing the binding of pyrrolamide-based DNA gyrase inhibitors to Hsp90β. Of the top-ranked compounds in molecular docking, 11 were selected for initial *in vitro* screening for Hsp90α and Hsp90β affinity using the fluorescence-based thermal shift assay (FTSA, Table 1).

Interestingly, while none of the compounds **1–11** bound Hsp90α, four Hsp90β binders were identified (Table 1). Comparing the inhibitory concentrations (IC_50_ values) for TopoIIα [39] with the dissociation constants (*K*_d_) for Hsp90β, the results for compounds **4–11** correlate to some extent, as compounds **4–7** inhibit neither of the proteins, whereas compounds **8–11** inhibit TopoIIα and interact with Hsp90β. The results highlight the necessity of the cationic center for the binding of the compounds to Hsp90β, while the basic center does not appear to be mandatory for TopoIIα inhibition, as compound **3** is also an inhibitor of this enzyme. The rigidification of the part of the inhibitor that contains the basic center was found to be important for binding to both Hsp90β and TopoIIα. It appears that substituents containing cyclic amines (compound **1** for TopoIIα and **8–11** for both proteins) are favored over more flexible ones (compounds **4** and **6**). Similarly, the 3,4-dichloro-5--methylpyrrolamide group is preferred, as introduction of the 4,5-dibro-mopyrrolamide resulted in a complete loss of affinity for TopoIIα and Hsp90β (compound **5** *vs* **11**). Initial structure-activity relationship (SAR) analysis showed that the primary carboxamide group in the *para* position to the pyrrolamide group on the central phenyl ring is important for enhancing the affinity for both TopoIIα and Hsp90β. In contrast, the introduction of the carboxamide group to the *meta* position of the phenyl ring (**1**) increased the activity for TopoIIα, but was detrimental for Hsp90β binding. The greatest improvement in affinity for Hsp90β was observed with the introduction of the primary carboxamide group to the central phenyl ring of compound **8**, as the affinity of analog **11** for Hsp90β is the highest in the series (*K*_d_ = 16 μM). In addition, compound **11** is more than 12-fold selective for the constitutive cytoplasmic isoform Hsp90β and was therefore selected for further optimization. The pyrrolamide scaffold also has low chemical similarity to known Hsp90 inhibitors (max Tanimoto coeficient = 0.52, Fig. S33) and therefore might provide new medicinal chemistry opportunities to improve inhibition and selectivity that are accessible using known binders.

**Fig. 3.**
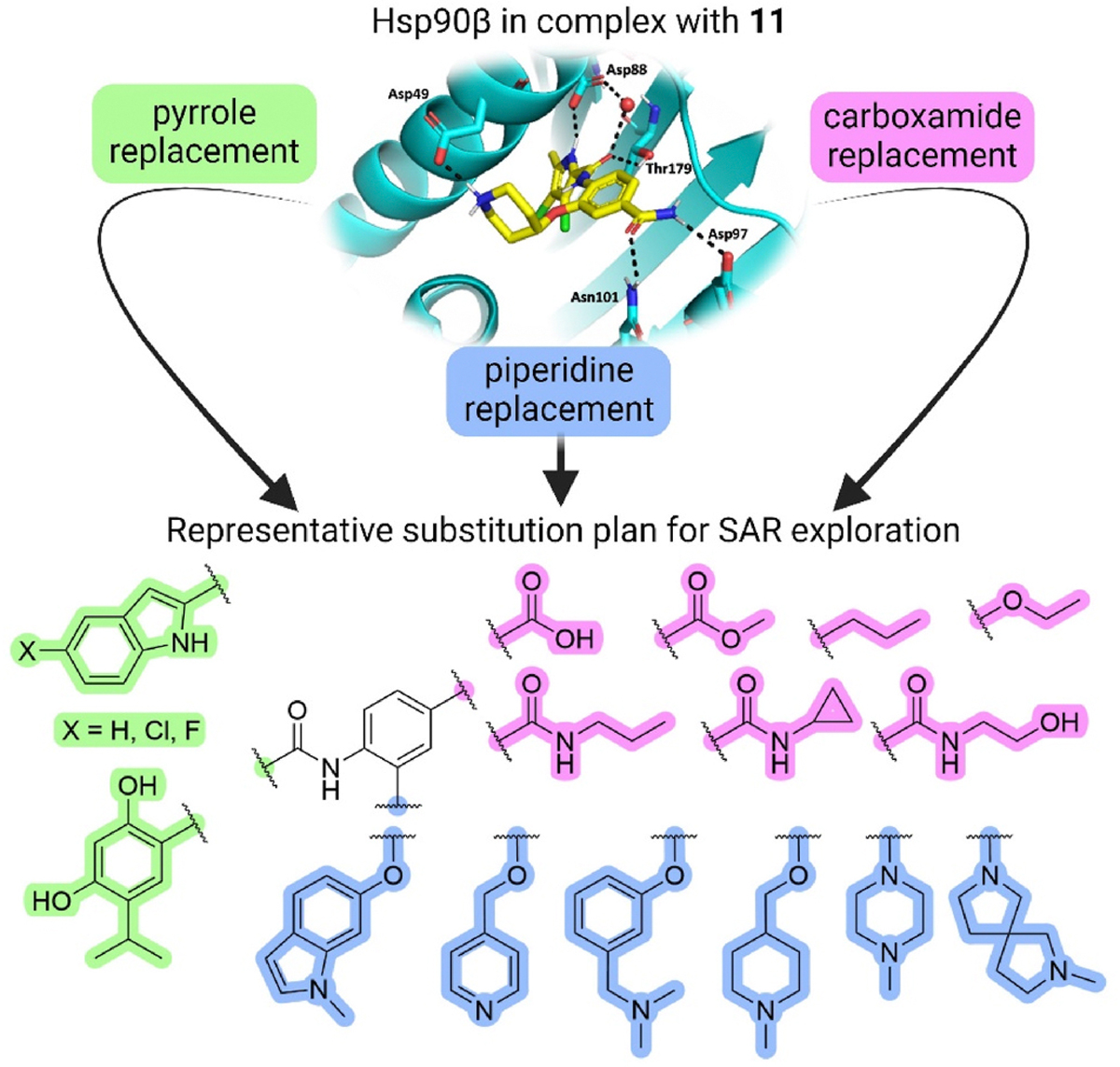
Schematic representation of the structural modifications that were designed based on the predicted binding mode of **11** in the N-terminal ATP-binding site of Hsp90β. In the SAR exploration study, the substituents at positions 1, 2 and 4 of the core phenyl ring will be modified in order to determine the most favorable combination to increase affinity for Hsp90β and selectivity against Hsp90α.

Optimization of the virtual screening hit **11** was guided by its docking binding mode in the ATP-binding site of Hsp90β (Fig. 2C) and molecular dynamics (MD) simulation trajectory analysis. The docking complex of **11** in the ATP-binding site of Hsp90β was subjected to a 500 ns MD simulation, which showed that the predicted binding mode of **11** is quite stable with the RMSD fluctuating around 2.4 Å (Fig. S1). The pyrrole NH group is predicted to form a hydrogen bond with the Asp88 side chain, while the carbonyl group at position 2 of the pyrrole forms hydrogen bonds with a structural water molecule and the Thr179 side chain, resembling the binding mode of the pyrrolamide moiety in the cocrystal structure of the inhibitor ULD2 with *E. coli* DNA gyrase (Fig. 2) [40]. Therefore, when optimizing compound **11**, we decided to leave the hydrogen bond donor and acceptor groups of the pyrrolamide moiety relatively intact, while increasing the size of the amide-bound substituent by replacing the pyrrole (e.g. by introducing the optionally substituted indole ring) (Fig. 3, highlighted in green).

We confirmed the relevance of the basic center, already established by SAR analysis of the eleven hits identified with virtual screening (Table 1), by molecular docking of compound **11** to the ATP-binding site of Hsp90β. This indicated that the piperidine amine forms an ionic interaction with the Asp49 chain (Fig. 2C). However, MD simulation trajectory analysis showed that this part of the inhibitor was rather flexible (Fig. S2), which was reflected in the binding interaction analysis that showed mainly water-mediated contacts to Asp49 (Fig. S3). Therefore, at position 2 of the core phenyl ring we mostly introduced substituents with a positively ionizable group for interaction with Asp49 (Fig. 3, highlighted in blue). In addition, we wanted to reduce the flexibility of this part of the inhibitor to increase the strength of the ionic interaction with Asp49. Since many known Hsp90 inhibitors (e.g. ganetespib [44]) carry an aromatic ring pointing into the same binding pocket, a subset of the designed analogs featured either an aromatic ring or an aromatic linker to the basic center.

According to the docking binding mode of **11**, the terminal carboxamide group interacts with the Asp97 and Asn101 side chains (Fig. 2C), while in the MD simulation trajectory these contacts are mostly water-mediated hydrogen bonds to Gly92 and Asp97 (Fig. S3). Since this carboxamide group appears to be important for Hsp90β affinity, we decided to further investigate its influence on affinity and selectivity by introducing different substituents to the carboxamide nitrogen or replacing it with various functional groups (Fig. 3, highlighted in pink).

### 2.2. Synthesis

**Scheme 1.**
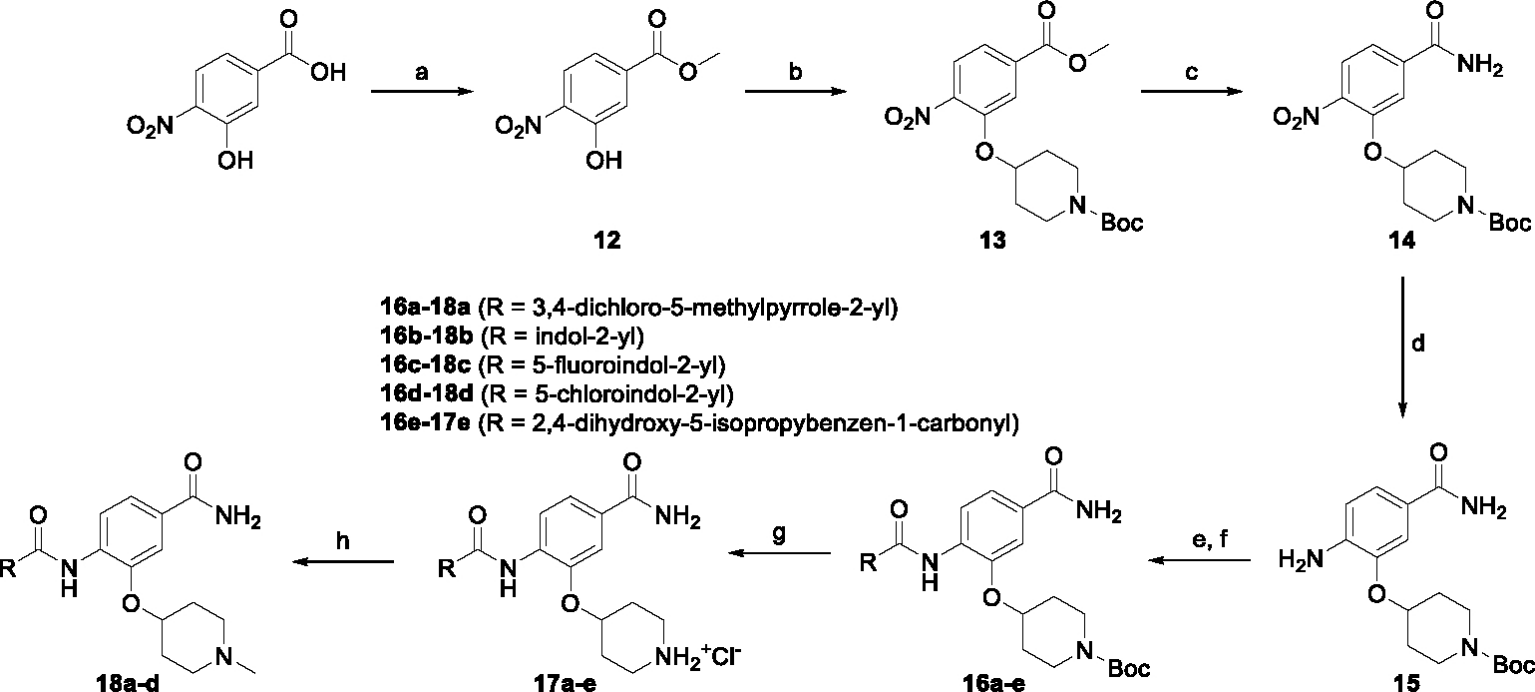
Reagents and conditions: (a) SOCl_2_, MeOH, 0 °C, 15 min to r.t., overnight; (b) *N*-Boc-4-hydroxypiperidine, PPh_3_, DIAD, THF anhydrous, 0 °C to r.t., overnight; (c) NH_3_, MeOH, 100 °C, overnight in a pressure tube; (d) H_2_, Pd/C, MeOH, overnight; (e) for **16a-d** (i) corresponding indole-2-carboxylic acid or 3,4-dichloro-5-methylpyrrole-2-carboxylic acid, SOCl_2_, 70 °C, 1 h; (ii) **15**, DCM anhydrous, pyridine, r.t., overnight; (f) for **16e** (i) 2,4-dihydroxy-5-isopropylbenzoic, acetic anhydride, 65 °C, 1 h; (ii) 2,4-diacetoxy-5-isopropylbenzoic acid, oxalyl chloride, DCM, DMF cat., r.t., 1.5 h; (iii) **15**, DCM anhydrous, pyridine, rt, overnight; (iiii) MeOH, 1 M NaOH, r.t., 30 min; (g) 4 M HCl in 1,4-dioxane, 1,4-dioxane, r.t., overnight; (h) (i) formaldehyde, triethylamine, MeOH/THF, r.t., 1 h; (ii) NaCNBH_3_, r.t., overnight.

**Scheme 2.**
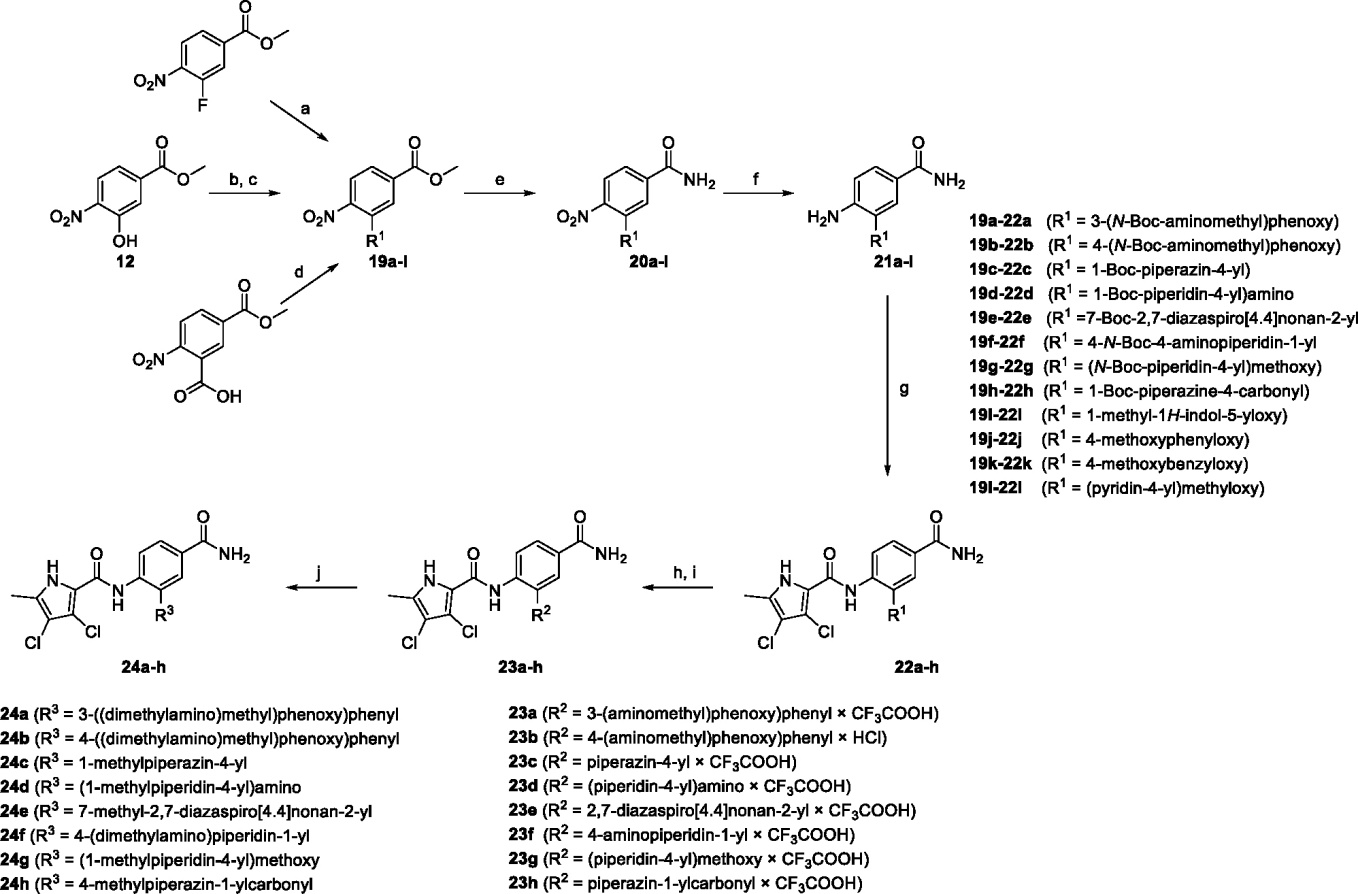
Reagents and conditions: (a) for compounds **19a-f** and **19i-j**, corresponding phenol or corresponding *N*^1^-Boc-protected diamine, K_2_CO_3_, DMF, 60 °C overnight; (b) for **19g**, *N*-Boc-4-hydroxypiperidine, PPh_3_, DIAD, THF anhydrous, 0 °C to r.t., overnight; (c) 4-methoxybenzylchloride for **19k** and 4-(chloromethyl) pyridine for **19l**, K_2_CO_3_, KI, acetone, 60 °C overnight; (d) for **19h** (i) DMF, EDC, HOBt, DIPEA, 0 °C, 20 min; (ii) 1-Boc-piperazine, r.t., overnight; (e) NH_3_, MeOH, 100 °C, overnight in a pressure tube; (f) H_2_, Pd/C, MeOH, overnight; (g) (i) 3,4-dichloro-5-methylpyrrole-2-carboxylic acid, SOCl_2_, DCM anhydrous, 70 °C, 1 h; (ii) **21a-l**, DCM anhydrous, pyridine, rt, overnight; (h) **23b**, 4 M HCl in 1,4-dioxane, 1,4-dioxane, 40 °C, overnight; (i) **23a**, **23c-h**, DCM, TFA, r.t., overnight; (j) (i) MeOH, formaldehyde, triethylamine, r.t., 1 h, (ii) NaCNBH_3_, r.t., overnight.

**Scheme 3.**
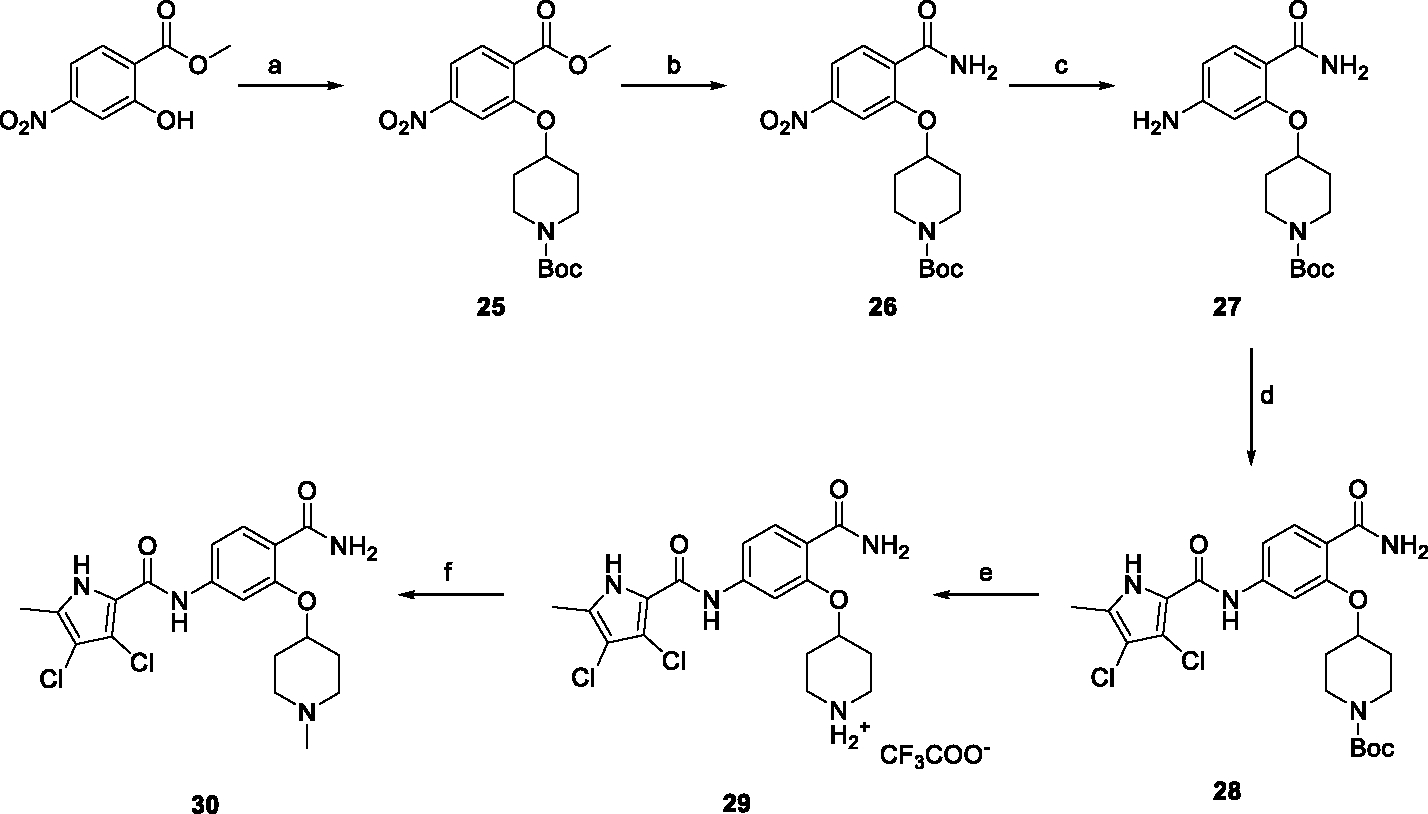
Reagents and conditions: (a) *N*-Boc-4-hydroxypiperidine, PPh_3_, DIAD, THF anhydrous, 0 °C to r.t., overnight; (b) NH_3_, MeOH, 100 °C, overnight in a pressure tube; (c) H_2_, Pd/C, MeOH, overnight; (d) (i) 3,4-dichloro-5-methylpyrrole-2-carboxylic acid, SOCl_2_, DCM anhydrous, 70 °C, 1 h; (ii) **33**, DCM anhydrous, pyridine, r.t., overnight; (e) TFA, DCM, r.t., overnight (f) (i) formaldehyde, triethylamine, MeOH, 1 h, r.t., (ii) NaCNBH_3_, r.t., overnight.

**Scheme 4.**
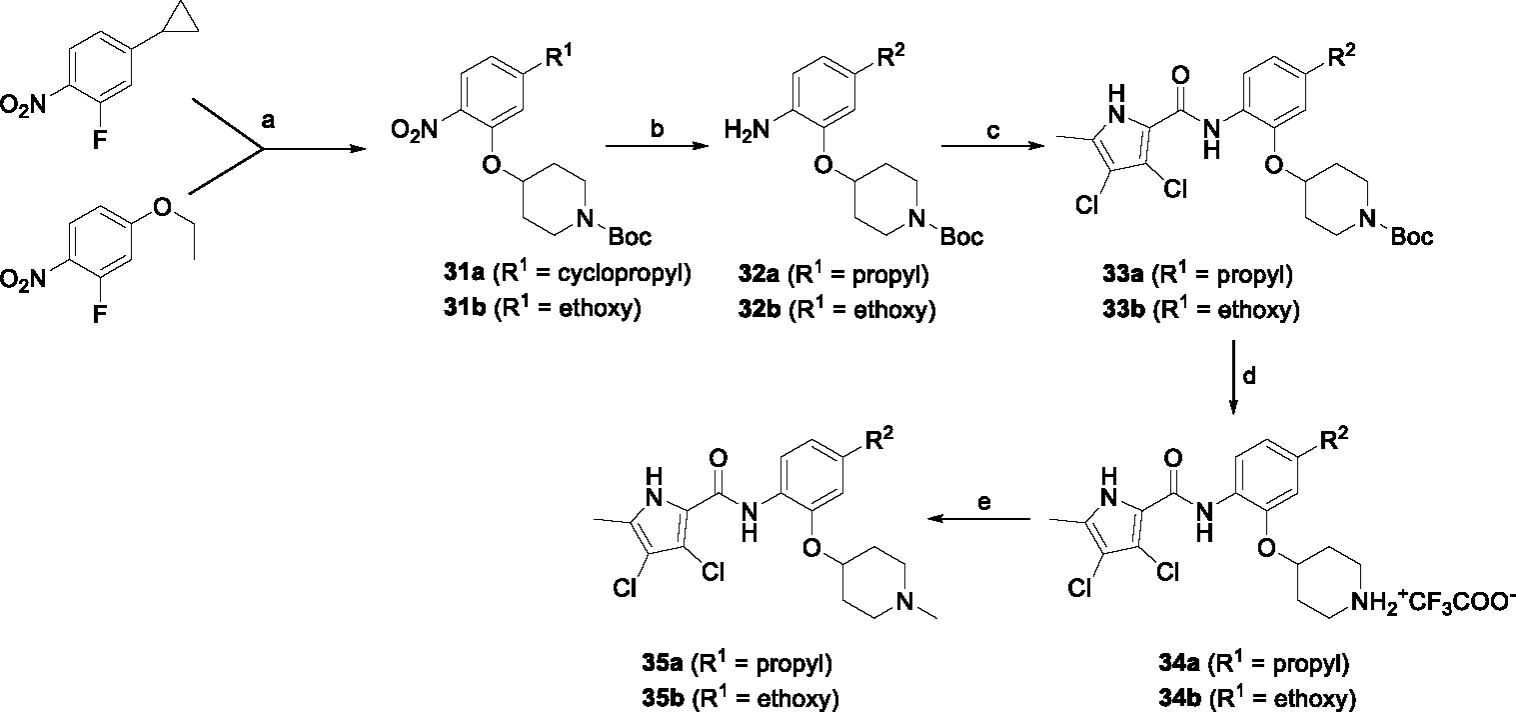
Reagents and conditions: (a) 1-Boc-4-hydroxypiperidine, NaH, DMF anhydrous, 60 °C, overnight; (b) H_2_, Pd/C, MeOH, overnight; (c) (i) 3,4-dichloro-5-methylpyrrole-2-carboxylic acid, SOCl_2_, DCM anhydrous, 70 °C, 1 h; (ii) **32a-b**, DCM anhydrous, pyridine, rt, overnight; (d) TFA, DCM, r.t., overnight (e) (i) formaldehyde, triethylamine, MeOH, r.t., 1 h, (ii) NaCNBH_3_, r.t., overnight.

The synthesis started with an acid chloride-mediated ester formation to produce compound **12** (Scheme 1), while in other cases commercially available compounds such as methyl 3-fluoro-4-nitrobenzoate, 5-(methoxycarbonyl)-2-nitrobenzoic acid (Scheme 2), methyl 2-hydroxy-4-nitrobenzoate (Scheme 3), 1-cyclopropyl-3-fluoro-4-nitrobenzene and 1-ethoxy-3-fluoro-4-nitrobenzene (Scheme 4) were used as starting materials. In the next step, substituents were introduced to the hydroxy group of the core benzene ring via the Mitsunobu reaction to obtain compounds **13** (Scheme 1), **19g** (Scheme 2) and **25** (Scheme 3). For the preparation of compound **19h**, the substituent was introduced to the carboxyl group of the benzene ring by amide coupling using 1-hydroxybenzotriazole (HOBt) and 1-(3-dimethylaminopropyl)-3-ethyl-carbodiimide (EDC) as coupling reagents (Scheme 2). In the nucleophilic aromatic (**19a-f** and **19i-j**) and aliphatic (**19k-l**) substitutions, potassium carbonate was used to deprotonate the corresponding phenols, which were then reacted with the corresponding fluorobenzene for **19a-b** and **19i-j** or alkyl chloride for **19k-l** (Scheme 2). While in other cases potassium carbonate was used to neutralize the hydrofluoric acid which formed upon the reaction of the corresponding amines with the fluorobenzene (**19c-f**). Similarly, sodium hydride was used to deprotonate the aliphatic alcohol of *N*-Boc-4-hydroxypiperidine in the preparation of **31a-b** (Scheme 4).

Next, aminolysis of the methyl ester groups of **13**, **19a-l** and **25** with ammonia led to the corresponding carboxamides **14**, **20a-l** and **26**. This was followed by the reduction of the nitro group to an amine either by catalytic hydrogenation for **15**, **21a-i** and **27** or by using iron in acetic acid to produce **21j-l** (Schemes 1–3). 3,4-Dichloro-5-methyl-1*H*-pyrrole-2-carboxylic acid was then converted *in situ* to acid chloride and coupled to the previously prepared aromatic amines to give the intermediates **16a-d** (Scheme 1), **22a-h** (Scheme 2) and **28** (Scheme 3) and the final compounds **22i-l** (Scheme 2). To prepare compound **16e**, the nucleophilic hydroxyl groups of 2,4-dihydroxy-5-isopropylbenzoic acid were first protected by acetylation with acetic anhydride. The carboxyl group was then activated by oxalyl chloride to form acid chloride and subsequently reacted with amine **15**. After removal of the acetyl protecting groups, compound **16e** was obtained (Scheme 1). In the next step, the Boc protecting groups of **16a-e**, **22a-h** and **28** were cleaved off by acidolysis to prepare **17a-e**, **23a-h** and **29**. The latter, except **17e**, were methylated by reductive amination using formaldehyde to give the final compounds **18a-d**, **24a-h** and **30** as tertiary amines.

In Scheme 4, the nucleophilic aromatic substitution was followed by catalytic hydrogenation to reduce the nitro group of **31a-b** to the amine groups in **32a-b** and simultaneously open the cyclopropyl ring in **32a**. From here on, the synthesis proceeded analogously to that described above. First, the pyrrole-2-carboxylic acid was coupled with the amines **32a-b** to give **33a-b**. Then the Boc protecting group of **33a,b** was removed by acidolysis using TFA to prepare aliphatic secondary amines **34a,b**, which were converted to tertiary amines **35a,b** by reductive amination.

**Scheme 5.**
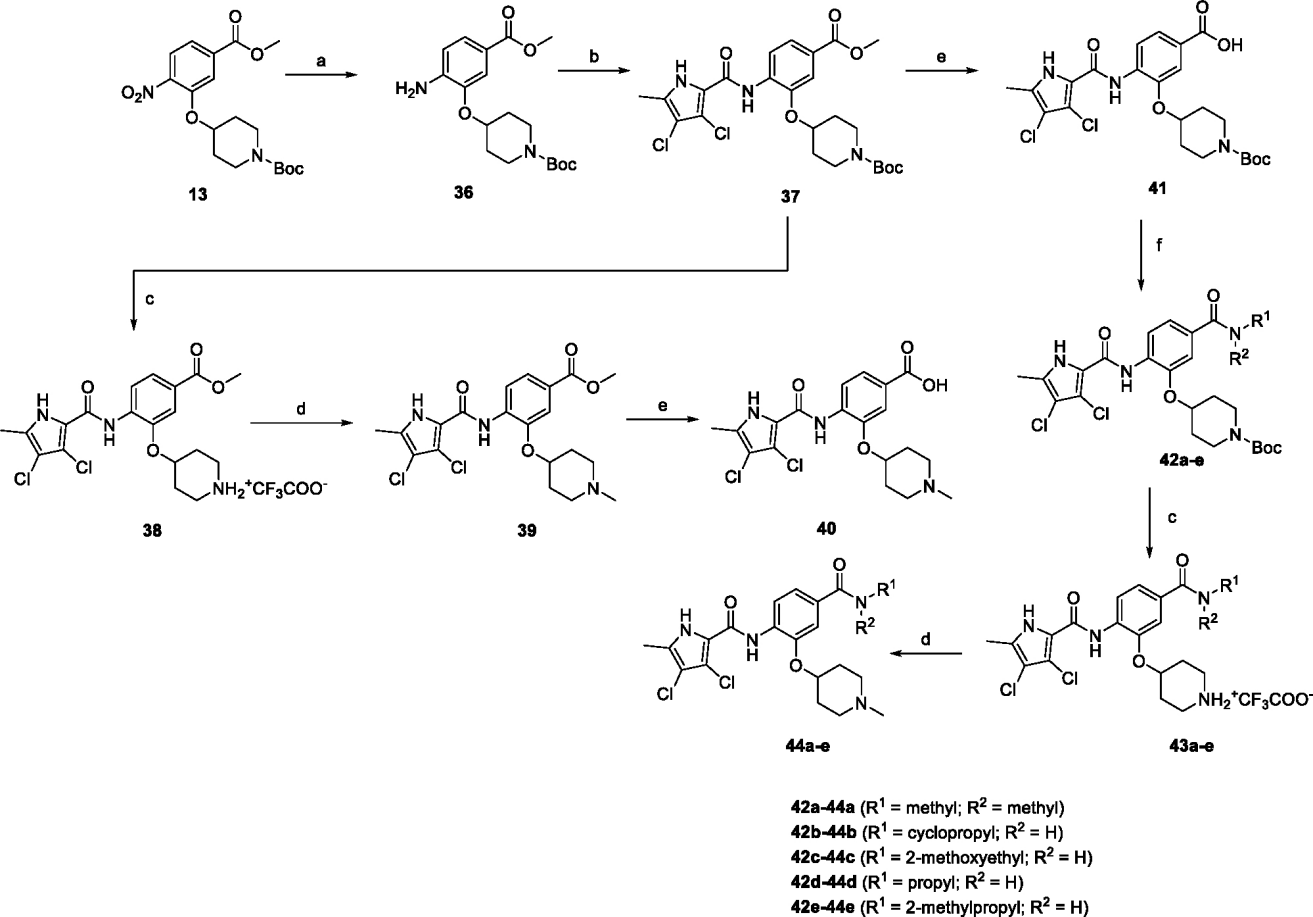
Reagents and conditions: (a) H_2_, Pd/C, MeOH, overnight; (b) (i) 3,4-dichloro-5-methylpyrrole-2-carboxylic acid, SOCl_2_, DCM anhydrous, 70 °C, 1 h; (ii) **36**, DCM anhydrous, pyridine, rt, overnight; (c) TFA, DCM, r.t., overnight; (d) (i) formaldehyde, triethylamine, MeOH, r.t., 1 h, (ii) NaCNBH_3_, r.t., overnight; (e) 1 M NaOH, MeOH/THF, 60 °C, 3 days; (f) (i) EDC, HOBt, DIPEA, DMF, 0 °C, 20 min; (ii) corresponding amine, r.t., overnight.

The first reactions in Scheme 5 proceeded in a similar order as in Scheme 4. First, the reduction of nitro to amino with catalytic hydrogenation gave compound **36**, which was then converted to the corresponding pyrrolamide **37**. The latter was then used in two different synthetic pathways. *N*-Boc protection was removed by acidolysis in TFA to give **38**, which was then methylated by formaldehyde and sodium cyanoborohydride to give the final compound **39**. The ester group of **39** was then hydrolyzed with sodium hydroxide to give carboxylic acid **40**. The ester group of **37** was hydrolyzed to yield **41**, which was then subjected to several amide couplings to prepare the amides **42a-e**. From here on, the procedure was analogous to other synthetic schemes. Acidolysis of the Boc protecting groups of **42a-e** with TFA to give amines **43a-e** was followed by methylation by reductive amination to give the final compounds **44a-e**.

**Scheme 6.**
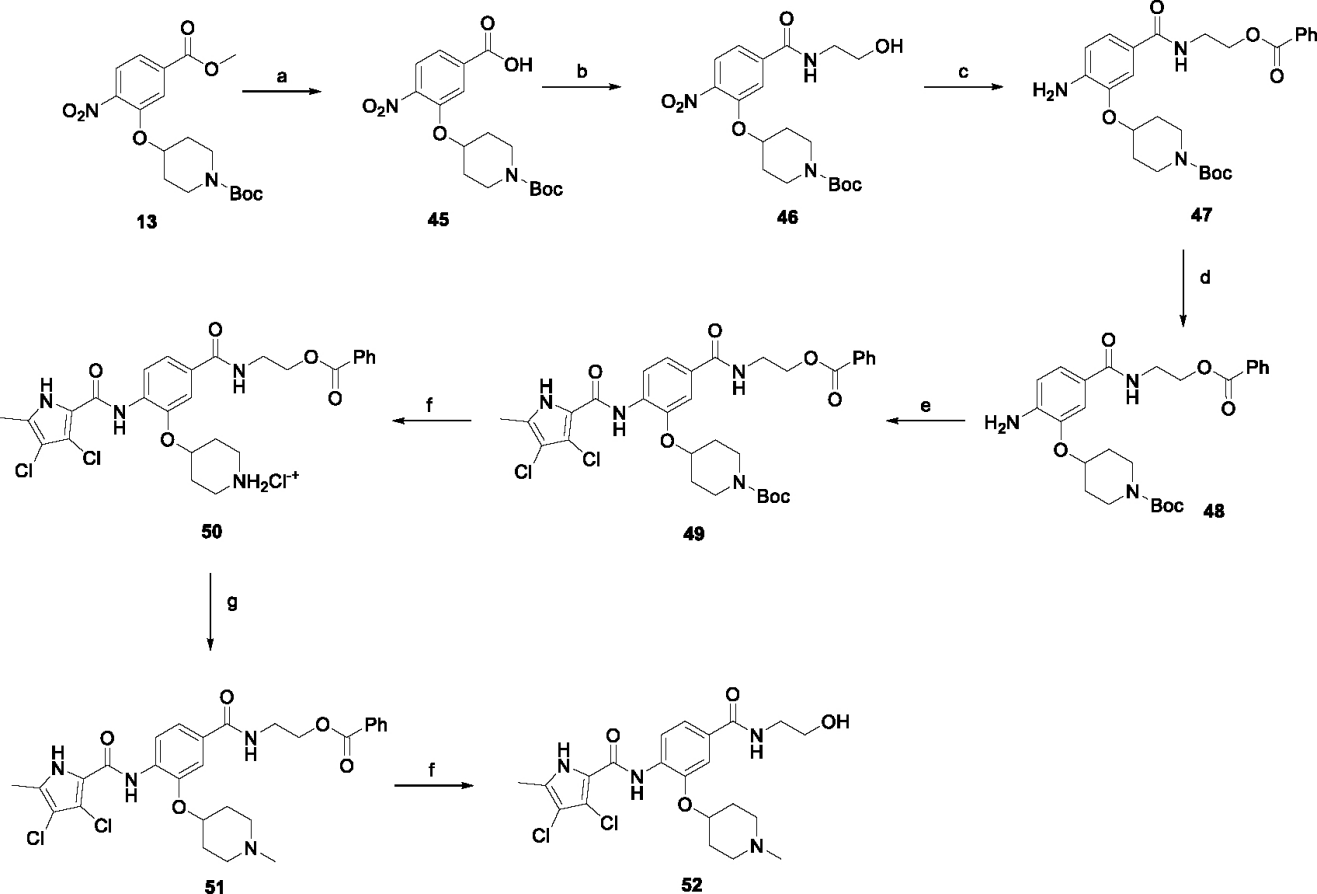
Reagents and conditions: (a) 1 M NaOH, MeOH/THF, 60 °C, overnight; (b) (i) EDC, HOBt, DIPEA, DMF, 0 °C, 20 min; (ii) 2-aminoethanol, r.t., overnight; (c) benzoyl chloride, K_2_CO_3_, dry THF, r.t., overnight (d) H_2_, Pd/C, MeOH, overnight; (e) (i) 3,4-dichloro-5-methylpyrrole-2-carboxylic acid, SOCl_2_, DCM anhydrous, 70 °C, 1 h; (ii) **48**, DCM anhydrous, pyridine, r.t., overnight; (f) 4 M HCl in 1,4-dioxane, 1,4-dioxane, r.t., overnight; (g) (i) formaldehyde, triethylamine, MeOH, 1 h, r.t., (ii) NaCNBH_3_, r.t., overnight; (g) 2 M NaOH, MeOH, rt, overnight.

Although compound **52** features a carboxamide substitution analogous to **44a-e**, the synthesis had to be carried out differently and is shown in Scheme 6. In the first step, the ester group of **13** was hydrolyzed to give **45**, which was then coupled with ethanolamine to give **46**. The aliphatic hydroxyl group of **46** was then protected with benzoyl chloride to form the ester **47**. Subsequently, the amine **48** was prepared by reduction of the nitro group of **47** and then coupled with the pyrrole-2-carboxylic acid chloride to give **49**. The Boc protecting group was removed with 4 M HCl in 1,4-dioxane to give the intermediate **50** in a salt form. In the penultimate step, compound **50** was *N-*methylated to give **51**, and finally the phenyl ester was hydrolyzed with sodium hydroxide solution to give **52**.

**Scheme 7.**
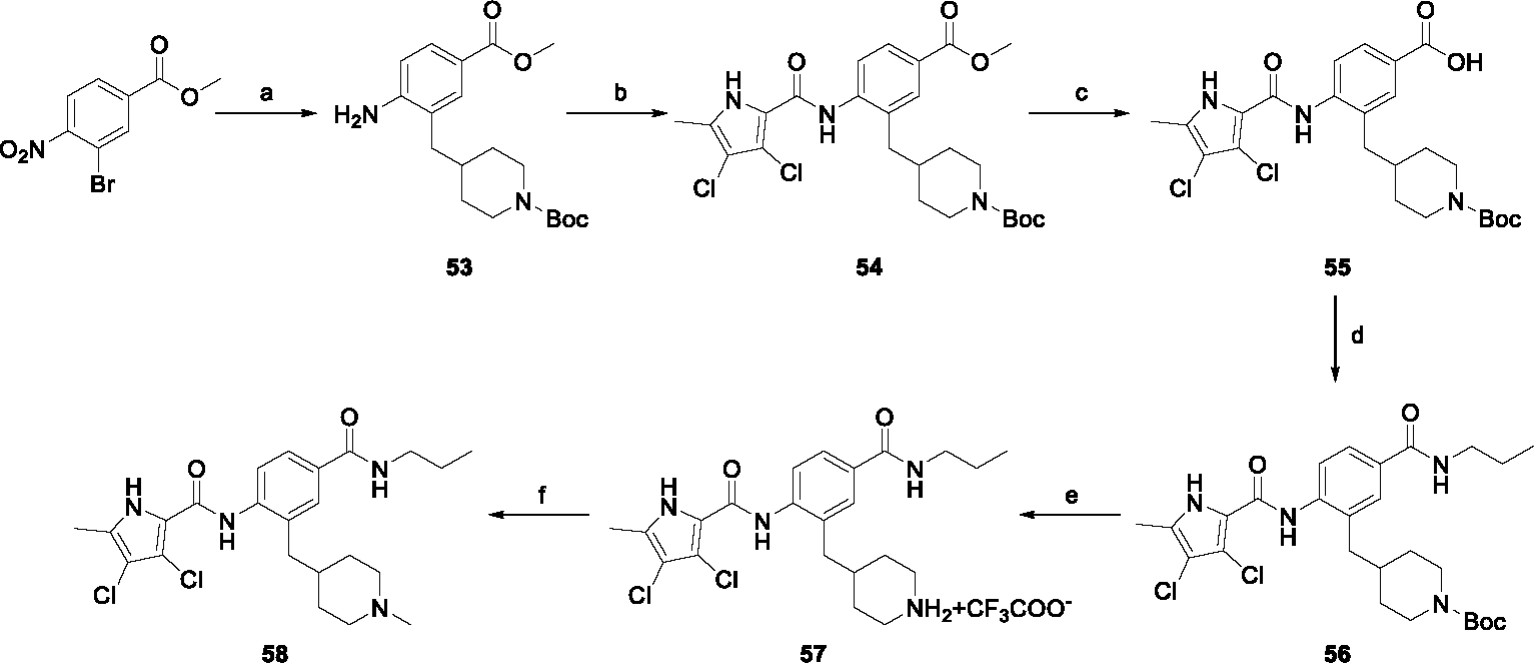
Reagents and conditions: (a) (i) 1-Boc-4-methylenepiperidine, potassium acetate, tetrakis(triphenylphosphine)-palladium, DMA, 120 °C, overnight; (ii) H_2_, Pd/C, MeOH, overnight; (b) (i) 3,4-dichloro-5-methylpyrrole-2-carboxylic acid, SOCl_2_, DCM anhydrous, 70 °C, 1 h; (ii) **53**, DCM anhydrous, pyridine, rt, overnight; (c) 1 M NaOH, MeOH/THF, 60 °C, overnight; (d) (i) EDC, HOBt, DIPEA, DMF, 0 °C, 20 min; (ii) propylamine, r.t., overnight; (e) TFA, DCM, r.t., overnight; (f) (i) formaldehyde, triethylamine, MeOH, r.t., 1 h, (ii) NaCNBH_3_, r.t., overnight.

In the first step of the synthesis of **58**, a Heck coupling was performed to synthesize a C–C bond at position 3 of the core phenyl ring using 1-Boc-4-methylenepiperidine as reagent (Scheme 7). After Heck coupling, catalytic hydrogenation was performed to reduce the nitro group to an amine group and saturate the double bond. Compound **53** was then subjected to the amide bond formation with the *in situ* formed 3,4-dichloro-5-methyl-1*H*-pyrrole-2-carbonyl chloride to yield amide **54**. The methyl ester group of **54** was then hydrolyzed to prepare **55**, which was then converted to **56** using EDC and HOBt as coupling reagents and propylamine as nucleophile. TFA was then used to form the secondary amine **57** in the form of a TFA salt. In the final step, compound **57** was methylated to produce the final compound **58**.

### 2.3. Evaluation of on-target activities against Hsp90β, Hsp90α and TopoIIα

All final compounds were analyzed biochemically for their inhibition of TopoIIα and their binding affinities for Hsp90β and its most similar paralog Hsp90α. For TopoIIα, the results are presented as the percentage of inhibited enzyme determined by a DNA relaxation assay when the protein was exposed to the compound at a concentration of 100 μM. This assay was performed to evaluate the selectivity of the compounds for the cytoplasmic Hsp90 protein family members. On the other hand, the dissociation constants (*K*_d_) of the final compounds with Hsp90α or Hsp90β N-terminal ATP-binding domain were determined using the fluorescence-based thermal shift assay (FTSA). Due to the solubility limitations of the compounds, the assay range for the determination of *K*_d_ values was mostly limited to the maximum tested concentration of 200 μM.

As we concluded from the compounds shown in Table 1, the replacement of the 3,4-dichloro-5-methylpyrrole moiety has a crucial role in selectivity and affinity for Hsp90β. To investigate this further, the 2,4-dihydroxy-5-isopropylphenyl group that is commonly found in resorcinol-based Hsp90 inhibitors [23] was introduced in place of the 3, 4-dichloro-5-methylpyrrole moiety. As expected, replacing the pyrrole in compound **11** with the resorcinol derivative resulted in a significantly increased affinity for both Hsp90 isoforms (Table 2, compound **17e**). However, this substitution rendered **17e** as a ligand with a more than three-fold preference for Hsp90α compared to Hsp90β, highlighting the importance of the appropriately substituted pyrrole for Hsp90β selectivity. Furthermore, methylation of the basic amino group on the substituent at position 2 of the phenyl ring in compound **11** resulted in **18a**, which still exhibited some Hsp90β selectivity, while binding both cytoplasmic Hsp90s approximately 5-times more tightly than **11** (Table 2). In contrast, compounds **18b-d**, in which the pyrrole was replaced by different indole rings, bound neither Hsp90α nor Hsp90β (Table 2), again demonstrating the importance of a suitable substituent at this position. Regardless of whether the effect of 3,4-dichloro-5-methylpyrrole on affinity and selectivity is due to its ideal size or its slightly more acidic nature that allows for stronger hydrogen bonding with Asp88 [45] (Fig. 2C), its positive effect in this series is evident. Therefore, we decided to retain both the 3,4-dichloro-5-methylpyrrole and a tertiary amine in the following optimization steps.

Next, changes were made to the *meta* (R^1^) or *ortho* (R^2^) position with respect to the pyrrole-2-carboxamide group (Table 3). Firstly, compounds **22i-k** containing various non-ionizable aromatic rings at position 2 did not bind to any of the cytoplasmic Hsp90 isoforms. Conversely, when a weakly basic pyridine was introduced at this position, weak binding of **22l** was observed. Some SAR information can also be drawn from the library of aliphatic tertiary amines **24a-h**. Compounds **24a** and **24b**, which contain an aromatic linker connecting the benzene core to the basic center, showed no Hsp90 binding in concentrations up to 200 μM. In contrast, inhibitors **24c**, **24d**, **24e** and **24g** with aliphatic nitrogen-containing heterocycles in the *meta* position are the most potent and selective Hsp90β ligands of the series. For example, compounds **24d** and **24g** bind to Hsp90β with a similar affinity and are more than 9- and 14-fold selective over Hsp90α, respectively. In addition, inhibitors **24c** and **24e** are almost as potent as **18a**. However, they show a complete absence of binding to Hsp90α at 200 μM, making them more than 30- (**24c**) and 27-fold (**24e**) selective for the Hsp90β paralog. The selectivity for these ligands in our assay is even greater than that of previously reported Hsp90β-selective compound KUNB-31 (23-fold). Overall, the results show that the position of the amine group can be varied to some extent, while excessive rigidification (**24h**), different spatial orientation (**24f**) and a shift of the piperidine to the *meta* position (**30**) are detrimental for Hsp90 binding.

Finally, we studied different modifications of the carboxamide group at position 4 of the core benzene ring (R^1^, Table 4). Most compounds that featured the secondary or tertiary carboxamide group retained at least some Hsp90β affinity (**44a-e**, **52**). The affinity differences compared to the parent compound **18a** were more drastic when the carboxamide was replaced entirely. In the case of **35a** and **35b**, more lipophilic, slightly larger and more flexible chains were attached directly to the phenyl core, leading to a decrease in affinity and selectivity. On the other hand, the methyl ester in **39** and the carboxylic acid in **40** also led to decrease in affinity in comparison with **18a**.

In contrast, the incorporation of short hydrophobic chains to form various secondary and tertiary amides (**44a**-**b** and **44d**-**e**) had little effect on Hsp90β affinity. The introduction of a methoxyethyl substituent at the carboxamide group in **44c** improved the selectivity to some extent, while 2-hydroxyethyl in **52** improved the affinity but reduced selectivity for Hsp90β. In compound **58**, the piperidine ring was bound via a carbon-carbon bond, as the ether oxygen was not predicted to form important interactions in the binding site (Fig. 2C). Unfortunately, compound **58** did not bind to either of the cytoplasmic Hsp90 isoforms.

All 29 final compounds listed in Tables 2–4 were also screened for TopoIIα inhibition at 100 μM and most compounds did not inhibit TopoIIα. The only TopoIIα inhibitors were compounds **44b**, **44d** and **44e** for which IC_50_ values were determined (Table 4, Fig. S34). In all three compounds the carboxamide nitrogen is substituted with a lipophilic group. This observation is consistent with the predicted binding mode of **11** (Fig. 2B) showing that the carboxamide group is oriented towards the hydrophobic pocket in TopoIIα binding site. Meanwhile, the most potent and selective Hsp90β ligands, **24c** and **24e**, did not inhibit TopoIIα. However, since simultaneous inhibition of Hsp90β and TopoIIα may have synergistic effects when evaluated in cancer cells, all new compounds with affinity for Hsp90β (*K*_d_ < 200 μM) were selected for evaluation of their antiproliferative activity in cancer cells.

### 2.4. Viability assessment in cancer cell lines

**Fig. 4.**
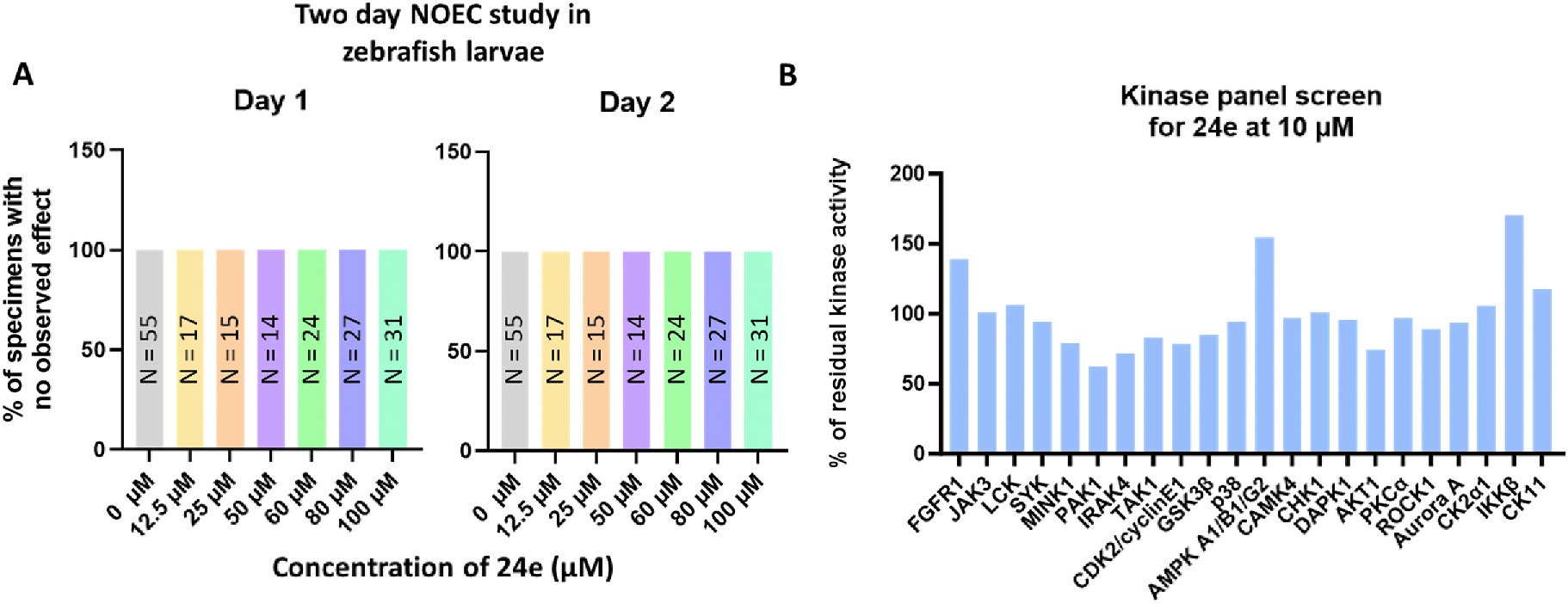
Graphical representation of antiproliferative IC_50_ values (mean ± SD) determined for all compounds that bind to Hsp90β with *K*_d_ values lower than 200 μM in two biological repetitions each performed in triplicate.

Based on the above selection criteria, compounds **17e**, **18a**, **22l**, **24c-e**, **24g**, **35a-b**, **39**, **40**, **44a-e** and **52** were selected for evaluation of their antiproliferative activity in breast cancer cell line MCF-7 by MTS assay (Fig. 4). Initially, the selected inhibitors were screened at a concentration of 50 μM. Compounds **17e**, **22l**, **24c**, **40**, **44c**, and **52** showed little to no growth inhibition of MCF-7 cells (IC_50_ > 50 μM) in spite of their binding to Hsp90. These results suggest that other factors, such as poor membrane permeability, may lead to a lack of cellular activity despite good on-target binding affinities.

Hsp90β selective inhibitor **24e** displayed antiproliferative activity against MCF-7 cells with an IC_50_ value of 14.2 ± 2.1 μM. Similar growth inhibition was also observed for compounds **24d** and **18a**, while an improvement in cellular potency was observed for the remaining Hsp90 inhibitors tested. For example, compound **24g**, which is a slightly weaker but still selective Hsp90β inhibitor, inhibited the growth of MCF-7 breast cancer cells in the low micromolar range (IC_50_ = 2.6 ± 0.0 μM). The strongest growth inhibitor of MCF-7 cells was the methyl ester carboxamide analog **39** (IC_50_ = 0.94 ± 0.06 μM), followed by the clinically evaluated 17-DMAG (positive control) and inhibitors **44d**, **35b**, **35a**, **44b**, **44e** and **44a** in that order. Compounds **44a-e** that are relatively strong binders of both Hsp90α and Hsp90β also display some residual TopoIIα inhibition, which could explain their potent *in vitro* anticancer activity. On the other hand, the results of weak Hsp90β inhibitors **35a** and **35b** are somewhat surprising, as their potential for MCF-7 growth inhibition substantially exceeds their on-target activity.

Based on these results, some of the most potent inhibitors of MCF-7 cell growth – namely, **35a-b**, **39**, **44a-b** and **44d-e** – along with the most Hsp90β-selective inhibitor, **24e**, were advanced to screening in various cancer cell lines of the NCI-60 (National cancer institute – 60 cell line) panel [46]. The order of potency in the initial screening was consistent with the IC_50_ values in MCF-7 cells, indicating greater potency of compounds **35a-b**, **39**, **44a-b** and **44d-e** compared to the most Hsp90β-selective compound **24e** (Figs. S21–S28). Therefore, these six inhibitors were progressed to IC_50_ determination. The results suggest that all examined compounds have broad anticancer potential as low micromolar to high nanomolar GI_50_ (growth inhibition 50 %) values were determined in all tested cancer types including leukemia, melanoma, non-small cell lung cancer, colon cancer, central nervous system (CNS) cancer, ovarian cancer, renal cancer, breast cancer and prostate cancer (Tables S3 and S4). This is consistent with the omnipresence of Hsp90 [1,21,47]. On the other hand, compound **24e** was able to slow the growth of most cancer cell lines by about 50 % at 10 μM screening (Fig. S21), but the potency did not meet the criteria for further GI_50_ determination at NCI. Therefore, we have additionally evaluated compound **24e** in leukemia cell line K562 and Ewing sarcoma cell line SK-N-MC and obtained IC_50_ values of 21.8 ± 5.5 μM and 28.5 ± 0.6 μM, respectively (Table S5). Even though **24e** was not highlighted as the most potent inhibitor of cancer cell growth, we have selected it for further biological evaluation, to better understand the changes caused by its Hsp90β affinity and selectivity.

### 2.5. Solubility determination of selected compounds

It has been reported that compounds containing the 3,4-dichloro-5-methylpyrrolamide moiety can exert solubility issues [39]. Therefore, we decided to evaluate the thermodynamic (TD) solubility of selected compounds in PBS buffer (pH = 7.4) at 37 °C. The TD solubility of representative compounds was determined by HPLC as previously described for the analogous TopoIIα inhibitors [39]. We selected two of the most promising Hsp90β selective inhibitors **24e** and **44d** that also exhibited *in vitro* anticancer activity. In addition, **24c** and **52** were tested to further investigate their unexpected lack of antiproliferative activity. Unexpectedly, compound **24c** (TD solubility <1 μM) is poorly soluble which may be attributed to higher rigidity of the piperazine ring directly bound to the core benzene ring. The solubility of **44d** (TD solubility = 17.0 ± 3.4 μM) and **52** (TD solubility = 5.1 ± 0.9 μM) is low to moderate, similar to the solubility of their TopoIIα-targeting predecessors [39]. The TD solubility of **24e** (TD solubility = 310 ± 10 μM) was the highest among the evaluated compounds. In addition to the remarkable selectivity for Hsp90β and activity in MCF-7 breast cancer cells, the favorable solubility highlights compound **24e** as one of the most promising compounds of the series.

### 2.6. Preliminary safety profile and additional assessment of 24e

First, the *K*_d_ value of **24e** with Hsp90βN was re-evaluated using isothermal titration calorimetry (ITC) to confirm the value obtained by FTSA. Indeed, endothermic binding of **24e** to Hsp90βN was determined with a *K*_d_ value of 5.2 ± 0.7 μM (Fig. S19) which is in line with our previous findings (*K*_d_ = 7.3 – CI:[4.8; 11] μM).

**Fig. 5.**
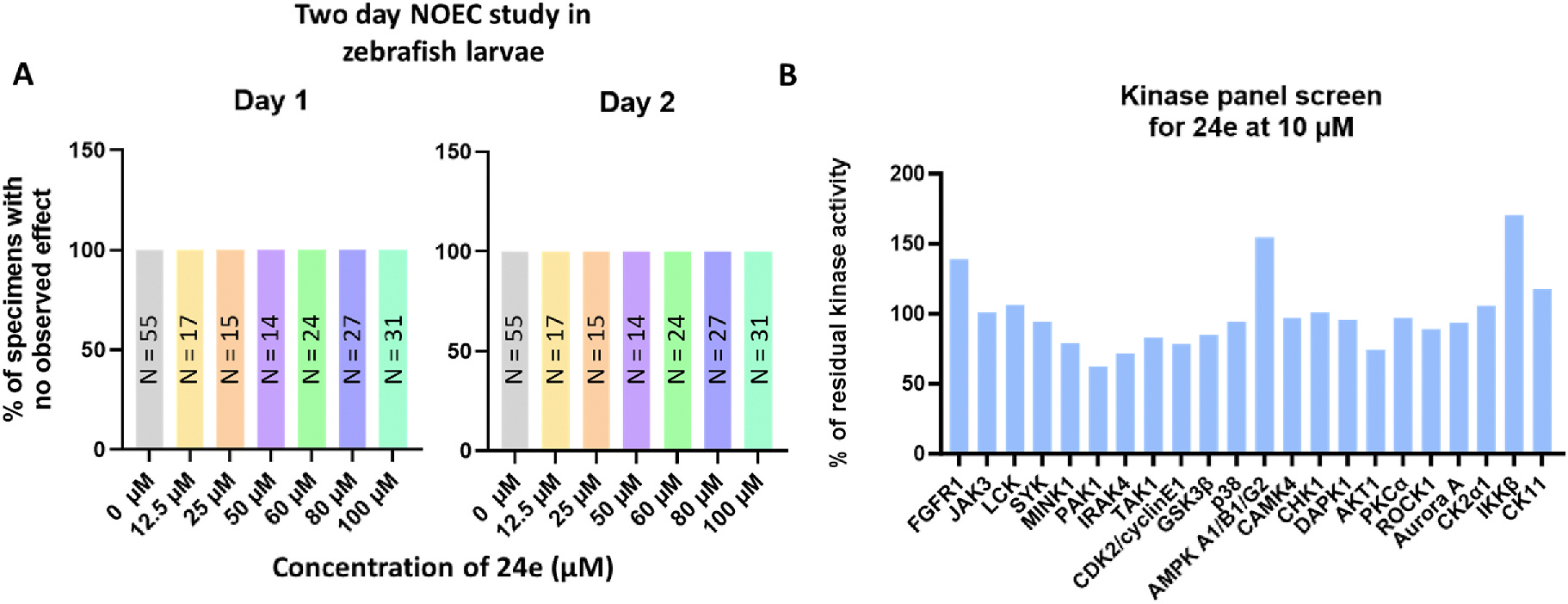
**A**) Graphical representation of a NOEC determination study in zebrafish larvae for compound **24e**. None of the specimens included in the study were affected by the inhibitor at concentrations up to 100 μM. The number of fish included in the study of individual concentrations are indicated on the bars; **B**) Graph representing residual kinase activity of compound **24e** when screened for inhibition at 10 μM.

Previously, Hsp90 inhibitors have been shown to be potentially susceptible to unwanted side effects in healthy cells [48]. Therefore, the toxicological profile of **24e** was evaluated in a zebrafish toxicology model where the highest no observed effect concentration (NOEC) was determined. As can be seen from the graph (Fig. 5A) the fish were exposed to increasing doses of **24e** for two days starting near the antiproliferative IC_50_ value determined in MCF-7 breast cancer cell line (12.5 μM). As no toxic effects in the fish were detected, the concentrations were gradually increased to 100 μM. All fish survived with no observed effect at this high concentration as well. None of the specimens showed any signs of toxicity in neither day 1 nor day 2 of the study indicating that concentrations up to 100 μM were not toxic for the zebrafish larvae, but this could potentially be influenced by poor uptake in the fish. However, calculated physicochemical properties of **24e** (MW = 450.37; clogP = 3.05; HBD = 3; HBA = 2; tPSA = 94.46 Å; number of rotatable bonds = 6) (LigandScout 4.5 Expert Available from Inte:Ligand GmbH) [49] are in agreement with characteristics derived from the set of zebrafish-absorbed compounds (MW ≤ 500; clogP ≤5.3; HBD ≤3; HBA ≤7; tPSA ≤124 Å; number of rotatable bonds ≤9). This suggests that **24e** is most likely absorbed by zebrafish and is truly biologically inert [50].

Furthermore, since **24e** and its analogs target the ATP-binding pocket of Hsp90, it is possible that they also compete with ATP for binding to other proteins. However, this is somewhat less likely because the Bergerat fold in the ATP-binding pocket of Hsp90 is specific for the GHKL family of proteins [23,36]. Nonetheless, the potential for off-target binding of Hsp90 inhibitors exists [51], and therefore **24e** was tested at a concentration of 10 μM against 22 representative protein kinases. From the results shown in Fig. 5B it can be seen, that **24e** at 10 μM reduces the kinase activity of the PAK1, IRAK4 and AKT1 kinases, by 37.4 %, 28.5 % and 25.8 %, respectively. Meanwhile, the inhibition of the remaining 19 kinases of the panel was not higher than 25 % (Table S2). Compared to treatment with the pan-kinase inhibitor sorafenib (10 μM, Fig. S20), the inhibition rates of **24e** are significantly lower. The absence of kinase inhibition, along with high NOEC determined in zebrafish larvae qualified **24e** for further studies.

### 2.7. Compound 24e induces apoptosis and causes cell cycle arrest in G0/G1 phase in MCF-7 cells

**Fig. 6.**
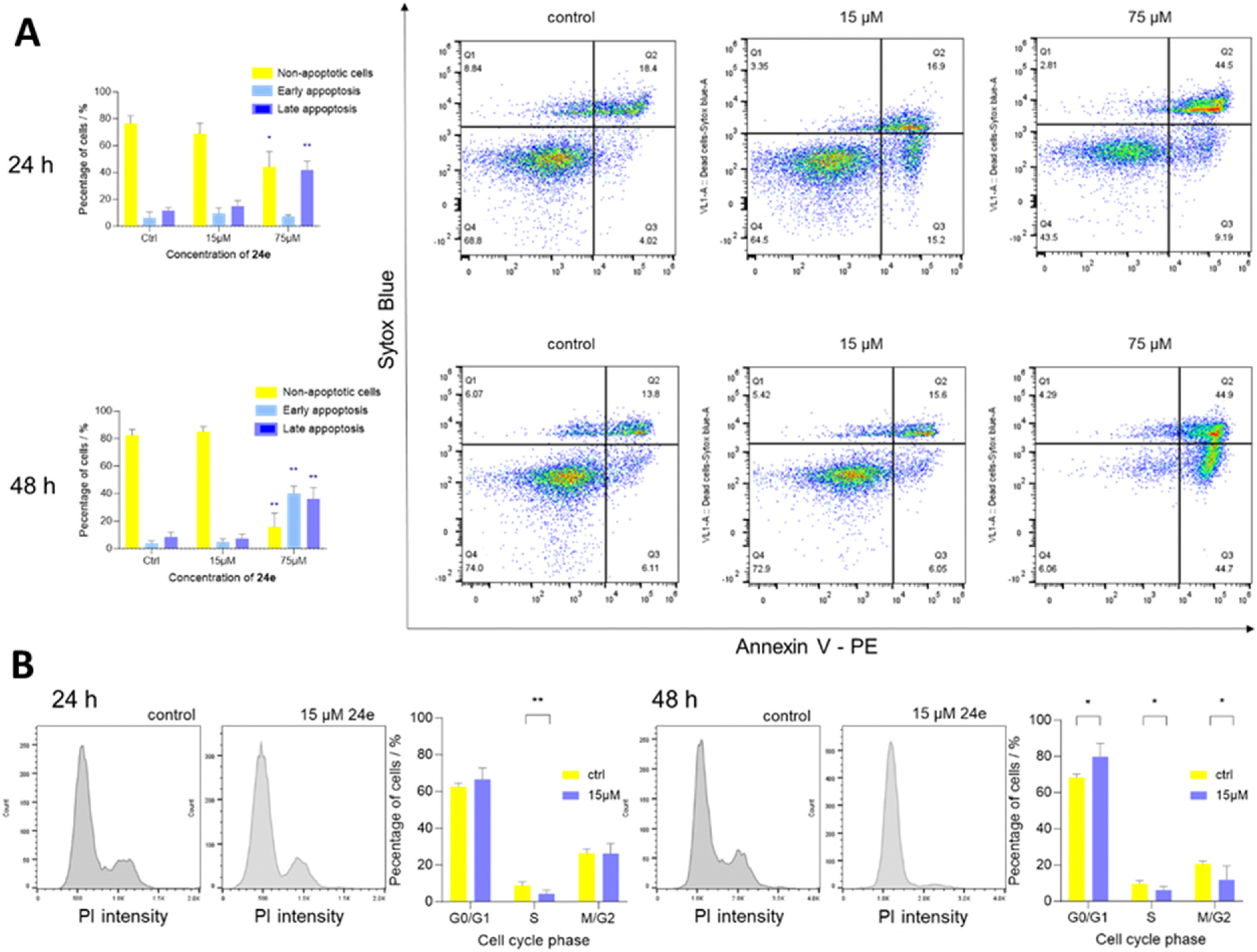
Induction of apoptosis (A) and cell cycle arrest (B) in MCF-7 cells by compound **24e**. MCF-7 cells were exposed to 15 μM or 75 μM **24e**. After 24 h or 48 h, apoptosis was measured by annexin V-PE/Sytox Blue staining, and cell cycle progression was assessed by propidium iodide. A) Percentages of viable (non-apoptotic), early apoptotic and late apoptotic cells – left, and representative scatter plots of annexin V-PE (x-axis) and Sytox blue (y-axis) – right. B) Representative cell cycle graphs and percentages of cells in each phase of the cell cycle. Data are presented as the means ± SD of at least two biological replicates. *p < 0.05, **p < 0.01 (Person’s *t*-test): treatment vs. control at time point.

Several Hsp90 inhibitors have been associated with degradation of key signalling molecules involved not only in cell proliferation but also in apoptosis and cell cycle progression [52,53]. We thus aimed to elucidate the mechanism of cytotoxicity of compound **24e** (Fig. 6). MCF-7 cells were subjected to treatment with either vehicle control or compound **24e** at concentrations of 15 μM and 75 μM over a period of 24 and 48 h. The extent of apoptosis was quantified using the Sytox Blue/annexin V assay. Our results revealed that compound **24e** at a concentration of 75 μM triggered apoptosis in MCF-7 cells already after 24-h treatment, with 50.1 % of cells undergoing apoptotic cell death compared to 17.6 % in control cells. Extending the treatment duration to 48 h enhanced the apoptotic response to compound **24e** in MCF-7 cells. The treatment with 75 μM compound **24e** increased the percentage of cells in early apoptosis (40.1 % vs 3.8 % for the control) and late apoptosis (36.2 % vs 8.6 % for the control). Lower concentration of compound **24e** (15 μM) did not significantly alter the externalisation of phosphatidyl serine, a measured marker of apoptosis.

Building on the observed pro-apoptotic activity of compound **24e** on MCF-7 cells, we further investigated its effect on the cell cycle at sub-apoptotic concentrations using propidium iodide (PI). Cells were treated with 15 μM compound **24e** for 24 or 48 h. We noticed that 15 μM of compound **24e** induced a notable arrest in G0/G1 phase of the cell cycle. After 24-h treatment, the first effects were already visible and after 48-h exposure they were significantly displayed (80.0 % vs 68 % for the control). On the other hand, S and G2/M phases were slightly but significantly decreased (6.2 % vs 9.7 % for the control and 11.6 % vs 20.6 % for the control, respectively). The arrest in the G0/G1 phase of the cell cycle suggests that compound **24e** might interfere with the mechanisms regulating the transition from G1 to S phase, which is corroborated by similar findings in studies with other Hsp90 inhibitors, such as 17-AAG [54]. This could involve the regulation of cyclin-dependent kinases (CDKs), as Hsp90 is known to interact with several proteins involved in cell cycle regulation, among them also CDKs [55]. Thus, the inhibition of Hsp90 could lead to disruption of these interactions, leading to cell cycle arrest in the G0/G1 phase.

### 2.8. Reduction of intracellular levels of Hsp90-dependent client proteins by 24e

**Fig. 7.**
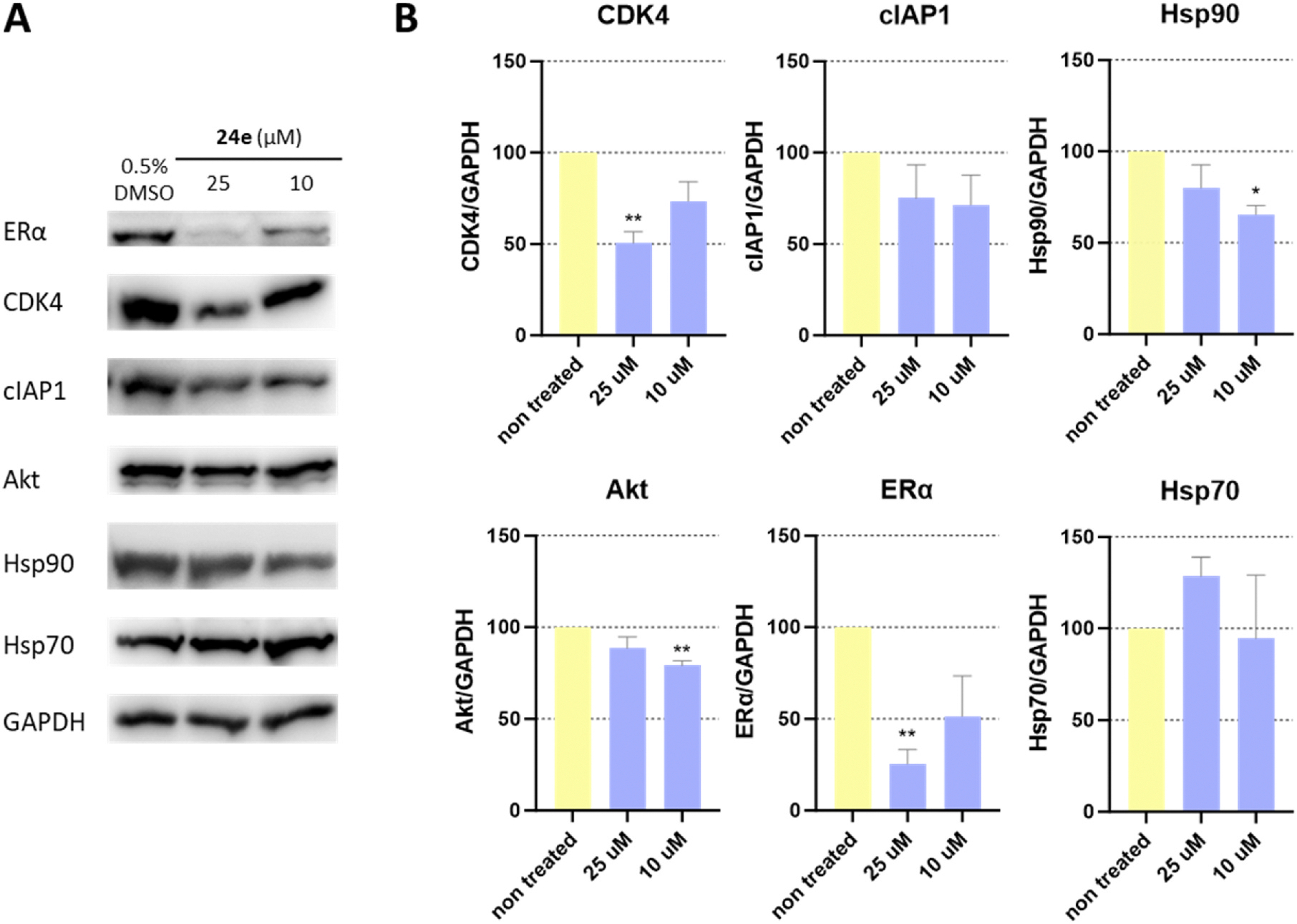
**A**) Representation of western blots of **24e** in MCF-7 breast cancer cell line where levels of ERα, CDK4, cIAP1, IGF1R, Akt (Hsp90 clients) and Hsp90, Hsp70 (HSR) were monitored. **B**) The protein levels were normalized and quantified in relation to GAPDH levels and the results are presented as bar graphs for each of the evaluated proteins. The bars represent mean values with SD. Unpaired *t*-test was applied to determine statistical significance (*p < 0.05, **p < 0.01). The images used for quantification are presented in Supplementary information in Figs. S29 and S30.

Inhibition of Hsp90 leads to a reduction in Hsp90 client protein levels, as they are unable to adopt their correct conformation and are consequently degraded via the ubiquitin-proteasome pathway. Therefore, we investigated the effects of **24e** on protein levels in the MCF-7 breast cancer cell line by Western blot analysis (Fig. 7). Well established Hsp90 clients like cyclin-dependent kinase 4 (CDK4), estrogen receptor α (ERα), insulin growth factor 1 receptor (IGF1R), cellular inhibitor of apoptosis protein 1 (cIAP) and protein kinase B (Akt) were selected for evaluation along with Hsp90 and Hsp70 to also assess the presence of HSR. After incubation with 25 and 10 μM concentrations of **24e** for 24 h, the intracellular levels of all client proteins were at least slightly reduced. Perhaps most importantly, the concentration of CDK4, a previously reported Hsp90β-dependent client protein [9], was reduced in a statistically significant in a dose-dependent manner. The effect was even more pronounced in the case of ERα, while the reduction in the levels of cIAP1, Akt and IGF1R was weaker but notable. We also observed a slight change in the levels of Hsp90 and Hsp70, which were slightly reduced and increased, respectively. Importantly, the decrease in Hsp90 levels at 10 μM was significant, while the increase in Hsp70 at 25 μM shows a statistically insignificant trend. This suggests that the Hsp90β-selective inhibitor **24e** is able to statistically significantly reduce intracellular levels of Hsp90-dependent client proteins without simultaneously inducing the HSR.

### 2.9. Modeling of compound 24e binding to Hsp90β

#### 2.9.1. Molecular dynamics simulation of 24e with Hsp90β

The binding mode of compound **24e** in the NTD of Hsp90β was modelled by a combination of molecular docking and MD simulations. In a recent study, X-ray co-crystal structures of all Hsp90 isoforms in complex with the purine ligand 6DMP were solved, shedding light on conformational flexibility and conserved water networks within the Hsp90 family [56]. A network of four conserved water molecules (referred to as A-D) was identified in all four 6DMP structures (Fig. S4). Notably, in the Hsp90α−6DMP structure, a unique Ser52 in the SAAA site (Hsp90α: Ser52, Hsp90β: Ala47, Grp94: Ala108, TRAP1: Ala120) forms an additional hydrogen bond with water B [56]. The displacement of water molecules A and B in the Hsp90β structure was proposed as a significant factor in achieving selectivity for the Hsp90β isoform [8]. Another crucial difference between Hsp90α and Hsp90β lies in the ILVI site (Hsp90α: Ile91, Hsp90β: Leu86, Grp94: Val147, TRAP1: Ile156) [56]. Introduction of substituents that sterically clash with bulkier Ile91 in Hsp90α serves as another mechanism for achieving selectivity towards Hsp90β [8,9].

**Fig. 8.**
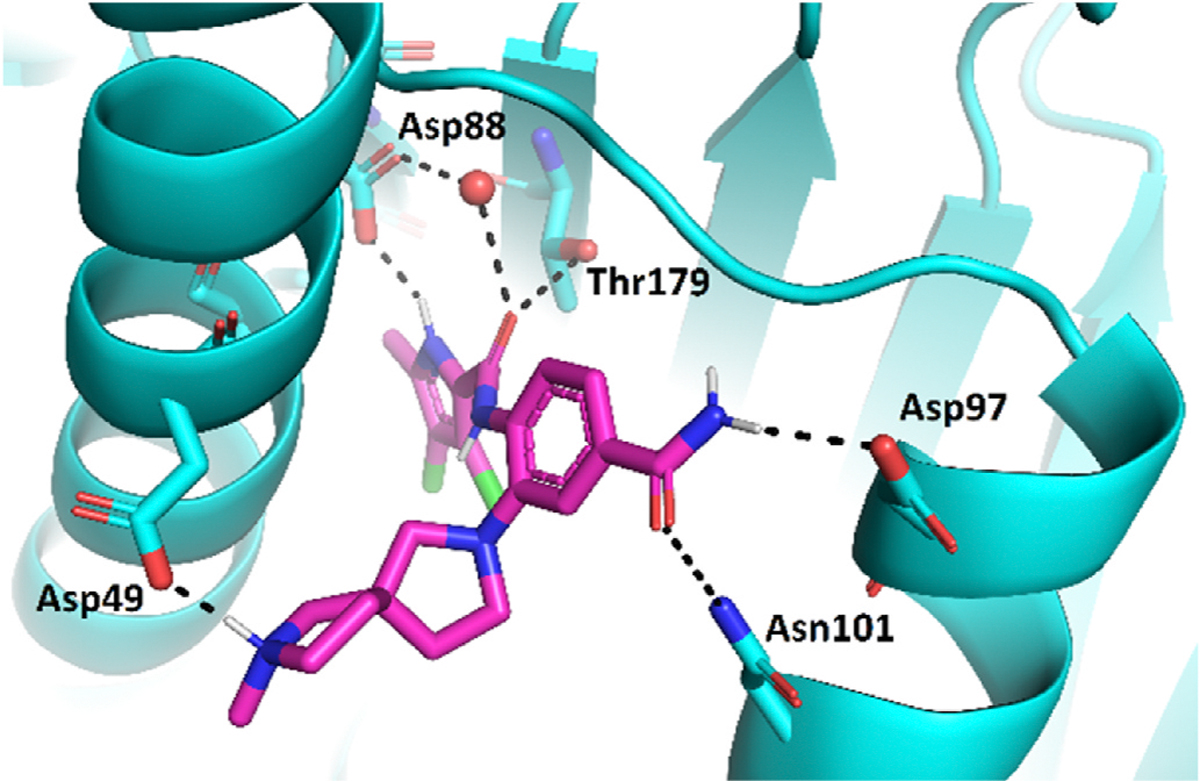
Docking binding mode of compound **(*R*)-24e** (in magenta sticks) in the ATP-binding site of Hsp90β (in cyan cartoon, PDB entry: 5UCJ). For clarity, only selected amino acid residues are presented as sticks. Hydrogen bonds are presented as black dashed lines. Structural water molecule is shown as a red sphere.

The virtual screening hits (Table 1) were identified by docking the compound library to the Hsp90β structure in complex with a selective inhibitor of this isoform (PDB entry: 5UCJ [8]), in which water molecules A and B were displaced upon inhibitor binding. Similarly, both enantiomers of compound **24e** were initially docked to the ATP-binding site of Hsp90β. The best ranking docking pose of (*R*)-**24e** (Glide score: 8.1) (Fig. 8) and (*S*)-**24e** (Glide score: 7.6) in complex with Hsp90β was then used in a 500 ns MD simulation to investigate the stability and interactions of the complex. (*R*)-**24e** showed a favorable stability of the docking complex (Fig. S5). Importantly, the flexibility of the substituent bearing the basic tertiary amine in the binding site was significantly reduced (Fig. S6) compared to the results of initial hit compound **11** (Fig. S2), leading to an ionic interaction with the Asp49 side chain for 85 % of the simulation time (Fig. S7). The binding mode of (*S*)-**24e** was less favorable, as the tertiary amine lost contact with Asp49 and did not form interactions with the binding site (Figs. S8–S10).

To investigate the binding of (*R*)-**24e** to the ATP-binding site of Hsp90α (PDB entry: 2XAB), we used two docking protocols. In the first, we retained water molecules A, B and C in the Hsp90α structure and then docked (*R*)-**24e**. The orientation of the ligand was completely different from that predicted in the Hsp90β structure. Namely, (*R*)-**24e** was oriented in such way that the carboxamide on the phenyl ring formed hydrogen bonds with Asp93 and water molecule C (Fig. S11). In the second docking protocol, waters A and B were removed from the Hsp90α structure prior to the docking of (*R*)-**24e**, resulting in a similar binding mode as in the case of Hsp90β. This binding mode of (*R*)-**24e** was stable in the MD simulation (Fig. S12) and a similar interaction network was formed (Fig. S13) as in the case of Hsp90β (Fig. S7).

Since no steric clashes with the Ile91 side chain in Hsp90α were predicted, we postulated that the difference in the SAAA site of Hsp90α and Hsp90β is the most critical driving factor for the selectivity of **24e** and its analogs. When the docking conformation of (*R*)-**24e** from Hsp90β was inserted into the binding site of Hsp90α, a clear overlap of the methyl group on the pyrrole moiety with the water molecule B was observed (Fig. S14). To confirm that the replacement of Ser52 in Hsp90α by Ala47 in Hsp90β was indeed responsible for the selectivity of **24e**, we prepared an S52A mutant of Hsp90α. To validate the mutant protein, we first tested KUNB-31, and its affinity for the Hsp90αN S52A mutant was better than for the wild-type Hsp90α with respective *K*_d_ values of 0.25 [0.23; 0.27] μM and 0.61 [0.50; 0.74] μM (Table 1). This mutation resulted in an even more significant spike in binding affinity of **24e**, as it bound Hsp90α S52A mutant with a *K*_d_ value of 9.5 ± 0.4 μM, while no binding to wild-type Hsp90α was detected (Table 3). Moreover, the affinity of **24e** to Hsp90α S52A mutant was comparable to that of Hsp90β (*K*_d_ = 7.3 CI:[4.8; 11] μM). Additionally, FTSA data for several newly synthesized compounds (**17e**, **18a**, **24g**, and **44e**) binding to the Hsp90αN S52A mutant demonstrated that both the Hsp90αN S52A and Hsp90βN proteins bound most of the tested compounds similarly, confirming that this single residue change at position 52 was sufficient to alter selectivity (Table S1). This underscores the significance of the Ser52-to-Ala52 switch in the binding of Hsp90β-selective compounds and identifies this residue as potentially the most crucial factor for achieving Hsp90β selectivity.

#### 2.9.2. Saturation transfer difference NMR of 24e with Hsp90β

**Fig. 9.**
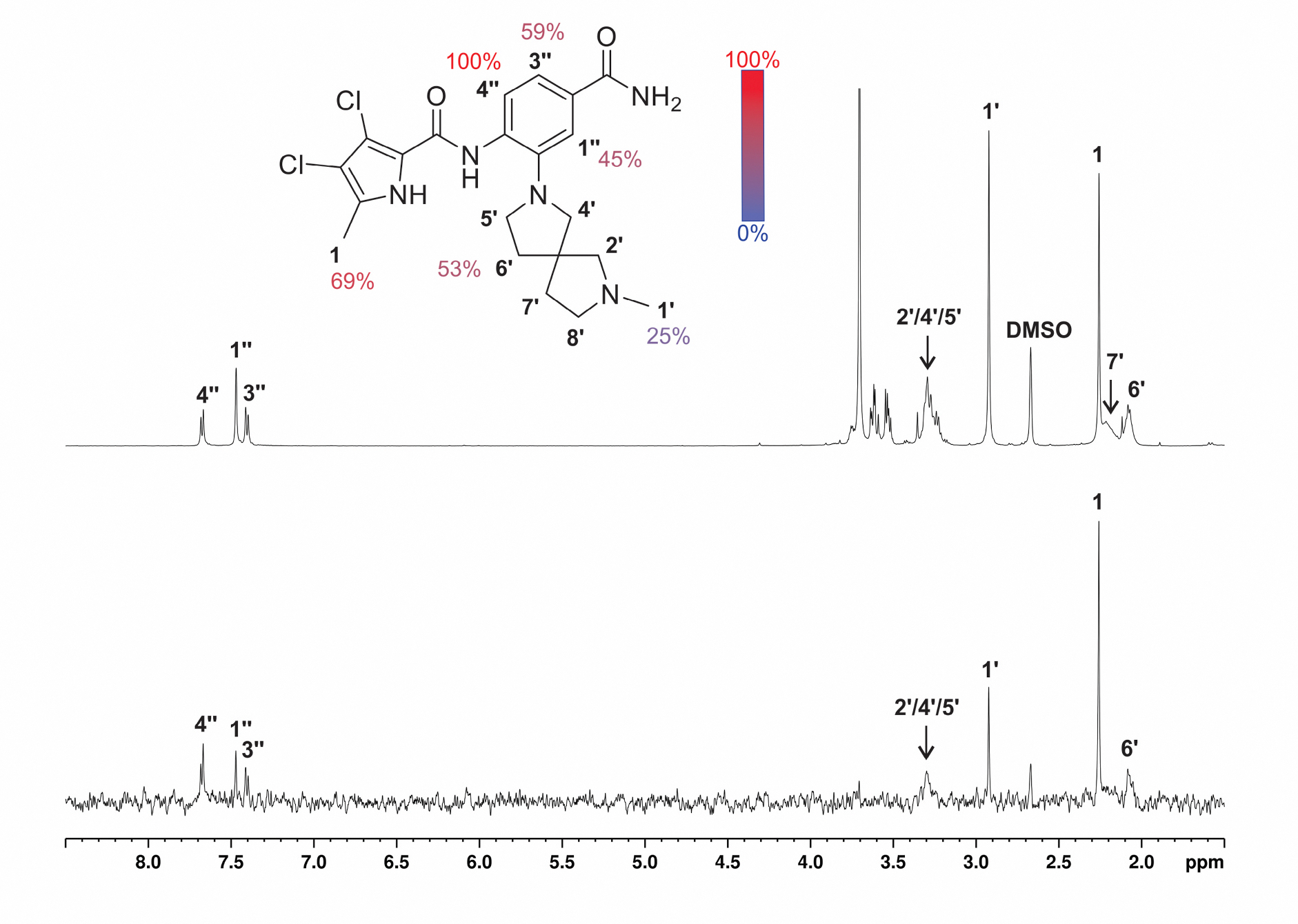
1D ^1^H STD NMR spectra for the compound **24e** recorded at an Hsp90β:ligand ratio of 1:200 and 600 MHz. The molecular structure illustrates the proton nomenclature and the color-coded relative degrees of saturation of the individual not-overlapping protons. The STD amplification factors were normalized to the intensity of the signal with the largest STD effect. Reference STD spectra (top) with proton assignment and difference STD spectra (bottom) are shown. The unassigned proton signals between 3.5 and 3.8 ppm belong to the protein buffer with glycerol. The proton signals were calibrated to the DSS signal at 0.0 ppm. The spectra are not to scale.

To further validate the *in silico* findings, we performed STD NMR (Fig. 9) and trNOESY (Fig. S32) studies with Hsp90β. The binding mode of **24e** in the ATP-binding site of Hsp90β proposed by molecular docking and MD simulations corresponds to the 1D ^1^H STD NMR amplification factors. These indicate that the binding is tightest in the region of the core aromatic ring proton 4″ and the methyl group of the 3,4-dichloro-5-methylpyrrole moiety. High saturation (69 %) of the methyl group attached to the pyrrole is likely to be particularly important for selectivity as this part of the molecule could interact with the water-mediated hydrogen bond network formed by Ala47/Ser52 and Leu86/Ile91 in Hsp90β/Hsp90α, respectively. As previously shown, the displacement of water molecules in this network and the greater steric availability in Hsp90β drive the selectivity for Hsp90β vs. Hsp90α [8,9,23]. This could make the 5-methylpyrrole moiety critical for the ability to discriminate between the two cytoplasmic isoforms. Based on the on-target activities, we can also see that the position of the basic center is important for binding, which was also corroborated by the STD NMR studies. While the saturation of the part of the molecule carrying the basic center is not as intensive, it still indicates interaction with the protein. This may allow an appropriate position of the basic center of **24e** in Hsp90β, which could ensure strong ionic interactions with Asp49. These interactions are consistent with the established SAR of the compound series and the interaction analysis of the MD simulation trajectory of **24e** in complex with Hsp90β.

## 3. Conclusion

Utilizing our previous knowledge on the GHKL protein family we virtually screened an in-house library of 953 TopoIIα and DNA gyrase B inhibitors. Eleven hits were evaluated, four of which inhibited Hsp90 with a preference for the Hsp90β isoform. Next, we prepared a focused library of 29 new analogs and established SAR. Among these compounds, compound **24e** emerged as a selective inhibitor of Hsp90β with a 27-fold higher affinity for Hsp90β than Hsp90α. **24e** and its analogs inhibited the growth of MCF-7 breast cancer cells in the high nanomolar to low micromolar range and displayed anticancer activity against various cancer types in an NCI-60 screen. Furthermore, **24e** showed promising physicochemical properties and sufficiently good safety profile to be further evaluated. **24e** was shown to induce apoptosis and cause G0/G1 cell cycle arrest in MCF-7 cell line. It also statistically significantly decreased the levels of Hsp90 client proteins ERα, CDK4 and Akt without a significant induction of the HSR. The binding mode study of **24e** using molecular modelling, protein mutations and STD NMR supports the SAAA hydrogen bond network as the key binding site motif that enables the design of Hsp90β-selective compounds.

## 4. Methods

### 4.1. Synthesis and analytical data

In the preparation of intermediates and final products, reagents and solvents purchased from Enamine Ltd. (Kyiv, Ukraine), Sigma-Aldrich (St. Louis, MO, USA), Fluorochem Ltd. (Derbyshire, UK), Activate Scientific GmbH (Prien am Chiemsee, Germany) and Apollo Scientific Ltd. (Stockport, UK) were used without further purification. Silica gel aluminum sheets (0.20 mm; 60 F^25^4) that were used for analytical thinlayer chromatography were procured from Merck (Darmstadt, Germany), while silica gel 60 (particle size, 230–400 mesh) was used for column chromatography. Isolera One System (Biotage, Uppsala, Sweden) was used for purification with a reversed phase column and compound detection at 254 nm was applied. NMR spectra (^1^H, ^13^C and ^19^F) were recorded on a 400 MHz NMR spectrometer (Bruker Advance 3, Bruker, Billerica, MA, USA). The usual designation of the splitting patterns was performed as follows: s, singlet; d, doublet; dd, double doublet; td, triple doublet; t, triplet; dt, double triplet; ddd, double of doublet of doublet; q, quartet; p, pentet; and m, multiplet. For the assessment of purity of the prepared compounds high performance liquid chromatography (HPLC) (1260 Infinity II LC system; Agilent Technologies, Santa Clara, CA, USA) coupled to a mass spectrometer (MS) (Expression CMS^L^; Advion Inc., Ithaca, NY, USA) was used. The method used for LC-MS incorporated a C18 column (Waters xBridge BEH; 4.6 mm × 150 mm, 3.5 μm) at 40 °C, where the flow rate was of the mobile phase was 1.5 mL/min. The volume of injection was 10 μL. Finally, the products were detected with a UV–Vis at 254 nm. Gradient elution was used, and the solvent A comprised 1 % CH_3_CN and 0.1 % HCOOH in doubledistilled H_2_O while solvent B was pure acetonitrile. The gradient of solvents was applied as follows: 0 → 1 min, 25 % B; 1 → 6 min, 25 %→98 % B; 6 → 6.5 min, 98 % B; 6.5 → 7 min, 98 %→25 % B; 7 → 10 min, 25 % B. The high-resolution mass spectra (HRMS) were obtained using Exactive Plus Orbitrap mass spectrometer (Thermo Scientific Inc., Waltham, MA, USA).

#### Methyl 3-hydroxy-4-nitrobenzoate (12).

3-Hydroxy-4-nitrobenzoic acid (10.0 g, 54.6 mmol) was suspended in MeOH (300 mL) and SOCl_2_ (11.9 mL, 164 mmol) was added dropwise at 0 °C. The mixture was stirred at room temperature overnight. The solvent was evaporated under reduced pressure and the residue was washed with petroleum ether (50 mL). Yield: 10.1 g (94 %); yellow amorphous powder; R_f_ (EtOAc/hexane = 1:4) = 0.38; ^1^H NMR (400 MHz, CDCl_3_) *δ* 10.51 (s, 1H, Ar-O*H*), 8.18 (d, *J* = 8.8 Hz, 1H, Ar–*H*), 7.84 (d, *J* = 1.8 Hz, 1H, Ar–*H*), 7.62 (dd, *J*_*1*_ = 8.8 Hz, *J*_*2*_ = 1.8 Hz, 1H, Ar–*H*), 3.97 (s, 3H, COOC*H*_*3*_); ^13^C NMR (101 MHz, DMSO-*d*_6_) *δ* 165.2151.8, 140.6, 134.9, 126.0, 119.9, 119.8, 53.2; LC-MS (ESI−) C_8_H_7_NO_5_ *m*/*z*: 195.7 [M − H]^−^.

#### General procedure A.

Respective substituted phenol (1 equiv.), respective aliphatic alcohol (1 equiv.), and PPh_3_ (1.3 equiv.) were dissolved in dry tetrahydrofuran under an argon atmosphere. DIAD (1.3 equiv.) was added dropwise at 0 °C. The mixture was stirred at room temperature overnight. The solvent was evaporated under reduced pressure and the products were purified using column chromatography.

#### 1-Boc-4-(5-(methoxycarbonyl)-2-nitrophenoxy)piperidine (13).

The synthesis was performed according to the general procedure A. Compound **12** (2.49 g, 12.6 mmol) and 1-Boc-4-hydroxypiperidine were used for the synthesis. Crude product was purified by column chromatography using EtOAc:hexane = 1:2 as eluent. Yield: 3.04 (63 %), yellow oil, R_f_ (EtOAc/hexane = 1:2) = 0.22; ^1^H NMR (400 MHz, CDCl_3_) *δ* 7.81 (d, *J* = 8.4 Hz, 1H, Ar–*H*), 7.74 (d, *J* = 1.6 Hz, 1H, Ar–*H*), 7.69 (dd, *J*_*1*_ = 8.4 Hz, *J*_*2*_ = 1.6 Hz, 1H, Ar–*H*), 4.84–4.74 (m, 1H, C*H*), 3.97 (s, 3H, COO–C*H*_*3*_), 3.62–3.50 (m, 4H, 2 × C*H*_*2*_), 1.98–1.82 (m, 4H, 2 × C*H*_*2*_), 1.47 (s, 9H, C(C*H*_*3*_)_*3*_); ^13^C NMR (101 MHz, DMSO-*d*_6_) *δ* 165.2, 156.6, 154.3, 149.6, 143.9, 134.7, 125.7, 122.1, 117.2, 79.3, 74.2, 68.3, 53.3, 30.2, 28.5, 22.4 – signals of the remaining dihydro-DIAD are also assigned; LC-MS (ESI−) C_18_H_24_N_2_O_7_ *m*/*z*: 366.0 [M − CH_3_]^−^.

#### General procedure B.

Respective methyl ester was dissolved or suspended in methanol. The reaction mixture was bubbled with NH_3(g)_, heated to 100 °C in a pressure tube and stirred overnight. The solvent was evaporated under reduced pressure and the product was additionally purified when needed.

#### 1-Boc-4-(5-carbamoyl-2-nitrophenoxy)piperidine (14).

The synthesis was performed according to the general procedure B with compound **13** (4.50 g, 11.83 mmol) as a starting material. The crude product was purified by column chromatography using EtOAc:hexane = 1:2 as eluent. Yield: 2.82 g (65 %); R_f_ (EtOAc/hexane = 2:1) = 0.10; ^1^H NMR (400 MHz, CDCl_3_) *δ* 7.84 (d, *J* = 8.3 Hz, 1H, Ar–*H*), 7.66 (d, *J* = 1.6 Hz, 1H, Ar–*H*), 7.32 (dd, *J*_*1*_ = 8.3 Hz, *J*_*2*_ = 1.6 Hz, 1H, Ar–*H*), 6.23 (s, 1H, CON*H*_*2*_*-H*_*A*_), 5.92 (s, 1H, CON*H*_*2*_*-H*_*A*_), 4.84–4.76 (m, 1H, C*H*), 3.62–3.47 (m, 4H, 2 × C*H*_*2*_), 1.98–1.80 (m, 4H, 2 × C*H*_*2*_), 1.47 (s, 9H, C(C*H*_*3*_)_*3*_); LC-MS (ESI−) C_17_H_23_N_3_O_6_ *m*/*z*: 364.1 [M − H]^−^.

#### General procedure C.

Respective nitrobenzene was dissolved in MeOH and palladium on charcoal (Pd/C, 10 % wt) was added. The reaction mixture was then bubbled with hydrogen and left to stir overnight in hydrogen atmosphere. The reaction mixture was filtered through Celite^®^ and the solvent evaporated under reduced pressure.

#### 1-Boc-4-(2-amino-5-carbamoylphenoxy)piperidine (15).

The synthesis was performed according to the general procedure C with compound **14** (2.77 g, 7.58 mmol) as a starting material. Yield: 2.49 g (98 %), brown solid, R_f_ (EtOAc/hexane = 2:1) = 0.30; ^1^H NMR (400 MHz, CDCl_3_) *δ* 7.43 (d, *J* = 1.8 Hz, 1H, Ar–*H*), 7.17 (dd, *J*_*1*_ = 8.1 Hz, *J*_*2*_ = 1.8 Hz, 1H, Ar–*H*), 6.68 (d, *J* = 8.1 Hz, 1H, Ar–*H*), 5.83 (s, 2H, CON*H*_*2*_), 4.62–4.51 (m, 1H, C*H*), 4.18 (s, 2H, Ar-N*H*_*2*_), 3.82–3.65 (m, 2H, C*H*_*2*_), 3.35–3.22 (m, 2H, C*H*_*2*_), 2.03–1.90 (m, 2H, C*H*_*2*_), 1.81–1.69 (m, 2H, C*H*_*2*_), 1.47 (s, 9H, C(C*H*_*3*_)_*3*_); MS (ESI+) C_17_H_25_N_3_O_4_ *m/z* = 336.0 ([M+H]^+^.

#### General procedure D.

Corresponding aryl carboxylic acid (1.2 equiv.) was dissolved in SOCl_2_ (2 mL/mmol), the reaction mixture was heated to 70 °C for 1 h and the solvent was evaporated under reduced pressure to yield an acid chloride. Corresponding aromatic amine (1 equiv.) was dissolved in anhydrous DCM (10 mL/mmol) and pyridine (1 mL/mmol) under argon atmosphere. The prepared mixture was added to the previously prepared acid chloride and the reaction mixture was stirred under an argon atmosphere at room temperature overnight. The solvent was evaporated under reduced pressure.

#### 1-Boc-4-(5-carbamoyl-2-(3,4-dichloro-5-methyl-1H-pyrrole-2-carboxamido)phenoxy)piperidine (16a).

The synthesis of **16a** was performed according to the previously published procedure [39].

#### 1-Boc-4-(5-carbamoyl-2-(1*H*-indole-2-carboxamido)phenoxy)piperidine(16b).

The synthesis was performed according to the general procedure D. Indole-2-carboxylic acid (0.529 g, 3.28 mmol) and compound **15** (1.00 g, 2.98 mmol) were used as starting materials. To the solid residue, EtOAc (30 mL) and water (20 mL) were added. The undissolved solid was filtered off, washed with EtOAc and dried to obtain a part of the pure product **16b** (0.154 g). The mother liquor was transferred to the separating funnel and the phases were separated. The organic phase was washed with saturated NaHCO_3_ solution (3 × 20 mL), 0.5 M HCl (3 × 20 mL) and brine (20 mL), dried over Na_2_SO_4_ and filtered. The solvent was evaporated under reduced pressure. The obtained solid was washed with diethyl ether and the solid residue was purified by column chromatography using DCM/MeOH = 20:1 to 10:1 as the mobile phase. Yield: 0.844 g (70 %); light brown amorphous solid; R_f_ (DCM/MeOH = 10:1) = 0.39; ^1^H NMR (DMSO-*d*_6_, 400 MHz): *δ* 11.85 (s, 1H, indole-N*H*), 9.38 (s, 1H, indole-CON*H*-Ar), 8.02 (d, *J* = 8.3 Hz, 1H, Ar–*H*), 7.99 (s, 1H, CON*H*_*2*_*-H*_*A*_), 7.68 (dd, *J*_*1*_ = 8.0 Hz, *J*_*2*_ = 0.7 Hz, 1H, Ar–*H*), 7.62 (d, *J* = 1.7 Hz, 1H, Ar–*H*), 7.55 (dd, *J*_*1*_ = 8.3 Hz, *J*_*2*_ = 1.7 Hz, 1H, Ar–*H*), 7.47 (dd, *J*_*1*_ = 8.3 Hz, *J*_*2*_ = 1.0 Hz, 1H, Ar–*H*), 7.39 (s, 1H, CON*H*_*2*_*-H*_*B*_), 7.31 (d, *J* = 1.5 Hz, 1H, Ar–*H*), 7.24 (ddd, *J*_*1*_ = 8.3 Hz, *J*_*2*_ = 7.1 Hz, *J*_*3*_ = 1.0 Hz, 1H, Ar–*H*) 7.08 (ddd, *J*_*1*_ = 8.0 Hz, *J*_*2*_ = 7.1 Hz, *J*_*3*_ = 0.7 Hz, 1H, Ar–*H*), 4.76–4.61 (m, 1H, C*H*), 3.74–3.59 (m, 2H, C*H*_*2*_), 3.29–3.16 (m, 2H, C*H*_*2*_), 1.97–1.90 (m, 2H, C*H*_*2*_), 1.76–1.64 (m, 2H, C*H*_*2*_), 1.38 (s, 9H, C(C*H*_*3*_)_*3*_); HRMS for C_26_H_29_O_5_N_4_ ([M − H]^−^): calculated 477.21338, found 477.21338.

#### 1-Boc-4-(5-carbamoyl-2-(1*H*-5-fluoroindole-2-carboxamido)phenoxy)piperidine (16c).

The synthesis was performed according to the general procedure D. 5-Fluoroindole-2-carboxylic acid (0.588 g, 3.28 mmol) and compound **15** (1.00 g, 2.98 mmol) were used as starting materials. DCM was added to the solid residue and the undissolved solid was filtered off, washed with DCM and dried, to obtain **16c** as a white solid. Yield: 0.712 g (44 %); white amorphous solid; R_f_ (DCM/MeOH = 10:1) = 0.30; ^1^H NMR (DMSO-*d*_6_, 400 MHz): *δ* 11.95 (d, *J* = 2.2 Hz, 1H, indole-N*H*), 9.45 (s, 1H, indole-CON*H*-Ar), 7.99 (s, 1H, CON*H*_*2*_*-H*_*A*_), 7.98 (d, *J* = 8.3 Hz, 1H, Ar–*H*), 7.62 (d, *J* = 1.8 Hz, 1H, Ar–*H*), 7.55 (dd, *J*_*1*_ = 8.3 Hz, *J*_*2*_ = 1.8 Hz, 1H, Ar–*H*), 7.50–7.41 (m, 2H, 2 × Ar–*H*), 7.39 (s, 1H, CON*H*_*2*_*-H*_*B*_), 7.30 (d, *J* = 2.2 Hz, 1H, Ar–*H*), 7.10 (td, *J*_*1*_ = 9.2 Hz, *J*_*2*_ = 2.5 Hz, 1H, Ar–*H*), 4.75–4.69 (m, 1H, CH), 3.69–3.63 (m, 2H, C*H*_*2*_), 3.26–3.18 (m, 2H, C*H*_*2*_), 1.97–1.89 (m, 2H, C*H*_*2*_), 1.73–1.64 (m, 2H, C*H*_*2*_), 1.38 (s, 9H, C(C*H*_*3*_)_*3*_); HRMS for C_26_H_28_O_5_N_4_F ([M − H]^−^): calculated 495.20492, found 495.20400.

#### 1-Boc-4-(5-carbamoyl-2-(1*H*-5-chloroindole-2-carboxamido)phenoxy)piperidine (16d).

The synthesis was performed according to the general procedure D. 5-Chloroindole-2-carboxylic acid (0.641 g, 3.28 mmol) and compound **15** (1.00 g, 2.98 mmol) were used as starting materials. DCM was added to the solid residue and the undissolved solid was filtered off, washed with DCM and dried to obtain 1**6d** as a white solid. Yield: 1.20 g (78 %); off-white amorphous solid; R_f_ (DCM/MeOH = 10:1) = 0.40; ^1^H NMR (DMSO-*d*_6_, 400 MHz): *δ* 12.04 (d, *J* = 2.2 Hz, 1H, indole-N*H*), 9.49 (s, 1H, indole-CON*H*-Ar), 8.00 (s, 1H, CON*H*_*2*_*-H*_*A*_), 7.96 (d, *J* = 8.3 Hz, 1H, Ar–*H*), 7.77 (d, *J* = 2.1 Hz, 1H, Ar–*H*), 7.62 (d, *J* = 1.8 Hz, 1H, Ar–*H*), 7.55 (dd, *J*_*1*_ = 8.3 Hz, *J*_*2*_ = 1.8 Hz, 1H, Ar–*H*), 7.48 (d, *J* = 8.8 Hz, 1H, Ar–*H*), 7.39 (s, 1H, CON*H*_*2*_*-H*_*B*_), 7.31 (d, *J* = 2.5 Hz, 1H, Ar–*H*), 7.24 (dd, *J*_*1*_ = 8.8 Hz, *J*_*2*_ = 2.1 Hz, 1H, Ar–*H*), 4.74–4.68 (m, 1H, C*H*), 3.69–3.63 (m, 2H, C*H*_*2*_), 3.27–3.19 (m, 2H, C*H*_*2*_), 1.98–1.87 (m, 2H, C*H*_*2*_), 1.72–1.64 (m, 2H, C*H*_*2*_), 1.38 (s, 9H, C(C*H*_*3*_)_*3*_); HRMS for C_26_H_28_O_5_N_4_Cl ([M − H]^−^): calculated 511.17537, found 511.17416.

#### 1-Boc-4-(5-carbamoyl-2-(2,4-dihydroxy-5-isopropylbenzamido)phenoxy)piperidine (16e).

Acetic anhydride (2.0 mL, 21 mmol) was added to the 2,4-dihydroxy-5-isopropylbenzoic acid (600 mg, 3.06 mmol) and two drops of 98 % H_2_SO_4_ were added. The reaction mixture was heated at 65 °C for 1 h and then cooled to room temperature. Cooled mixture was poured onto a stirred mixture of ice and water (20 mL). The water phase was extracted with EtOAc (20 mL). The organic layer was washed with brine (20 mL), dried over Na_2_SO_4_ and filtered. The product was further purified by column chromatography using DCM/MeOH = 15:1 as eluent and the solvent was removed under pressure to yield crude 2,4-diacetoxy-5-isopropylbenzoic acid (R_f_ (DCM/MeOH = 15:1) = 0.13). 2,4-Diacetoxy-5-isopropylbenzoic acid (0.280 g, 0.999 mmol) was dissolved in anhydrous DCM (10 mL), then oxalyl chloride (0.257 mL, 3.00 mmol) and 3 drops of anhydrous DMF were added under an argon atmosphere. The reaction mixture was stirred for 1.5 h at room temperature, then the solvent was removed under reduced pressure. Compound **15** (0.335 g, 0.999 mmol) was dissolved in anhydrous DCM (10 mL) and pyridine (1 mL/mmol) under an argon atmosphere. The prepared mixture was added to the previously prepared acid chloride and the reaction mixture was stirred under an argon atmosphere at room temperature overnight. The solvent was evaporated under reduced pressure and the product was partly purified using column chromatography (mobile phase DCM/MeOH = 20:1). The crude product was then dissolved in MeOH (10 mL) and 1 M NaOH (2.70 mL, 2.70 mmol) was added to the mixture. After 30 min the reaction mixture was concentrated under reduced pressure and acidified with 1 M HCl to pH = 3. The formed precipitate was filtered off and washed with diethyl ether to yield compound **16e**. 3 step yield: 14 %; white amorphous solid; ^1^H NMR (400 MHz, DMSO-*d*_6_) *δ* 11.19 (s, 1H, Ar-O*H*), 10.84 (s, 1H, Ar-CON*H*-Ar or Ar-O*H*), 10.10 (s, 1H, Ar-CON*H*-Ar or Ar-O*H*), 8.55 (d, *J* = 8.5 Hz, 1H, Ar–*H*), 7.91 (s, 1H, CON*H*_*2*_*-H*_*A*_), 7.79 (s, 1H, Ar–*H*), 7.59 (d, *J* = 1.5 Hz, 1H, Ar–*H*), 7.51 (dd, *J*_*1*_ = 8.5 Hz, *J*_*2*_ = 1.6 Hz, 1H, Ar–*H*), 7.29 (s, 1H, CON*H*_*2*_*-H*_*B*_), 6.53 (s, 1H, Ar–*H*), 4.74–4.65 (m, 1H, O–C*H*), 3.84–3.74 (m, 2H, C*H*_*2*_), 3.20–3.07 (m, 3H, Ar-C*H*(CH_3_)_2_ and C*H*_*2*_), 2.04–1.99 (m, 2H, C*H*_*2*_), 1.66–1.55 (m, 2H, C*H*_*2*_), 1.42 (s, 9H, C(C*H*_*3*_)_*3*_), 1.15 (d, *J* = 6.9 Hz, 6H, CH(C*H*_*3*_)_*2*_) ppm; MS (ESI+) C_27_H_35_N_3_O_7_ *m/z* = 514.1 ([M+H]^+^).

#### 4-(5-Carbamoyl-2-(3,4-dichloro-5-methyl-1*H*-pyrrole-2-carboxamido)phenoxy)piperidin-1-ium chloride (17a).

The synthesis of **17a** was performed according to the previously published procedure [39].

#### 4-(5-Carbamoyl-2-(1*H*-indole-2-carboxamido)phenoxy)piperidin-1-ium chloride (17b).

To a solution of compound **16b** (0.844 g, 1.76 mmol) in a mixture of DMF (10 mL) and 1,4-dioxane (20 mL) 4 M HCl in 1,4-dioxane (30 mL) was added and the mixture was stirred overnight at room temperature. The solvent was evaporated under reduced pressure and the solid residue was washed with 1,4-dioxane to obtain 0.442 g of **17b.** The solvent of the mother liquor was evaporated under reduced pressure and the solid residue was washed with acetonitrile to obtain 0.297 g of **17b**. Both parts of the product were combined to obtain **17b**. Yield: 0.739 g (quantitative); light brown amorphous solid; ^1^H NMR (400 MHz, DMSO-*d*_6_): *δ* 11.91 (d, *J* = 2.2 Hz, 1H, indoleN*H*), 9.62 (s, 1H, indole-CON*H*-Ar), 8.99 (s, 1H, N*H*_*2*_^+^*-H*_*a*_), 8.86 (s, 1H, N*H*_*2*_^+^*-H*_*b*_), 8.04 (s, 1H, CON*H*_*2*_*-H*_*A*_), 7.94 (d, *J* = 8.3 Hz, 1H, Ar–*H*), 7.66–7.71 (m, 1H, Ar–*H*), 7.64 (d, *J* = 1.8 Hz, 1H, Ar–*H*), 7.57 (dd, *J*_*1*_ = 8.3 Hz, *J*_*2*_ = 1.8 Hz, 1H, Ar–*H*), 7.48 (dd, *J*_*1*_ = 8.3 Hz, *J*_*2*_ = 1.0 Hz, 1H, Ar–*H*), 7.41 (s, 1H, CON*H*_*2*_*-H*_*B*_), 7.34 (dd, *J*_*1*_ = 2.2 Hz, *J*_*2*_ = 1.0 Hz, 1H, Ar–*H*), 7.24 (ddd, *J*_*1*_ = 8.2 Hz, *J*_*2*_ = 6.9 Hz, *J*_*2*_ = 1.2 Hz, 1H, Ar–*H*), 7.09 (ddd, *J*_*1*_ = 8.0 Hz, *J*_*2*_ = 6.9 Hz, *J*_*3*_ = 1.0 Hz, 1H, Ar–*H*), 4.79–4.88 (m, 1H, C*H*), 3.35–3.25 (m, 2H, C*H*_*2*_), 3.15–3.05 (m, 2H, C*H*_*2*_), 2.20–2.09 (m, 2H, C*H*_*2*_), 2.05–1.93 (m, 2H, C*H*_*2*_); ^13^C NMR (101 MHz, DMSO-*d*_6_) *δ* 167.6, 160.0, 148.5, 137.4, 131.7, 131.6, 130.8, 127.5, 124.4, 124.0, 122.3, 121.0, 120.5, 113.2, 112.9, 104.5, 70.4, 34.5, 27.4; HRMS for C_21_H_23_O_3_N_4_ ([M+H]^+^): calculated 379.17647, found 379.17481.

#### 4-(5-Carbamoyl-2-(1*H*-5-fluoroindole-2-carboxamido)phenoxy)piperidin-1-ium chloride (17c).

To a solution of compound **16c** (0.712 g, 1.43 mmol) in a mixture of DMF (15 mL) and 1,4-dioxane (20 mL) 4 M HCl in 1,4-dioxane (40 mL) was added and the mixture was stirred overnight at room temperature. The solvent was evaporated under reduced pressure and the solid residue was washed with acetonitrile. The solid residue was additionally washed with diethyl ether and dried. Yield: 0.587 g (95 %); ^1^H NMR (400 MHz, DMSO-*d*_6_): *δ* 12.03 (d, *J* = 2.2 Hz, 1H, indole-N*H*), 9.69 (s, 1H, indole-CON*H*-Ar), 8.98 (s, 1H, N*H*_*2*_^+^*-H*_*a*_), 8.90 (s, 1H, N*H*_*2*_^+^*-H*_*b*_), 8.90 (s, 1H, NH_2_^+^-*H*_*a*_), 8.04 (s, 1H, CON*H*_*2*_*-H*_*A*_), 7.90 (d, *J* = 8.3 Hz, 1H, Ar–*H*), 7.64 (d, *J* = 1.8 Hz, 1H, Ar–*H*), 7.57 (dd, *J*_*1*_ = 8.2 Hz, *J*_*2*_ = 1.8 Hz, 1H, Ar–*H*), 7.50–7.44 (m, 2H, 2 × Ar–*H*), 7.41 (s, 1H, CON*H*_*2*_*-H*_*B*_), 7.34 (d, *J* = 2.1 Hz, 1H, Ar–*H*), 7.11 (td, *J*_*1*_ = 9.2 Hz, *J*_*2*_ = 2.6 Hz, 1H, Ar–*H*), 4.86–4.79 (m, 1H, C*H*), 3.34–3.25 (m, 2H, C*H*_*2*_), 3.14–3.05 (m, 2H, C*H*_*2*_), 2.17–2.09 (m, 2H, C*H*_*2*_), 2.02–1.93 (m, 2H, C*H*_*2*_); ^13^C NMR (101 MHz, DMSO-*d*_6_): *δ* 167.6, 159.8, 157.7 (d, *J* = 233.8 Hz), 148.7, 134.1, 133.3, 131.9, 130.6, 127.5 (d, *J* = 10.3 Hz), 124.3, 120.9, 114.2 (d, *J* = 9.6 Hz), 113.5–112.8 (overlapping signals for 2 × ^13^C, one doublet and one singlet), 106.4 (d, *J* = 22.9 Hz), 104.5 (d, *J* = 5.5 Hz), 70.4, 34.5, 27.4 ppm; HRMS for C_21_H_22_O_3_N_4_F ([M+H]^+^): calculated 397.16705, found 397.16551.

#### 4-(5-Carbamoyl-2-(1*H*-5-chloroindole-2-carboxamido)phenoxy)piperidin-1-ium chloride (17d).

To a solution of compound **16d** (1.20 g, 2.34 mmol) in a mixture of DMF (20 mL) and 1,4-dioxane (20 mL) 4 M HCl in 1,4-dioxane (50 mL) was added and the mixture was stirred overnight at room temperature. The solvent was evaporated under reduced pressure and the solid residue was washed with 1,4-dioxane to obtain a part of pure **17d** (0.380 g). Mother liquor was concentrated under reduced pressure and to the solid residue acetonitrile was added, and the undissolved solid was filtered off and dried to obtain another part of pure **17d** (0.668 g). Yield: 1.04 g (99 %); white amorphous solid; ^1^H NMR (400 MHz, DMSO-*d*_6_): *δ* 12.12 (d, *J* = 2.2 Hz, 1H, indole-N*H*), 9.73 (s, 1H, indole-CON*H*-Ar), 8.92 (s, 1H, N*H*_*2*_^+^*-H*_*a*_), 8.87 (s, 1H, N*H*_*2*_^+^-*H*_*b*_), 8.04 (s, 1H, CON*H*_*2*_*-H*_*A*_), 7.88 (d, *J* = 8.3 Hz, 1H, Ar–*H*), 7.77 (d, *J* = 2.1 Hz, 1H, Ar–*H*), 7.64 (d, *J* = 1.8 Hz, 1H, Ar–*H*), 7.57 (dd, *J*_*1*_ = 8.3 Hz, *J*_*2*_ = 1.8 Hz, 1H, Ar–*H*), 7.49 (d, *J* = 8.7 Hz, 1H, Ar–*H*), 7.42 (s, 1H, CON*H*_*2*_*-H*_*B*_), 7.34 (d, *J* = 2.1 Hz, 1H, Ar–*H*), 7.25 (dd, *J*_*1*_ = 8.8 Hz, *J*_*2*_ = 2.1 Hz, 1H, Ar–*H*), 4.85–4.79 (m, H, C*H*), 3.34–3.25 (m, 2H, C*H*_*2*_), 3.15–3.05 (m, 2H, C*H*_*2*_), 2.17–2.08 (m, 2H, C*H*_*2*_), 2.04–1.91 (m, 2H, C*H*_*2*_); ^13^C NMR (101 MHz, DMSO-*d*_6_): *δ* 167.6, 159.7, 148.8, 135.8, 133.1, 132.0, 130.6, 128.5, 125.0, 124.49, 124.40, 121.3, 120.9, 114.6, 113.3, 104.1, 70.3, 27.4; Signal for one aliphatic carbon not seen; HRMS for C_21_H_22_O_3_N_4_Cl ([M+H]^+^): calculated 413.13749, found 413.13579.

#### 4-(5-Carbamoyl-2-(2,4-dihydroxy-5-isopropylbenzamido)phenoxy)piperidin-1-ium chloride (17e).

To a solution of compound **16e** (50 mg, 0.097 mmol) in 1,4-dioxane (10 mL) 4 M HCl in 1,4-dioxane (0.608 mL, 2.43 mmol) was added and the mixture was stirred overnight at room temperature. The solvent was evaporated under reduced pressure and diethyl ether was added to the residue to form a precipitate which was filtered off. The precipitate was then purified using Isolera One (gradient of solvents: 0 → 3 min, 100 % water; 3 → 20 min, 0 %→100 % MeCN and 100 %→0 % water; 20 → 25 min, 100 % MeOH; 6.5 → 7 min, 98 %→25 % B; 7 → 10 min, 25 % B; and flow rate 12 mL/min) and the solvent was evaporated. Yield: 15 mg (34 %); off-white amorphous solid; ^1^H NMR (400 MHz, DMSO-*d*_6_): *δ* 10.94 (s, 1H, Ar-CON*H*-Ar), 10.33–9.74 (m, 2H, N*H*_*2*_^+^), 8.55 (d, *J* = 8.5 Hz, 1H, Ar–*H*), 7.91 (s, 1H, CON*H*_*2*_*-H*_*A*_), 7.78 (s, 1H, Ar–*H*), 7.58 (d, *J* = 1.7 Hz, 1H, Ar–*H*), 7.53 (dd, *J*_*1*_ = 8.5 Hz, *J*_*2*_ = 1.7 Hz, 1H, Ar–*H*), 7.30 (s, 1H, CON*H*_*2*_*-H*_*B*_), 6.56 (s, 1H, Ar–*H*), 4.78–4.71 (m, 1H, O–C*H*), 3.25–3.17 (m, 2H, C*H*_*2*_), 3.14–3.06 (m, 1H, Ar-C*H*(CH_3_)_2_), 3.02–2.92 (m, 2H, C*H*_*2*_), 2.17–2.04 (m, 2H, C*H*_*2*_), 1.90–1.79 (m, 2H, C*H*_*2*_), 1.15 (d, *J* = 6.9 Hz, 6H, CH(C*H*_*3*_)_*2*_); ^13^C NMR (101 MHz, MeOD) *δ* 170.3, 166.0, 159.9, 157.3, 145.6, 132.9, 128.3, 128.2, 127.3, 120.9, 120.0, 112.2, 109.9, 102.6, 70.3, 41.0, 27.5, 26.3, 21.7; HRMS for C_22_H_27_N_3_O_5_ ([M+H]^+^): calculated 414.2024, found 414.2015; HPLC: t_r_ = 3.23 min (97.1 % at 254 nm).

#### General procedure E.

Corresponding secondary or primary amine salt was dissolved in either methanol or a mixture of methanol and another solvent (DCM or THF). Then triethylamine (2 equiv.) and 37 % water solution of formaldehyde (5 equiv.) were added to the reaction mixture. After 1h sodium cyanoborohydride (1.5 equiv.) was added and the mixture was stirred at room temperature overnight. The solvent was evaporated under reduced pressure.

#### *N*-(4-Carbamoyl-2-((1-methylpiperidin-4-yl)oxy)phenyl)-3,4-dichloro-5-methyl-1*H*-pyrrole-2-carboxamide (18a).

Synthesis was performed according to the general procedure E with compound **17a** (0.102 g, 0.409 mmol) as a starting material. Crude product was triturated with methanol. Yield 8.0 % (14 mg); grey amorphous solid. ^1^H NMR (400 MHz, DMSO-*d*_6_): *δ* = 12.44 (s, 1H, pyrrole-N*H*), 9.16 (s, 1H, pyrrole-CON*H*-Ar), 8.43 (d, 1H, *J* = 8.4 Hz, Ar–*H*), 7.94 (s, 1H, CON*H*_*2*_*-H*_*A*_), 7.60 (d, 1H, *J* = 1.9 Hz, Ar–*H*), 7.53 (dd, 1H, *J*_*1*_ = 8.5 Hz, *J*_*2*_ = 1.8 Hz, Ar–*H*), 7.33 (s, 1H, CON*H*_*2*_*-H*_*B*_), 4.51–4.64 (m, 1H, C*H*), 2.62–2.75 (m, 2H, piperidine-H_2_), 2.23 (s, 3H, C*H*_*3*_ C*H*_*3*_), 2.12–2.21 (m, 5H, C*H*_*2*_ and C*H*_*3*_), 1.96–2.08 (m, 2H, C*H*_*2*_), 1.62–1.77 (m, 2H, C*H*_*2*_) ppm; ^13^C NMR (100 MHz, DMSO-*d*_6_): *δ* 179.1, 174.5, 167.6, 145.5, 140.2, 131.4, 130.3, 121.1, 118.7, 112.3, 110.1, 109.0, 74.5, 53.1, 46.2, 31.3, 11.3; MS (ESI) *m/z* = 425.5 ([M+H]^+^); HPLC: t_r_ = 4.57 min (97.7 % at 254 nm).

#### *N*-(4-Carbamoyl-2-((1-methylpiperidin-4-yl)oxy)phenyl)-1*H*-indole-2-carboxamide (18b).

The synthesis was performed according to the general procedure E with compound **17b** (0.200 g, 0.483 mmol) as a starting material. The product was purified by column chromatography using DCM/MeOH/NH_4_OH = 10:1:0.1 as the mobile phase. Yield: 0.081 mg (43 %); white amorphous solid; R_f_ (DCM/MeOH/NH_4_OH = 10:1:0.1) = 0.06; ^1^H NMR (400 MHz, DMSO-*d*_6_): *δ* 11.88 (s, 1H, indoleN*H*), 9.31 (s, 1H, indole-CON*H*-Ar), 8.07 (d, *J* = 8.3 Hz, 1H, Ar–*H*), 7.99 (s, 1H, CON*H*_*2*_*-H*_*A*_), 7.69 (d, *J* = 8.1 Hz, 1H, Ar–*H*), 7.62 (d, *J* = 1.9 Hz, 1H, Ar-6*H*), 7.55 (dd, *J*_*1*_ = 8.3 Hz, *J*_*2*_ = 1.9 Hz, 1H, Ar–*H*), 7.48 (dd, *J*_*1*_ = 8.3 Hz, *J*_*2*_ = 1.0 Hz, 1H, Ar–*H*), 7.37 (s, 1H, CON*H*_*2*_*-H*_*B*_), 7.33–7.27 (m, 1H, Ar–*H*), 7.24 (ddd, *J*_*1*_ = 8.3 Hz, *J*_*2*_ = 7.0 Hz, *J*_*3*_ = 1.2 Hz, 1H, Ar–*H*), 7.08 (ddd, *J*_*1*_ = 8.0 Hz, *J*_*2*_ = 7.0 Hz, *J*_*3*_ = 1.0 Hz, 1H, Ar–*H*), 4.59–4.53 (m, 1H, C*H*), 2.65–2.56 (m, 2H, C*H*_*2*_), 2.28–2.18 (m, 2H, C*H*_*2*_), 2.14 (s, 3H, C*H*_*3*_), 2.02–1.90 (m, 2H, C*H*_*2*_), 1.88–1.73z (m, 2H, C*H*_*2*_); ^13^C NMR (101 MHz, DMSO-*d*_6_): *δ* 167.7, 159.8, 147.8, 137.5, 131.6, 131.2, 131.0, 127.5, 124.5, 122.3, 120.9, 120.6, 113.7, 113.0, 104.0, 73.8, 52.6, 46.3, 30.8; HRMS for C_22_H_25_O_3_N_4_ ([M+H]^+^): calculated 393.19212, found 393.19131; HPLC: t_r_ = 2.25 min (96.6 % at 254 nm).

#### *N-*(4-Carbamoyl-2-((1-methylpiperidin-4-yl)oxy)phenyl)-1*H*-5-fluoroindole-2-carboxamide (18c).

The synthesis was performed according to the general procedure E with compound **17c** (0.200 g, 0.463 mmol) as a starting material. The product was purified by column chromatography using DCM/MeOH/NH_4_OH = 10:1:0.1 as eluent. Yield: 0.051 mg (27 %); white amorphous solid; R_f_ (DCM/MeOH/NH_4_OH = 10:1:0.1) = 0.04; ^1^H NMR (400 MHz, DMSO-*d*_6_): *δ* 11.98 (s, 1H, indole-N*H*), 9.38 (s, 1H, indole-CON*H*-Ar), 8.06–7.97 (m, 2H, Ar–*H* and CON*H*_*2*_*-H*_*A*_), 7.63 (d, *J* = 1.9 Hz, 1H, Ar–*H*), 7.54 (dd, *J*_*1*_ = 8.3 Hz, *J*_*2*_ = 1.9 Hz, 1H, Ar–*H*), 7.44–7.51 (m, 2H, 2 × Ar–*H*), 7.37 (s, 1H, CON*H*_*2*_*-H*_*B*_), 7.29 (dd, *J*_*1*_ = 2.3 Hz, *J*_*2*_ = 0.8 Hz, 1H, Ar–*H*), 7.11 (td, *J*_*1*_ = 9.3 Hz, *J*_*2*_ = 2.5 Hz, 1H, Ar–*H*), 4.51–4.58 (m, 1H, C*H*), 2.50 (m, 2H, C*H*_*2*_, overlapping with the signal for DMSO), 2.15–2.25 (m, 2H, C*H*_*2*_), 2.13 (s, 3H, C*H*_*3*_), 1.91–2.00 (m, 2H, C*H*_*2*_), 1.71–1.84 (m, 2H, C*H*_*2*_); ^13^C NMR (101 MHz, DMSO-*d*_6_): *δ* 167.68, 159.51, 157.74 (d, *J* = 233.2 Hz), 148.14, 134.19, 133.27, 131.29, 131.08, 127.6 (d, *J* = 10.7 Hz), 122.67, 120.85, 114.19 (d, *J* = 9.8 Hz), 113.75, 113.27 (d, *J* = 26.4 Hz), 106.38 (d, *J* = 22.9 Hz), 104.02 (d, *J* = 5.1 Hz), 73.91, 52.64, 46.30, 30.84; HRMS for C_22_H_24_O_3_N_4_F ([M+H]^+^): calculated 411.18270, found 411.18206; HPLC: t_r_ = 2.55 min (95.3 % at 254 nm).

#### *N*-(4-Carbamoyl-2-((1-methylpiperidin-4-yl)oxy)phenyl)-1*H*-5-chloroindole-2-carboxamide (18d).

The synthesis was performed according to general procedure E and compound **17d** (0.100 g, 0.234 mmol) was used as starting material. The residue was washed with water and acetonitrile to yield a clean white solid without further purification. Yield: 0.067 mg (67 %); white amorphous solid; R_f_ (DCM/MeOH/NH_4_OH = 10/1/0.1) = 0.06 ^1^H NMR (DMSO-*d*_6_, 400 MHz): *δ* 12.07 (s, 1H, indole-N*H*), 9.42 (s, 1H, indole-CON*H*-Ar), 8.03–7.96 (m, 2H, Ar–*H* and CON*H*_*2*_*-H*_*A*_), 7.78 (d, *J* = 2.1 Hz, 1H, Ar–*H*), 7.63 (d, *J* = 1.9 Hz, 1H, Ar–*H*), 7.55 (dd, *J*_*1*_ = 8.3 Hz, *J*_*2*_ = 1.8 Hz, 1H, Ar–*H*), 7.48 (d, *J* = 8.7 Hz, 1H, Ar–*H*), 7.38 (s, 1H, CON*H*_*2*_*-H*_*B*_), 7.29 (d, *J* = 1.9 Hz, 1H, Ar–*H*), 7.24 (dd, *J*_*1*_ = 8.7 Hz, *J*_*2*_ = 2.1 Hz, 1H, Ar–*H*), 4.58–4.51 (m, 1H, C*H*), 2.63–2.54 (m, 2H, C*H*_*2*_), 2.24–2.16 (m, 2H, C*H*_*2*_), 2.13 (s, 3H, piperidine-C*H*_*3*_), 1.98–1.90 (m, 2H, C*H*_*2*_), 1.82–1.73 (m, 2H, C*H*_*2*_); ^13^C NMR (101 MHz, DMSO-*d*_6_): *δ* 167.7, 159.5, 148.3, 135.8, 133.1, 131.4, 131.0, 128.5, 125.0, 124.6, 122.9, 121.3, 120.8, 114.6, 113.8, 103.6, 73.9, 52.6, 46.4, 30.9; HRMS for C_22_H_24_O_3_N_4_Cl ([M+H]^+^): calculated 427.15314, found 427.15158; HPLC: t_r_ = 4.26 min (96.8 % at 254 nm).

#### General procedure F.

DMF (3 mL/mmol) was added to respective amine or phenol (1.2 equiv.) and K_2_CO_3_ (1.5 equiv.). Methyl 3-fluoro-4-nitrobenzoate (1 equiv.) was then added and the reaction mixture was heated to 60 °C–100 °C overnight. The solvent was evaporated under reduced pressure.

#### Methyl 3-(3-(((Boc)amino)methyl)phenoxy)-4-nitrobenzoate (19a).

The synthesis was performed according to the general procedure F (60 °C) using 3-(*N*-Boc-aminomethyl)phenol (0.410 g, 1.84 mmol) as starting material. The residue was taken up in ethyl acetate (30 mL), washed with 0.5 M NaOH (40 mL) and brine (40 mL), dried over Na_2_SO_4_ and filtered. The solvent was evaporated under reduced pressure and additionally purified by column chromatography using EtOAc:hexane = 1:2 as the mobile phase. Yield: 0.658 mg (89 %); yellow solid; R_f_ (EtOAc: hexane = 1:2) = 0.22. ^1^H NMR (400 MHz, CDCl_3_): *δ* 7.96 (d, *J* = 8.4 Hz, 1H, Ar–*H*), 7.85 (dd, *J*_*1*_ = 8.4 Hz, *J*_*2*_ = 1.7 Hz, 1H, Ar–*H*), 7.65 (d, *J* = 1.6 Hz, 1H, Ar–*H*), 7.36 (t, *J* = 7.9 Hz, 1H, Ar–*H*), 7.14 (d, *J* = 7.7 Hz, 1H, Ar–*H*), 7.00 (s, 1H,Ar–*H*), 6.94 (dd, *J*_*1*_ = 8.1 Hz, *J*_*2*_ = 2.0 Hz, 1H, Ar–*H*), 4.88 (s, 1H, N*H*COO), 4.33 (d, *J* = 5.8 Hz, 2H, Ar-C*H*_*2*_-NH), 3.91 (s, 3H, COO–C*H*_*3*_), 1.45 (s, 9H, C(C*H*_*3*_)_*3*_); MS (ESI+) C_27_H_35_N_3_O_7_ *m/z* = 402.9 [M+H]^+^.

#### Methyl 3-(4-(((Boc)amino)methyl)phenoxy)-4-nitrobenzoate (19b).

The synthesis was performed according to the general procedure F (60 °C) using 4-(*N*-Boc-aminomethyl)phenol (0.500 g, 2.24 mmol) as starting material. The residue was taken up in ethyl acetate (30 mL), washed with 0.5 M NaOH (40 mL) and brine (40 mL), dried over Na_2_SO_4_ and filtered. The solvent was evaporated under reduced pressure and additionally purified by column chromatography using EtOAc:hexane = 1:2 as the mobile phase. Yield: 0.766 mg (85 %); yellow solid; R_f_ (EtOAc: hexane = 1:2) = 0.21; ^1^H NMR (400 MHz, CDCl_3_): *δ* 7.95 (d, *J* = 8.4 Hz, 1H, Ar–*H*), 7.84 (dd, *J*_*1*_ = 8.4 Hz, *J*_*2*_ = 1.6 Hz, 1H, Ar–*H*), 7.64 (d, *J* = 1.6 Hz, 1H, Ar–*H*), 7.35–7.29 (m, 2H, 2 × Ar–*H*), 7.06–6.99 (m, 2H, 2 × Ar–*H*), 4.88 (s, 1H, N*H*COO), 4.33 (d, *J* = 5.7 Hz, 2H, Ar-C*H*_*2*_-NH), 3.90 (s, 3H, COO–C*H*_*3*_), 1.47 (s, 9H, C(C*H*_*3*_)_*3*_); MS (ESI+) C_27_H_35_N_3_O_7_ *m/z* = 402.7 [M+H]^+^.

#### 1-Boc-4-(5-(methoxycarbonyl)-2-nitrophenyl)piperazine (19c).

The synthesis was performed according to the general procedure F (60 °C) using *N*-Boc-piperazine (0.500 g, 2.68 mmol) as starting material. The residue was taken up in ethyl acetate (50 mL), washed with 1 % citric acid (3 × 50 mL) and brine (50 mL), dried over Na_2_SO_4_ and filtered. The solvent was removed under reduced pressure. Yield: 0.973 mg (99 %); orange oil; R_f_ (EtOAc:hexane = 1:2) = 0.35; ^1^H NMR (400 MHz, CDCl_3_): *δ* 7.82 (d, *J* = 1.6 Hz, 1H, Ar–*H*), 7.78 (d, *J* = 8.4 Hz, 1H, Ar–*H*), 7.72 (dd, *J*_*1*_ = 8.4 Hz, *J*_*2*_ = 1.6 Hz, 1H, Ar–*H*), 3.95 (s, 3H, COO–C*H*_*3*_) 3.61–3.54 (m, 4H, 2 × C*H*_*2*_), 3.11–3.00 (m, 4H, 2 × C*H*_*2*_), 1.48 (s, 9H, C(C*H*_*3*_)_*3*_); MS (ESI+) C_17_H_23_N_3_O_6_ *m/z* = 366.1 [M+H]^+^.

#### 1-Boc-4-((5-(methoxycarbonyl)-2-nitrophenyl)amino)piperidine (19d).

The synthesis was performed according to the general procedure F (60 °C) using 1-Boc-4-aminopiperidine (0.500 g, 2.50 mmol) as starting material. The residue was taken up in ethyl acetate (50 mL), washed with 1 % citric acid (3 × 50 mL) and brine (50 mL), dried over Na_2_SO_4_ and filtered. The solvent was removed under reduced pressure. Yield: 0.973 mg (99 %); light red oil; R_f_ (EtOAc:hexane = 1:2) = 0.34; ^1^H NMR (400 MHz, CDCl_3_): *δ* 8.23 (d, *J* = 8.9 Hz, 1H, Ar–*H*), 8.05 (d, *J* = 7.4 Hz, 1H, Ar-N*H*), 7.57 (d, *J* = 1.6 Hz, 1H, Ar–*H*), 7.24 (dd, *J*_*1*_ = 8.9 Hz, *J*_*2*_ = 1.6 Hz, 1H, Ar–*H*), 4.09–3.99 (m, 2H, C*H*_*2*_), 3.95 (s, 3H, COO–C*H*_*3*_), 3.83–3.70 (m, 1H, C*H*), 3.16–3.02 (m, 2H, C*H*_*2*_), 2.12–2.05 (m, 2H, C*H*_*2*_), 1.64–1.51 (m, 2H, C*H*_*2*_), 1.48 (s, 9H, C(C*H*_*3*_)_*3*_); MS (ESI+) C_17_H_23_N_3_O_6_ *m/z* = 365.1 [M-CH_3_+H]^+^.

#### 2-Boc-7-(5-(methoxycarbonyl)-2-nitrophenyl)-2,7-diazaspiro [4.4]nonane (19e).

The synthesis was performed according to the general procedure F (60 °C) using 2-Boc-2,7-diazaspiro[4.4]nonane (0.625 g, 2.76 mmol) as starting material. The residue was taken up in ethyl acetate (50 mL), washed with 1 % citric acid (3 × 50 mL) and brine (50 mL), dried over Na_2_SO_4_ and filtered. The solvent was removed under reduced pressure. Yield: 0.973 mg (99 %); orange oil; R_f_ (EtOAc:hexane = 1:1) = 0.37; ^1^H NMR (400 MHz, CDCl_3_): *δ* 7.75 (d, *J* = 8.5 Hz, 1H, Ar–*H*), 7.58 (s, 1H, Ar–*H*), 7.39–7.35 (m, 1H, Ar–*H*), 3.94 (s, 3H, COO–C*H*_*3*_), 3.53–3.36 (m, 4H, 2 × C*H*_*2*_), 3.34–3.08 (m, 4H, 2 × C*H*_*2*_), 2.09–1.83 (m, 4H 2 × C*H*_*2*_), 1.46 (s, 9H, C(C*H*_*3*_)_*3*_); ^13^C NMR (101 MHz, DMSO-*d*_6_) *δ* 165.8, 154.0, 142.1, 138.9, 133.9, 127.3, 117.8, 116.1, 78.8, 58.8, 54.4, 54.2, 53.1, 49.6, 48.6, 47.7, 45.3, 45.1, 34.4, 34.2, 33.5, 28.6, rotamers are present in the spectra; MS (ESI+) C_20_H_27_N_3_O_6_ *m/z* = 405.6 [M+H]^+^.

#### Methyl 3-(4-((Boc)amino)piperidin-1-yl)-4-nitrobenzoate (19f).

The synthesis was performed according to the general procedure F (60 °C) using 4-Boc-4-aminopiperidine (0.500 g, 2.50 mmol) as starting material. The residue was taken up in ethyl acetate (50 mL), washed with 1 % citric acid (3 × 50 mL) and brine (50 mL), dried over Na_2_SO_4_ and filtered. The solvent was removed under reduced pressure. Yield: 0.823 mg (87 %); orange oil; R_f_ (EtOAc:hexane = 1:1) = 0.51; ^1^H NMR (400 MHz, CDCl_3_): *δ* 7.80 (d, *J* = 1.7 Hz, 1H, Ar–*H*), 7.76 (d, *J* = 8.4 Hz, 1H, Ar–*H*), 7.65 (dd, *J*_*1*_ = 8.4 Hz, *J*_*2*_ = 1.7 Hz, 1H, Ar–*H*), 4.50 (d, *J* = 6.7 Hz, 1H CON*H*CH), 3.95 (s, 3H, COO–C*H*_*3*_), 3.70–3.56 (m, 1H, C*H*), 3.32–3.22 (m, 2H, C*H*_*2*_), 2.99–2.90 (m, 2H, C*H*_*2*_), 2.08–2.00 (m, 2H, C*H*_*2*_), 1.65–1.54 (m, 2H, C*H*_*2*_), 1.46 (s, 9H, C(C*H*_*3*_)_*3*_); MS (ESI+) C_218_H_25_N_3_O_6_ *m/z* = 379.6 [M+H]^+^.

#### 1-Boc-4-((5-(methoxycarbonyl)-2-nitrophenoxy)methyl)piperidine (19g).

The synthesis was performed according to the general procedure A using 4-(hydroxymethyl)-*N*-Boc-piperidine (1.20 g, 5.57 mmol) as starting material. Crude product was purified by column chromatography using EtOAc:hexane = 1:4 to EtOAc:hexane = 1:2 as the mobile phase. Yield: 1.63 (54 %); off-white amorphous solid; R_f_ (EtOAc:hexane = 1:2) = 0.20; ^1^H NMR (400 MHz, DMSO-*d*_6_): *δ* = 8.00 (d, *J* = 8.4 Hz, 1H, Ar–*H*), 7.76 (d, *J* = 1.6 Hz, 1H, Ar–*H*), 7.66 (dd, *J*_*1*_ = 8.3, *J*_*2*_ = 1.6 Hz, 1H, Ar–*H*), 4.12 (d, *J* = 6.1 Hz, 2H, Ar-O-C*H*_*2*_), 4.02–3.92 (m, 2H, piperidin-C*H*_*2*_), 3.91 (s, 3H, COOC*H*_*3*_), 2.00–1.88 (m, 1H, C*H*), 1.76–1.64 (m, 2H, C*H*_*2*_), 1.40 (s, 9HC(C*H*_*3*_)_*3*_), signal for the missing C*H*_*2*_ is covered with DIAD residue; MS (ESI+) C_19_H_26_N_2_O_7_ *m*/*z:* 416.6 [M+Na]^+^.

#### General procedure G.

Corresponding carboxylic acid (1 equiv.) was dissolved in DMF. EDC (1.2 equiv.), HOBt (1.3 equiv.) and NMM (2 equiv.) were added on an ice bath. The reaction mixture was stirred for 20 min and then corresponding amine (1 equiv.) was added. The reaction mixture was stirred overnight at room temperature. The solvent was evaporated under reduced pressure and the residue was taken up in EtOAc (50 mL) which was then washed with 1 % citric acid (2 × 50 mL) and NaHCO_3_ (2 × 50 mL). The organic layer was washed with brine (50 mL), dried over Na_2_SO_4_, filtered and the solvent was evaporated under reduced pressure.

#### 1-Boc-4-(5-(methoxycarbonyl)-2-nitrobenzoyl)piperazine (19h).

The synthesis was performed according to the general procedure G using 5-(methoxycarbonyl)-2-nitrobenzoic acid (0.800 g, 3.55 mmol) and *N*-Boc-piperazine (0.794 g, 4.62 mmol) as starting materials. Following extraction, the solution was concentrated under reduced pressure and hexane was added to form a precipitate which was filtered off. Yield: 0.860 g (61 %); white solid; R_f_ (EtOAc:hexane = 2:1) = 0.41; ^1^H NMR (400 MHz, DMSO-*d*_6_): *δ* 8.33 (d, *J* = 8.6 Hz, 1H, Ar–*H*), 8.21 (dd, *J*_*1*_ = 8.6 Hz, *J*_*2*_ = 1.8 Hz, 1H, Ar–*H*), 8.04 (d, *J* = 1.8 Hz, 1H, Ar–*H*), 3.92 (s, 3H, COO–C*H*_*3*_), 3.69–3.57 (m, 2H, C*H*_*2*_), 3.52–3.40 (m, 2H, C*H*_*2*_), 3.30–3.17 (m, 4H, 2 × C*H*_*2*_), 1.41 (s, 9H, C(C*H*_*3*_)_*3*_); MS (ESI+) C_18_H_23_N_3_O_7_ *m/z* = 378.9 [M-CH_3_+H]^+^.

#### Methyl 3-((1-methyl-1*H*-indol-5-yl)oxy)-4-nitrobenzoate (19i).

The synthesis was performed according to the general procedure F (80 °C) using 1-methyl-1*H*-indol-5-ol (0.400 g, 2.72 mmol) as starting material. The product was purified by column chromatography using EtOAc:hexane = 1:3 as the mobile phase. Yield: 0.676 g (76 %); orange solid; R_f_ (EtOAc:hexane = 1:3) = 0.11; ^1^H NMR (400 MHz, DMSO-*d*_6_): *δ* 8.15 (d, *J* = 8.4 Hz, 1H, Ar–*H*), 7.75 (dd, *J*_*1*_ = 8.4 Hz, *J*_*2*_ = 1.7 Hz, 1H, Ar–*H*), 7.56 (d, *J* = 8.8 Hz, 1H, Ar–*H*), 7.45 (d, *J* = 3.0 Hz, 1H, Ar–*H*), 7.39 (d, *J* = 2.4 Hz, 1H, Ar–*H*), 7.33 (d, *J* = 1.7 Hz, 1H, Ar–*H*), 7.02 (dd, *J*_*1*_ = 8.8 Hz, *J*_*2*_ = 2.4 Hz, 1H, Ar–*H*), 6.46 (dd, *J*_*1*_ = 3.0 Hz, *J*_*2*_ = 0.7 Hz, 1H, Ar–*H*), 3.84 (s, 3H, COO–C*H*_*3*_), 3.77 (s, 3H, indole-C*H*_*3*_); MS (ESI+) C_17_H_14_N_2_O_5_ *m/z* = 326.9 [M-CH_3_+H]^+^.

#### Methyl 3-(4-methoxyphenoxy)-4-nitrobenzoate (19j).

The reaction was performed according to the general procedure F at 100 °C using 4-methoxyphenol (0.654 g, 5.27 mmol) as starting material. Crude product was purified by column chromatography using EtOAc:hexane = 1:4 as mobile phase. Yield: 1.35 g (89 %); yellow amorphous powder; R_f_ (EtOAc:hexane = 1:2) = 0.24; ^1^H NMR (400 MHz, DMSO-*d*_6_): *δ* 8.16 (d, *J* = 8.4 Hz, 1H, Ar–H), 7.79 (dd, *J*_*1*_ = 8.4 Hz, *J*_*2*_ = 1.7 Hz, 1H, Ar–H), 7.37 (d, *J* = 1.7 Hz, 1H, Ar–H), 7.23–7.14 (m, 2H, 2 × Ar–H), 7.09–7.01 (m, 2H, 2 × Ar–H), 3.83 (s, 3H, COOC*H*_*3*_), 3.79 (s, 3H, Ar-O-C*H*_*3*_); MS (ESI+) C_15_H_13_NO_6_ *m*/*z*: 382,0 [M + C_2_H_6_OS + H]^+^.

#### General procedure H.

Acetone was added to compound **12** (1 equiv.), K_2_CO_3_ (2 equiv.) and the corresponding alkyl halogenide (1 equiv.). A tip of spatula of KI was added and the reaction mixture was stirred at 60 °C overnight. The solvent was evaporated under reduced pressure.

#### Methyl 3-((4-methoxybenzyl)oxy)-4-nitrobenzoate (19k).

The reaction was performed according to the general procedure H using 4-methoxybenzyl chloride (0.753 g, 5.58 mmol) as starting material. The product was purified by column chromatography using EtOAc: hexane = 1:2 as the mobile phase. Yield: 1.50 g (93 %); yellow solid; R_f_ (EtOAc: hexane = 1:2) = 0.25; ^1^H NMR (400 MHz, CDCl_3_): *δ* 7.86–7.79 (m, 2H, 2 × Ar–*H*), 7.69 (dd, *J*_*1*_ = 8.4 Hz, *J*_*2*_ = 1.6 Hz, 1H, Ar–*H*), 7.41–7.36 (m, 2H, 2 × Ar–*H*), 6.95–6.90 (m, 2H, 2 × Ar–*H*), 5.22 (s, 2H, Ar-O-C*H*_*2*_ -Ar), 3.96 (s, 3H, COOC*H*_*3*_), 3.82 (s, 3H, Ar-O-C*H*_*3*_); MS (ESI+) C_16_H_15_NO_6_ *m*/*z*: 380,8 [M + CH_3_CN + Na]^+^.

#### Methyl 4-nitro-3-(pyridin-4-ylmethoxy)benzoate (19l).

The reaction was performed according to the general procedure H using 4-(chloromethyl)pyridine (2.06 g, 26.6 mmol) as starting material. Methanol was added to the residue after evaporation and the precipitate was filtered off to yield pure product. Yield: 2.05 g (62 %); brown solid; R_f_ (EtOAc: hexane = 1:1) = 0.16; ^1^H NMR (400 MHz, DMSO-*d*_6_): *δ* 8.65–8.57 (m, 2H, 2 × Ar–*H*), 8.08 (d, *J* = 8.4 Hz, 1H, Ar–*H*), 7.87 (d, *J* = 1.1 Hz, 1H, Ar–*H*), 7.72 (dd, *J*_1_ = 8.4 Hz, *J*_*2*_ = 1.6 Hz, 1H, Ar–*H*), 7.55–7.37 (m, 2H, 2 × Ar–*H*), 5.50 (s, 2H, Ar-O-C*H*_*2*_), 3.91 (s, 3H, COOC*H*_*3*_); MS (ESI +) C_14_H_12_N_2_O_5_ *m*/*z* = 288,9 [M+H]^+^.

#### *tert*-Butyl (3-(5-carbamoyl-2-nitrophenoxy)benzyl)carbamate (20a).

The synthesis was performed according to the general procedure B using compound **19a** (0.640 g, 1.59 mmol) as starting material. The product was purified by column chromatography using EtOAc:hexane = 2:1 as the mobile phase. Yield: 0.456 g (74 %); yellow solid; R_f_ (EtOAc: hexane = 2:1) = 0.14; ^1^H NMR (400 MHz, CDCl_3_): *δ* 7.99 (d, *J* = 8.4 Hz, 1H, Ar–*H*), 7.59 (dd, *J*_*1*_ = 8.4 Hz, *J*_*2*_ = 1.1 Hz, 1H, Ar–*H*), 7.43–7.35 (m, 2H, 2 × Ar–*H*), 7.13 (d, *J* = 7.8 Hz, 1H, Ar–*H*), 7.04–6.97 (m, 2H, 2 × Ar–*H*), 6.39 (s, 1H, CON*H*_*2*_*-H*_*A*_), 5.58 (s, 1H, CON*H*_*2*_*-H*_*B*_), 5.00 (s, 1H, N*H*COO), 4.31 (d, *J* = 6.0 Hz, 2H. Ar-C*H*_*2*_-NH), 1.41 (s, 9H, C(C*H*_*3*_)_*3*_); MS (ESI +) C_19_H_21_N_3_O_6_ *m*/*z* = 387.6 [M+H]^+^.

#### *tert*-Butyl (4-(5-carbamoyl-2-nitrophenoxy)benzyl)carbamate (20b).

The synthesis was performed according to the general procedure B using compound **19b** (0.766 g, 1.90 mmol) as starting material. The product was purified by column chromatography using EtOAc:hexane = 2:1 as the mobile phase. Yield: 0.556 g (75 %); yellow solid; R_f_ (EtOAc: hexane = 2:1) = 0.12; ^1^H NMR (400 MHz, CDCl_3_): *δ* 7.97 (d, *J* = 8.4 Hz, 1H, Ar–*H*), 7.56 (dd, *J*_*1*_ = 8.4 Hz, *J*_*2*_ = 1.6 Hz, 1H, Ar–*H*), 7.44 (d, *J* = 1.6 Hz, 1H, Ar–*H*), 7.34–7.29 (m, 2H, 2 × Ar–*H*), 7.05–6.99 (m, 2H, 2 × Ar–*H*), 6.17 (s, 1H, CON*H*_*2*_*-H*_*A*_), 5.66 (s, 1H CON*H*_*2*_*-H*_*B*_), 4.95 (s, 1H, N*H*COO), 4.32 (d, *J* = 5.8 Hz, 2H, Ar-C*H*_*2*_-NH), 1.47 (s, 9H, C(C*H*_*3*_)_*3*_); MS (ESI +) C_19_H_21_N_3_O_6_ *m*/*z* = 331.7 [M-(*t*-Bu) + H]^+^.

#### 1-Boc-4-(5-carbamoyl-2-nitrophenyl)piperazine (20c).

The synthesis was performed according to the general procedure B using compound **19c** (0.950 g, 2.60 mmol) as starting material. Yield: 0.901 g (99 %); orange solid; ^1^H NMR (400 MHz, CDCl_3_): *δ* 7.82 (d, *J* = 8.3 Hz, 1H, Ar–*H*), 7.66 (d, *J* = 1.8 Hz, 1H, Ar–*H*), 7.35 (dd, *J*_*1*_ = 8.3 Hz, *J*_*2*_ = 1.8 Hz, 1H, Ar–*H*), 6.06 (s, 1H, CON*H*_*2*_*-H*_*A*_), 5.71 (s, 1H, CON*H*_*2*_*-H*_*B*_), 3.72–3.43 (m, 4H, 2 × C*H*_*2*_), 3.10–3.01 (m, 4H, 2 × C*H*_*2*_), 1.48 (s, 9H, C(C*H*_*3*_)_*3*_); MS (ESI +) C_16_H_22_N_4_O_5_ *m*/*z* = 250.7 [M-Boc + H]^+^.

#### 1-Boc-4-((5-carbamoyl-2-nitrophenyl)amino)piperidine (20d).

The synthesis was performed according to the general procedure B using compound **19d** (0.766 g, 1.90 mmol) as starting material. Yield: 0.860 g (96 %); red solid; ^1^H NMR (400 MHz, CDCl_3_): *δ* 8.25 (d, *J* = 8.8 Hz, 1H, Ar–*H*), 8.10 (d, *J* = 7.6 Hz, 1H, Ar-N*H*), 7.47 (d, *J* = 1.7 Hz, 1H, Ar–*H*), 6.85 (dd, *J*_*1*_ = 8.8 Hz, *J*_*2*_ = 1.7 Hz, 1H, Ar–*H*), 6.08 (s, 1H, CON*H*_*2*_*-H*_*A*_), 5.68 (s, 1H, CON*H*_*2*_*-H*_*B*_), 4.11–3.99 (m, 2H, C*H*_*2*_), 3.84–3.74 (m, 1H, C*H*), 3.13–3.00 (m, 2H, C*H*_*2*_), 2.14–2.04 (m, 2H, C*H*_*2*_), 1.48 (s, 9H, C (C*H*_*3*_)_*3*_), the signal for the remaining C*H*_*2*_ is overlapped with solvent; MS (ESI −) C_17_H_24_N_4_O_5_ *m*/*z* = 264.8 [M-Boc + H]^−^.

#### 2-Boc-7-(5-carbamoyl-2-nitrophenyl)-2,7-diazaspiro[4.4]nonane (20e).

The synthesis was performed according to the general procedure B using compound **19e** (0.800 g, 1.97 mmol) as starting material. Yield: 0.752 g (94 %); orange solid; R_f_ (EtOAc:hexane = 1:1) = 0.05; ^1^H NMR (400 MHz, CDCl_3_): *δ* 7.78 (d, *J* = 8.4 Hz, 1H, Ar–*H*), 7.47–7.43 (m, 1H, Ar–*H*), 7.00 (dd, *J*_*1*_ = 8.4 Hz, *J*_*2*_ = 1.0 Hz, 1H, Ar–*H*), 6.12 (s, 1H, CON*H*_*2*_*-H*_*A*_), 5.75 (s, 1H, CON*H*_*2*_*-H*_*B*_), 3.55–3.08 (m, 8H, 4 × C*H*_*2*_), 2.04–1.85 (m, 4H, 2 × C*H*_*2*_), 1.46 (s, 9H, C(C*H*_*3*_)_*3*_); ^13^C NMR (101 MHz, DMSO-*d*_6_) *δ* 167.2, 154.0, 142.2, 138.8, 137.9, 126.8, 116.2, 115.1, 78.9, 59.0, 54.5, 54.2, 49.6, 48.6, 47.7, 45.3, 45.1, 34.3, 34.2, 33.6, 28.6 rotamers are present in the spectra; MS (ESI +) C_19_H_26_N_4_O_5_ *m*/*z* = 390.5 [M+H]^+^.

#### *tert*-Butyl (1-(5-carbamoyl-2-nitrophenyl)piperidin-4-yl)carbamate (20f).

The synthesis was performed according to the general procedure B using compound **19f** (0.800 g, 1.97 mmol) as starting material. Yield: 0.656 g (91 %); yellow solid: R_f_ (EtOAc: hexane = 1:1) = 0.08; ^1^H NMR (400 MHz, CDCl_3_): *δ* 7.80 (d, *J* = 8.4 Hz, 1H, Ar–*H*), 7.64 (d, *J* = 1.5 Hz, 1H, Ar–*H*), 7.29 (dd, *J*_*1*_ = 8.4 Hz, *J*_*2*_ = 1.8 Hz, 1H, Ar–*H*), 6.08 (s, 1H, CON*H*_*2*_*-H*_*A*_), 5.72 (s, 1H, CON*H*_*2*_*-H*_*B*_), 4.51 (d, *J* = 7.3 Hz, 1H, CON*H*CH), 3.70–3.57 (m, 1H, C*H*), 3.34–3.24 (m, 2H, C*H*_*2*_), 3.01–2.91 (m, 2H, C*H*_*2*_), 2.09–2.00 (m, 2H, C*H*_*2*_), 1.46 (s, 9H, C(C*H*_*3*_)_*3*_), remaining C*H*_*2*_ is overlapped with solvent; MS (ESI +) C_17_H_24_N_4_O_5_ *m*/*z* = 364.6 [M+H]^+^.

#### 1-Boc-4-((5-carbamoyl-2-nitrophenoxy)methyl)piperidine (20g).

The synthesis was performed according to the general procedure B using compound 19g (2.20 g, 5.58 mmol) as starting material. The product was purified by column chromatography using EtOAc:hexane = 2:1 as the mobile phase. Yield: 0.901 g (43 %); yellow solid; R_f_ (EtOAc: hexane = 2:1) = 0.13; ^1^H NMR (400 MHz, DMSO-*d*_6_): *δ* 8.22 (s, 1H, CON*H*_*2*_-*H*_*a*_), 7.94 (d, *J* = 8.4 Hz, 1H, Ar–*H*), 7.73 (d, *J* = 1.6 Hz, 1H, Ar–*H*), 7.71 (s, 1H, CON*H*_*2*_-*H*_*b*_), 7.56 (dd, *J*_*1*_ = 8.4 Hz, *J*_*2*_ = 1.6 Hz, 1H, Ar–*H*), 4.09 (d, *J* = 6.2 Hz, 2H, OC*H*_*2*_-piperidine), 4.02–3.91 (m, 2H, C*H*_*2*_), 2.84–2.63 (m, 2H, C*H*_*2*_), 2.04–1.88 (m, 1H, C*H*), 1.78–1.68 (m, 2H, C*H*_*2*_), 1.40 (s, 9H, C(C*H*_*3*_)_*3*_), 1.27–1.10 (m, 2H, C*H*_*2*_); MS (ESI+) C_18_H_25_N_3_O_6_ *m*/*z*: 381.0 [M+H]^+^.

#### 1-Boc-4-(5-carbamoyl-2-nitrobenzoyl)piperazine (20h).

The synthesis was performed according to the general procedure B using compound **19h** (0.860 g, 2.19 mmol) as starting material. Yield: 0.752 g (91 %); white solid; ^1^H NMR (400 MHz, DMSO-*d*_6_) *δ* 8.33–8.27 (m, 2H, CON*H*_*2*_*-H*_*A*_ and Ar–*H*), 8.12 (dd, *J*_*1*_ = 8.6 Hz, *J*_*2*_ = 1.9 Hz, 1H), 7.96 (d, *J* = 1.9 Hz, 1H), 7.82 (s, 1H, CON*H*_*2*_*-H*_*B*_), 3.68–3.58 (m, 2H, C*H*_*2*_), 3.49–3.39 (m, 2H, C*H*_*2*_), 3.30–3.18 (m, 4H, 2 × C*H*_*2*_), 1.41 (s, 9H, C (C*H*_*3*_)_*3*_); MS (ESI−) C_16_H_20_N_4_O_6_ *m*/*z* = 363.5 [M-CH_3_-H]^−^.

#### 3-((1-Methyl-1*H*-indol-5-yl)oxy)-4-nitrobenzamide (20i).

The synthesis was performed according to the general procedure B using compound **19i** (0.630 g, 1.93 mmol) as starting material. The product was purified by column chromatography using EtOAc:hexane = 2:1 as the mobile phase. Yield: 0.427 g (71 %); yellow solid; R_f_ (EtOAc: hexane = 2:1) = 0.13; ^1^H NMR (400 MHz, DMSO-*d*_6_): *δ* 8.19 (s, 1H, CON*H*_*2*_*-H*_*A*_), 8.10 (d, *J* = 8.4 Hz, 1H, Ar–*H*), 7.68 (dd, *J*_*1*_ = 8.4 Hz, *J*_*2*_ = 1.7 Hz, 1H, 2 × Ar–*H*), 7.65 (s, 1H, CON*H*_*2*_*-H*_*A*_), 7.54 (d, *J* = 8.8 Hz, 1H, Ar–*H*), 7.43 (d, *J* = 3.0 Hz, 1H, Ar–*H*), 7.35 (d, *J* = 1.7 Hz, 1H, Ar–*H*), 7.34 (d, *J* = 2.4 Hz, 1H, Ar–*H*), 6.98 (dd, *J*_*1*_ = 8.8 Hz, *J*_*2*_ = 2.4 Hz, 1H, Ar–*H*), 6.44 (dd, *J*_*1*_ = 3.0 Hz, *J*_*2*_ = 0.7 Hz, 1H, Ar–*H*), 3.83 (s, 3H, indole-C*H*_*3*_); MS (ESI +) C_16_H_13_N_3_O_4_ *m*/*z* = 311.7 [M+H]^+^.

#### 3-(4-Methoxyphenoxy)-4-nitrobenzamide (20j).

The synthesis was performed according to the general procedure B using compound **19j** (0.700 g, 2.31 mmol) as starting material. The product was purified by column chromatography using EtOAc:hexane = 1:1 as the mobile phase. Yield: 0.462 g (70 %); yellow solid; R_f_ (EtOAc: hexane = 1:1) = 0.10; ^1^H NMR (400 MHz, DMSO-*d*_6_) *δ* 8.22 (s, 1H, CON*H*_*2*_-*H*_*a*_), 8.11 (d, *J* = 8.4 Hz, 1H, Ar–*H*), 7.73 (dd, *J*_*1*_ = 8.4 Hz, *J*_*2*_ = 1.7 Hz, 1H, Ar–*H*), 7.70 (s, 1H, CON*H*_*2*_*-H*_*b*_), 7.41 (d, *J* = 1.7 Hz, 1H, Ar–*H*), 7.15–7.08 (m, 2H, 2 × Ar–*H*), 7.07–6.98 (m, 2H, 2 × Ar–*H*), 3.78 (s, 3H, ArOC*H*_*3*_); MS (ESI−) C_14_H_12_N_2_O_5_ *m*/*z*: 286.8 [M − H]^−^.

#### 3-((4-Methoxybenzyl)oxy)-4-nitrobenzamide (20k).

The synthesis was performed according to the general procedure B using compound **19**k (0.704 g, 2.22 mmol) as starting material. A precipitate formed in the reaction mixture which was filtered off to yield pure product. Yield: 0.384 g (57 %); yellow solid; R_f_ (EtOAc: hexane = 1:1) = 0.12; ^1^H NMR (400 MHz, DMSO-*d*_6_): *δ* 8.23 (s, 1H, CON*H*_*2*_ -*H*_*a*_), 7.95 (d, *J* = 8.4 Hz, 1H, Ar–*H*), 7.87 (d, *J* = 1.6 Hz, 1H, Ar–*H*), 7.72 (s, 1H, CON*H*_*2*_*-H*_*b*_*),* 7.58 (dd, *J*_*1*_ = 8.4 Hz, J_2_ = 1.6 Hz, 1H, Ar–*H*), 7.43–7.35 (m, 2H, 2 × Ar–*H*), 7.02–6.94 (m, 2H, 2 × Ar–*H),* 5.27 (s, 2H, OC*H*_*2*_-Ar), 3.76 (s, 3H, ArOC*H*_*3*_); MS (ESI+) C_15_H_14_N_2_O_5_ *m*/*z*: 324.8 [M+Na]^+^.

#### 4-Nitro-3-(pyridin-4-ylmethoxy)benzamide (20l).

The synthesis was performed according to the general procedure B using compound **19i** (0.630 g, 1.93 mmol) as starting material. A precipitate formed in the reaction mixture, which was filtered off to yield pure product. Yield: 0.811 g (86 %); light brown solid; R_f_ (DCM: MeOH = 20:1) = 0.08; ^1^H NMR (400 MHz, DMSO-*d*_6_): *δ* 8.64–8.60 (m, 2H, 2 × Ar–*H*), 8.24 (s, 1H, CON*H*_*2*_ -*H*_*a*_), 8.03 (d, *J* = 8.4 Hz, 1H, Ar–*H*), 7.84 (d, *J* = 1.7 Hz, 1H, Ar–*H*), 7.74 (s, 1H, CON*H*_*2*_ -*H*_*b*_), 7.63 (dd, *J*_*1*_ = 8.4 Hz, *J*_*2*_ = 1.7 Hz, 1H, Ar–*H*), 7.47–7.42 (m, 2H, 2 × Ar–H), 5.45 (s, 2H, ArOC*H*_*2*_); MS (ESI +) C_13_H_11_N_3_O_4_ *m*/*z* = 273.7 [M+H]^+^.

#### *tert*-Butyl (3-(2-amino-5-carbamoylphenoxy)benzyl)carbamate (21a).

The synthesis was performed according to the general procedure C using compound **20a** (0.4300 g, 1.11 mmol) as starting material. Yield: 0.384 g (97 %); grey solidified oil; R_f_ (EtOAc: hexane = 2:1) = 0.10; ^1^H NMR (400 MHz, CDCl_3_): *δ* 7.46 (dd, *J*_*1*_ = 8.3 Hz, *J*_*2*_ = 1.9 Hz, 1H, Ar–*H*), 7.34 (d, *J* = 1.9 Hz, 1H, Ar–*H*), 7.28 (t, *J* = 8.1 Hz, 1H, Ar–*H*), 7.01 (d, *J* = 7.7 Hz, 1H, Ar–*H*), 6.92 (s, 1H, Ar–*H*), 6.87 (dd, *J*_*1*_ = 8.0 Hz, *J*_*2*_ = 2.1 Hz, 1H, Ar–*H*), 6.81 (d, *J* = 8.3 Hz, 1H, Ar–*H*), 6.10–5.32 (m, 2H, CON*H*_*2*_), 4.92 (s, 1H, N*H*COO), 4.28 (d, *J* = 5.7 Hz, 2H, Ar-C*H*_*2*_-NH), 4.19 (s, 2H, Ar-N*H*_*2*_), 1.44 (s, 9H, C(C*H*_*3*_)_*3*_); MS (ESI +) C_19_H_23_N_3_O_4_ *m*/*z* = 357.9 [M+H]^+^.

#### *tert*-Butyl (4-(2-amino-5-carbamoylphenoxy)benzyl)carbamate (21b).

The synthesis was performed according to the general procedure C using compound **20b** (0.520 g, 1.34 mmol) as starting material. Yield: 0.471 g (98 %); brown solidified oil; ^1^H NMR (400 MHz, CDCl_3_): *δ* 7.44 (dd, *J*_*1*_ = 8.3 Hz, *J*_*2*_ = 2.0 Hz, 1H, Ar–*H*), 7.33 (d, *J* = 2.0 Hz, 1H), 7.26–7.22 (m, 2H, 2 × Ar–*H*), 6.97–6.91 (m, 2H, 2 × Ar–*H*), 6.81 (d, *J* = 8.3 Hz, 1H, Ar–*H*), 5.81–5.32 (m, 2H, CON*H*_*2*_), 4.85 (s, 1H, N*H*COO), 4.28 (d, *J* = 5.8 Hz, 2H), 4.20 (s, 2H, Ar-C*H*_*2*_-NH), 1.46 (s, 9H, C(C*H*_*3*_)_*3*_); MS (ESI +) C_19_H_23_N_3_O_4_ *m*/*z* = 357.8 [M+H]^+^.

#### 1-Boc-4-(2-amino-5-carbamoylphenyl)piperazine (21c).

The synthesis was performed according to the general procedure C using compound **20c** (0.400 g, 1.09 mmol) as starting material. Yield: 0.334 g (95 %); brown solid; ^1^H NMR (400 MHz, CDCl_3_): *δ* 7.55 (d, *J* = 1.9 Hz, 1H, Ar–*H*), 7.36 (dd, *J*_*1*_ = 8.2 Hz, *J*_*2*_ = 2.0 Hz, 1H, Ar–*H*), 6.71 (d, *J* = 8.2 Hz, 1H, Ar–*H*), 6.00–5.38 (m, 2H, CON*H*_*2*_), 4.35 (s, 2H, Ar-N*H*_*2*_), 3.72–3.39 (m, 4H, 2 × C*H*_*2*_), 2.91–2.82 (m, 4H, 2 × C*H*_*2*_), 1.49 (s, 9H, C (C*H*_*3*_)_*3*_); MS (ESI +) C_16_H_24_N_4_O_3_ *m*/*z* = 321.1 [M+H]^+^.

#### 1-Boc-4-((2-amino-5-carbamoylphenyl)amino)piperidine (21d).

The synthesis was performed according to the general procedure C using compound **20d** (0.450 g, 1.19 mmol) as starting material. The residue was taken up in EtOAc and then hexane was added to form a precipitate which was then filtered off to yield a pure product. Yield: 0.396 g (100 %); grey solid; ^1^H NMR (400 MHz, DMSO-*d*_6_): *δ* 7.51 (s, 1H), 7.06–7.00 (m, 2H, 2 × Ar–*H*), 6.82 (s, 1H), 6.50 (d, *J* = 7.8 Hz, 1H, Ar–*H*), 5.06 (s, 2H, CON*H*_*2*_), 4.27 (d, *J* = 7.8 Hz, 1H, Ar-N*H*), 3.54–3.42 (m, 1H, C*H*), 3.02–2.80 (m, 2H, C*H*_*2*_), 1.97–1.86 (m, 2H, C*H*_*2*_), 1.41 (s, 9H, C(C*H*_*3*_)_*3*_), 1.33–1.18 (m, 2H, C*H*_*2*_); MS (ESI +) C_17_H_26_N_4_O_3_ *m*/*z* = 335.1 [M+H]^+^.

#### 2-Boc-7-(2-amino-5-carbamoylphenyl)-2,7-diazaspiro[4.4] nonane (21e).

The synthesis was performed according to the general procedure C using compound **20e** (0.490 g, 1.25 mmol) as starting material. Yield: 0.449 g (99 %); grey solid; ^1^H NMR (400 MHz, CDCl_3_): 7.59–7.55 (m, 1H, Ar–*H*), 7.30 (dd, *J*_*1*_ = 8.2 Hz, *J*_*2*_ = 1.6 Hz, 1H, Ar–*H*), 6.70 (dd, *J*_*1*_ = 8.2 Hz, *J*_*2*_ = 2.3 Hz, 1H, Ar–*H*), 5.70 (s, 2H, CON*H*_*2*_), 4.18 (s, 2H, Ar-N*H*_*2*_), 3.47–2.95 (m, 8H, 4 × C*H*_*2*_), 2.01–1.86 (m, 4H, 2 × C*H*_*2*_), 1.47 (s, 9H, C(C*H*_*3*_)_*3*_); ^13^C NMR (101 MHz, DMSO-*d*_6_) *δ* 168.6, 154.1, 145.7, 135.5, 123.6, 122.2, 118.0, 113.6, 78.7, 78.6, 60.2, 60.1, 57.2, 56.8, 50.0, 48.1, 47.3, 45.6, 45.4, 36.8, 36.1, 35.2, 35.1, 28.7, rotamers are present in the spectra; MS (ESI +) C_19_H_28_N_4_O_3_ *m*/*z* = 360.7 [M+H]^+^.

#### *tert*-Butyl (1-(2-amino-5-carbamoylphenyl)piperidin-4-yl)carbamate (21f).

The synthesis was performed according to the general procedure C using compound **20f** (0.450 g, 1.23 mmol) as starting material. Yield: 0.412 g (100 %); grey solid; R_f_ (DCM:MeOH = 20:1) = 0.06; ^1^H NMR (400 MHz, CDCl_3_): *δ* 7.56 (d, *J* = 1.6 Hz, 1H, Ar–*H*), 7.35 (dd, *J*_*1*_ = 8.2 Hz, *J*_*2*_ = 1.6 Hz, 1H, Ar–*H*), 6.70 (d, *J* = 8.2 Hz, 1H, Ar–*H*), 5.73 (s, 2H, CON*H*_*2*_), 4.59–4.51 (m, 1H, CON*H*CH), 4.32 (s, 2H, Ar-N*H*_*2*_), 3.69–3.56 (m, 1H, C*H*), 3.13–3.02 (m, 2H, C*H*_*2*_), 2.82–2.66 (m, 2H, C*H*_*2*_), 2.12–1.98 (m, 2H, C*H*_*2*_), 1.61–1.50 (m, 2H, C*H*_*2*_), 1.47 (s, 9H, C (C*H*_*3*_)_*3*_); MS (ESI +) C_17_H_26_N_4_O_3_ *m*/*z* = 334.7 [M+H]^+^.

#### 1-Boc-4-((2-amino-5-carbamoylphenoxy)methyl)piperidine (21g).

The synthesis was performed according to the general procedure C using compound **20g** (0.600 g, 1.58 mmol) as starting material. Yield: 0.540 g (98 %); white solid; R_f_ (EtOAc:hexane = 2:1) = 0,11; ^1^H NMR (400 MHz, DMSO-*d*_6_): *δ* 7.58 (s, 1H, CON*H*_*2*_ -*H*_*a*_), 7.31–7.26 (m, 2H, 2 × Ar–*H*), 6.89 (s, 1H, CON*H*_*2*_ -*H*_*b*_), 6.58 (d, *J* = 7.9 Hz, 1H, Ar–*H*), 5.22 (s, 2H, Ar-N*H*_*2*_), 4.03–3.95 (m, 2H, C*H*_*2*_), 3.83 (d, *J* = 6.4 Hz, 2H, OC*H*_*2*_-piperidin), 2.86–2.65 (m, 2H, C*H*_*2*_), 2.01–1.87 (m, 1H, C*H*), 1.87–1.77 (m, 2H, C*H*_*2*_), 1.40 (s, 9H, C(C*H*_*3*_)_*3*_), 1.25–1.11 (m, 2H, C*H*_*2*_); MS (ESI +) C_18_H_27_N_3_O_4_ *m*/*z* = 349.9 [M+H]^+^.

#### 1-Boc-4-(2-amino-5-carbamoylbenzoyl)piperazine (21h).

The synthesis was performed according to the general procedure C using compound **20h** (0.505 g, 1.33 mmol) as starting material. A precipitate formed in a cooled (ice bath) reaction mixture. The precipitate was filtered off to yield pure product. Yield: 0.464 g (100 %); off-white solid; ^1^H NMR (400 MHz, DMSO-*d*_6_) *δ* 7.67–7.61 (m, 2H, Ar–*H* and CON*H*_*2*_-*H*_*A*_), 7.58 (d, *J* = 2.1 Hz, 1H, Ar–*H*), 6.97 (s, 1H, CON*H*_*2*_*-H*_*B*_), 6.67 (d, *J* = 8.6 Hz, 1H, Ar–*H*), 5.72 (s, 2H, Ar-N*H*), 3.60–3.34 (m, 8H, 4 × C*H*_*2*_), 1.41 (s, 9H, C(C*H*_*3*_)_*3*_); MS (ESI +) C_17_H_24_N_4_O_4_ *m*/*z*: 349.0 [M+H]^+^.

#### 4-Amino-3-((1-methyl-1*H*-indol-5-yl)oxy)benzamide (21i).

The synthesis was performed according to the general procedure C using compound **20i** (0.505 g, 1.33 mmol) as starting material. Yield: 0.303 g (84 %); brown solid; ^1^H NMR (400 MHz, DMSO-*d*_6_) *δ* 7.55 (s, 1H, CON*H*_*2*_*-H*_*A*_), 7.45–7.39 (m, 2H, 2 × Ar–*H*), 7.34 (d, *J* = 3.0 Hz, 1H, Ar–*H*), 7.17 (d, *J* = 1.9 Hz, 1H, Ar–*H*), 7.09 (d, *J* = 2.2 Hz, 1H, Ar–*H*), 6.92–6.83 (m, 2H, Ar–*H* and CON*H*_*2*_*-H*_*B*_), 6.73 (d, *J* = 8.3 Hz, 1H, Ar–*H*), 6.36 (dd, *J*_*1*_ = 3.0 Hz, *J*_*2*_ = 0.7 Hz, 1H, Ar–*H*), 5.48 (s, 2H, Ar-N*H*_*2*_), 3.79 (s, 3H, indole-C*H*_*3*_); MS (ESI +) C_16_H_15_N_3_O_2_ *m*/*z*: 281.9 [M+H]^+^.

#### 4-Amino-3-(4-methoxyphenoxy)benzamide (21j).

To compound **20j** (0.430 g, 1.49 mmol) and solid iron (0.833 g, 14.9 mmol) acetic acid (15 mL) was added, and the reaction mixture was stirred overnight at room temperature. The mixture was diluted with water (10 mL) and methanol (10 mL) and filtered over Celite^®^ 545. The solvent was evaporated under reduced pressure. The residue was taken up in EtOAc (45 mL) and washed with NaHCO_3_ (45 mL) and NaCl (20 mL), dried over Na_2_SO_4_ and filtered. The solvent was then evaporated under reduced pressure. Yield: 0.279 g (73 %); light red solid; R_f_ (EtOAc:hexane = 2:1) = 0.24; ^1^H NMR (400 MHz, DMSO-*d*_6_): *δ* 7.58 (s, 1H, CON*H*_*2*_-*H*_*a*_), 7.44 (dd, *J*_*1*_ = 8.3 Hz, *J*_*2*_ = 2.0 Hz, 1H, Ar–*H*), 7.23 (d, *J* = 2.0 Hz, 1H, Ar–*H*), 6.97–6.87 (m, 5H, 4 × Ar–*H* in CON*H*_*2*_-*H*_*b*_), 6.73 (d, *J* = 8.3 Hz, 1H, Ar–*H*), 5.49 (s, 2H, Ar-N*H*_*2*_), 3.73 (s, 3H, ArOC*H*_*3*_); MS (ESI+) C_14_H_14_N_2_O_3_ *m*/*z*: 258.8 [M+H]^+^.

#### 4-Amino-3-((4-methoxybenzyl)oxy)benzamide (21k).

To compound **20k** (0.300 g, 0.990 mmol) and solid iron (0.554 g, 9.92 mmol) acetic acid (10 mL) was added, and the reaction mixture was stirred overnight at room temperature. The mixture was diluted with water (10 mL) and methanol (10 mL) and filtered over Celite^®^ 545. The solvent was evaporated under reduced pressure. The residue was taken up in EtOAc (35 mL) and washed with NaHCO_3_ (35 mL) and NaCl (20 mL), dried over Na_2_SO_4_ and filtered. The solvent was then evaporated under reduced pressure. Yield: 0.214 g (79 %); light brown solid; R_f_ (DCM: MeOH = 15:1) = 0.21; ^1^H NMR (400 MHz, DMSO-*d*_6_): *δ* 7.58 (s, 1H, CON*H*_*2*_-*H*_*a*_), 7.46–7.40 (m, 3H, 3 × Ar–*H*), 7.30 (dd, *J*_*1*_ = 8.2 Hz, *J*_*2*_ = 1.8 Hz, 1H, Ar–*H*), 7.00–6.92 (m, 2H, 2 × Ar–*H*), 6.90 (s, 1H, CON*H*_*2*_-*H*_*b*_), 6.61 (d, *J* = 8.2 Hz, 1H, Ar–*H)*, 5.22 (s, 2H, OC*H*_*2*_Ar), 5.04 (s, 2H, Ar-N*H*_*2*_), 3.76 (s, 3H, ArOC*H*_*3*_); MS (ESI+) C_15_H_16_N_2_O_3_ *m*/*z* = 272.8 [M+H]^+^.

#### 4-Amino-3-(pyridin-4-ylmethoxy)benzamide (21l).

To compound **20j** (0.500 g, 1.83 mmol) and solid iron (1.02 g, 18.3 mmol) acetic acid (15 mL) was added, and the reaction mixture was stirred overnight at room temperature. The mixture was diluted with water (10 mL) and methanol (10 mL) and filtered over Celite^®^ 545. The solvent was evaporated under reduced pressure. The residue was taken up in EtOAc (45 mL) and washed with NaHCO_3_ (45 mL) and NaCl (20 mL), dried over Na_2_SO_4_ and filtered. The solvent was then evaporated under reduced pressure. Yield: 0.284 g (64 %); light brown solid; R_f_ (DCM: MeOH:NH_4_OH = 20:1:0.1) = 0.13; ^1^H NMR (400 MHz, DMSO-*d*_6_): *δ* 8.67–8.52 (m, 2H, 2 × Ar–*H*), 7.58 (s, 1H, CON*H*_*2*_ -*H*_*a*_), 7.57–7.47 (m, 2H, 2 × Ar–*H*), 7.41 (d, *J* = 1.8 Hz, 1H, Ar–*H*), 7.32 (dd, *J*_*1*_ = 8.2 Hz, *J*_*2*_ = 1.8 Hz, 1H, Ar–*H*), 6.92 (s, 1H, CON*H*_*2*_-*H*_*b*_), 6.64 (d, *J* = 8.2 Hz, 1H, Ar–*H*), 5.42 (s, 2H, Ar-N*H*_*2*_), 5.20 (s, 2H, ArOC*H*_*2*_); MS (ESI +) C_13_H_13_N_3_O_2_ *m*/*z* = 243.7 [M+H]^+^.

#### *tert*-Butyl (3-(5-carbamoyl-2-(3,4-dichloro-5-methyl-1*H*-pyrrole-2-carboxamido)phenoxy)benzyl)carbamate (22a).

The synthesis was performed according to the general procedure D using compound **21a** (0.360 g, 1.01 mmol) as starting material. The residue was taken up in DCM (100 mL) and extracted with 0.5 M HCl (50 mL). A precipitate that formed was filtered off and was further purified by column chromatography using DCM:MeOH = 15:1 as the mobile phase. Yield: 0.140 g (26 %); light brown solid; R_f_ (DCM:MeOH = 15:1) = 0.16; ^1^H NMR (400 MHz, DMSO-*d*_6_) *δ* 12.47 (s, 1H, pyrrole-N*H*), 9.17 (s, 1H, pyrrole-CON*H*-Ar), 8.51 (d, *J* = 8.6 Hz, 1H, Ar–*H*), 7.95 (s, 1H, CON*H*_*2*_*-H*_*A*_), 7.78 (dd, *J*_*1*_ = 8.6 Hz, *J*_*2*_ = 1.8 Hz, 1H, Ar–*H*), 7.55 (d, *J* = 1.8 Hz, 1H, Ar–*H*), 7.44–7.33 (m, 3H, Ar–*H* and CON*H*_*2*_*-H*_*B*_ and N*H*COO or Ar–*H*), 7.04 (d, *J* = 7.2 Hz, 1H Ar–*H*), 6.97–6.89 (m, 2H, Ar–*H* and Ar–*H* or N*H*COO), 4.11 (d, *J* = 6.2 Hz, 2H, Ar-C*H*_*2*_-NH), 2.22 (s, 3H, pyrrole-C*H*_*3*_), 1.35 (s, 9H, C (C*H*_*3*_)_*3*_); MS (ESI −) C_25_H_26_Cl_2_N_4_O_5_ *m*/*z* = 531.3 [M+H]^−^.

#### *tert*-Butyl (4-(5-carbamoyl-2-(3,4-dichloro-5-methyl-1*H*-pyrrole-2-carboxamido)phenoxy)benzyl)carbamate (22b).

The synthesis was performed according to the general procedure D using compound **21b** (0.360 g, 1.01 mmol) as starting material. The residue was taken up in DCM (100 mL) and extracted with 1 M HCl (50 mL). A precipitate that formed was filtered off and it contained both the product and the Boc deprotected analogue. Therefore, the mixture was used in the next step without further purification. MS (ESI −) C_25_H_26_Cl_2_N_4_O_5_ *m*/*z* = 531.1 [M − H]^−^; MS (ESI +) C_25_H_26_Cl_2_N_4_O_5_ *m*/*z* = 533.2 [M+H]^+^.

#### 1-Boc-4-(5-carbamoyl-2-(3,4-dichloro-5-methyl-1*H*-pyrrole-2-carboxamido)phenyl)piperazine (22c).

The synthesis was performed according to the general procedure D using compound **21c** (0.344 g, 1.07 mmol) as starting material. The residue was purified by column chromatography using DCM:MeOH = 15:1 as the mobile phase. The solvent of the collected fractions was evaporated, and the residue was additionally washed with MeOH to yield pure product. Yield: 0.154 g (29 %); off-white solid; R_f_(DCM:MeOH = 15:1) = 0.33; ^1^H NMR (400 MHz, DMSO-*d*_6_): *δ* 12.43 (s, 1H, pyrrole-N*H*), 9.77 (s, 1H, pyrrole-CON*H*-Ar), 8.44 (d, *J* = 8.7 Hz, 1H, Ar–*H*), 7.93 (s, 1H, CON*H*_*2*_*-H*_*A*_), 7.89 (d, *J* = 1.2 Hz, 1H, Ar–*H*), 7.75 (dd, *J*_*1*_ = 8.7 Hz, *J*_*2*_ = 1.2 Hz, 1H, Ar–*H*), 7.32 (s, 1H, CON*H*_*2*_*-H*_*B*_), 3.60–3.46 (m, 4H, 2 × C*H*_*2*_), 2.87–2.78 (m, 4H, 2 × C*H*_*2*_), 2.24 (s, 3H, pyrrole-C*H*_*3*_), 1.44 (s, 9H, C(C*H*_*3*_)_*3*_); LC-MS (ESI+) C_22_H_27_Cl_2_N_5_O_4_ *m*/*z*: 495.8 [M+H]^+^

#### 1-Boc-4-((5-carbamoyl-2-(3,4-dichloro-5-methyl-1*H*-pyrrole-2-carboxamido)phenyl)amino)piperidine (22d).

The synthesis was performed according to the general procedure D using compound **21d** (0.250 g, 1.01 mmol) as starting material. The residue was purified by column chromatography using DCM:MeOH = 15:1 as the mobile phase. The solvent of the collected fractions was evaporated, and the residue was additionally washed with MeOH to yield pure product. Yield: 0.115 g (30 %); off-white solid; R_f_ (DCM:MeOH = 15:1) = 0.25; ^1^H NMR (400 MHz, CDCl_3_): *δ* 9.40 (s, 1H, pyrrole-N*H*), 8.57 (s, 1H, pyrrole-CON*H*-Ar), 7.84 (d, *J* = 8.3 Hz, 1H, Ar–*H*), 7.46 (d, *J* = 1.8 Hz, 1H, Ar–*H*), 7.19 (dd, *J*_*1*_ = 8.3 Hz, *J*_*2*_ = 1.8 Hz, 1H, Ar–*H*), 6.05 (s, 1H, CON*H*_*2*_*-H*_*A*_), 5.56 (s, 1H, CON*H*_*2*_*-H*_*A*_), 4.07–3.92 (m, 2H, C*H*_*2*_), 3.57–3.46 (m, 2H, C*H* and Ar-N*H*), 3.00–2.88 (m, 2H, C*H*_*2*_), 2.05–1.96 (m, 2H, C*H*_*2*_), 1.46 (s, 9H, C(C*H*_*3*_)_*3*_), 1.43–1.35 (m, 2H, C*H*_*2*_); MS (ESI −) C_23_H_29_Cl_2_N_5_O_4_ *m*/*z* = 507.9 [M − H]^−^.

#### 2-Boc-7-(5-carbamoyl-2-(3,4-dichloro-5-methyl-1*H*-pyrrole-2-carboxamido)phenyl)-2,7-diazaspiro[4.4]nonane (22e).

The synthesis was performed according to the general procedure D using compound **21e** (0.400 g, 1.11 mmol) as the starting material. The residue was purified by column chromatography using DCM:MeOH = 20:1 as the mobile phase. The solvent of the collected fractions was evaporated, and the residue was additionally washed with EtOAc:hexane = 1:1 to yield a pure product. Yield: 0.340 g (53 %); white solid; R_f_ (DCM: MeOH = 20:1) = 0.16; ^1^H NMR (400 MHz, DMSO-*d*_6_): *δ* 12.41 (s, 1H, pyrrole-N*H*), 9.36 (s, 1H, pyrrole-CON*H*-Ar), 8.25–8.16 (m, 1H, Ar–*H*), 7.93 (s, 1H, CON*H*_*2*_*-H*_*A*_), 7.81–7.74 (m, 1H, Ar–*H*), 7.65–7.59 (m, 1H, Ar–*H*), 7.31 (s, 1H, CON*H*_*2*_*-H*_*B*_), 3.39–3.00 (m, 8H, 4 × C*H*_*2*_), 2.23 (s, 3H, pyrrole-C*H*_*3*_), 2.00–1.84 (m, 4H, 2 × C*H*_*2*_), 1.40 (s, 9H, C(C*H*_*3*_)_*3*_); ^13^C NMR (101 MHz, DMSO-*d*_6_): *δ* 167.8, 157.0, 154.1, 140.4, 135.4, 135.0, 130.3, 130.2, 130.0, 123.7, 123.4, 120.2, 119.6, 119.4, 119.3, 110.1, 110.1, 109.0, 78.7, 78.7, 61.2, 57.2, 56.6, 52.7, 52.5, 48.4, 47.5, 45.5, 45.2, 36.8, 35.7, 35.7, 35.5, 28.6, 11.2, rotamers are present in the spectra; MS (ESI −) C_25_H_31_Cl_2_N_5_O_4_ *m*/*z* = 534.4 [M − H]^−^.

#### *tert*-Butyl (1-(5-carbamoyl-2-(3,4-dichloro-5-methyl-1*H*-pyrrole-2-carboxamido)phenyl)piperidin-4-yl)carbamate (22f).

The synthesis was performed according to the general procedure D using compound **21f** (0.400 g, 1.20 mmol) as starting material. The residue was purified by column chromatography using DCM:MeOH = 20:1 as the mobile phase. The solvent of the collected fractions was evaporated, and the residue was additionally washed with MeOH to yield pure product. Yield: 0.290 g (48 %); white solid; R_f_ (DCM:MeOH = 20:1) = 0.19; ^1^H NMR (400 MHz, DMSO-*d*_6_): *δ* 12.39 (s, 1H, pyrrole-N*H*), 9.80 (s, 1H, pyrrole-CON*H*-Ar), 8.45 (d, *J* = 8.6 Hz, 1H, Ar–*H*), 7.93 (s, 1H, CON*H*_*2*_*-H*_*A*_), 7.90 (d, *J* = 1.6 Hz, 1H, Ar–*H*), 7.71 (dd, *J*_*1*_ = 8.6 Hz, *J*_*2*_ = 1.6 Hz, 1H, Ar–*H*), 7.31 (s, 1H, CON*H*_*2*_*-H*_*B*_), 7.11 (d, *J* = 7.8 Hz, 1H, CON*H*CH), 3.50–3.39 (m, 1H, C*H*), 2.95–2.87 (m, 2H, C*H*_*2*_), 2.82–2.73 (m, 2H, C*H*_*2*_), 2.24 (s, 3H, pyrrole-C*H*_*3*_), 1.91–1.81 (m, 2H, C*H*_*2*_), 1.69–1.57 (m, 2H, C*H*_*2*_), 1.41 (s, 9H, C(C*H*_*3*_)_*3*_); MS (ESI +) C_23_H_29_Cl_2_N_5_O_4_ *m*/*z* = 509.4 [M+H]^+^.

#### 1-Boc-4-((5-carbamoyl-2-(3,4-dichloro-5-methyl-1*H*-pyrrole-2-carboxamido)phenoxy)methyl)piperidine (22g).

The synthesis was performed according to the general procedure D using compound **21g** (0.333 g, 1.72 mmol) as starting material. The residue was taken up in DCM (40 mL) and extracted with 0.5 M HCl (2 × 50 mL), saturated NaHCO_3_ (20 mL) and brine (20 mL). The solvent of the organic layer was evaporated and the product was further purified by column chromatography using DCM:MeOH = 15:1 as the mobile phase. The solvent of the collected fractions was evaporated, and the residue was additionally washed with MeOH to yield pure product. Yield: 0.134 g (18 %); R_f_(DCM:MeOH = 15:1) = 0.18; ^1^H NMR (400 MHz, DMSO-*d*_6_): *δ* 12.45 (s, 1H, pyrrole-N*H*), 9.12 (s, 1H, pyrrole-CON*H*-Ar), 8.44 (d, *J* = 8.4 Hz, 1H, Ar–*H*), 7.95 (s, 1H, CON*H*_*2*_*-H*_*a*_), 7.59–7.49 (m, 2H, 2 × Ar–*H*), 7.34 (s, 1H, CON*H*_*2*_*-H*_*b*_), 4.07–3.99 (m, 4H, OC*H*_*2*_-piperidin and C*H*_*2*_), 2.87–2.69 (m, 2H, C*H*_*2*_), 2.23 (s, 3H, pyrrole-C*H*_*3*_) 2.11–1.87 (m, 1H, C*H*), 1.84–1.75 (m, 2H, C*H*_*2*_), 1.41 (s, 9H, C(C*H*_*3*_)_*3*_), 1.33–1.20 (m, 2H, C*H*_*2*_); MS (ESI −) C_24_H_30_Cl_2_N_4_O_5_ *m*/*z* = 523.2 [M − H]^−^.

#### 1-Boc-4-(5-carbamoyl-2-(3,4-dichloro-5-methyl-1*H*-pyrrole-2-carboxamido)benzoyl)piperazine (22h).

The synthesis was performed according to the general procedure D using compound **21h** (0.411 g, 1.18 mmol) as starting material. The residue was purified by column chromatography using DCM:MeOH = 12:1 as the mobile phase. The solvent of the collected fractions was evaporated, and the residue first co-evaporated with toluene and then additionally washed with MeOH to yield pure product. Yield: 0.113 g (18 %); brown solid; R_f_ (DCM:MeOH = 9:1) = 0.39; ^1^H NMR (400 MHz, DMSO-*d*_6_): *δ* 12.37 (s, 1H, pyrrole-N*H*), 9.42 (s, 1H, pyrrole-CON*H*-Ar), 8.21 (d, *J* = 8.7 Hz, 1H, Ar–*H*), 8.01 (s, 1H, CON*H*_*2*_*-H*_*A*_), 7.98 (dd, *J*_*1*_ = 8.7 Hz, *J*_*2*_ = 2.0 Hz, 1H, Ar–*H*), 7.86 (d, *J* = 2.0 Hz, 1H, Ar–*H*), 7.46 (s, 1H, CON*H*_*2*_*-H*_*B*_), 3.67–3.60 (m, 2H, C*H*_*2*_), 2.23 (s, 3H, pyrrole-C*H*_*3*_), 1.40 (s, 9H, C (C*H*_*3*_)_*3*_), 3 × C*H*_*2*_ overlapped with solvent; MS (ESI+) C_23_H_27_Cl_2_N_5_O_5_ *m*/*z*: 523.7 [M+H]^+^.

#### *N*-(4-Carbamoyl-2-((1-methyl-1*H*-indol-5-yl)oxy)phenyl)-3,4-dichloro-5-methyl-1*H*-pyrrole-2-carboxamide (22i).

The synthesis was performed according to the general procedure D using compound **21i** (0.180 g, 0.639 mmol) as starting material. The residue was taken up in DCM (150 mL) and extracted with 1 M HCl (100 mL). The solvent was evaporated and the residue was further purified by column chromatography using DCM:MeOH = 15:1 as the mobile phase. Yield: 0.050 g (17 %); white solid; R_f_ (DCM:MeOH = 15:1) = 0.12; ^1^H NMR (400 MHz, DMSO-*d*_6_): *δ* 12.48 (s, 1H, pyrrole-N*H*), 9.36 (s, 1H, pyrrole-CON*H*-Ar), 8.48 (d, *J* = 8.5 Hz, 1H, Ar–*H*), 7.91 (s, 1H, CON*H*_*2*_*-H*_*A*_), 7.68 (dd, *J*_*1*_ = 8.5 Hz, *J*_*2*_ = 1.5 Hz, 1H, Ar–*H*), 7.50 (d, *J* = 8.8 Hz, 1H, Ar–*H*), 7.43–7.19 (m, 4H, 3 × Ar–*H* and, CON*H*_*2*_*-H*_*B*_), 7.00 (dd, *J*_*1*_ = 8.8 Hz, *J*_*2*_ = 1.9 Hz, 1H, Ar–*H*), 6.41 (d, *J* = 2.6 Hz, 1H, Ar–*H*), 3.82 (s, 3H, indole-C*H*_*3*_), 2.23 (s, 3H, pyrrole-C*H*_*3*_); ^13^C NMR (101 MHz, DMSO-*d*_6_): *δ* 166.8, 156.5, 148.8, 146.8, 133.7, 131.6, 131.1, 129.8, 129.6, 128.5, 122.3, 119.0, 118.6, 116.0, 113.7, 111.1, 110.2, 109.6, 108.7, 100.4, 32.7, 10.8; HRMS calcd. for C_22_H_19_O_3_N_4_Cl_2_ [M+H]^+^ 457.08287, found 457.08259; HPLC: t_r_ = 4.45 min (96.3 % at 254 nm).

#### *N*-(4-Carbamoyl-2-(4-methoxyphenoxy)phenyl)-3,4-dichloro-5-methyl-1*H*-pyrrole-2-carboxamide (22j).

The synthesis was performed according to the general procedure D using compound **21j** (0.243 g, 1.25 mmol) as starting material. The residue was taken up in DCM (40 mL) and extracted with 1 M HCl (20 mL). The solvent was evaporated and the residue was further purified by column chromatography using DCM:MeOH = 15:1 as the mobile phase. The solvent of the collected fractions was evaporated, and the residue was additionally washed with MeOH to yield pure product. Yield: 0.072 g (16 %); off-white solid; R_f_ (DCM:MeOH = 15:1) = 0.30; ^1^H NMR (400 MHz, DMSO-*d*_6_): *δ* 12.48 (s, 1H, pyrrole-N*H*), 9.25 (s, 1H, pyrrole-CON*H*-Ar), 8.46 (d, *J* = 8.5 Hz, 1H, Ar–*H*), 7.94 (s, 1H, CON*H*_*2*_*-H*_*a*_), 7.70 (dd, *J*_*1*_ = 8.5 Hz, *J*_*2*_ = 1.9 Hz, 1H, Ar–*H*), 7.40 (d, *J* = 1.9 Hz, 1H, Ar–*H*), 7.35 (s, 1H, CON*H*_*2*_*-H*_*b*_), 7.10–7.05 (m, 2H, 2 × Ar–*H*), 7.02–6.97 (m, 2H, 2 × Ar–*H*), 3.76 (s, 3H, ArOC*H*_*3*_), 2.23 (s, 3H, pyrrole-C*H*_*3*_); ^13^C NMR (101 MHz, DMSO-*d*_6_): *δ* 167.1, 156.9, 156.4, 149.5, 146.4, 132.2, 130.3, 130.2, 123.3, 120.4, 119.8, 119.0, 116.7, 115.7, 110.7, 109.2, 55.9, 11.2; HRMS calcd. for C_20_H_17_Cl_2_N_3_O_4_ [M+H]^+^: 434.06689, found: 434.06690; HPLC: t_r_ = 6.57 min (98.8 % at 254 nm).

#### *N*-(4-Carbamoyl-2-((4-methoxybenzyl)oxy)phenyl)-3,4-dichloro-5-methyl-1*H*-pyrrole-2-carboxamide (22k).

The synthesis was performed according to the general procedure D using compound **21k** (0.230 g, 1.18 mmol) as starting material. The product was purified by column chromatography using DCM:MeOH = 15:1 as the mobile phase. The solvent of the collected fractions was evaporated, and the residue was additionally washed with MeOH to yield pure product. Yield: 0.040 g (11 %); off-white solid; R_f_ (DCM:MeOH = 15:1) = 0.23; ^1^H NMR (400 MHz, DMSO-*d*_6_) *δ* 12.39 (s, 1H, pyrrole-N*H*), 9.17 (s, 1H, pyrrole-CON*H-*Ar), 8.44 (d, *J* = 8.4 Hz, 1H, Ar–*H*), 7.97 (s, 1H, CON*H*_*2*_-*H*_*a*_), 7.75 (s, 1H, Ar–*H*), 7.66–7.28 (m, 4H, 3 × Ar–*H* and CON*H*_*2*_*-H*_*b*_), 7.03–6.97 (m, 2H, 2 × Ar–*H*), 5.17 (s, 2H, C*H*_*2*_), 3.79 (s, 3H, ArOC*H*_*3*_), 2.20 (s, 3H, pyrrole-C*H*_*3*_); ^13^C NMR (101 MHz, DMSO-*d*_6_): *δ* 167.7, 159.9, 156.7, 146.9, 131.1, 130.8, 130.2, 129.6, 128.4, 121.1, 119.0, 118.2, 114.3, 111.3, 110.4, 109.0, 70.9, 55.7, 11.2; HRMS calcd. for C_21_H_19_N_3_O_4_Cl_2_ [M − H]^−^ 434.06798 found 434.06690; HPLC: t_r_ = 6.47 min (96.0 % at 254 nm).

#### *N*-(4-Carbamoyl-2-(pyridin-4-ylmethoxy)phenyl)-3,4-dichloro-5-methyl-1*H*-pyrrole-2-carboxamide (22l).

The synthesis was performed according to the general procedure D using compound **21l** (0.230 g, 1.18 mmol) as starting material. The product was purified by column chromatography using DCM:MeOH:NH_4_OH = 15:1:0.1 as the mobile phase. The solvent of the collected fractions was evaporated, and the residue was additionally washed with MeOH to yield pure product. Yield: 0.020 g (2 %); white solid; R_f_ (DCM:MeOH:NH_4_OH = 9:1:0.1) = 0.25; off-white solid; ^1^H NMR (400 MHz, DMSO-*d*_6_): *δ* 12.42 (s, 1H, pyrrole-N*H*), 9.17 (s, 1H, pyrrole-CON*H*-Ar), 8.79–8.55 (m, 2H, 2 × Ar–*H*), 8.44 (d, *J* = 8.5 Hz, 1H, Ar–*H*), 7.95 (s, 1H, CON*H*_*2*_-*H*_*a*_), 7.71 (d, *J* = 1.8 Hz, 1H, Ar–*H*), 7.59 (dd, *J*_*1*_ = 8.4 Hz, *J*_*2*_ = 1.8 Hz, 1H, Ar–*H*), 7.56–7.53 (m, 2H, 2 × Ar–*H*), 7.37 (s, 1H, CON*H*_*2*_-*H*_*b*_), 5.33 (s, 2H, C*H*_*2*_), 2,21 (s, 3H, pyrrole-C*H*_*3*_); ^13^C NMR (101 MHz, DMSO-*d*_6_): *δ* 167.5, 156.8, 150.3, 146.7, 145.3, 130.7, 130.2, 129.8, 123.4, 121.4, 119.1, 118.7, 111.5, 110.4, 109.0, 69.4, 11.2; HRMS calcd. for C_19_H_16_Cl_2_N_4_O_3_ [M − H]^−^: 417.05267, found: 417.05327; HPLC: t_r_ = 4.23 min (99.2 % at 254 nm).

#### General procedure I.

To the corresponding Boc-protected amine (1 equiv.) DCM (5 mL/0.1 mmol) was added. To the reaction mixture TFA (20 equiv.) was added and the reaction mixture was stirred at room temperature overnight.

#### *N*-(2-(3-(Aminomethyl)phenoxy)-4-carbamoylphenyl)-3,4-dichloro-5-methyl-1*H*-pyrrole-2-carboxamide×CF_3_COOH (23a).

The synthesis was performed according to the general procedure I using compound **22a** (0.130 g, 0.244 mmol) as starting material. The solvent was evaporated to yield pure product. Yield: 0.123 g (92 %); white solid; ^1^H NMR (400 MHz, DMSO-*d*_6_): *δ* 12.50 (s, 1H, pyrrole-N*H*), 9.20 (s, 1H, pyrrole-CON*H*-Ar), 8.52 (d, *J* = 8.6 Hz, 1H, Ar–*H*), 8.14 (s, 3H, N*H*_*3*_^+^), 7.97 (s, 1H, CON*H*_*2*_*-H*_*A*_), 7.79 (dd, *J*_*1*_ = 8.6 Hz, *J*_*2*_ = 1.9 Hz, 1H, Ar–*H*), 7.56–7.45 (m, 2H, 2 × Ar–*H*), 7.40 (s, 1H, CON*H*_*2*_*-H*_*B*_), 7.27 (d, *J* = 7.9 Hz, 1H, Ar–*H*), 7.21–7.12 (m, 2H, 2 × Ar–*H*), 4.05 (q, *J* = 5.7 Hz, 2H, Ar-C*H*_*2*_-NH_3_^+^), 2.22 (s, 3H, pyrrole-C*H*_*3*_); MS (ESI+) C_20_H_18_Cl_2_N_4_O_3_ *m*/*z*: 432.6 [M+H]^+^.

#### *N*-(2-(4-(Aminomethyl)phenoxy)-4-carbamoylphenyl)-3,4-dichloro-5-methyl-1*H*-pyrrole-2-carboxamide×HCl (23b).

Crude product **22b** (0.040 g, 0.075 mmol) was dissolved in 1,4-dioxane (3 mL) and 4 M HCl in 1,4-dioxane (374 μL, 20 eq.) was added. The reaction mixture was stirred at 40 °C overnight. The solvent was evaporated to yield pure product. Yield: 0.035 g (100 %); light brown solid; ^1^H NMR (400 MHz, DMSO-*d*_6_): *δ* 12.49 (s, 1H, pyrrole-N*H*), 9.21 (s, 1H, pyrrole-CON*H*-Ar), 8.50 (d, *J* = 8.6 Hz, 1H, Ar–*H*), 8.11 (s, 3H, N*H*_*3*_^+^), 7.99 (s, 1H, CON*H*_*2*_*-H*_*A*_), 7.79 (dd, *J*_*1*_ = 8.6 Hz, *J*_*2*_ = 1.8 Hz, 1H, Ar–*H*), 7.54–7.49 (m, 3H, 3 × Ar–*H*), 7.40 (s, 1H, CON*H*_*2*_*-H*_*B*_), 7.19–7.14 (m, 2H, 2 × Ar–*H*), 4.04 (q, *J* = 5.4 Hz, 2H, Ar-C*H*_*2*_-NH^+^_3_), 2.22 (s, 3H, pyrrole-C*H*_*3*_); MS (ESI −) C_20_H_18_Cl_2_N_4_O_3_ *m*/*z* = 430.5 [M − H]^−^.

#### *N*-(4-Carbamoyl-2-(piperazin-1-yl)phenyl)-3,4-dichloro-5-methyl-1*H*-pyrrole-2-carboxamide×CF_3_COOH (23c).

The synthesis was performed according to the general procedure I using compound **22c** (0.120 g, 0.242 mmol) as starting material. The solvent was evaporated to yield pure product. Yield: 0.122 g (99 %); off-white solid; ^1^H NMR (400 MHz, DMSO-*d*_6_): *δ* 12.46 (s, 1H, pyrrole-N*H*), 9.57 (s, 1H, pyrrole-CON*H*-Ar), 8.66 (s, 2H, N*H*_*2*_^+^), 8.43 (d, *J* = 8.5 Hz, 1H, Ar–*H*), 8.01 (s, 1H, CON*H*_*2*_*-H*_*A*_), 7.84–7.75 (m, 2H, 2 × Ar–*H*), 7.38 (s, 1H, CON*H*_*2*_*-H*_*B*_), 3.34–3.22 (m, 4H, 2 × C*H*_*2*_), 3.10–2.99 (m, 4H, 2 × C*H*_*2*_), 2.24 (s, 3H, pyrrole-C*H*_*3*_); MS (ESI+) C_17_H_19_Cl_2_N_5_O_2_ *m*/*z*: 395.6 [M+H]^+^.

#### *N*-(4-Carbamoyl-2-(piperidin-4-ylamino)phenyl)-3,4-dichloro-5-methyl-1*H*-pyrrole-2-carboxamide×CF_3_COOH (23d).

The synthesis was performed according to the general procedure I using compound **22d** (0.081 g, 0.159 mmol) as starting material. The solvent was evaporated to yield pure product. Yield: 0.083 g (100 %); light yellow solid; ^1^H NMR (400 MHz, DMSO-*d*_6_): *δ* 12.31 (s, 1H, pyrrole-N*H*), 8.90 (s, 1H, pyrrole-CON*H*-Ar), 8.50–8.41 (m, 1H, N*H*_*2*_^+^*-H*_*A*_), 8.33–8.22 (m, 1H, N*H*_*2*_^+^*-H*_*B*_), 7.87 (s, 1H, CON*H*_*2*_*-H*_*A*_), 7.77 (d, *J* = 8.2 Hz, 1H, Ar–*H*), 7.36–7.28 (m, 3H, CON*H*_*2*_*-H*_*B*_ and 2 × Ar–*H*), 5.15–5.06 (m, 1H, C*H* or N*H*), 3.68–3.60 (m, 1H, C*H* or N*H*), 3.35–3.26 (m, 2H, C*H*_*2*_), 3.10–2.98 (m, 2H, C*H*_*2*_), 2.24 (s, 3H, pyrrole-C*H*_*3*_), 2.11–2.02 (m, 2H, C*H*_*2*_), 1.66–1.55 (m, 2H, C*H*_*2*_); ^19^F NMR (376 MHz, DMSO-*d*_6_) *δ* − 74.43 (s); MS (ESI+) C_18_H_21_Cl_2_N_5_O_2_ *m*/*z*: 409.8 [M+H]^+^.

#### *N*-(4-Carbamoyl-2-(2,7-diazaspiro[4.4]nonan-2-yl)phenyl)-3,4-dichloro-5-methyl-1*H*-pyrrole-2-carboxamide×CF_3_COOH (23e).

The synthesis was performed according to the general procedure I using compound **22e** (0.200 g, 0.373 mmol) as starting material. The solvent was evaporated and the residue was additionally washed with Et_2_O to yield pure product. Yield: 0.203 g (99 %); light brown solid; ^1^H NMR (400 MHz, DMSO-*d*_6_): *δ* 12.43 (s, 1H, pyrrole-N*H*), 9.33 (s, 1H, pyrrole-CON*H*-Ar), 8.93–8.75 (m, 2H), 8.24 (d, *J* = 8.5 Hz, 1H, Ar–*H*), 7.94 (s, 1H, CON*H*_*2*_*-H*_*A*_), 7.79 (d, *J* = 1.9 Hz, 1H, Ar–*H*), 7.65 (dd, *J*_*1*_ = 8.5 Hz, *J*_*2*_ = 1.9 Hz, 1H, Ar–*H*), 7.34 (s, 1H, CON*H*_*2*_*-H*_*B*_), 3.35–3.08 (m, 8H, 4 × C*H*_*2*_), 2.24 (s, 3H, pyrrole-C*H*_*3*_), 2.13–1.93 (m, 4H, 2 × C*H*_*2*_); ^13^C NMR (101 MHz, DMSO-*d*_6_) *δ* 167.7, 158.8 (q, *J* = 36.1 Hz), 157.0, 140.2, 135.2, 130.3, 130.0, 123.6, 120.5, 119.7, 119.3, 116.2 (q, *J* = 291.9 Hz), 110.2, 109.0, 60.8, 54.6, 52.4, 48.3, 44.9, 36.0, 35.6, 11.2; ^19^F NMR (376 MHz, DMSO) *δ* − 74.73; MS (ESI+) C_20_H_23_Cl_2_N_5_O_2_ *m*/*z*: 436.7 [M+H]^+^.

#### *N*-(2-(4-Aminopiperidin-1-yl)-4-carbamoylphenyl)-3,4-dichloro-5-methyl-1*H*-pyrrole-2-carboxamide×CF_3_COOH (23f).

The synthesis was performed according to the general procedure I using compound **22f** (0.200 g, 0.392 mmol) as starting material. The solvent was evaporated and the residue was additionally washed with Et_2_O to yield pure product. Yield: 0.205 g (100 %); light brown solid; ^1^H NMR (400 MHz, DMSO-*d*_6_) *δ* 12.43 (s, 1H, pyrrole-N*H*), 9.73 (s, 1H, pyrrole-CON*H*-Ar), 8.48 (d, *J* = 8.6 Hz, 1H, Ar–*H*), 8.06–7.87 (m, 5H, N*H*_*3*_^+^ and CON*H*_*2*_*-H*_*A*_ and Ar–*H*), 7.74 (dd, *J*_*1*_ = 8.6 Hz, *J*_*2*_ = 1.9 Hz, 1H, Ar–*H*), 7.33 (s, 1H, CON*H*_*2*_*-H*_*B*_), 3.28–3.16 (m, 1H, C*H*), 3.02–2.95 (m, 2H, C*H*_*2*_), 2.89–2.80 (m, 2H, C*H*_*2*_), 2.25 (s, 3H, pyrrole-C*H*_*3*_), 2.07–1.98 (m, 2H, C*H*_*2*_), 1.83–1.69 (m, 2H, C*H*_*2*_); MS (ESI+) C_18_H_21_Cl_2_N_5_O_2_ *m*/*z*: 410.6 [M+H]^+^.

#### *N*-(4-Carbamoyl-2-(piperidin-4-ylmethoxy)phenyl)-3,4-dichloro-5-methyl-1*H*-pyrrole-2-carboxamide×CF_3_COOH (23g).

The synthesis was performed according to the general procedure I using compound **22g** (0.114 g, 0.217 mmol) as starting material. A precipitate formed in the reaction mixture which was filtered off to yield pure product. Yield: 0.101 g (86 %); white solid; Rf (DKM: methanol:NH_4_OH = 6:1:0.1) = 0.11; 1H NMR (400 MHz, DMSO-*d*_6_): *δ* 12.47 (s, 1H, pyrrole-N*H*), 9.14 (s, 1H, pyrrole-CON*H*-Ar), 8.61–8.51 (m, 1H, N*H*_*2*_^+^*-H*_*a*_), 8.43 (d, *J* = 8.4 Hz, 1H, Ar–*H*), 8.28–8.20 (m, 1H, N*H*_*2*_^+^*-H*_*b*_), 7.94 (s, 1H, CON*H*_*2*_*-H*_*a*_), 7.62–7.53 (m, 2H, 2 × Ar–*H*), 7.36 (s, 1H, CON*H*_*2*_*-H*_*b*_), 4.07 (d, *J* = 6.7 Hz, 2H, OC*H*_*2*_-piperidin), 3.43–3.21 (m, 2H, C*H*_*2*_), 3.06–2.88 (m, 2H, C*H*_*2*_), 2.24 (s, 3H, pyrrole-C*H*_*3*_), 2.23–2.12 (m, 1H, C*H)* 2.04–1.95 (m, 2H, C*H*_*2*_), 1.52–1.38 (m 2H, C*H*_*2*_); MS (ESI +) C_19_H_22_Cl_2_N_4_O_3_ *m*/*z* = 424.7 [M+H]^+^.

#### *N*-(4-Carbamoyl-2-(piperazine-1-carbonyl)phenyl)-3,4-dichloro-5-methyl-1*H*-pyrrole-2-carboxamide×CF_3_COOH (23h).

The synthesis was performed according to the general procedure I using compound **22h** (0.100 g, 0.191 mmol) as starting material. The precipitate that formed in the reaction mixture was filtered off and the product was used in the next step without further purification. Yield: 0.060 g (59 %); off-white solid; ^1^H NMR (400 MHz, DMSO-*d*_6_): *δ* 12.34 (s, 1H, pyrrole-N*H*), 9.55 (s, 1H, pyrrole-CON*H*-Ar), 8.84 (s, 2H, N*H*_*2*_^+^), 8.07 (d, *J* = 8.6 Hz, 1H, Ar–*H*), 8.02 (s, 1H, CON*H*_*2*_*-H*_*A*_), 7.99 (dd, *J*_*1*_ = 8.6 Hz, *J*_*2*_ = 2.0 Hz, 1H, Ar–*H*), 7.91 (d, *J* = 2.0 Hz, 1H, Ar–*H*), 7.49 (s, 1H, CON*H*_*2*_*-H*_*B*_), 3.86–3.77 (m, 2H, C*H*_*2*_), 3.67–3.53 (m, 2H, C*H*_*2*_), 3.21–3.12 (m, 4H, 2 × C*H*_*2*_), 2.24 (s, 3H, pyrrole-C*H*_*3*_); MS (ESI+) C_18_H_19_Cl_2_N_5_O_3_ *m*/*z*: 423.6 [M+H]^+^.

#### *N*-(4-Carbamoyl-2-(3-((dimethylamino)methyl)phenoxy)phenyl)-3,4-dichloro-5-methyl-1*H*-pyrrole-2-carboxamide (24a).

The synthesis was performed according to the general procedure E and compound **23c** (0.055 g, 0.102 mmol) was used as starting material. The product was purified by column chromatography using DCM/MeOH/NH_4_OH = 9:1:0.1 as the mobile phase. Yield: 0.021 g (43 %); white solid; R_f_(DCM:MeOH:NH_4_OH = 9:1:0.1) = 0.23; ^1^H NMR (400 MHz, DMSO-*d*_6_): *δ* 12.46 (s, 1H, pyrrole-N*H*), 9.15 (s, 1H, pyrrole-CON*H*-Ar), 8.49 (d, *J* = 8.6 Hz, 1H, Ar–*H*), 7.96 (s, 1H, CON*H*_*2*_*-H*_*A*_), 7.78 (dd, *J*_*1*_ = 8.6 Hz, *J*_*2*_ = 1.5 Hz, 1H, Ar–*H*), 7.57 (d, *J* = 1.5 Hz, 1H, Ar–*H*), 7.41–7.31 (m, 2H, Ar–*H* and CON*H*_*2*_*-H*_*B*_), 7.08 (d, *J* = 7.6 Hz, 1H, Ar–*H*), 7.01–6.90 (m, 2H, 2 × Ar–*H*), 3.38 (s, 2H, Ar-C*H*_*2*_-N(CH_3_)_2_), 2.21 (s, 3H, pyrrole-C*H*_*3*_), 2.11 (s, 6H, N(C*H*_*3*_)_*2*_), ^13^C NMR (101 MHz, DMSO-*d*_6_): *δ* 166.5, 156.5, 156.3, 144.3, 141.6, 132.7, 129.9, 124.1, 124.0, 119.7, 118.5, 118.4, 117.3, 115.9, 110.3, 108.7, 62.9, 44.9, 10.8; HRMS calcd. for C_22_H_23_O_3_N_4_Cl_2_ [M+H]^+^ 461.11417, found 461.11360; HPLC: t_r_ = 5.14 min (95.3 % at 254 nm).

#### *N*-(4-Carbamoyl-2-(4-((dimethylamino)methyl)phenoxy)phenyl)-3,4-dichloro-5-methyl-1*H*-pyrrole-2-carboxamide (24b).

The synthesis was performed according to general procedure E and compound **23c** (0.055 g, 0.102 mmol) was used as starting material. The product was purified by column chromatography using DCM/MeOH/NH_4_OH = 9:1:0.1 as the mobile phase. Yield: 0.019 g (66 %); white solid; R_f_(DCM:MeOH:NH_4_OH = 9:1:0.1) = 0.16; ^1^H NMR (400 MHz, DMSO-*d*_6_): *δ* 12.46 (s, 1H, pyrrole-N*H*), 9.16 (s, 1H, pyrrole-CON*H*-Ar), 8.49 (d, *J* = 8.6 Hz, 1H, Ar–*H*), 7.96 (s, 1H, CON*H*_*2*_*-H*_*A*_), 7.77 (dd, *J*_*1*_ = 8.6 Hz, *J*_*2*_ = 1.9 Hz, 1H, Ar–*H*), 7.56 (d, *J* = 1.9 Hz, 1H, Ar–*H*), 7.37 (s, 1H, Ar–*H,* CON*H*_*2*_*-H*_*B*_), 7.34–7.28 (m, 2H, 2 × Ar–*H*), 7.04–6.97 (m, 2SH, 2 × Ar–*H*), 3.38 (s, 2H, Ar-C*H*_*2*_-N(CH_3_)_2_), 2.21 (s, 3H, pyrroleC*H*_*3*_), 2.14 (s, 6H, N(C*H*_*3*_)_*2*_); ^13^C NMR (101 MHz, DMSO-*d*_6_) *δ* 166.5, 156.5, 155.1, 144.4, 134.4, 132.6, 130.5, 129.9, 123.9, 119.7, 118.5, 118.1, 117.2, 110.3, 108.7, 62.6, 44.9, 10.8; HRMS calcd. for C_22_H_23_O_3_N_4_Cl_2_ [M+H]^+^ 461.11417, found 461.11346; HPLC: t_r_ = 4.62 min (97.8 % at 254 nm).

#### *N*-(4-Carbamoyl-2-(4-methylpiperazin-1-yl)phenyl)-3,4-dichloro-5-methyl-1*H*-pyrrole-2-carboxamide (24c).

The synthesis was performed according to the general procedure E using compound **23c** (0.110 g, 0.216 mmol) as starting material. The crude was washed with EtOAc, hexane and methanol to yield pure product. Yield: 0.057 g (65 %); white solid; ^1^H NMR (400 MHz, DMSO-*d*_6_) *δ* 12.42 (s, 1H, pyrrole-N*H*), 9.77 (s, 1H, pyrrole-CON*H*-Ar), 8.43 (d, *J* = 8.6 Hz, 1H, Ar–*H*), 7.95 (s, 1H, CON*H*_*2*_*-H*_*A*_), 7.88 (d, *J* = 1.8 Hz, 1H, Ar–*H*), 7.73 (dd, *J*_*1*_ = 8.6 Hz, *J*_*2*_ = 1.8 Hz, 1H, Ar–*H*), 7.32 (s, 1H, CON*H*_*2*_*-H*_*B*_), 2.87 (t, *J* = 4.3 Hz, 4H, 2 × C*H*_*2*_), 2.60–2.52 (m, 4H, 2 × C*H*_*2*_), 2.26 (s, 3H, piperazine-C*H*_*3*_), 2.24 (s, 3H, pyrrole-C*H*_*3*_); ^13^C NMR (101 MHz, DMSO-*d*_6_) *δ* 167.1, 156.6, 141.3, 136.5, 129.8, 129.2, 125.0, 121.1, 118.9, 118.3, 109.6, 108.5, 55.1, 52.1, 46.0, 10.8; HRMS (ESI+) calcd. for C_18_H_22_Cl_2_N_5_O_2_ [M+H]^+^: 410.11451, found: 410.11390; HPLC: t_r_ = 3.08 min (96.5 % at 254 nm).

#### *N*-(4-Carbamoyl-2-((1-methylpiperidin-4-yl)amino)phenyl)-3,4-dichloro-5-methyl-1*H*-pyrrole-2-carboxamide (24d).

The synthesis was performed according to the general procedure E using compound **23d** (0.070 g, 0.134 mmol) as starting material. The product was purified by column chromatography using DCM/MeOH/NH_4_OH = 9:1:0.1 as the mobile phase. Yield: 0.044 g (77 %); white solid; R_f_(DCM:MeOH: NH_4_OH = 9:1:0.1) = 0.05; ^1^H NMR (400 MHz, DMSO-*d*_6_) *δ* 12.26 (s, 1H, pyrrole-N*H*), 8.88 (s, 1H, pyrrole-CON*H*-Ar), 7.89 (s, 1H, CON*H*_*2*_*-H*_*A*_), 7.65 (d, *J* = 8.2 Hz, 1H, Ar–*H*), 7.32–7.21 (m, 3H, 2 × Ar–*H* and CON*H*_*2*_-*H*_*B*_), 4.90 (d, *J* = 6.6 Hz, 1H, N*H*), 2.77–2.69 (m, 2H, C*H*_*2*_), 2.23 (s, 3H, pyrrole-C*H*_*3*_), 2.19 (s, 3H, N–C*H*_*3*_), 2.13–2.04 (m, 2H, C*H*_*2*_), 1.94–1.84 (m, 2H, C*H*_*2*_), 1.52–1.40 (m, 2H, C*H*_*2*_), C*H* is overlapped with water – not visible; ^13^C NMR (101 MHz, DMSO-*d*_6_) *δ* 167.9, 157.3, 139.3, 131.1, 128.4, 128.4, 122.9, 119.6, 116.9, 113.0, 110.5, 108.4, 53.9, 48.8, 46.0, 31.5, 10.8; HRMS (ESI+) calcd. for C_19_H_24_Cl_2_N_5_O_2_ [M+H]^+^: 424.13016, found: 424.12945; HPLC: t_r_ = 4.08 min (99.6 % at 254 nm).

#### *N*-(4-Carbamoyl-2-(7-methyl-2,7-diazaspiro[4.4]nonan-2-yl)phenyl)-3,4-dichloro-5-methyl-1*H*-pyrrole-2-carboxamide (24e).

The synthesis was performed according to the general procedure E using compound **23e** (0.100 g, 0.182 mmol) as starting material. The product was purified by column chromatography using DCM/MeOH/NH_4_OH = 10:1:0.1 as the mobile phase. Yield: 0.070 g (85 %); white solid; R_f_(DCM: MeOH:NH_4_OH = 10:1:0.1) = 0.12; ^1^H NMR (400 MHz, DMSO-*d*_6_): *δ* 12.39 (s, 1H, pyrrole-N*H*), 9.28 (s, 1H, pyrrole-CON*H*-Ar), 8.15 (d, *J* = 8.4 Hz, 1H, Ar–*H*), 7.93 (s, 1H, CON*H*_*2*_*-H*_*A*_), 7.72 (d, *J* = 1.8 Hz, 1H, Ar–*H*), 7.58 (dd, *J*_*1*_ = 8.5 Hz, *J*_*2*_ = 1.8 Hz, 1H, Ar–*H*), 7.30 (s, 1H, CON*H*_*2*_*-H*_*A*_), 3.18–3.05 (m, 4H, 2 × C*H*_*2*_), 2.94–2.59 (m, 4H, 2 × C*H*_*2*_), 2.38 (s, 3H, NC*H*_*3*_), 2.23 (s, 3H, pyrrole-C*H*_*3*_), 2.05–1.83 (m, 4H, 2 × C*H*_*2*_); ^13^C NMR (101 MHz, DMSO-*d*_6_): *δ* 167.4, 156.6, 140.6, 134.1, 130.0, 129.5, 122.5, 120.5, 118.9, 118.6, 109.7, 108.5, 67.1, 63.3, 55.3, 51.9, 48.0, 41.8, 38.1, 37.6, 10.8; HRMS (ESI+) calcd. for C_21_H_26_Cl_2_N_5_O_2_ [M+H]^+^: 450.14636, found: 450.14409; HPLC: t_r_ = 4.48 min (98.0 % at 254 nm).

#### *N*-(4-Carbamoyl-2-(4-(dimethylamino)piperidin-1-yl)phenyl)-3,4-dichloro-5-methyl-1*H*-pyrrole-2-carboxamide (24f).

The synthesis was performed according to the general procedure E using compound **23f** (0.100 g, 0.190 mmol) as starting material. The product was purified by column chromatography using DCM/MeOH/NH_4_OH = 10:1:0.1 as the mobile phase. Yield: 0.061 g (74 %): white solid; R_f_(DCM: MeOH:NH_4_OH = 10:1:0.1) = 0.09; ^1^H NMR (400 MHz, DMSO-*d*_6_): *δ* 12.43 (s, 1H, pyrrole-N*H*), 9.78 (s, 1H, pyrrole-CON*H*-Ar), 8.42 (d, *J* = 8.6 Hz, 1H, Ar–*H*), 7.93 (s, 1H, CON*H*_*2*_*-H*_*A*_), 7.86 (d, *J* = 1.9 Hz, 1H, Ar–*H*), 7.72 (dd, *J*_*1*_ = 8.6 Hz, *J*_*2*_ = 1.9 Hz, 1H, Ar–*H*), 7.31 (s, 1H, CON*H*_*2*_*-H*_*B*_), 3.02–2.93 (m, 2H, C*H*_*2*_), 2.82–2.70 (m, 2H, C*H*_*2*_), 2.45–2.27 (m, 6H, N(C*H*_*3*_*)*_*2*_), 2.24 (s, 3H, pyrrole-C*H*_*3*_), 2.01–1.87 (m, 2H, C*H*_*2*_), 1.73–1.58 (m, 2H, C*H*_*2*_), the signal for the remaining C*H* is overlapped with solvent; ^13^C NMR (101 MHz, DMSO-*d*_6_): *δ* 167.6, 157.0, 142.1, 136.8, 130.3, 129.7, 125.3, 121.5, 119.3, 118.7, 110.0, 109.0, 61.5, 52.4, 41.5, 28.7, 11.2; HRMS (ESI+) calcd. for C_20_H_26_Cl_2_N_5_O_2_ [M+H]^+^: 438.14581, found: 438.14417; HPLC: t_r_ = 3.66 min (98.7 % at 254 nm).

#### *N*-(4-Carbamoyl-2-((1-methylpiperidin-4-yl)methoxy)phenyl)-3,4-dichloro-5-methyl-1*H*-pyrrole-2-carboxamide (24g).

The synthesis was performed according to the general procedure E using compound **23g** (0.055 g, 0.102 mmol) as starting material. The product was purified by column chromatography using DCM/MeOH/NH_4_OH = 9:1:0.1 as the mobile phase. Yield: 0.011 g (39 %); white solid; R_f_(DCM: MeOH:NH_4_OH = 9:1:0.1) = 0.10; ^1^H NMR (400 MHz, DMSO-*d*_6_): *δ* 12.44 (s, 1H, pyrrole-N*H*), 9.16 (s, 1H, pyrrole-CON*H*-Ar), 8.44 (d, *J* = 8.4 Hz, 1H, Ar–*H*), 7.95 (s, 1H, CON*H*_*2*_*-H*_*a*_), 7.62–7.50 (m, 2Hz, 2 × Ar–*H*), 7.33 (s, 1H, CON*H*_*2*_*-H*_*b*_), 4.02 (d, *J* = 5.8 Hz, 2H, OC*H*_*2*_-piperidine), 2.90–2.78 (m, 2H, C*H*_*2*_), 2.26–2.20 (m, 6H, 2 × C*H*_*3*_), 2.00–1.92 (m, 2H, C*H*_*2*_), 1.88–1.71 (m, 3H, C*H*_*2*_ and C*H*), 1.45–1.28 (m, 2H, C*H*_*2*_); ^13^C NMR (101 MHz, DMSO-*d*_6_): δ167.6, 156.8, 147.1, 130.3, 130.3, 129.7, 120.9, 119.1, 118.4, 110.8, 110.1, 109.1, 73.5, 55.2, 46.4, 35.5, 28.9, 11.3; HRMS (ESI+) calcd. for C_20_H_24_Cl_2_N_4_O_3_ [M+H]^+^: 439.12982, found: 439,12927; HPLC: t_r_ = 4.04 min (97.2 % at 254 nm).

#### *N*-(4-Carbamoyl-2-(4-methylpiperazine-1-carbonyl)phenyl)-3,4-dichloro-5-methyl-1*H*-pyrrole-2-carboxamide (24h).

The synthesis was performed according to the general procedure E using compound **23h** (0.060 g, 0.112 mmol) as starting material. The product was purified by column chromatography using DCM/MeOH/NH_4_OH = 9:1:0.1 as the mobile phase. Yield: 0.023 (47 %); light brown solid; R_f_(DCM:MeOH:NH_4_OH = 9:1:0.1) = 0.09; ^1^H NMR (400 MHz, DMSO-*d*_6_): *δ* 12.41 (s, 1H, pyrrole-N*H*), 9.35 (s, 1H, pyrrole-CON*H*-Ar), 8.27 (d, *J* = 8.7 Hz, 1H, Ar–*H*), 8.03 (s, 1H, CON*H*_*2*_*-H*_*A*_), 7.98 (dd, *J*_*1*_ = 8.7 Hz, *J*_*2*_ = 2.0 Hz, 1H, Ar–*H*), 7.84 (d, *J* = 2.0 Hz, 1H, Ar–*H*), 7.45 (s, 1H, CON*H*_*2*_*-H*_*B*_), 3.74–3.60 (m, 2H, C*H*_*2*_), 2.45–2.14 (m, 12H, pyrrole-C*H*_*3*_ and piperidine-C*H*_*3*_ and 3 × C*H*_*2*_); ^13^C NMR (101 MHz, DMSO-*d*_6_): *δ* 166.7, 166.6, 156.6, 137.8, 129.8, 129.3, 129.2, 126.6, 125.6, 121.8, 118.4, 110.6, 108.7, 54.7, 54.1, 48.6, 47.1, 45.5, 10.8; HRMS (ESI+) calcd. for C_19_H_22_Cl_2_N_5_O_3_ [M+H]^+^: 438.10942, found: 438.10884; HPLC: t_r_ = 2.75 min (99.2 % at 254 nm).

#### 1-Boc-4-(2-(methoxycarbonyl)-5-nitrophenoxy)piperidine (25).

The synthesis was performed according to the general procedure A using methyl 2-hydroxy-4-nitrobenzoate (1.86 g, 9.43 mmol) as starting material. The product was purified by column chromatography using EtOAc:hexane = 1:3 as the mobile phase. Yield: 1.84 g (51 %); yellow solidified oil; R_f_(EtOAc:hexane = 1:3) = 0.25; ^1^H NMR (400 MHz, CDCl_3_): *δ* 7.90 (d, *J* = 8.5 Hz, 1H, Ar–*H*), 7.83 (dd, *J*_*1*_ = 8.5 Hz, *J*_*2*_ = 2.0 Hz, 1H, Ar–*H*), 7.80 (d, *J* = 2.0 Hz, 1H, Ar–*H*), 4.79–4.71 (m, 1H, C*H*), 3.93 (s, 3H, COO–C*H*_*3*_), 3.58 (t, *J* = 5.5 Hz, 4H, 2 × C*H*_*2*_), 1.99–1.82 (m, 4H, 2 × C*H*_*2*_), 1.48 (s, 9H, C(C*H*_*3*_)_*3*_); MS (ESI+) C_18_H_24_N_2_O_7_ *m*/*z*: 365.9 [M-CH_3_+H]^+^.

#### 1-Boc-4-(2-carbamoyl-5-nitrophenoxy)piperidine (26).

The synthesis was performed according to the general procedure B using compound **25** (0.750 g, 1.97 mmol) as stating material. Yield: 0.698 g (97 %); off-white solid; ^1^H NMR (400 MHz, DMSO-*d*_6_): *δ* 7.93 (d, *J* = 2.0 Hz, 1H, Ar–*H*), 7.85 (dd, *J*_*1*_ = 8.4 Hz, *J*_*2*_ = 2.0 Hz, 1H, Ar–*H*), 7.79 (d, *J* = 8.4 Hz, 1H, Ar–*H*), 7.77 (s, 1H, CON*H*_*2*_*-H*_*A*_), 7.70 (s, 1H, CON*H*_*2*_*-H*_*A*_), 5.03–4.82 (m, 1H, C*H*), 3.63–3.52 (m, 2H, C*H*_*2*_), 3.32–3.25 (m, 2H, C*H*_*2*_), 1.97–1.86 (m, 2H, C*H*_*2*_), 1.72–1.59 (m, 2H, C*H*_*2*_), 1.41 (s, 9H, C (C*H*_*3*_)_*3*_); MS (ESI+) C_17_H_23_N_3_O_6_ *m*/*z*: 265.7 [M-Boc + H]^+^.

#### 1-Boc-4-(5-amino-2-carbamoylphenoxy)piperidine (27).

The synthesis was performed according to the general procedure C using compound **26** (0.650 g, 1.78 mmol) as the starting material. Yield: 0.562 g (94 %); brown solid; R_f_(EtOAc:hexane = 2:1) = 0.05; ^1^H NMR (400 MHz, CDCl_3_) *δ* 8.01 (d, *J* = 8.5 Hz, 1H, Ar–*H*), 7.56 (s, 1H, CON*H*_*2*_-*H*_*A*_), 6.35 (dd, *J*_*1*_ = 8.5 Hz, *J*_*2*_ = 2.1 Hz, 1H, Ar–*H*), 6.21 (d, *J* = 2.1 Hz, 1H, Ar–*H*), 5.61 (s, 1H, CON*H*_*2*_*-H*_*B*_), 4.65–4.51 (m, 1H, C*H*), 4.01 (s, 2H, Ar-N*H*_*2*_), 3.85–3.69 (m, 2H, C*H*_*2*_), 3.30–3.21 (m, 2H, C*H*_*2*_), 2.11–1.98 (m, 2H, C*H*_*2*_), 1.83–1.72 (m, 2H, C*H*_*2*_), 1.47 (s, 9H, C(C*H*_*3*_)_*3*_); MS (ESI+) C_17_H_25_N_3_O_4_ *m*/*z*: 336.1 [M+H]^+^.

#### 1-Boc-4-(2-carbamoyl-5-(3,4-dichloro-5-methyl-1*H*-pyrrole-2-carboxamido)phenoxy)piperidine (28).

The synthesis was performed according to the general procedure D using compound **27** (0.350 g, 1.04 mmol) as starting material. The product was purified by column chromatography using DCM:MeOH = 20:1 as the mobile phase. After the column the product was additionally washed with methanol to yield a pure product. Yield: 0.415 g (78 %); white solid; R_f_(DCM:MeOH = 20:1) = 0.17; ^1^H NMR (400 MHz, DMSO-*d*_6_): *δ* 12.24 (s, 1H, pyrrole-N*H*), 9.63 (s, 1H, pyrrole-CON*H*-Ar), 7.81 (d, *J* = 8.6 Hz, 1H, Ar–*H*), 7.62 (d, *J* = 1.7 Hz, 1H, Ar–*H*), 7.47 (s, 1H, CON*H*_*2*_*-H*_*A*_), 7.43 (s, 1H, CON*H*_*2*_*-H*_*B*_), 7.29 (dd, *J*_*1*_ = 8.6 Hz, *J*_*2*_ = 1.7 Hz, 1H, Ar–*H*), 4.70–4.60 (m, 1H, C*H*), 3.70–3.58 (m, 2H, C*H*_*2*_), 3.28–3.19 (m, 2H, C*H*_*2*_), 2.23 (s, 3H, pyrroleC*H*_*3*_), 2.05–1.94 (m, 2H, C*H*_*2*_), 1.76–1.63 (m, 2H, C*H*_*2*_), 1.41 (s, 9H, C (C*H*_*3*_)_*3*_); MS (ESI+) C_23_H_28_Cl_2_N_4_O_5_ *m*/*z*: 510.9 [M+H]^+^.

#### *N*-(4-Carbamoyl-3-(piperidin-4-yloxy)phenyl)-3,4-dichloro-5-methyl-1*H*-pyrrole-2-carboxamide×CF_3_COOH (29).

The synthesis was performed according to the general procedure I using compound **28** (0.185 g, 0.362 mmol) as starting material. The solvent was evaporated and the residue was washed with Et_2_O to yield a pure product. Yield: 0.187 g (98 %); off-white solid; ^1^H NMR (400 MHz, DMSO-*d*_6_): *δ* 12.24 (s, 1H, pyrrole-N*H*), 9.63 (s, 1H, pyrrole-CON*H*-Ar), 8.50 (s, 2H, N*H*_*2*_^+^), 7.72 (d, *J* = 8.5 Hz, 1H, Ar–*H*), 7.61 (d, *J* = 1.8 Hz, 1H, Ar–*H*), 7.51 (s, 1H, CON*H*_*2*_*-H*_*A*_), 7.42 (s, 1H, CON*H*_*2*_*-H*_*B*_), 7.28 (dd, *J*_*1*_ = 8.5 Hz, *J*_*2*_ = 1.8 Hz, 1H, Ar–*H*), 4.76–4.70 (m, 1H, C*H*), 3.32–3.21 (m, 2H, C*H*_*2*_), 3.20–3.07 (m, 2H, C*H*_*2*_), 2.24 (s, 3H, pyrrole-C*H*_*3*_), 2.17–2.08 (m, 2H, C*H*_*2*_), 2.02–1.90 (m, 2H, C*H*_*2*_); MS (ESI+) C_18_H_20_Cl_2_N_4_O_3_ *m*/*z*: 410.8 [M+H]^+^.

#### *N*-(4-Carbamoyl-3-((1-methylpiperidin-4-yl)oxy)phenyl)-3,4-dichloro-5-methyl-1*H*-pyrrole-2-carboxamide (30).

The synthesis was performed according to the general procedure E using compound **29** (0.100 g, 0.190 mmol) as starting material. The product was purified by column chromatography using DCM/MeOH/NH_4_OH = 10:1:0.1 as the mobile phase. Yield: 0.069 g (85 %); white solid; R_f_(DCM:MeOH: NH_4_OH = 9:1:0.1) = 0.06; ^1^H NMR (400 MHz, DMSO-*d*_6_): *δ* 12.25 (s, 1H, pyrrole-N*H*), 9.64 (s, 1H, pyrrole-CON*H*-Ar), 7.83 (d, *J* = 8.6 Hz, 1H, Ar–*H*), 7.63 (d, *J* = 1.8 Hz, 1H, Ar–*H*), 7.53 (s, 1H, CON*H*_*2*_*-H*_*A*_), 7.47 (s, 1H, CON*H*_*2*_*-H*_*B*_), 7.28 (dd, *J*_*1*_ = 8.6 Hz, *J*_*2*_ = 1.8 Hz, 1H, Ar–*H*), 4.57–4.48 (m, 1H, O–C*H*), 2.79–2.65 (m, 2H, C*H*_*2*_), 2.45–2.35 (m, 2H, C*H*_*2*_), 2.30 (s, 3H), 2.23 (s, 3H, pyrrole-C*H*_*3*_), 2.10–2.01 (m, 2H, C*H*_*2*_), 1.90–1.77 (m, 2H, C*H*_*2*_); ^13^C NMR (101 MHz, DMSO-*d*_6_): *δ* 165.8, 157.5, 155.5, 142.4, 131.8, 128.4, 119.4, 118.2, 111.9, 111.8, 108.7, 105.3, 72.9, 51.9, 45.2, 29.8, 10.8; HRMS (ESI+) calcd. for C_19_H_23_Cl_2_N_4_O_3_ [M+H]^+^: 425.11417, found: 425.11349; HPLC: t_r_ = 4.45 min (99.1 % at 254 nm).

#### General procedure J.

*N*-Boc-4-hydroxypiperidin (1 equiv.) was dissolved in DMF (3 mL/mmol) and NaH (1.5 equiv.) was added on an ice bath. The reaction mixture was stirred for 20 min and respective 4-substituted 2-fluoro-1-nitrobenzene (1 equiv.) was added and the reaction mixture was stirred at room temperature overnight. The solvent was evaporated under reduced pressure. The residue was taken up in ethyl acetate (40 mL) and washed with 10 % citric acid (2 × 30 mL), water (30 mL) and brine (30 mL). The organic layer was dried over Na_2_SO_4_, filtered and the solvent was evaporated under reduced pressure.

#### 1-Boc-4-(5-cyclopropyl-2-nitrophenoxy)piperidine (31a).

The synthesis was performed according to the general procedure J using 4-cyclopropyl-2-fluoro-1-nitrobenzene (0.405 g, 2.24 mmol) as starting material. Yield: 0.756 g (93 %); yellow oil; ^1^H NMR (400 MHz, CDCl_3_) *δ* 7.78 (d, *J* = 8.5 Hz, 1H, Ar–*H*), 6.76 (d, *J* = 1.6 Hz, 1H, Ar–*H*), 6.63 (dd, *J*_*1*_ = 8.5 Hz, *J*_*2*_ = 1.7 Hz, 1H, Ar–*H*), 4.71–4.64 (m, 1H, C*H*), 3.62–3.48 (m, 4H, 2 × C*H*_*2*_), 1.95–1.84 (m, 5H, C*H* and 2 × C*H*_*2*_), 1.47 (s, 9H, C (C*H*_*3*_)_*3*_), 1.13–1.06 (m, 2H, C*H*_*2*_), 0.80–0.73 (m, 2H, C*H*_*2*_); MS (ESI+) C_19_H_26_N_2_O_5_ *m*/*z*: 263.7 [M-Boc + H]^+^.

#### 1-Boc-4-(5-ethoxy-2-nitrophenoxy)piperidine (31b).

The synthesis was performed according to the general procedure J using 4-ethoxy-2-fluoro-1-nitrobenzene (0.460 g, 3.73 mmol) as starting material. Yield: 0.839 g (92 %); yellow oil; ^1^H NMR (400 MHz, CDCl_3_): *δ* 7.97 (d, *J* = 9.2 Hz, 1H, Ar–*H*), 6.54–6.48 (m, 2H, 2 × Ar–*H*), 4.64 (p, *J* = 4.5 Hz, 1H, C*H*), 4.09 (q, *J* = 6.9 Hz, 2H, C*H*_*2*_), 3.65–3.46 (m, 4H, 2 × C*H*_*2*_), 1.94–1.83 (m, 4H, 2 × C*H*_*2*_), 1.47 (s, 9H, C(C*H*_*3*_)_*3*_), 1.45 (t, *J* = 6.0 Hz, 3H, OCH_2_C*H*_*3*_); MS (ESI+) C_18_H_26_N_2_O_6_ *m*/*z*: 267.4 [M-Boc + H]^+^.

#### 1-Boc-4-(2-amino-5-propylphenoxy)piperidine (32a).

The synthesis was performed according to the general procedure C using compound **31a** (0.756 g, 2.09 mmol) as starting material. Yield: 0.670 g (96 %); off-white solid; ^1^H NMR (400 MHz, CDCl_3_): *δ* 6.68–6.57 (m, 3H, 3 × Ar–*H*), 4.48–4.40 (m, 1H, C*H*), 3.77–3.69 (m, 2H, C*H*_*2*_), 3.36–3.27 (m, 2H, C*H*_*2*_), 2.49–2.44 (m, 2H, C*H*_*2*_), 2.00–1.89 (m, 2H, C*H*_*2*_), 1.81–1.71 (m, 2H, C*H*_*2*_), 1.47 (s, 9H, C(C*H*_*3*_)_*3*_), 0.92 (t, *J* = 7.3 Hz, 3H, CH_2_C*H*_*3*_); MS (ESI+) C_19_H_30_N_2_O_3_ *m*/*z*: 335.7 [M+H]^+^.

#### 1-Boc-4-(2-amino-5-ethoxyphenoxy)piperidine (32b).

The synthesis was performed according to the general procedure C using compound **31b** (0.750 g, 2.05 mmol) as starting material. Yield: 0.688 g (100 %); off-white solid; ^1^H NMR (400 MHz, CDCl_3_): *δ* 6.66 (d, *J* = 8.5 Hz, 1H, Ar–*H*), 6.46 (d, *J* = 2.6 Hz, 1H, Ar–*H*), 6.37 (dd, *J*_*1*_ = 8.5 Hz, *J*_*2*_ = 2.6 Hz, 1H, Ar–*H*), 4.46–4.37 (m, 1H, C*H*), 3.95 (q, *J* = 7.0 Hz, 2H, C*H*_*2*_), 3.77–3.66 (m, 2H, C*H*_*2*_), 3.35–3.24 (m, 2H, C*H*_*2*_), 1.99–1.89 (m, 2H, C*H*_*2*_), 1.81–1.70 (m, 2H, C*H*_*2*_), 1.47 (s, 9H, C(C*H*_*3*_)_*3*_), 1.38 (t, *J* = 7.0 Hz, 3H, OCH_2_C*H*_*3*_); MS (ESI+) C_18_H_28_N_2_O_4_ *m*/*z*: 337.1 [M+H]^+^.

#### 1-Boc-4-(2-(3,4-dichloro-5-methyl-1*H*-pyrrole-2-carboxamido)-5-isopropylphenoxy)piperidine (33a

The synthesis was performed according to general procedure D using compound **32a** (0.370 g, 1.11 mmol) as starting material. The product was purified by column chromatography using EtOAc:hexane = 1:4 as the mobile phase. Yield: 0.280 (50 %); white solid; R_f_(EtOAc:hexane = 1:4) = 0.33; ^1^H NMR (400 MHz, DMSO-*d*_6_): *δ* 12.36 (s, 1H, pyrrole-N*H*), 8.95 (s, 1H, pyrrole-CON*H*-Ar), 8.23 (d, *J* = 8.2 Hz, 1H, Ar–*H*), 7.03 (d, *J* = 1.6 Hz, 1H, Ar–*H*), 6.79 (dd, *J*_*1*_ = 8.3 Hz, *J*_*2*_ = 1.5 Hz, 1H, Ar–*H*), 4.73–4.65 (m, 1H, C*H*), 3.82–3.73 (m, 2H, C*H*_*2*_), 3.16–3.02 (m, 2H, C*H*_*2*_), 2.22 (s, 3H, pyrrole-C*H*_*3*_), 2.03–1.97 (m, 2H, C*H*_*2*_), 1.64–1.47 (m, 4H, 2 × C*H*_*2*_), 1.41 (s, 9H, C (C*H*_*3*_)_*3*_), 0.90 (t, *J* = 7.3 Hz, 3H, CH_2_C*H*_*3*_); MS (ESI+) C_25_H_33_Cl_2_N_3_O_4_ *m*/*z*: 510.7 [M+H]^+^.

#### 1-Boc-4-(2-(3,4-dichloro-5-methyl-1*H*-pyrrole-2-carboxamido)-5-ethoxyphenoxy)piperidine (33b).

The synthesis was performed according to general procedure D using compound **32b** (0.370 g, 1.10 mmol) as starting material. The product was purified by column chromatography using DCM:MeOH = 50:1 as the mobile phase. Yield: 0.320 g (57 %); off-white solid; R_f_(DCM:MeOH = 30:1) = 0.34; ^1^H NMR (400 MHz, DMSO-*d*_6_): *δ* 12.32 (s, 1H, pyrrole-N*H*), 8.83 (s, 1H, pyrrole-CON*H*-Ar), 8.20 (d, *J* = 8.9 Hz, 1H, Ar–*H*), 6.78 (d, *J* = 2.6 Hz, 1H, Ar–*H*), 6.55 (dd, *J*_*1*_ = 8.9 Hz, *J*_*2*_ = 2.6 Hz, 1H, Ar–*H*), 4.75–4.65 (m, 1H, C*H*), 4.01 (q, *J* = 7.0 Hz, 2H, C*H*_*2*_), 3.80–3.69 (m, 2H, C*H*_*2*_), 3.15–2.99 (m, 2H, C*H*_*2*_), 2.22 (s, 3H, pyrrole-C*H*_*3*_), 2.07–1.91 (m, 2H, C*H*_*2*_), 1.56–1.45 (m, 2H, C*H*_*2*_), 1.41 (s, 9H, C(C*H*_*3*_)_*3*_), 1.32 (t, *J* = 7.0 Hz, 3H, OCH_2_C*H*_*3*_); MS (ESI+) C_24_H_31_Cl_2_N_3_O_5_ *m*/*z*: 509.9 [M+H]^+^.

#### 3,4-Dichloro-5-methyl-*N*-(2-(piperidin-4-yloxy)-4-propylphenyl)-1*H*-pyrrole-2-carboxamide×CF_3_COOH (34a).

The synthesis was performed according to the general procedure I using compound **33a** (0.140 g, 0.274 mmol) as starting material. The solvent was evaporated, and the residue was washed with Et_2_O to yield a pure product. Yield: 0.141 g (98 %); white solid; ^1^H NMR (400 MHz, DMSO-*d*_6_): *δ* 12.38 (s, 1H, pyrrole-N*H*), 8.95 (s, 1H, pyrrole-CON*H*-Ar), 8.55–8.43 (m, 1H, N*H*_*2*_^+^*-H*_*A*_), 8.41–8.29 (m, 1H, N*H*_*2*_^+^*-H*_*B*_), 8.22 (d, *J* = 8.2 Hz, 1H, Ar–*H*), 7.03 (d, *J* = 1.4 Hz, 1H, Ar–*H*), 6.82 (dd, *J*_*1*_ = 8.3 Hz, *J*_*2*_ = 1.4 Hz, 1H, Ar–*H*), 4.82–4.74 (m, 1H, C*H*), 3.16–3.02 (m, 2H, C*H*_*2*_), 2.23 (s, 3H, pyrrole-C*H*_*3*_), 2.21–2.14 (m, 2H, C*H*_*2*_), 1.89–1.78 (m, 2H, C*H*_*2*_), 1.65–1.53 (m, 2H, C*H*_*2*_), 0.90 (t, *J* = 7.3 Hz, 3H CH_2_C*H*_*3*_), the remaining 2 × C*H*_*2*_ are overlapped with solvent; MS (ESI+) C_20_H_25_Cl_2_N_3_O_2_ *m*/*z*: 409.7 [M+H]^+^.

#### 3,4-Dichloro-*N*-(4-ethoxy-2-(piperidin-4-yloxy)phenyl)-5-methyl-1*H*-pyrrole-2-carboxamide×CF_3_COOH (34b).

The synthesis was performed according to the general procedure I using compound **33b** (0.150 g, 0.293 mmol) as starting material. The solvent was evaporated, and the residue was washed with Et_2_O to yield a pure product. Yield: 0.153 g (100 %); off-white solid; ^1^H NMR (400 MHz, DMSO-*d*_6_): *δ* 12.35 (s, 1H, pyrrole-N*H*), 8.83 (s, 1H, pyrrole-CON*H*-Ar), 8.56–8.43 (m, 1H, N*H*_*2*_^+^*-H*_*A*_), 8.43–8.29 (m, 1H, N*H*_*2*_^+^*-H*_*B*_), 8.19 (d, *J* = 8.9 Hz, 1H, Ar–*H*), 6.79 (d, *J* = 2.6 Hz, 1H, Ar–*H*), 6.58 (dd, *J*_*1*_ = 8.9 Hz, *J*_*2*_ = 2.5 Hz, 1H, Ar–*H*), 4.82–4.73 (m, 1H, C*H*), 4.02 (q, *J* = 6.9 Hz, 2H, C*H*_*2*_), 3.35–3.28 (m, 2H, C*H*_*2*_), 3.13–3.02 (m, 2H, C*H*_*2*_), 2.22 (s, 3H, pyrroleC*H*_*3*_), 2.20–2.13 (m, 2H, C*H*_*2*_), 1.88–1.77 (m, 2H, C*H*_*2*_), 1.33 (t, *J* = 7.0 Hz, 3H, OCH_2_C*H*_*3*_); MS (ESI+) C_19_H_23_Cl_2_N_3_O_3_ *m*/*z*: 411.5 [M+H]^+^.

#### 3,4-Dichloro-5-methyl-*N*-(2-((1-methylpiperidin-4-yl)oxy)-4-propylphenyl)-1*H*-pyrrole-2-carboxamide (35a).

The synthesis was performed according to the general procedure E using compound **34a** (0.150 g, 0.293 mmol) as starting material. The product was purified by column chromatography using DCM/MeOH/NH_4_OH = 20:1:0.1 as the mobile phase. Yield: 0.055 g (85 %); white solid; R_f_(DCM:MeOH: NH_4_OH = 20:1:0.1) = 0.12; ^1^H NMR (400 MHz, DMSO-*d*_6_): *δ* 12.36 (s, 1H, pyrrole-N*H*), 8.97 (s, 1H, pyrrole-CON*H*-Ar), 8.24 (d, *J* = 8.2 Hz, 1H, Ar–*H*), 6.99 (d, *J* = 1.5 Hz, 1H, Ar–*H*), 6.78 (dd, *J*_*1*_ = 8.3 Hz, *J*_*2*_ = 1.5 Hz, 1H, Ar–*H*), 4.54–4.45 (m, 1H, C*H*), 2.75–2.62 (m, 2H, C*H*_*2*_), 2.25–2.14 (m, 8H, C*H*_*2*_ and NC*H*_*3*_ and pyrrole-C*H*_*3*_), 2.03–1.94 (m, 2H, C*H*_*2*_), 1.73–1.52 (m, 4H, 2 × C*H*_*2*_), 0.89 (t, *J* = 7.3 Hz, 3H, CH_2_C*H*_*3*_), the remaining C*H*_*2*_ is overlapped with solvent; ^13^C NMR (101 MHz, DMSO-*d*_6_): *δ* 156.0, 145.4, 138.1, 129.1, 125.9, 120.4, 119.2, 119.0, 113.3, 109.1, 108.3, 73.4, 52.5, 45.8, 37.1, 30.8, 24.2, 13.6, 10.7; HRMS (ESI+) calcd. for C_21_H_28_Cl_2_N_3_O_2_ [M+H]^+^: 424.15531, found: 424.15438; HPLC: t_r_ = 6.39 min (99.4 % at 254 nm).

#### 3,4-Dichloro-*N*-(4-ethoxy-2-((1-methylpiperidin-4-yl)oxy)phenyl)-5-methyl-1*H*-pyrrole-2-carboxamide (35b).

The synthesis was performed according to the general procedure E using compound **34b** (0.090 g, 0.171 mmol) as starting material. The product was purified by column chromatography using DCM/MeOH/NH_4_OH = 20:1:0.1 as the mobile phase. Yield: 0.049 g (68 %); white solid; R_f_ (DCM:MeOH: NH_4_OH = 20:1:0.1) = 0.11; ^1^H NMR (400 MHz, DMSO-*d*_6_): *δ* 12.33 (s, 1H, pyrrole-N*H*), 8.85 (s, 1H), 8.22 (d, *J* = 9.0 Hz, 1H, Ar–*H*), 6.73 (d, *J* = 2.5 Hz, 1H, Ar–*H*), 6.54 (dd, *J*_*1*_ = 9.0 Hz, *J*_*2*_ = 2.5 Hz, 1H, Ar–*H*), 4.57–4.49 (m, 1H, C*H*), 4.01 (q, *J* = 7.0 Hz, 2H, C*H*_*2*_), 2.28–2.15 (m, 8H, C*H*_*2*_ and NC*H*_*3*_ and pyrrole-C*H*_*3*_), 2.03–1.94 (m, 2H, C*H*_*2*_), 1.72–1.61 (m, 2H, C*H*_*2*_), 1.32 (t, *J* = 7.0 Hz, 3H, OCH_2_C*H*_*3*_), the remaining C*H*_*2*_ is overlapped with solvent; ^13^C NMR (101 MHz, DMSO-*d*_6_): *δ* 155.9, 155.3, 146.8, 128.9, 121.6, 120.3, 119.0, 108.9, 108.3, 105.3, 101.2, 73.5, 63.3, 52.4, 45.8, 30.7, 14.6, 10.7; HRMS (ESI+) calcd. for C_20_H_26_Cl_2_N_3_O_3_ [M+H]^+^: 426.13457, found: 426.13365; HPLC: t_r_ = 5.66 min (99.2 % at 254 nm).

#### 1-Boc-4-(2-amino-5-(methoxycarbonyl)phenoxy)piperidine (36).

The synthesis was performed according to the general procedure C using compound **13** (3.80 g, 6.99 mmol) as starting material. The residue was washed with Et_2_O to yield a pure product. Yield: 2.18 g (89 %); off-white solid; R_f_ (EtOAc:hexane = 2:1) = 0.56; ^1^H NMR (400 MHz, CDCl_3_): *δ* 7.54 (dd, *J*_*1*_ = 8.2 Hz, *J*_*2*_ = 1.7 Hz, 1H, Ar–*H*), 7.47 (d, *J* = 1.7 Hz, 1H, Ar–*H*), 6.68 (d, *J* = 8.2 Hz, 1H, Ar–*H*), 4.60–4.52 (m, 1H, C*H*), 4.21 (s, 2H, Ar-N*H*_*2*_), 3.86 (s, 1H 3.79–3.69 (m, 2H, C*H*_*2*_), 3.37–3.24 (m, 2H, C*H*_*2*_), 2.05–1.94 (m, 2H, C*H*_*2*_), 1.82–1.70 (m, 2H, C*H*_*2*_), 1.47 (s, 9H, C(C*H*_*3*_)_*3*_); ^13^C NMR (101 MHz, DMSO-*d*_6_): *δ* 166.8, 154.4, 145.1, 142.7, 124.8, 116.5, 115.1, 113.2, 79.1, 73.0, 51.8, 30.7, 28.6, 22.4; MS (ESI+) C_18_H_26_N_2_O_5_ *m*/*z*: 351.0 [M+H]^+^.

#### 1-Boc-4-(2-(3,4-dichloro-5-methyl-1*H*-pyrrole-2-carboxamido)-5-(methoxycarbonyl)phenoxy)piperidinee (37).

The synthesis was performed according to the general procedure D using compound **36** (0.700 g, 2.60 mmol) as starting material. Methanol was added to the residue and a precipitate formed that was filtered off to yield a pure product. Yield: 0.993 g (95 %); grey solid; ^1^H NMR (400 MHz, DMSO-*d*_6_): *δ* 12.49 (s, 1H, pyrrole-N*H*), 9.22 (s, 1H, pyrrole-CON*H*-Ar), 8.52 (d, *J* = 8.9 Hz, 1H, Ar–*H*), 7.68–7.60 (m, 2H, 2 × Ar–*H*), 4.87–4.79 (m, 1H, C*H*), 3.85 (s, 3H, COO–C*H*_*3*_), 3.84–3.76 (m, 2H, C*H*_*2*_), 3.17–3.05 (m, 2H, C*H*_*2*_), 2.24 (s, 3H, pyrrole-C*H*_*3*_), 2.07–1.98 (m, 2H, C*H*_*2*_), 1.60–1.49 (m, 2H, C*H*_*2*_), 1.41 (s, 9H, C(C*H*_*3*_)_*3*_); ^13^C NMR (101 MHz, CDCl_3_): *δ* 166.7, 157.3, 154.7, 145.3, 132.8, 129.2, 125.2, 123.4, 119.4, 118.9, 112.9, 111.9, 111.1, 79.9, 74.7, 52.2, 41.1, 31.0, 28.4, 11.5; MS (ESI+) C_24_H_29_Cl_2_N_3_O_6_ *m*/*z*: 525.8 [M+H]^+^.

#### Methyl 4-(3,4-dichloro-5-methyl-1*H*-pyrrole-2-carboxamido)-3-(piperidin-4-yloxy)benzoate×CF_3_COOH (38).

The synthesis was performed according to the general procedure I using compound **37** (0.187 g, 0.355 mmol) as starting material. The residue was washed with Et_2_O to yield a pure product. Yield: 0.181 g (94 %); grey solid; ^1^H NMR (400 MHz, DMSO-*d*_6_): *δ* 12.51 (s, 1H, pyrrole-N*H*), 9.22 (s, 1H, pyrrole-CON*H*-Ar), 8.56–8.48 (m, 2H, Ar–*H* and N*H*_*2*_^+^*-H*_*A*_), 8.38–8.30 (m, 1H, N*H*_*2*_^+^*-H*_*B*_), 7.72–7.64 (m, 2H, 2 × Ar–*H*), 4.99–4.89 (m, 1H, C*H*), 3.85 (s, 3H, COO–C*H*_*3*_), 3.17–3.05 (m, 2H, C*H*_*2*_), 2.24 (s, 3H, pyrrole-C*H*_*3*_), 2.23–2.17 (m, 2H, C*H*_*2*_), 1.92–1.80 (m, 2H, C*H*_*2*_), the remaining C*H*_*2*_ is overlapped with solvent; MS (ESI+) C_19_H_21_Cl_2_N_3_O_4_ *m*/*z*: 425.7 [M+H]^+^.

#### Methyl 4-(3,4-dichloro-5-methyl-1*H*-pyrrole-2-carboxamido)-3-((1-methylpiperidin-4-yl)oxy)benzoate (39).

The synthesis was performed according to the general procedure E using compound **38** (0.167 g, 0.309 mmol) as starting material. The product was purified by column chromatography using DCM/MeOH/NH_4_OH = 9:1:0.1 as the mobile phase. Yield: 0.111 g (82 %); white solid; R_f_(DCM:MeOH:NH_4_OH = 9:1:0.1) = 0.42; ^1^H NMR (400 MHz, DMSO-*d*_6_): *δ* 12.47 (s, 1H, pyrrole-N*H*), 9.23 (s, 1H, pyrrole-CON*H*-Ar), 8.52 (d, *J* = 8.4 Hz, 1H, Ar–*H*), 7.65–7.55 (m, 2H, 2 × Ar–*H*), 4.65–4.56 (m, 1H, C*H*), 3,84 (s, 3H, COO–C*H*_*3*_), 2.70–2.63 (m, 2H, C*H*_*2*_), 2.26–2.16 (m, 8H, pyrrole-C*H*_*3*_ and piperidine-C*H*_*3*_ and C*H*_*2*_), 2.06–1.97 (m, 2H, C*H*_*2*_), 1.76–1.65 (m, 2H, C*H*_*2*_); ^13^C NMR (101 MHz, DMSO-*d*_6_): *δ* 165.8, 156.4, 145.0, 132.8, 130.1, 124.4, 122.7, 118.6, 118.5, 113.1, 109.9, 108.7, 74.1, 52.4, 52.1, 45.7, 30.6, 10.8; HRMS (ESI+) calcd. for C_20_H_24_Cl_2_N_3_O_4_ [M+H]^+^: 440.11317, found: 440.11317; HPLC: t_r_ = 5.39 min (99.3 % at 254 nm).

#### General procedure K.

Corresponding methyl ester (1 equiv.) was taken up in a mixture of MeOH and THF (2:1, respectively). 1 M NaOH was added to the solution and the reaction mixture was stirred 1–3 nights at 50 °C. The solvent was evaporated and water was added to the residue. The solution was acidified to pH = 3 and the formed precipitate was filtered off, washed with Et_2_O and dried.

#### 4-(3,4-Dichloro-5-methyl-1*H*-pyrrole-2-carboxamido)-3-((1-methylpiperidin-4-yl)oxy)benzoic acid (40).

The synthesis was performed according to the general procedure K using compound **39** (0.167 g, 0.309 mmol) as starting material. Yield: 0.020 g (25 %); white solid; ^1^H NMR (400 MHz, DMSO-*d*_6_): *δ* 12.48 (s, 1H, pyrrole-N*H*), 9.22 (s, 1H, pyrrole-CON*H*-Ar), 8.49 (d, *J* = 9.0 Hz, 1H, Ar–*H*), 7.63–7.57 (m, 2H, 2 × Ar–*H*), 4.65–4.57 (m, 1H, C*H*), 2.77–2.69 (m, 2H, C*H*_*2*_), 2.29–2.20 (m, 8H, pyrrole-C*H*_*3*_ and piperidine-C*H*_*3*_ and C*H*_*2*_), 2.07–2.00 (m, 2H, C*H*_*2*_), 1.78–1.66 (m, 2H, C*H*_*2*_); ^13^C NMR (101 MHz, DMSO-*d*_6_): *δ* 167.0, 156.4, 145.0, 132.3, 123.0, 125.9, 122.8, 118.7, 118.5, 113.2, 109.9, 108.7, 79.3, 79.0, 78.7, 52.4, 45.5, 30.5, 10.8; HRMS (ESI+) calcd. for C_19_H_22_Cl_2_N_3_O_4_ [M+H]^+^: 426.09819, found: 426.09819; LC-MS: t_r_ = 4.56 min, (98.6 % at 254 nm).

#### 3-((1-(*tert*-Butoxycarbonyl)piperidin-4-yl)oxy)-4-(3,4-dichloro-5-methyl-1*H*-pyrrole-2-carboxamido)benzoic acid (41).

The synthesis was performed according to general procedure K using compound **37** (0.300 g, 0.570 mmol) as starting material. Yield: 0.205 g (70 %); brown solid; ^1^H NMR (400 MHz, DMSO-*d*_6_): *δ* 12.92 (s, 1H, COO*H*), 12.47 (s, 1H, pyrrole-N*H*), 9.20 (s, 1H, pyrrole-CON*H*-Ar), 8.49 (d, *J* = 8.4 Hz, 1H, Ar–*H*), 7.65–7.59 (m, 2H, 2 × Ar–*H*), 4.85–4.75 (m, 1H, C*H*), 3.86–3.75 (m, 2H, C*H*_*2*_), 3.20–3.02 (m, 2H, C*H*_*2*_), 2.24 (s, 3H, pyrrole-C*H*_*3*_), 2.08–1.97 (m, 2H, C*H*_*2*_), 1.61–1.48 (m, 2H, C*H*_*2*_), 1.41 (s, 9H, C (C*H*_*3*_)_*3*_); ^13^C NMR (101 MHz, DMSO-*d*_6_): *δ* 167.4, 156.8, 154.4, 145.3, 132.9, 130.4, 126.3, 123.4, 119.2, 119.0, 113.9, 110.3, 109.1, 79.3, 74.6, 41.5, 31.1, 28.5, 11.3; MS (ESI+) C_23_H_27_Cl_2_N_3_O_6_ *m*/*z*: 511.6 [M+H]^+^.

#### 1-Boc-4-(2-(3,4-dichloro-5-methyl-1*H*-pyrrole-2-carboxamido)-5-(dimethylcarbamoyl)phenoxy)piperidine (42a).

The synthesis was performed according to the general procedure G using compound **41** (0.120 g, 234 mmol) as the starting material. Yield: 0.120 g (95 %); off-white solid; R_f_(DCM:MeOH = 9:1) = 0.51; ^1^H NMR (400 MHz, CDCl_3_): *δ* 9.88 (s, 1H, pyrrole-N*H*), 9.23 (s, 1H, pyrrole-CON*H*-Ar), 8.51 (d, *J* = 8.3 Hz, 1H, Ar–*H*), 7.09 (d, *J* = 1.6 Hz, 1H, Ar–*H*), 7.03 (dd, *J*_*1*_ = 8.3 Hz, *J*_*2*_ = 1.6 Hz, 1H, Ar–*H*), 4.59–4.51 (m, 1H, C*H*), 4.01–3.88 (m, 2H, C*H*_*2*_), 3.17–3.00 (m, 8H, C*H*_*2*_ and CON(C*H*_*3*_)_*2*_), 2.31 (s, 3H, pyrrole-C*H*_*3*_), 2.12–2.03 (m, 2H, C*H*_*2*_), 1.80–1.65 (m, 2H, C*H*_*2*_), 1.48 (s, 9H, C(C*H*_*3*_)_*3*_); MS (ESI+) C_25_H_32_Cl_2_N_4_O_5_ *m*/*z*: 538.8 [M+H]^+^.

#### 1-Boc-4-(5-(cyclopropylcarbamoyl)-2-(3,4-dichloro-5-methyl-1*H*-pyrrole-2-carboxamido)phenoxy)piperidine (42b).

The synthesis was performed according to the general procedure G using compound **41** (0.120 g, 234 mmol) as the starting material. Yield: 0.111 g (86 %); light brown solid; R_f_(DCM:MeOH = 9:1) = 0.49; ^1^H NMR (400 MHz, DMSO-*d*_6_): *δ* 12.43 (s, 1H, pyrrole-N*H*), 9.12 (s, 1H, pyrrole-CON*H*-Ar), 8.41 (d, *J* = 8.5 Hz, 1H, Ar–*H*), 8.38 (d, *J* = 3.9 Hz, 1H, CON*H*–CH), 7.57 (d, *J* = 1.7 Hz, 1H, Ar–*H*), 7.48 (dd, *J*_*1*_ = 8.5 Hz, *J*_*2*_ = 1.7 Hz, 1H, Ar–*H*), 4.81–4.70 (m, 1H, O–C*H*), 3.86–3.74 (m, 2H, C*H*_*2*_), 3.18–3.05 (m, 2H, C*H*_*2*_), 2.86–2.77 (m, 1H, CONH–C*H*), 2.23 (s, 3H, pyrrole-C*H*_*3*_), 2.07–1.99 (m, 2H, C*H*_*2*_), 1.60–1.49 (m, 2H, C*H*_*2*_), 1.41 (s, 9H, C(C*H*_*3*_)_*3*_), 0.74–0.68 (m, 2H, CONH–CH(CH_2_C*H*_*2*_)), 0.59–0.54 (m, 2H, CONH–CH (C*H*_*2*_CH_2_)); MS (ESI−) C_26_H_32_Cl_2_N_4_O_5_ *m*/*z*: 548.6 [M − H]^−^.

#### 1-Boc-4-(2-(3,4-dichloro-5-methyl-1*H*-pyrrole-2-carboxamido)-5-((2-methoxyethyl)carbamoyl)phenoxy)piperidine-1-carboxylate (42c).

The synthesis was performed according to the general procedure G using compound **41** (0.120 g, 234 mmol) as the starting material. Yield: 0.117 g (88 %); off-white solid; ^1^H NMR (400 MHz, DMSO-*d*_6_): *δ* 10.32 (s, 1H, pyrrole-CON*H*-Ar), 8.44 (d, *J* = 8.4 Hz, 1H, Ar–*H*), 8.34 (s, 1H, CON*H*–CH_2_), 7.54 (d, *J* = 1.3 Hz, 1H, Ar–*H*), 7.46 (dd, *J*_*1*_ = 8.4 Hz, *J*_*2*_ = 1.3 Hz, 1H, Ar–*H*), 4.83–4.76 (m, 1H, O–C*H*), 3.76–3.48 (m, 4H, 2 × C*H*_*2*_), 3.46–3.39 (m, 4H, 2 × C*H*_*2*_), 3.27 (s, 3H, CH_2_–O–C*H*_*3*_), 2.02 (s, 3H, pyrrole-C*H*_*3*_), 1.89–1.71 (m, 4H, 2 × C*H*_*2*_), 1.40 (s, 9H, C(C*H*_*3*_)_*3*_); MS (ESI−) C_26_H_34_Cl_2_N_4_O_6_ *m*/*z*: 568.9 [M+H]^+^.

#### 1-Boc-4-(2-(3,4-dichloro-5-methyl-1*H*-pyrrole-2-carboxamido)-5-(propylcarbamoyl)phenoxy)piperidine (42d).

The synthesis was performed according to the general procedure G using compound **41** (0.120 g, 234 mmol) as the starting material. Yield: 0.121 g (93 %); light brown solid; R_f_(DCM:MeOH = 9:1) = 0.43; ^1^H NMR (400 MHz, CDCl_3_): *δ* 9.54 (s, 1H, pyrrole-N*H*), 9.27 (s, 1H, pyrrole-CON*H*-Ar), 8.53 (d, *J* = 8.5 Hz, 1H, Ar–*H*), 7.56 (d, *J* = 1.7 Hz, 1H, Ar–*H*), 7.21 (dd, *J*_*1*_ = 8.5 Hz, *J*_*2*_ = 1.7 Hz, 1H, Ar–*H*), 6.15 (t, *J* = 5.6 Hz, 1H, CON*H*–CH_2_), 4.71–4.61 (m, 1H, O–C*H*), 4.02–3.88 (m, 2H, C*H*_*2*_), 3.46–3.40 (m, 2H, C*H*_*2*_), 3.19–3.08 (m, 2H, C*H*_*2*_), 2.31 (s, 3H, pyrrole-C*H*_*3*_), 2.15–2.06 (m, 2H, C*H*_*2*_), 1.78–1.61 (m, 4H, 2 × C*H*_*2*_), 1.48 (s, 9H, C(C*H*_*3*_)_*3*_), 1.00 (t, *J* = 7.4 Hz, 3H, CH_2_–C*H*_*3*_); ^13^C NMR (101 MHz, DMSO-*d*_6_): *δ* 165.81, 156.79, 154.43, 145.37, 131.24, 130.29, 130.20, 120.80, 119.22, 118.94, 112.39, 110.16, 109.05, 79.33, 74.51, 41.49, 31.11, 28.52, 22.98, 11.97, 11.24; MS (ESI+) C_26_H_34_Cl_2_N_4_O_5_ *m*/*z*: 552.9 [M+H]^+^.

#### 1-Boc-4-(2-(3,4-dichloro-5-methyl-1*H*-pyrrole-2-carboxamido)-5-(isobutylcarbamoyl)phenoxy)piperidine (42e).

The synthesis was performed according to the general procedure G using compound **41** (0.120 g, 234 mmol) as the starting material. Yield: 0.128 g (93 %); brown solid; R_f_(DCM:MeOH = 9:1) = 0.54; ^1^H NMR (400 MHz, CDCl_3_): *δ* 9.54 (s, 1H, pyrrole-N*H*), 9.27 (s, 1H, pyrrole-CON*H*-Ar), 8.54 (d, *J* = 8.5 Hz, 1H, Ar-*H*), 7.56 (d, *J* = 1.7 Hz, 1H), 7.22 (dd, *J*_*1*_ = 8.5 Hz, *J*_*2*_ = 1.7 Hz, 1H, Ar–*H*), 6.19 (t, *J* = 5.8 Hz, 1H, CON*H*–CH_2_), 4.70–4.60 (m, 1H, O–C*H*), 4.01–3.91 (m, 2H, C*H*_*2*_), 3.34–3.25 (m, 2H, C*H*_*2*_), 3.17–3.06 (m, 2H, C*H*_*2*_), 2.31 (s, 3H, pyrrole-C*H*_*3*_), 2.14–2.06 (m, 2H, C*H*_*2*_), 1.98–1.86 (m, 1H, CH_2_C*H*(CH_3_)_2_), 1.78–1.67 (m, 2H, C*H*_*2*_), 1.48 (s, 9H, C(C*H*_*3*_)_*3*_), 0.99 (d, *J* = 6.7 Hz, 6H, CH_2_CH(C*H*_*3*_)_*2*_); MS (ESI+) C_27_H_36_Cl_2_N_4_O_5_ *m*/*z*: 566.4 [M+H]^+^.

#### 3,4-Dichloro-*N*-(4-(dimethylcarbamoyl)-2-(piperidin-4-yloxy)phenyl)-5-methyl-1*H*-pyrrole-2-carboxamide×CF_3_COOH(43a).

The synthesis was performed according to the general procedure I using compound **42a** (0.105 g, 0.195 mmol) as the starting material. Yield: 0.107 g (99 %); off-white solid; ^1^H NMR (400 MHz, DMSO-*d*_6_): *δ* 12.47 (s, 1H, pyrrole-N*H*), 9.10 (s, 1H, pyrrole-CON*H*-Ar), 8.56–8.48 (m, 1H, N*H*_*2*_^+^*-H*_*A*_), 8.41 (d, *J* = 8.3 Hz, 1H, Ar–*H*), 8.39–8.31 (m, 1H, N*H*_*2*_^+^*-H*_*B*_), 7.24 (d, *J* = 1.6 Hz, 1H, Ar–*H*), 7.06 (dd, *J*_*1*_ = 8.3 Hz, *J*_*2*_ = 1.6 Hz, 1H, Ar–*H*), 4.89–4.84 (m, 1H, C*H*), 3.41–3.29 (m, 2H, C*H*_*2*_), 3.14–3.03 (m, 2H, C*H*_*2*_), 2.96 (s, 6H, CON(C*H*_*3*_)_*2*_), 2.24 (s, 3H, pyrrole-C*H*_*3*_), 2.22–2.15 (m, 2H, C*H*_*2*_), 1.90–1.79 (m, 2H, C*H*_*2*_); MS (ESI+) C_20_H_24_Cl_2_N_4_O_3_ *m*/*z*: 438.8 [M+H]^+^.

#### 3,4-Dichloro-*N*-(4-(cyclopropylcarbamoyl)-2-(piperidin-4-yloxy)phenyl)-5-methyl-1*H*-pyrrole-2-carboxamide×CF_3_COOH (43b).

The synthesis was performed according to the general procedure I using compound **42b** (0.100 g, 0.181 mmol) as the starting material. Yield: 0.100 g (98 %); white solid; ^1^H NMR (400 MHz, DMSO-*d*_6_): *δ* 12.46 (s, 1H, pyrrole-N*H*), 9.13 (s, 1H, pyrrole-CON*H*-Ar), 8.59–8.49 (m, 1H, N*H*_*2*_^+^*-H*_*A*_), 8.44–8.33 (m, 3H, CON*H*–CH and Ar–*H* and N*H*_*2*_^+^*-H*_*A*_), 7.57 (d, *J* = 1.6 Hz, 1H, Ar–*H*), 7.50 (dd, *J*_*1*_ = 8.5 Hz, *J*_*2*_ = 1.6 Hz, 1H, Ar–*H*), 4.90–4.84 (m, 1H, O–C*H*), 3.40–3.30 (m, 2H, C*H*_*2*_), 3.17–3.05 (m, 2H, C*H*_*2*_), 2.86–2.78 (m, 1H, CONH–C*H*), 2.24 (s, 3H), 2.22–2.16 (m, 2H, C*H*_*2*_), 1.92–1.81 (m, 2H, C*H*_*2*_), 0.75–0.67 (m, 2H, CONH–CH (CH_2_C*H*_*2*_)), 0.61–0.51 (m, 2H, CONH–CH(C*H*_*2*_CH_2_)); MS (ESI+) C_21_H_24_Cl_2_N_4_O_3_ *m*/*z*: 450.9 [M+H]^+^.

#### 3,4-Dichloro-*N*-(4-((2-methoxyethyl)carbamoyl)-2-(piperidin-4-yloxy)phenyl)-5-methyl-1*H*-pyrrole-2-carboxamide×CF_3_COOH (43c).

The synthesis was performed according to the general procedure I using compound **42c** (0.93 g, 0.163 mmol) as the starting material. Yield: 0.095 g (100 %); off-white solid; ^1^H NMR (400 MHz, DMSO-*d*_6_): *δ* 12.47 (s, 1H, pyrrole-N*H*), 9.14 (s, 1H, pyrrole-CON*H*-Ar), 8.57–8.47 (m, 2H, CON*H*–CH_2_ and N*H*_*2*_^+^*-H*_*A*_), 8.46–8.35 (m, 2H, N*H*_*2*_^+^*-H*_*B*_ and Ar–*H*), 7.62 (d, *J* = 1.6 Hz, 1H, Ar–*H*), 7.55 (dd, *J*_*1*_ = 8.4 Hz, *J*_*2*_ = 1.6 Hz, 1H, Ar–*H*), 4.91–4.83 (m, 1H, O–C*H*), 3.27 (s, 3H, CH_2_–O–C*H*_*3*_), 3.16–3.05 (m, 2H, C*H*_*2*_), 2.27–2.19 (m, 5H, pyrrole-C*H*_*3*_ and C*H*_*2*_), 1.93–1.81 (m, 2H, C*H*_*2*_), the remaining 3 × C*H*_*2*_ are overlapped with solvent; MS (ESI+) C_21_H_26_Cl_2_N_4_O_4_ *m*/*z*: 468.9 [M+H]^+^.

#### 3,4-Dichloro-5-methyl-*N*-(2-(piperidin-4-yloxy)-4-(propylcarbamoyl)phenyl)-1*H*-pyrrole-2-carboxamide×CF_3_COOH (43d).

The synthesis was performed according to the general procedure I using compound **42d** (0.110 g, 0.199 mmol) as the starting material. Yield: 0.111 g (98 %); off-white solid; ^1^H NMR (400 MHz, DMSO-*d*_6_): *δ* 12.46 (s, 1H, pyrrole-N*H*), 9.13 (s, 1H, pyrrole-CON*H*-Ar), 8.52–8.44 (m, 1H, N*H*_*2*_^+^*-H*_*A*_), 8.44–8.38 (m, 2H, CON*H*–CH_2_ and Ar–*H*), 8.38–8.30 (m, 1H, N*H*_*2*_^+^*-H*_*B*_), 7.60 (d, *J* = 1.7 Hz, 1H, Ar–*H*), 7.53 (dd, *J*_*1*_ = 8.5 Hz, *J*_*2*_ = 1.7 Hz, 1H, Ar–*H*), 4.91–4.83 (m, 1H, O–C*H*), 3.37–3.31 (m, 2H, C*H*_*2*_), 3.26–3.19 (m, 2H, C*H*_*2*_), 3.15–3.06 (m, 2H, C*H*_*2*_), 2.24 (s, 3H, pyrrole-C*H*_*3*_), 2.22–2.16 (m, 2H, C*H*_*2*_), 1.92–1.82 (m, 2H, C*H*_*2*_), 1.59–1.48 (m, 2H, C*H*_*2*_), 0.90 (t, *J* = 7.4 Hz, 3H, CH_2_–C*H*_*3*_); ^13^C NMR (101 MHz, DMSO-*d*_6_): *δ* 165.8, 158.6 (d, *J* = 34.7 Hz), 156.8, 145.2, 131.1, 130.4, 130.2, 120.9, 119.2, 119.1, 116.6 (q, *J* = 294.4 Hz), 112.2, 110.4, 109.1, 71.4, 41.7, 41.5, 28.1, 23.0, 12.0, 11.3; ^19^F NMR (376 MHz, DMSO) *δ* – 74.38; MS (ESI−) C_21_H_24_Cl_2_N_4_O_3_ *m*/*z*: 450.5 [M − H]^−^.

3,4-Dichloro-*N*-(4-(isobutylcarbamoyl)-2-(piperidin-4-yloxy)phenyl)-5-methyl-1*H*-pyrrole-2-carboxamide×CF_3_COOH (43e).

The synthesis was performed according to the general procedure I using compound **42e** (0.120 g, 0.199 mmol) as the starting material. Yield: 0.123 g (100 %); white solid; ^1^H NMR (400 MHz, DMSO-*d*_6_): *δ* 12.46 (s, 1H, pyrrole-N*H*), 9.13 (s, 1H, pyrrole-CON*H*-Ar), 8.53–8.47 (m, 1H, N*H*_*2*_^+^*-H*_*A*_), 8.44–8.39 (m, 2H, CON*H*–CH_2_ and Ar–*H*), 8.39–8.31 (m, 1H, N*H*_*2*_^+^*-H*_*B*_), 7.60 (d, *J* = 1.6 Hz, 1H, Ar–*H*), 7.55 (dd, *J*_*1*_ = 8.5 Hz, *J*_*2*_ = 1.6 Hz, 1H, Ar–*H*), 4.92–4.83 (m, 1H, O–C*H*), 3.39–3.32 (m, 2H, C*H*_*2*_), 3.16–3.06 (m, 4H, 2 × C*H*_*2*_), 2.24 (s, 3H, pyrrole-C*H*_*3*_), 2.23–2.16 (m, 2H, C*H*_*2*_), 1.93–1.78 (m, 3H, CH_2_C*H*(CH_3_)_2_ and C*H*_*2*_), 0.89 (d, *J* = 6.7 Hz, 6H, CH_2_CH(C*H*_*3*_)_*2*_); MS (ESI+) C_22_H_28_Cl_2_N_4_O_3_ *m*/*z*: 466.7 [M+H]^+^.

#### 3,4-Dichloro-*N*-(4-(dimethylcarbamoyl)-2-((1-methylpiperidin-4-yl)oxy)phenyl)-5-methyl-1*H*-pyrrole-2-carboxamide (44a).

The synthesis was performed according to the general procedure E using compound **43a** (0.090 g, 0.163 mmol) as the starting material. The product was purified by column chromatography using DCM/MeOH/NH_4_OH = 15:1:0.1 as the mobile phase. Yield: 0.020 g (27 %); white solid; R_f_(DCM:MeOH:NH_4_OH = 15:1:0.1) = 0.08; ^1^H NMR (400 MHz, DMSO-*d*_6_): *δ* 12.45 (s, 1H, pyrrole-N*H*), 9.11 (s, 1H, pyrrole-CON*H*-Ar), 8.41 (d, *J* = 8.3 Hz, 1H, Ar–*H*), 7.21 (s, 1H, Ar–*H*), 7.04 (dd, *J*_*1*_ = 8.3 Hz, *J*_*2*_ = 1.5 Hz, 1H, Ar–*H*), 4.72–4.60 (m, 1H, C*H*), 2.96 (s, 6H, CON(C*H*_*3*_)_*2*_), 2.45–2.36 (m, 2H, C*H*_*2*_), 2.24 (s, 3H, pyrrole-C*H*_*3*_), 2.15–2.02 (m, 2H, C*H*_*2*_), 1.85–1.69 (m, 2H, C*H*_*2*_), the remaining C*H*_*2*_ is overlapped with solvent; ^13^C NMR (101 MHz, DMSO-*d*_6_): *δ* 169.6, 156.3, 144.9, 131.7, 129.7, 129.0, 119.9, 118.8, 118.7, 111.9, 109.7, 108.5, 52.0, 44.5, 34.9, 29.8, 10.8; HRMS (ESI+) calcd. for C_21_H_27_Cl_2_N_4_O_3_ [M+H]^+^: 453.14547, found: 453.14480; HPLC: t_r_ = 4.44 min (98.5 % at 254 nm).

#### 3,4-Dichloro-*N*-(4-(cyclopropylcarbamoyl)-2-((1-methylpiperidin-4-yl)oxy)phenyl)-5-methyl-1*H*-pyrrole-2-carboxamide (44b).

The synthesis was performed according to the general procedure E using compound **43b** (0.090 g, 0.159 mmol) as the starting material. The product was purified by column chromatography using DCM/MeOH/NH_4_OH = 15:1:0.1 as the mobile phase. Yield: 0.021 g (28 %); white solid; R_f_(DCM:MeOH:NH_4_OH = 15:1:0.1) = 0.05; ^1^H NMR (400 MHz, DMSO-*d*_6_): *δ* 12.44 (s, 1H, pyrrole-N*H*), 9.15 (s, 1H, pyrrole-CON*H*-Ar), 8.42 (d, *J* = 8.5 Hz, 1H, Ar–*H*), 8.39 (d, *J* = 3.8 Hz, 1H, CON*H*–CH), 7.54 (d, *J* = 1.7 Hz, 1H, Ar–*H*), 7.48 (dd, *J*_*1*_ = 8.5 Hz, *J*_*2*_ = 1.7 Hz, 1H, Ar–*H*), 4.62–4.53 (m, 1H, O–C*H*), 2.85–2.77 (m, 1H, CONH–C*H*), 2.73–2.65 (m, 2H, C*H*_*2*_), 2.24–2.16 (m, 8H, pyrrole-C*H*_*3*_ and piperidine-C*H*_*3*_ and C*H*_*2*_), 2.06–1.98 (m, 2H, C*H*_*2*_), 1.77–1.63 (m, 2H, C*H*_*2*_), 0.75–0.67 (m, 2H, CONH–CH(CH_2_C*H*_*2*_)), 0.61–0.54 (m, 2H, CONH–CH(C*H*_*2*_CH_2_)); ^13^C NMR (101 MHz, DMSO-*d*_6_): *δ* 166.7, 156.3, 145.0, 130.8, 129.8, 129.5, 120.3, 118.7, 118.3, 111.8, 109.7, 108.6, 74.0, 52.5, 45.7, 30.7, 23.0, 10.8, 5.8; HRMS (ESI+) calcd. for C_22_H_27_Cl_2_N_4_O_3_ [M+H]^+^: 465.14547, found: 465.14484; HPLC: t_r_ = 4.56 min (99.2 % at 254 nm).

#### 3,4-Dichloro-*N*-(4-((2-methoxyethyl)carbamoyl)-2-((1-methylpiperidin-4-yl)oxy)phenyl)-5-methyl-1*H*-pyrrole-2-carboxamide (44c).

The synthesis was performed according to the general procedure E using compound **43c** (0.085 g, 0.146 mmol) as the starting material. The product was purified by column chromatography using DCM/MeOH/NH_4_OH = 15:1:0.1 as the mobile phase. Yield: 0.036 g (51 %); white solid; R_f_(DCM:MeOH:NH_4_OH = 15:1:0.1) = 0.03; ^1^H NMR (400 MHz, DMSO-*d*_6_): *δ* 12.44 (s, 1H, pyrrole-N*H*), 9.16 (s, 1H, pyrrole-CON*H*-Ar), 8.51 (t, *J* = 5.1 Hz, 1H, CON*H*–CH_2_), 8.44 (d, *J* = 8.5 Hz, 1H, Ar–*H*), 7.59 (d, *J* = 1.7 Hz, 1H, Ar–*H*), 7.52 (dd, *J*_*1*_ = 8.5 Hz, *J*_*2*_ = 1.7 Hz, 1H, Ar–*H*), 4.64–4.54 (m, 1H, O–C*H*), 3.50–3.40 (m, 4H, CON-H–C*H*_*2*_–C*H*_*2*_–O), 3.28 (s, 3H, CH_2_–O–C*H*_*3*_), 2.76–2.66 (m, 2H, C*H*_*2*_), 2.27–2.18 (m, 8H, pyrrole-C*H*_*3*_ and piperidine-C*H*_*3*_ and C*H*_*2*_), 2.08–2.00 (m, 2H, C*H*_*2*_), 1.78–1.66 (m, 2H, C*H*_*2*_); ^13^C NMR (101 MHz, DMSO-*d*_6_): *δ* 165.5, 156.3, 145.0, 130.8, 129.8, 129.4, 120.4, 118.7, 118.4, 111.7, 109.7, 108.6, 73.9, 70.6, 58.0, 52.5, 45.6, 30.7, 10.8; HRMS (ESI+) calcd. for C_22_H_29_Cl_2_N_4_O_4_ [M+H]^+^: 483.15604, found: 483.15524; HPLC: t_r_ = 4.41 min (95.3 % at 254 nm).

#### 3,4-Dichloro-5-methyl-*N*-(2-((1-methylpiperidin-4-yl)oxy)-4-(propylcarbamoyl)phenyl)-1*H*-pyrrole-2-carboxamide (44d).

The synthesis was performed according to the general procedure E using compound **43d** (0.100 g, 0.176 mmol) as the starting material. The product was purified by column chromatography using DCM/MeOH/NH_4_OH = 9:1:0.1 as the mobile phase. Yield: 0.080 g (98 %); white solid; R_f_(DCM:MeOH:NH_4_OH = 9:1:0.1) = 0.18; ^1^H NMR (400 MHz, DMSO-*d*_6_): *δ* 12.44 (s, 1H, pyrrole-N*H*), 9.15 (s, 1H, pyrrole-CON*H*-Ar), 8.47–8.37 (m, 2H, and Ar–*H*), 7.57 (d, *J* = 1.6 Hz, 1H, Ar–*H*), 7.51 (dd, *J*_*1*_ = 8.5 Hz, *J*_*2*_ = 1.6 Hz, 1H, Ar–*H*), 4.63–4.53 (m, 1H, O–C*H*), 3.26–3.19 (m, 2H, C*H*_*2*_), 2.74–2.64 (m, 2H, C*H*_*2*_), 2.26–2.15 (m, 8H, pyrrole-C*H*_*3*_ and piperidine-C*H*_*3*_ and C*H*_*2*_), 2.08–1.98 (m, 2H, C*H*_*2*_), 1.76–1.64 (m, 2H, C*H*_*2*_), 1.59–1.48 (m, 2H, C*H*_*2*_), 0.90 (t, *J* = 7.4 Hz, 3H, CH_2_–C*H*_*3*_); ^13^C NMR (101 MHz, DMSO-*d*_6_): *δ* 165.3, 156.3, 145.0, 130.7, 129.8, 129.7, 120.2, 118.7, 118.4, 111.7, 109.7, 108.6, 74.0, 52.6, 45.7, 41.0, 30.7, 22.5, 11.5, 10.8; HRMS (ESI+) calcd. for C_22_H_29_Cl_2_N_4_O_3_ [M+H]^+^: 467.16112, found: 467.16052; HPLC: t_r_ = 4.95 min (98.2 % at 254 nm).

#### 3,4-Dichloro-*N*-(4-(isobutylcarbamoyl)-2-((1-methylpiperidin-4-yl)oxy)phenyl)-5-methyl-1*H*-pyrrole-2-carboxamide (44e).

The synthesis was performed according to the general procedure E using compound **43e** (0.100 g, 0.172 mmol) as the starting material. The product was purified by column chromatography using DCM/MeOH/NH_4_OH = 13:1:0.1 as the mobile phase. Yield: 0.038 g (46 %); white solid; R_f_(DCM:MeOH:NH_4_OH = 13:1:0.1) = 0.09; ^1^H NMR (400 MHz, DMSO-*d*_6_): *δ* 12.44 (s, 1H, pyrrole-N*H*), 9.16 (s, 1H, pyrrole-CON*H*-Ar), 8.47–8.37 (m, 2H, CON*H*–CH_2_ and Ar–*H*), 7.57 (d, *J* = 1.6 Hz, 1H, Ar–*H*), 7.52 (dd, *J*_*1*_ = 8.5 Hz, *J*_*2*_ = 1.6 Hz, 1H, Ar–*H*), 4.62–4.53 (m, 1H, O–C*H*), 3.09 (t, *J* = 6.4 Hz, 2H, C*H*_*2*_), 2.73–2.63 (m, 2H, C*H*_*2*_), 2.26–2.14 (m, 8H, pyrrole-C*H*_*3*_ and piperidine-C*H*_*3*_ and C*H*_*2*_), 2.07–1.99 (m, 2H, C*H*_*2*_), 1.90–1.79 (m, 1H, CH_2_C*H*(CH_3_)_2_), 1.75–1.65 (m, 2H, C*H*_*2*_), 0.89 (d, *J* = 6.7 Hz, 6H, CH_2_CH(C*H*_*3*_)_*2*_); ^13^C NMR (101 MHz, DMSO-*d*_6_): *δ* 165.5, 156.3, 145.1, 130.7, 129.8, 120.2, 118.7, 118.4, 111.7, 109.7, 108.6, 74.0, 52.5, 46.8, 45.7, 30.8, 28.2, 20.3; HRMS (ESI+) calcd. for C_23_H_31_Cl_2_N_4_O_3_ [M+H]^+^: 481.17677, found: 481.17602; HPLC: t_r_ = 5.05 min (97.7 % at 254 nm).

#### 3-((1-(Boc)piperidin-4-yl)oxy)-4-nitrobenzoic acid (45).

The synthesis was performed according to general procedure K using compound **13** (2.00 g, 5.26 mmol) as starting material. Yield: 1.60 g (84 %); yellow solid; R_f_(DCM:MeOH = 20:1) = 0.30; ^1^H NMR (400 MHz, DMSO-*d*_6_): *δ* 13.67 (s, 1H, COO*H*), 7.96 (d, *J* = 8.3 Hz, 1H, Ar–*H*), 7.82 (d, *J* = 1.4 Hz, 1H, Ar–*H*), 7.64 (dd, *J*_*1*_ = 8.3 Hz, *J*_*2*_ = 1.5 Hz, 1H, Ar–*H*), 4.96–5.02 (m, 1H, C*H*), 3.43–3.51 (m, 2H, C*H*_*2*_), 3.34–3.40 (m, 2H, C*H*_*2*_), 1.84–1.93 (m, 2H, C*H*_*2*_), 1.56–1.66 (m, 2H, C*H*_*2*_), 1.41 (s, 9H, C (C*H*_*3*_)_*3*_); MS (ESI+) C_17_H_22_N_2_O_7_ *m*/*z*: 410.2 [M+H]^+^.

#### 1-Boc-4-(5-((2-hydroxyethyl)carbamoyl)-2-nitrophenoxy)piperidine (46).

The synthesis was performed according to the general procedure G using compound **45** (1.00 g, 4.09 mmol) as the starting material. The product was additionally purified by column chromatography using DCM:MeOH = 15:1 as the mobile phase. Yield: 0.927 g (55 %); yellow oil; R_f_(DCM:MeOH = 15:1) = 0.16; ^1^H NMR (400 MHz, DMSO-*d*_6_): *δ* 8.72 (t, *J* = 5.5 Hz, 1H, Ar-CON*H*), 7.95 (d, *J* = 8.4 Hz, 1H, Ar–*H*), 7.77 (d, *J* = 1.4 Hz, 1H, Ar–*H*), 7.55 (dd, *J*_*1*_ = 8.4 Hz, *J*_*2*_ = 1.4 Hz, 1H, Ar–*H*), 4.98–4.92 (m, 1H, C*H*), 4.78 (t, *J* = 5.6 Hz, 1H, CH_2_–O*H*), 3.57–3.34 (m, 8H, 4 × C*H*_*2*_), 1.95–1.84 (m, 2H, C*H*_*2*_), 1.70–1.58 (m, 2H, C*H*_*2*_), 1.41 (s, 9H, C(C*H*_*3*_)_*3*_); MS (ESI+) C_19_H_27_N_3_O_7_ *m*/*z*: 410.2 [M+H]^+^.

#### 1-Boc-4-(2-amino-5-((2-(benzoyloxy)ethyl)carbamoyl)phenoxy)piperidine (47).

To compound **46** (0.813 g, 1.99 mmol) and K_2_CO_3_ (0.480 g, 2.89 mmol) dry THF (20 mL) was added under an argon atmosphere and then benzoyl chloride (0.500 mL, 4.30 mmol). The reaction was then stirred at 50 °C for 2 days. A precipitate that formed in the reaction mixture was filtered off and washed with water to yield a pure product. Yield: 0.802 g (79 %); light yellow oil; R_f_(DCM:MeOH = 20:1) = 0.17; ^1^H NMR (400 MHz, CDCl_3_): *δ* 8.10–8.02 (m, 2H, 2 × Ar–*H*), 7.83 (d, *J* = 8.3 Hz, 1H, Ar–*H*), 7.65–7.57 (m, 2H, 2 × Ar–*H*), 7.50–7.43 (m, 2H, 2 × Ar–*H*), 7.29 (dd, *J*_*1*_ = 8.3 Hz, *J*_*2*_ = 1.6 Hz, 1H, Ar–*H*), 6.89 (t, *J* = 4.9 Hz, 1H, Ar-CON*H*), 4.82–4.75 (m, 1H, C*H*), 4.64–4.58 (m, 2H, COO–C*H*_*2*_), 3.87 (dd, *J*_*1*_ = 10.2 Hz, *J*_*2*_ = 4.9 Hz, 2H, CONH–C*H*_*2*_), 3.63–3.45 (m, 4H, 2 × C*H*_*2*_), 1.98–1.80 (m, 4H, 2 × C*H*_*2*_), 1.47 (s, 9H, C(C*H*_*3*_)_*3*_); MS (ESI−) C_26_H_31_N_3_O_8_ *m*/*z*: 512.5 [M − H]^−^.

#### 1-Boc-4-(2-amino-5-((2-(benzoyloxy)ethyl)carbamoyl)phenoxy)piperidine (48).

The synthesis was performed according to the general procedure G using compound **47** (1.00 g, 4.09 mmol) as the starting material. The product was additionally purified by column chromatography using DCM:MeOH = 30:1 as the mobile phase. Yield: 0.584 g (78 %); white solid; R_f_(DCM:MeOH = 30:1) = 0.11; ^1^H NMR (400 MHz, CDCl_3_): *δ* 8.08–8.03 (m, 2H, 2 × Ar–*H*), 7.61–7.56 (m, 1H, Ar–*H*), 7.49–7.42 (m, 2H, 2 × Ar–*H*), 7.39 (d, *J* = 1.8 Hz, 1H, Ar–*H*), 7.14 (dd, *J*_*1*_ = 8.1 Hz, *J*_*2*_ = 1.8 Hz, 1H, Ar–*H*), 6.68 (d, *J* = 8.1 Hz, 1H, Ar–*H*), 6.50 (t, *J* = 5.2 Hz, 1H, Ar-CON*H*), 4.61–4.52 (m, 3H, C*H* and COO–C*H*_*2*_), 4.10 (s, 2H, N*H*_*2*_), 3.83 (dd, *J*_*1*_ = 10.6 Hz, *J*_*2*_ = 5.2 Hz, 2H, C*H*_*2*_), 3.74 (s, 2H, C*H*_*2*_), 3.33–3.23 (m, 2H, C*H*_*2*_), 2.03–1.93 (m, 2H, C*H*_*2*_), 1.80–1.69 (m, 2H, C*H*_*2*_), 1.47 (s, 9H, C(C*H*_*3*_)_*3*_); MS (ESI+) C_26_H_33_N_3_O_8_ *m*/*z*: 483.5 [M+H]^+^.

#### 1-Boc-4-(5-((2-(benzoyloxy)ethyl)carbamoyl)-2-(3,4-dichloro-5-methyl-1H-pyrrole-2-carboxamido)phenoxy)piperidine (49).

The synthesis was performed according to the general procedure G using compound **48** (1.00 g, 4.09 mmol) as the starting material. To the solid residue, EtOAc (30 mL) and water (20 mL) were added. The undissolved solid was filtered off, washed with EtOAc and dried to obtain a pure product. Yield: 0.252 g (34 %); brown solid; R_f_(DCM:MeOH = 9:1) = 0.37; ^1^H NMR (400 MHz, DMSO-*d*_6_): *δ* 12.44 (s, 1H, pyrrole-N*H*), 9.13 (s, 1H, pyrrole-CON*H*-Ar), 8.71–8.62 (m, 1H, Ar–*H*), 8.43 (d, *J* = 8.5 Hz, 1H, Ar–*H*), 8.06–7.91 (m, 2H, 2 × Ar–*H*), 7.69–7.46 (m, 5H, Ar-CON*H* and 4 × Ar–*H*), 4.79–4.69 (m, 1H, C*H*), 4.49–4.31 (m, 2H, C*H*_*2*_), 3.88–3.72 (m, 2H, C*H*_*2*_), 3.72–3.53 (m, 2H, C*H*_*2*_), 3.14–3.01 (m, 2H, C*H*_*2*_), 2.23 (s, 3H, pyrrole-C*H*_*3*_), 2.09–1.96 (m, 2H, C*H*_*2*_), 1.61–1.49 (m, 2H, C*H*_*2*_), 1.41 (s, 9H, C(C*H*_*3*_)_*3*_); MS (ESI−) C_32_H_36_Cl_2_N_4_O_7_ *m*/*z*: 656.9 [M − H]^−^.

#### 2-(4-(3,4-Dichloro-5-methyl-1*H*-pyrrole-2-carboxamido)-3-(piperidin-4-yloxy)benzamido)ethyl benzoate×HCl (50).

To a suspension of **49** (0.250 g, 3.87 mmol) in DMF (10 mL) 4 M HCl in 1,4-dioxane (3.87 mL, 155 mmol) was added and the reaction was stirred at 60 °C overnight. The solvent was evaporated and the residue was washed with MeOH to yield a pure product. Yield: 0.205 g (91 %); brown solid; ^1^H NMR (400 MHz, DMSO-*d*_6_): *δ* 12.47 (s, 1H, pyrrole-N*H*), 9.13 (s, 1H, pyrrole-CON*H*-Ar), 8.70 (t, *J* = 5.6 Hz, 1H, Ar-CON*H*), 8.60–8.52 (m, 1H, N*H*_*2*_^+^*-H*_*A*_), 8.48–8.39 (m, 2H, N*H*_*2*_^+^*-H*_*B*_ and Ar–*H*), 8.03–7.97 (m, 2H, Ar–*H*), 7.69–7.64 (m, 1H, Ar–*H*), 7.61 (d, *J* = 1.6 Hz, 1H, Ar–*H*), 7.56–7.50 (m, 4H, 4 × Ar–*H*), 4.90–4.82 (m, 1H, C*H*), 4.41 (t, *J* = 5.6 Hz, 2H, COO–C*H*_*2*_), 3.66 (dd, *J*_*1*_ = 11.3 Hz, *J*_*2*_ = 5.5 Hz, 2H, CONH–C*H*_*2*_), 3.13–3.03 (m, 2H, C*H*_*2*_), 2.27–2.16 (m, 5H, C*H*_*3*_ and C*H*_*2*_), 1.93–1.82 (m, 2H, C*H*_*2*_), the remaining C*H*_*2*_ is overlapped with solvent; MS (ESI+) C_27_H_28_Cl_2_N_4_O_5_ *m*/*z*: 558.8 [M − H]^−^.

#### 2-(4-(3,4-Dichloro-5-methyl-1*H*-pyrrole-2-carboxamido)-3-((1-methylpiperidin-4-yl)oxy)benzamido)ethyl benzoate (51).

The synthesis was performed according to the general procedure E using compound **50** (0.195 g, 0.327 mmol) as the starting material. The product was purified by column chromatography using DCM/MeOH = 9:1 as the mobile phase. Yield: 0.138 g (73 %); white solid; R_f_(DCM: MeOH = 9:1) = 0.19; ^1^H NMR (400 MHz, DMSO-*d*_6_): *δ* 12.44 (s, 1H, pyrrole-N*H*), 9.15 (s, 1H, pyrrole-CON*H*-Ar), 8.66 (t, *J* = 5.6 Hz, 1H, Ar-CON*H*), 8.43 (d, *J* = 8.4 Hz, 1H, Ar–*H*), 8.02–7.95 (m, 2H, 2 × Ar–*H*), 7.69–7.64 (m, 1H, Ar–*H*), 7.58–7.44 (m, 5H, 5 × Ar–*H*), 4.58–4.50 (m, 1H, C*H*), 4.41 (t, *J* = 5.6 Hz, 2H, COO–C*H*_*2*_), 3.65 (dd, *J*_*1*_ = 11.2 Hz, *J*_*2*_ = 5.6 Hz, 2H, CONH–C*H*_*2*_), 2.72–2.65 (m, 2H, C*H*_*2*_), 2.24 (s, 3H, C*H*_*3*_), 2.18 (s, 3H, C*H*_*3*_), 2.16–2.10 (m, 2H, C*H*_*2*_), 2.06–1.96 (m, 2H, C*H*_*2*_), 1.74–1.62 (m, 2H, C*H*_*2*_); ^13^C NMR (101 MHz, DMSO-*d*_6_): *δ* 166.4, 166.3, 156.8, 145.5, 133.8, 131.3, 130.3, 130.2, 129.8, 129.7, 129.2, 120.7, 119.2, 118.9, 112.1, 110.2, 109.1, 74.5, 63.8, 53.0, 49.1, 46.2, 31.2, 11.3; HRMS (ESI+) calcd. for C_28_H_31_Cl_2_N_4_O_5_ [M+H]^+^: 537.16660, found: 537.16594.

#### 3,4-Dichloro-*N*-(4-((2-hydroxyethyl)carbamoyl)-2-((1-methylpiperidin-4-yl)oxy)phenyl)-5-methyl-1*H*-pyrrole-2-carboxamide (52).

Compound **51** (0.080 g, 0.140 mmol) was suspended in MeOH (30 mL) and 2 M NaOH (0.698 mL, 1.4 mmol) was added and the reaction mixture was stirred at room temperature overnight. The solvent was evaporated and the product was purified by column chromatography using DCM:MeOH:NH_4_OH = 9:1:0.1 as the mobile phase. Yield: 0.043 g (66 %); white solid; R_f_(DCM:MeOH:NH_4_OH = 9:1:0.1) = 0.13; ^1^H NMR (400 MHz, DMSO-*d*_6_): *δ* 12.44 (s, 1H, pyrrole-N*H*), 9.16 (s, 1H, pyrrole-CON*H*-Ar), 8.45–8.39 (m, 2H, Ar-CON*H* and Ar–*H*), 7.59 (d, *J* = 1.6 Hz, 1H, Ar–*H*), 7.52 (dd, *J*_*1*_ = 8.5 Hz, *J*_*2*_ = 1.7 Hz, 1H, Ar–*H*), 4.76 (t, *J* = 5.5 Hz, 1H, CH_2_–O*H*), 4.63–4.55 (m, 1H, C*H*), 3.51 (q, *J* = 6.1 Hz, 2H, CONH–C*H*_*2*_ or C*H*_*2*_–OH), 2.73–2.66 (m, 2H, C*H*_*2*_), 2.24 (s, 3H, pyrrole-C*H*_*3*_), 2.22–2.13 (m, 5H, piperidine-C*H*_*3*_ and C*H*_*2*_), 2.07–1.99 (m, 2H, C*H*_*2*_), 1.76–1.65 (m, 2H, C*H*_*2*_), the remaining 2 × C*H*_*2*_ are overlapped with solvent; ^13^C NMR (101 MHz, DMSO-*d*_6_): *δ* 165.6, 156.3, 145.0, 130.8, 129.8, 129.6, 120.4, 118.7, 118.4, 111.7, 109.7, 108.6, 73.9, 59.8, 52.6, 45.6, 42.2, 30.7, 10.8; HRMS (ESI+) calcd. for C_21_H_27_Cl_2_N_4_O_4_ [M+H]^+^: 469.14039, found: 469.14017; HPLC: t_r_ = 3.97 min (99.4 % at 254 nm).

#### 1-Boc-4-(2-amino-5-(methoxycarbonyl)benzyl)piperidine (53).

Dimethylacetamide (10 mL) was added to methyl 3-bromo-4-nitrobenzoate (0.500 g, 1.92 mmol) and CH_3_COO–K^+^ (0.377 g, 3.84 mmol). The mixture was bubbled with argon and sonicated for 10 min. Then 1-Boc-4-methylenepiperidine (0.759 mL, 2 eq.) was added and the mixture was bubbled with argon for another 5 min. Finally, tetrakis (triphenylphosphine)-palladium (0.202 g, 0.288 mmol) was added and the reaction mixture was stirred overnight at 120 °C. The mixture was cooled and EtOAc (40 mL) and water (30 mL) were added to the mixture. The organic layer was additionally washed with water (30 mL), brine (30 mL), dried over Na_2_SO_4_ and filtered. The solvent was evaporated, and the intermediate was partly purified by column chromatography using EtOAc:hexane = 1:4 as the mobile phase (R_f_(EtOAc:hexane = 1:2) = 0.50). Crude product was then dissolved in methanol and palladium on charcoal (Pd/C) was added. The reaction mixture was then bubbled with hydrogen and left to stir overnight in hydrogen atmosphere. The reaction mixture was filtered through Celite^®^ and the solvent was evaporated under reduced pressure. The product was purified by column chromatography using EtOAc:hexane = 1:4 as the mobile phase. Two-step yield: 0.297 g (44 %); light pink oil; R_f_(EtOAc:hexane = 1:2) = 0.14; ^1^H NMR (400 MHz, CDCl_3_): *δ* 7.74 (dd, *J*_*1*_ = 8.3 Hz, *J*_*2*_ = 2.0 Hz, 1H, Ar–*H*), 7.69 (d, *J* = 2.0 Hz, 1H, Ar–*H*), 6.65 (d, *J* = 8.3 Hz, 1H, Ar–*H*), 4.14–4.07 (m, 2H, C*H*_*2*_), 4.00 (s, 2H, Ar-N*H*_*2*_), 3.86 (s, 3H, COO–C*H*_*3*_), 2.72–2.57 (m, 2H, C*H*_*2*_), 2.44 (d, *J* = 7.1 Hz, 2H, Ar-C*H*_*2*_-CH), 1.78–1.69 (m, 1H, Ar-CH_2_-C*H*), 1.68–1.60 (m, 2H, C*H*_*2*_), 1.45 (s, 9H, C(C*H*_*3*_)_*3*_), 1.25–1.14 (m, 2H, C*H*_*2*_); MS (ESI+) C_19_H_28_N_2_O_4_ *m*/*z*: 349.1 [M+H]^+^.

#### 1-Boc-4-(2-(3,4-dichloro-5-methyl-1*H*-pyrrole-2-carboxamido)-5-(methoxycarbonyl)benzyl)piperidine (54).

The synthesis was performed according to the general procedure D using compound **54** (0.250 g, 0.717 mmol) as starting material. Crude was first co-evaporated with toluene and further purified by column chromatography using DCM:MeOH = 30:1 as the mobile phase. Yield: 0.268 g (71 %); white solid; R_f_(DCM:MeOH = 30:1) = 0.27; ^1^H NMR (400 MHz, CDCl_3_): *δ* 9.39 (s, 1H, pyrrole-N*H*), 8.51 (s, 1H, pyrrole-CON*H*-Ar), 8.35 (d, *J* = 8.6 Hz, 1H, Ar–*H*), 7.94 (dd, *J*_*1*_ = 8.6 Hz, *J*_*2*_ = 1.8 Hz, 1H, Ar–*H*), 7.84 (d, *J* = 1.8 Hz, 1H, Ar–*H*), 4.16–4.01 (m, 2H, C*H*_*2*_), 3.92 (s, 3H, COO–C*H*_*3*_), 2.68 (d, *J* = 7.2 Hz, 2H, Ar-C*H*_*2*_-CH), 2.66–2.53 (m, 2H, C*H*_*2*_), 2.33 (s, 3H, pyrrole-C*H*_*3*_), 1.80–1.67 (m, 1H, Ar-CH_2_-C*H*), 1.65–1.58 (m, 2H, C*H*_*2*_), 1.45 (s, 9H, C(C*H*_*3*_)_*3*_), 1.28–1.16 (m, 2H, C*H*_*2*_); MS (ESI+) C_25_H_31_Cl_2_N_3_O_5_ *m*/*z*: 523.9 [M+H]^+^.

#### 3-((1-(Boc)piperidin-4-yl)methyl)-4-(3,4-dichloro-5-methyl-1*H*-pyrrole-2-carboxamido)benzoic acid (55).

The synthesis was performed according to the general procedure K using compound **47** (0.170 g, 0.324 mmol) as starting material. The reaction mixture was stirred for 3 days. Yield: 0.148 g (90 %); white solid; ^1^H NMR (400 MHz, DMSO-*d*_6_): *δ* 12.86 (s, 1H, COO*H*), 12.35 (s, 1H, pyrrole-N*H*), 8.86 (s, 1H, pyrrole-CON*H*-Ar), 8.02 (d, *J* = 8.2 Hz, 1H, Ar–*H*), 7.84–7.78 (m, 2H, 2 × Ar–*H*), 3.89 (d, *J* = 11.7 Hz, 2H, C*H*_*2*_), 2.74–2.55 (m, 4H, 2 × C*H*_*2*_), 2.23 (s, 3H, pyrrole-C*H*_*3*_), 1.79–1.67 (m, 1H, C*H*), 1.54–1.44 (m, 2H, C*H*_*2*_), 1.37 (s, 9H, C(C*H*_*3*_)_*3*_), 1.13–1.01 (m, 2H, C*H*_*2*_); MS (ESI+) C_24_H_29_Cl_2_N_3_O_5_ *m*/*z*: 508.6 [M+H]^+^.

#### 1-Boc-4-(2-(3,4-dichloro-5-methyl-1*H*-pyrrole-2-carboxamido)-5-(propylcarbamoyl)benzyl)piperidine (56).

The synthesis was performed according to the general procedure G using compound **55** (0.122 g, 0.239 mmol) as starting material. Yield: 0.120 g (92 %); off-white solid; R_f_(DCM:MeOH = 9:1) = 0.48; ^1^H NMR (400 MHz, CDCl_3_): *δ* 9.62 (s, 1H, pyrrole-N*H*), 8.48 (s, 1H, pyrrole-CON*H*-Ar), 8.27 (d, *J* = 8.5 Hz, 1H, Ar–*H*), 7.67 (d, *J* = 2.1 Hz, 1H, Ar–*H*), 7.58 (dd, *J*_*1*_ = 8.5 Hz, *J*_*2*_ = 2.1 Hz, 1H, Ar–*H*), 6.12 (t, *J* = 5.6 Hz, 1H, CON*H*CH), 4.18–3.96 (m, 2H, C*H*_*2*_), 3.48–3.37 (m, 2H, C*H*_*2*_), 2.67 (d, *J* = 7.2 Hz, 2H, C*H*_*2*_), 2.63–2.53 (m, 2H, C*H*_*2*_), 2.31 (s, 3H, pyrrole-C*H*_*3*_), 1.76–1.61 (m, 4H, 2 × C*H*_*2*_), 1.45 (s, 9H, C(C*H*_*3*_)_*3*_), 1.29–1.15 (m, 2H, C*H*_*2*_), 1.00 (t, *J* = 7.4 Hz, 3H, CH_2_C*H*_*3*_), 0.91–0.79 (m, 1H, C*H*); MS (ESI+) C_27_H_36_Cl_2_N_4_O_4_ *m*/*z*: 551.5 [M+H]^+^.

#### 3,4-Dichloro-5-methyl-*N*-(2-(piperidin-4-ylmethyl)-4-(propylcarbamoyl)phenyl)-1*H*-pyrrole-2-carboxamide×CF_3_COOH (57).

The synthesis was performed according to the general procedure I using compound **56** (0.105 g, 0.190 mmol) as starting material. The solvent was evaporated and the residue was washed with Et_2_O to yield a pure product. Yield: 0.108 g (100 %); white solid; ^1^H NMR (400 MHz, DMSO-*d*_6_): *δ* 12.33 (s, 1H, pyrrole-N*H*), 8.87 (s, 1H, pyrrole-CON*H*-Ar), 8.50–8.36 (m, 2H, N*H*_*2*_^+^), 8.19–8.05 (m, 1H, Ar–*H*), 7.92–7.85 (m, 1H, Ar–*H*), 7.78–7.71 (m, 2H, Ar–*H* and CON*H*CH), 3.27–3.17 (m, 4H, 2 × C*H*_*2*_), 2.83–2.71 (m, 2H, C*H*_*2*_), 2.71–2.65 (m, 2H, C*H*_*2*_), 2.23 (s, 3H, pyrrole-C*H*_*3*_), 1.91–1.79 (m, 1H, C*H*), 1.66 (d, *J* = 13.2 Hz, 2H, C*H*_*2*_), 1.60–1.47 (m, 2H, C*H*_*2*_), 1.40–1.26 (m, 2H, C*H*_*2*_), 0.90 (t, *J* = 7.4 Hz, 3H, CH_2_C*H*_*3*_); MS (ESI+) C_22_H_28_Cl_2_N_4_O_2_ *m*/*z*: 451.4 [M+H]^+^.

#### 3,4-Dichloro-5-methyl-*N*-(2-((1-methylpiperidin-4-yl)methyl)-4-(propylcarbamoyl)phenyl)-1*H*-pyrrole-2-carboxamide (58).

The synthesis was performed according to the general procedure E using compound **57** (0.078 g, 0.149 mmol) as starting material. The product was purified by column chromatography using DCM/MeOH/NH_4_OH = 9:1:0.1 as the mobile phase. Yield: 0.051 g (81 %); white solid; R_f_(DCM: MeOH:NH_4_OH = 9:1:0.1) = 0.18; ^1^H NMR (400 MHz, DMSO-*d*_6_): *δ* 12.31 (s, 1H, pyrrole-N*H*), 8.80 (s, 1H, pyrrole-CON*H*-Ar), 8.39 (t, *J* = 5.7 Hz, 1H, CON*H*CH), 7.90 (d, *J* = 8.6 Hz, 1H, Ar–*H*), 7.76–7.67 (m, 2H, 2 × Ar–*H*), 3.25–3.18 (m, 2H, C*H*_*2*_), 2.74–2.67 (m, 2H, C*H*_*2*_), 2.65–2.60 (m, 2H, C*H*_*2*_), 2.23 (s, 3H, pyrrole-C*H*_*3*_), 2.10 (s, 3H, NC*H*_*3*_), 1.79–1.67 (m, 2H, C*H*_*2*_), 1.58–1.45 (m, 5H, C*H* and 2 × C*H*_*2*_), 1.28–1.16 (m, 2H, C*H*_*2*_), 0.89 (t, *J* = 7.4 Hz, 3H CH_2_C*H*_*3*_); ^13^C NMR (101 MHz, DMSO-*d*_6_): *δ* 165.6, 157.2, 138.1, 132.1, 130.7, 129.7, 129.0, 125.4, 123.4, 119.1, 110.0, 108.4, 55.2, 46.0, 41.0, 38., 35.4, 31.8, 22.5, 11.5, 10.8; HRMS (ESI+) calcd. for C_23_H_31_Cl_2_N_4_O_2_ [M+H]^+^: 465.18241, found: 465.18036; HPLC: t_r_ = 5.07 min (96.9 % at 254 nm).

### 4.2. Molecular modelling

Molecular docking calculations were performed using Schrödinger Release 2022–1 (Schrödinger, LLC, New York, NY, USA, 2022). The co-crystal structures of Hsp90β (PDB entry: 5UCJ [8]), Hsp90α (PDB entry: 2XAB [57]) and topoisomerase IIα (PDB entry: 4R1F [58]) in complex with ligands were prepared using Protein Preparation Wizard with the default settings: bond orders were assigned using CCD database, missing hydrogens were added, termini were capped, the missing side chains were modelled with Prime, and het protonation states (pH 7.0 ± 2.0) were modelled with Epik [59]. The receptor grids were calculated for the ligand-binding sites. In the case of Hsp90α and Hsp90β, structural water molecule C (Fig. S4) was retained, while other crystal waters and cosolvents were deleted. Asp93 and Asp88 carboxylate groups were defined as hydrogen bond acceptor constraints, whereas water molecule C was defined as hydrogen bond donor constraint in Hsp90α and Hsp90β, respectively. In the case of TopoIIα, all crystal waters and cosolvents were deleted, and Asn102 carboxamide group was defined as hydrogen bond acceptor and donor constraint. Ligand structures were prepared using LigPrep module and ionized with Epik at pH = 7.4 using OPLS4 force field. The compounds were then docked using the Glide XP protocol as implemented in Schrödinger Release 2022–1 (Glide, Schrödinger, LLC, New York, NY, USA, 2022). The highest scored docking conformation was used for analysis and presentation.

Hsp90α or Hsp90β in docking complex with inhibitor **11** or **24e** was used as an input for molecular dynamics simulation using Desmond [60]. The structures of the docking complexes were prepared with System Builder. The TIP4P water molecules up to 10 Å from the protein surface were added to solvate the system in an orthorhombic box. Solvated system was then neutralized by adding sodium and chloride ions at a concentration of 0.15 M. OPLS_2005 force field [61] was used for parametrization of the protein-ligand complex. Default Desmond relaxation protocol was used for the equilibration stage: (1) 100 ps of Brownian dynamics NVT, 10 K, small timesteps, with restraints on the solute heavy atoms, (2) 12 ps NVT, 10 K, with small timesteps and restraints on the solute heavy atoms, (3) 12 ps NPT, 10 K, and restraints on the solute heavy atoms, (4) 24 ps unrestrained NPT. The equilibration was followed by the 500 ns long production stage: NPT ensemble at 300 K and 1.013 bar pressure with Langevin thermostat and barostat (1 and 2 ps relaxation time, respectively), RESPA integrator with 2 fs time step, cut-off scheme at 9.0 Å. Molecular dynamics trajectory was analyzed using Simulation Interactions Diagram algorithm in Maestro.

A list of human Hsp90α and Hsp90β binders were curated from the BindingDB [62] using the UNIPROT IDs P07900 and P08238, respectively. SMILES were converted to fingerprints using the ‘RDKFingerprint’ method in RDKit using a bitsize of 2048. Tanimoto similarity values were calculated with the ‘pdist’ method from the SciPy Python library.

### 4.3. Cloning, expression, and purification (Hsp90 NTD)

The N-terminal domain of Hsp90α and Hsp90β (Hsp90αN and Hsp90βN) encoding plasmids were constructed by inserting the DNA sequences encoding the N-terminal domain of human Hsp90α (corresponding to amino acids 1–241) and Hsp90β (corresponding to amino acids 1–239), respectively, into the pET21b vector (Novagen, Madison, WI, USA). Hsp90αN (S52A) mutant plasmid was created from the Hsp90αN plasmid by performing site-directed mutagenesis using PCR. All resulting protein constructs include an N-terminal His6 tag with a thrombin cleavage site.

Hsp90 NTD proteins were expressed in *Escherichia coli* BL21 (DE3) strain. Plasmid transformed bacterial cultures were grown in shaker flasks in LB media supplemented with ampicillin until OD600 of 0.6 at 37 °C. Then temperature was reduced to 30 °C and target protein expression was induced by the addition of 1 mM IPTG. 4 h after induction, bacteria were centrifuged and resuspended in buffer (25 mM Tris-HCl, 100 mM NaCl, 100 mM imidazole, pH 7.5) and lysed by sonication. Protein was purified from the soluble fraction using a Ni-IDA immobilized metal affinity column (Cytiva) followed by Q-Sepharose anion-exchange column (Cytiva). SDS-PAGE analysis determined protein purity to be higher than 95 %. Protein concentrations were determined by UV-VIS spectrophotometry.

### 4.4. Fluorescent-based thermal shift assay (FTSA)

Experiments were performed using Rotor-Gene Q 6-Plex spectrofluorimeter (excitation 365 nm, detection 460 nm). Solutions containing 10 μM of protein and various concentrations of ligand (0–200 μM) were heated up from 25 °C to 95 °C at the rate of 1 °C/min. Protein unfolding was detected using 8-anilino-1naphthalenesulfonate fluorescent dye at 100 μM concentration. Experiments were carried in buffer composing of 50 mM sodium phosphate, 100 mM sodium chloride, 2 % DMSO, pH 7.5. Fitting of melting curves and binding affinities were performed using Thermott [63].

### 4.5. Isothermal titration calorimetry (ITC)

Solutions containing 18–20 μM of protein were loaded into Malvern MicroCal PEAQ-ITC calorimeter’s cell. The protein solutions were titrated with 200 μM ligand solutions. Experiments were performed at a constant 25 °C temperature. The buffer used for experiments was composed of 50 mM sodium phosphate, 100 mM sodium chloride, 2 % DMSO, pH 7.5. Raw heat data was integrated using NITPIC (version 2.0.7) [64] and then subsequently a one-to-one binding model was applied using SEDPHAT (version 15.2b) [65].

### 4.6. Topoisomerase IIα relaxation assay

Inhibitory activities against TopoIIα were determined with commercially available relaxation assay kits (Inspiralis Limited, Norwich, UK) on Pierce^™^ streptavidin coated 96-well microtiter plates (Thermo Scientific, Rockford, IL, USA). The plates were rehydrated with wash buffer (20 mM Tris⋅HCl, 137 mM NaCl, 0.005 % w/v BSA, 0.05 % v/v Tween 20, pH 7.6). Then biotinylated triplex forming oligonucleotide dissolved in wash buffer added for 5 min to immobilize at room temperature. Excess oligonucleotide was washed off with wash buffer. Next, enzymatic reaction was performed: the reaction volume of 29 μL in buffer (50 mM Tris⋅HCl, 10 mM MgCl_2_, 125 mM NaCl, 5 mM DTT, 100 μg/mL albumin, 1 mM ATP, pH 7.5) contained 0.75 μg of supercoiled pNO1 plasmid, human DNA topoisomerase IIα (1.5 U in the initial screening and 1 U for IC_50_ determination), inhibitor, 1 % DMSO and 0.008 % Tween 20 was added in the screening. Reaction mixtures were incubated at 37 °C for 30 min. After that, the TF buffer (50 mM NaOAc, 50 mM NaCl and 50 mM MgCl_2_, pH 5.0) was added and the mixtures were incubated for 30 min at room temperature. During this period biotin–oligonucleotide–plasmid triplex was formed. Excess plasmid was washed off with TF buffer. The solution of Diamond Dye in T10 buffer (10 mM Tris⋅HCl, 1 mm EDTA, pH 8.0) was added next and the mixture was left to incubate for 15 min in the dark. Afterwards, fluorescence was measured with a microplate reader (BioTek Synergy H4, excitation: 485 nm, emission: 537 nm). Initial screening was performed at 100 μM concentration of the prepared final compounds. Compounds **44b**, **44d** and **44e** displayed some activity at 100 μM and were progressed to IC_50_ determination. As higher concentrations of the compounds were used, the samples were incubated in T10 buffer without Diamond Dye for 20 min and then transferred to Eppendorf tubes, extracted with 2 × 100 μL of butan-1-ol and the aqueous phase was returned to a fresh black-walled plate. DNA stain (Diamond Dye) was then added to 1X concentration, and the fluorescence was then measured (Ex: 495 nm; Em: 537 nm). As the positive control, etoposide (TCI, Tokyo, Japan; IC_50_ = 91 μM) was used. An average value for the negative (dilution buffer only for IC_50_ determination) and positive (human TopoIIα only) controls was calculated. The activity of the samples was then calculated as a percentage of the positive control. The percentage activities were then plotted against compound concentration, curves fitted (2-parameter, exponential decay: y = a*exp(−b*x)) and IC_50_ values were calculated (Fig. S343).

### 4.7. Cell culture

Hormone dependent breast cancer cell line MCF-7 (ATCC-HTB-22; ATCC) and Ewing sarcoma cancer cell line SK-N-MC (kind gift from Beat Schäfer) were cultured as monolayer. MCF-7 cells were maintained in low-glucose DMEM (Sigma – Aldrich, St. Louis, MO, USA), while SK-N-MC and K562 cells were grown in RPMI 1640 medium (Sigma-Aldrich, St. Louis, MO, USA). Both media were supplemented with 10 % heat inactivated fetal bovine serum albumin, 100 U/mL penicillin, 100 μg/mL streptomycin and 2 mM L-glutamine (Sigma-Aldrich, St. Louis, MO, USA). The cells were grown at 37 °C in a humidified atmosphere containing 5 % CO_2_.

### 4.8. MTS assay

The antiproliferative activities of the final compounds were evaluated using the breast cancer cell line MCF-7 (ATCC HTB-22; ATCC, Manassas, VA, USA), the Ewing sarcoma cell line SK-N-MC (a kind gift from Beat Schäfer), and the chronic myelogenous leukemia cell line K562 (ATCC CCL-243; ATCC, Manassas, VA, USA) via an MTS assay (Promega, Madison, WI, USA). MCF-7 and SK-N-MC cells are adherent, while K562 cells are suspended in the media. The assay was conducted according to the manufacturer’s instructions. MCF-7 cells were cultured in Dulbecco’s modified Eagle’s medium with low glucose (Sigma-Aldrich, St. Louis, MO, USA), while RPMI 1640 medium (Sigma-Aldrich, St. Louis, MO, USA) was used for SK-N-MC and K562 cells. Both media were supplemented with 10 % fetal bovine serum (Gibco, Thermo Fisher Scientific, Waltham, MA, USA), 100 U/mL penicillin (Sigma-Aldrich, St. Louis, MO, USA), 100 μg/mL streptomycin (Sigma-Aldrich, St. Louis, MO, USA), and 2 mM L-glutamine (Sigma-Aldrich, St. Louis, MO, USA). All cell types were incubated at 37 °C in a 5 % CO_2_ atmosphere. For the experiments, 96-well plates were used, with 2000 cells (MCF-7 and SK-N-MC) or 20,000 cells (K562) plated per well After the incubation period of 24 h the cells were treated with either the compounds or 1 μM 17-DMAG as the positive or 0.5 % DMSO as the vehicle control. After 72 h CellTiter96 Aqueous One Solution Reagent (10 μL; Promega, Madison, WI, USA) was added to all wells and after 3 h of incubation the absorbance was measured by a microplate reader (Synergy 4 Hybrid; BioTek, Winooski, VT, USA). Two repetitions of the experiments were performed, and each was carried out as a triplicate. The graphs were drawn using GraphPad Prism 8.0 software (San Diego, CA, USA) and the IC_50_ values, which represent the concentration at which the inhibitors produced a half maximal response. The results are presented as means from the independent measurements with given standard deviations.

### 4.9. Cytotoxic effects evaluation on a panel of cancer cell lines (NIH NCI-60)

Assays were performed according to previously published procedures [66].

### 4.10. In vivo no observed effect concentration (NOEC) determination in zebrafish larvae

The determination of NOEC was performed in zebrafish larvae (mitfa^b692/b692^; ednrba^b140/b140^) which were raised for 2 days post fertilization at 28 °C, afterwards they were kept at 34 °C. At 3 days post fertilization the larvae were moved into a 12-well plate (8–12 per well) (VWR, Avantor, Cat No. 10062–894). There they were treated with different concentrations of compound **24e** as indicated in the Kaplan-Meier curve. For better solubility and to ensure comparability with controls, DMSO concentration of 1 % was maintained in all wells. To obtain the final dilutions of **24e** for testing, respective amounts from a 10 mM stock solution were pre-diluted in DMSO. Next, these pre-diluted solutions were mixed with E3 larvae medium to a volume of 0.5 mL that was then added to 1 mL E3 per well containing the allotted fish larvae. To make a preliminary safety assessment, both survival rate and potential aberrations in development were observed and documented on the fourth and fifth day post fertilization. All experiments were conducted twice in technical triplicates.

### 4.11. Kinase panel assay

The assay was performed according to the manufacturer’s protocol [67], detailed description can be found in supplementary information.

### 4.12. Apoptosis evaluation

To assess the apoptosis of MCF-7 cells, detection of phosphatidylserines, by R-phycoerythrin – Annexin V conjugate (R-PE Annexin V; Invitrogen, Carlsbad, CA, USA) and nucleic acids in dead cells by SYTOX Blue Dead Cell Stain (Invitrogen, Carlsbad, CA, USA), was used as instructed by the manufacturer. Briefly, MCF-7 cells were seeded in a six-well plate at a density of 250,000 and were left to attach for 24 h. Afterwards the cells were washed with PBS and incubated in full DMEM medium containing vehicle control, 15 or 75 μM **24e** for 24–48 h. Detached cells were then collected with the medium. Next, attached cells were harvested and merged with the cells from the medium. Cold PBS was then used to wash the collected cells twice, and then the cells were resuspended in 100 μL annexin-binding buffer (Invitrogen, Carlsbad, CA, USA) containing 2.5 μL R-PE Annexin V solution and 750 nM SytoxBlue. Next, this mixture was incubated in the dark for 15 min at room temperature. 200 μL of annexin-binding buffer was added directly before the measurement. A minimum of 10,000 events were collected with a flow cytometer (Attune NxT; Invitrogen, Carlsbad, CA, USA). Annexin V (ANV)−/SYTOX Blue (SB)− indicated the viable cells that are not undergoing apoptosis, while ANV+/SB− showed early apoptotic or proapoptotic cells, ANV+/SB + colored the late apoptotic cells, and lastly ANV− /SB + indicated the necrotic cells.

### 4.13. Cell cycle assay

Propidium iodide (PI, Sigma-Aldrich, St. Louis, MO, USA) was used to perform analysis of the cell cycle arrest. First, 250,000 MCF-7 cells were seeded in a six-well plate and left to attach for 24 h. Thereafter, the cells were washed with PBS and treated in full DMEM medium containing vehicle control or 15 μM **24e** for 24–48 h. In the next step, the medium with detached cells was collected. The attached cells were harvested and merged with the cells from the medium. Cells were washed twice with PBS. Following the washing step, the cells were fixed and permeabilized by 15-min incubation with ice-cold 85 % ethanol at −20 °C. Then they were rehydrated with PBS at room temperature. Next, incubation of the cells with 500 μL of PI-binding buffer containing 1 μM PI and 1 mg/mL Ribonuclease A (Qiagen, Hilden, Germany) was performed in the dark for 15 min at room temperature. In the final step, a minimum of 10,000 events were collected with a flow cytometer (Attune NxT; Invitrogen, Carlsbad, CA, USA).

### 4.14. Thermodynamic solubility determination

For compounds **24e**, **24c**, **44d** and **52**, thermodynamic aqueous solubility determination was performed. In order to quantify the sample concentrations a standard curve was prepared with a UPLC (Thermo Scientific Dionex Ultimate 3000 Binary Rapid Separation liquid chromatography system, Thermo Fisher Scientific, USA) analysis using completely dissolved DMSO stock solutions with a concentration of 10 mM of the aforementioned compound. These stocks were diluted in 75 % 0.1 % TFA in water: 25 % MeCN to prepare samples for the calibration curves in at least six concentration points for each compound. Then the compounds (1–2 mg) were weighed into Eppendorf tubes and corresponding amount of PBS was added into each tube to reach the maximal concentration of 1 mg/mL. These tubes were then shaken at 20 RPM at 37 °C. Afterwards, the formed suspensions were centrifuged at 18000 RPM for 10 min at room temperature. The supernatants were pipetted off and further diluted (a 2-fold dilution) with 75 % 0.1 % TFA in water: 25 % MeCN to prepare the samples for UPLC (Thermo Scientific Dionex Ultimate 3000 Binary Rapid Separation liquid chromatography system, Thermo Fisher Scientific, USA) analyses. The UPLC system featured Waters C18 Acquity UPLC HSS column (1.8 μm, 2.1 × 50 mm) and as the mobile phase, 0.1 % TFA in ultrapure water (A) and acetonitrile (B) were used (gradient is given as % of B): 0–45 s 10 %, 45s–6.5 min 10%–90 %, 6.5–7 min 90 %%, 7–7.5 min 90%–10 %, 7.5–8 min 10 %; flow rate 0.4 mL/min and injection volume 10 μL. The obtained data was processed in Chromeleon CDS software (Thermo Fisher Scientific, USA) and solubilities were calculated in Excel (Microsoft, USA).

### 4.15. Western blotting

MCF-7 cells were treated with 25 μM and 10 μM of **24e** or 0.5 % DMSO. The cells were incubated with the compound or negative control for 24 h. Afterwards, they were rinsed with PBS (Gibco, Thermo Fisher Scientific, Waltham, MA, USA) and then lysed using RIPA buffer that comprised 50 mM Tris-HCl pH 7.4, 150 mM NaCl, 1 % NP-40, 0.5 % sodium deoxycholate, 1 mM EDTA. RIPA buffer was additionally supplemented with 1:100 Halt^™^ Protease Inhibitor Cocktail (Thermo Fisher Scientific, Waltham, MA, USA) and 1:100 Halt^™^ Phosphatase Inhibitor Cocktail (Thermo Fisher Scientific, Waltham, MA, USA). The lysates were then frozen for the minimal amount of 24 h. When the lysates were thawed, they were sonicated and then centrifuged at 15000 rpm for 20 min at 4 °C. After centrifugation only the supernatants were collected and form there protein concentration was measured using DC protein assay (Bio-Rad, Hercules, California, USA). Electrophoresis on SDS PAGE (10 % acrylamide/bisacrylamide gel) was then performed at 80 V for 15 min and afterwards at 130 V for 60 min to separate the isolated proteins (20 μg). Afterwards the separated proteins were transferred onto a nitrocellulose membrane using iBlot 2 Dry Blotting System (Thermo Fisher Scientific, Waltham, MA, USA). Then the membranes were incubated with 5 % BSA for 1 h at room temperature to block nonspecific binding sites. Then solutions of primary antibodies were added to the membranes, and they were incubated at 4 °C overnight. Primary antibodies (Cell Signaling, Danvers, MA. USA). that were used in the experiments encompassed anti-GAPDH Rabbit mAb (1:2500), anti-Hsp90 Rabbit mAb (1:1000), anti-Hsp70 Mouse mAb (1:1000), anti-IGF1R Rabbit (1:1000), anti-CDK4 Rabbit (1:1000), anti-ERK Rabbit mAb (1:1000), anti-Akt Rabbit mAb (1:1000), anti-cIAP1 Rabbit mAb (1:1000). As secondary antibodies anti-rabbit IgG, HRP-linked antibody (1:10000) and anti-mouse IgG, Hrp-linked antibody (1:10000) were used. Prior to detection, the membranes were incubated with secondary antibodies (Cell Signaling, Danvers, MA. USA) for 1 h at room temperature. After washing, the SuperSignal^™^ West Femto Maximum Sensitivity Substrate was added (Thermo Fisher Scientific, Waltham, MA, USA). For visualization of the blots UVITEC Cambridge Imaging System (UVITEC, Cambridge, UK) was used and quantification was performed by densitometric analysis of western blot bands. This was done by NineAlliance software. The relative densities were calculated in relation to GAPDH which was used as a loading control.

### 4.16. STD NMR spectroscopy

High-resolution NMR spectra were recorded on Bruker Avance Neo 600 MHz spectrometer using cryoprobes at 25 °C. Data were collected using the pulse sequences provided by the Bruker library of pulse programs and analyzed using Bruker Topspin 4.2.0. The residual water signal was suppressed by excitation-sculpting [68] with 2 ms selective pulses, and a T_1_ρ filter of 100 ms was used to eliminate background protein resonances. The ^1^H spectral widths were 5882 Hz. NMR samples were prepared in a buffer of 50 mM K-phosphate (pD 7.5), 100 mM KCl in D_2_O, complemented with 5 mM MgSO_4_, 2 mM DTT-*d*_10_, 0.02 % NaN_3_ and 2 % DMSO-*d*_6_. The complete assignment of protons (Tables S6 and S7) was achieved by combination of TOCSY and trNOESY spectra. The ^1^H STD and trNOESY spectra were recorded at a protein:ligand ratio of 1:200, where the protein concentration was 1.5 μM and the ligand concentration was 0.3 mM.

The ^1^H STD ligand epitope mapping experiments [69] were performed with 65536 data points, a relaxation delay of 1.63 s, and 5840 scans. The short protein saturation time of 0.5 s was used to avoid the influence of relaxation on the STD amplification factors [70]. The on-resonance selective saturation of Hsp90β was applied at −0.827 ppm with a transmitter offset referenced to 4.70 ppm. The off-resonance irradiation was applied at 30 ppm for the reference spectrum. The spectra were zero-filled and apodized with an exponential line-broadening function of 3 Hz. The errors in the STD amplification factor were estimated according to the formula [71]:

$$\text{STD}\;\text{amplification}\;\text{factor}\;\text{absolute}\;\text{error}=\text{STD}\;\text{amplification}\;\text{factor}\;\times {\left[\left({\left(\frac{N_{STD}}{I_{STD}}\right)}^{2}+{\left(\frac{N_{REF}}{I_{REF}}\right)}^{2}\right)\right]}^{\frac{1}{2}}$$

N_STD_ and N_REF_ are noise levels in STD and reference spectra. I_STD_ and I_REF_ are signal intensities in STD and reference spectra. The relative errors of the amplification factors for all protons of **24e** are less than 4 %.

The trNOESY [72] spectra were recorded with 4096 data points in t_2_, 64 scans, 324 complex points in t_1_, and a relaxation delay of 1.5 s. A mixing time of 350 ms was chosen according to the binding affinity of the derivatives to compromise between sufficient signal-to-noise ratio and reduced spin diffusion. The spectra were apodized with a squared sine bell function shifted by π/2 in both dimensions.

## Supplementary Material

SI

## Figures and Tables

**Table 1:** Inhibitory activities and binding of virtual screening hits on human TopoIIα, and Hsp90α and Hsp90β evaluated by DNA relaxation assay and fluorescence thermal shift assay, respectively.

Compound	Structure	IC_50_ (μM)	*K*_d_ (μM)	
		TopoIIα [39]	Hsp90α	Hsp90β

**1**	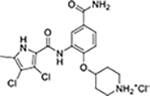	3.9 ± 3.9	≥200	≥200
**2**	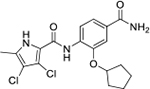	>40	≥200	≥200
**3**	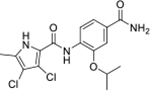	3.2 ± 1.8	≥200	≥200
**4**	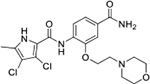	>100	≥200	≥200
**5**	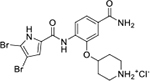	>100	≥200	≥200
**6**	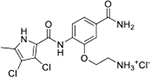	>100	≥200	≥200
**7**	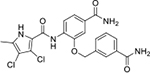	>100	≥200	≥200
**8**	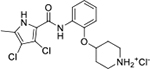	29 ± 19	≥200	87 CI^a^:[45]; 170]
**9**	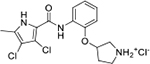	35 ± 1	≥200	26 CI[20; 34]
**10**	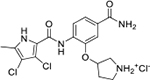	15 ± 9	≥200	17 CI:[11; 24]
**11**	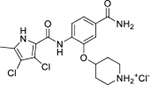	7.6 ± 0.4	≥200	16 CI:[11; 24]

a CI – confidence interval.

**Table 2:** Half maximal inhibitory concentration (IC_50_) of TopoIIα and dissociation constants with Hsp90α and Hsp90β with the first set of inhibitors (**17e**, **18a–d**) evaluated by the DNA relaxation assay and fluorescence-based thermal shift assay, respectively. The results of fluorescence-based thermal shift assay for KUNB-31, which was used as an Hsp90β-selective inhibitor positive control are also presented in the table.

Compound	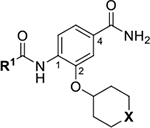		IC_50_ (μM)	*K*_d_ (μM)	
			
	R^1^	X	TopoIIα	Hsp90α	Hsp90β

**17e**	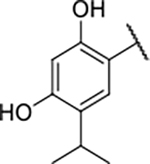	${\mathrm{NH}}_{2}^{+}{\mathrm{Cl}}^{-}$	>100	0.81 – CI:[0.76; 0.86]	2.7 – CI:[2.5; 2.8]
**18a**	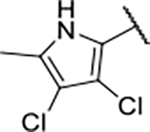	**NCH**_**3**_	>100	21 – CI:[14; 32]	3 – CI:[2.2; 4.2]
**18b**	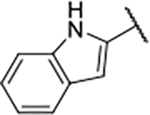	**NCH**_**3**_	>100	≥200	≥200
**18c**	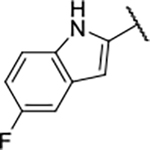	**NCH**_**3**_	>100	≥200	≥200
**18d**	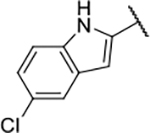	**NCH**_**3**_	>100	≥200	≥200
**KUNB-31**	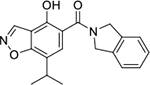		not tested	0.61 [0.50; 0.74]	0.026 [0.019; 0.034]

**Table 3:** Half maximal inhibitory concentration (IC_50_) of TopoIIα and dissociation constants with Hsp90α and Hsp90β with the second set of inhibitors (**22i–l**, **24a–h** and **30**) evaluated by the DNA relaxation assay and fluorescence thermal shift assay, respectively.

Compound	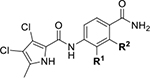		IC_50_ (μM)	*K*_d_ (μM)	
			
	R^1^	R^2^	TopoIIα	Hsp90α	Hsp90β

**22i**	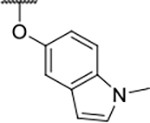	-H	>100	≥200	≥200
**22j**	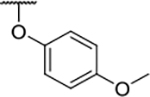	-H	>100	≥200	≥200
**22k**	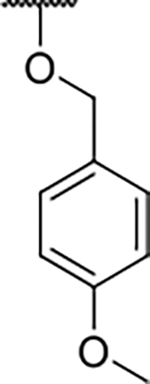	-H	>100	≥200	≥200
**22l**	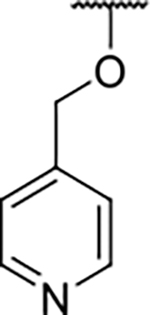	-H	>100	≥200	126
**24a**	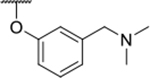	-H	>100	≥200	≥200
**24b**	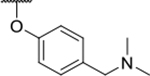	-H	>100	≥200	≥200
**24c**	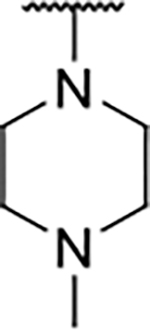	-H	>100	≥200	6.6 – CI:[4.4; 10]
**24d**	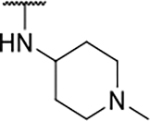	-H	>100	≥200	21 – CI:[10; 45]
**24e**	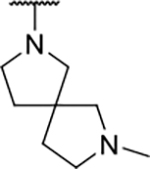	-H	>100	≥200	7.3 – CI:[4.8; 11]
**24f**	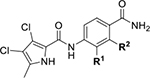	-H	>100	≥200	≥200
**24g**	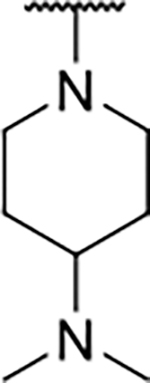	-H	>100	≥300	21– CI:[16; 26]1
**24h**	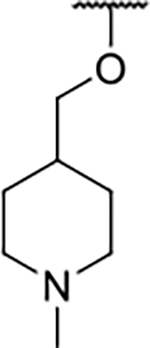	-H	>100	≥200	≥200
**30**	-H	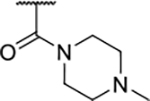	>100	≥200	≥200

**Table 4:** Half maximal inhibitory concentration (IC_50_) of TopoIIα given as mean ± SD and dissociation constants with Hsp90α and Hsp90β with the third set of inhibitors (**35a–b**, **39, 40**, **44a–e**, **52** and **58**) evaluated by the DNA relaxation assay and fluorescence-based thermal shift assay, respectively.

Compound	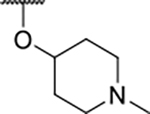		IC_50_ (μM)	*K*_d_ (μM)	
			
	R^1^	X	TopoIIα	Hsp90α	Hsp90β

**35a**	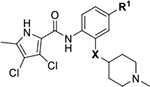	-O-	>100	≥200	110 – CI:[64; 180]
**35b**	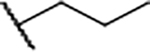	-O-	>100	≥200	49 – CI:[31; 79]
**39**	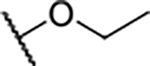	-O-	>100	40 – CI:[19; 81]	15 – CI:[12; 17]
**40**	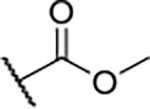	-O-	>100	≥200	10 – CI:[9; 12]
**44a**	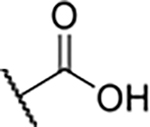	-O-	>100	24 – I:[14; 43]	4.7 – CI:[2.2; 10]
**44b**	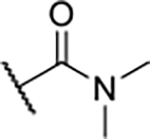	-O-	43 ± 5	12 – CI:[7; 20]	2 – CI:[1.1; 3.4]
**44c**	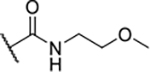	-O-	>100	≥200	14 – CI:[11; 18]
**44d**	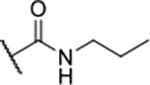	-O-	58 ± 0.2	23 – CI:[18; 29]	2.6 – CI:[2; 3.4]
**44e**	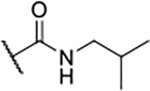	-O-	64 ± 11	60 – CI:[51; 71]	4.7 – CI:[3.5; 6.2]
**52**	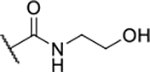	-O-	>100	7.4 – CI:[5.2; 10]	1.8 – CI:[1.3; 2.3]
**58**	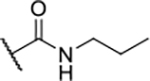	-CH_2_-	>100	≥200	≥200

## Data Availability

Data will be made available on request.
