# Supplementary material for: Hiding in plain sight: Optimizing topoisomerase IIα inhibitors into Hsp90β selective binders
Source: Eur J Med Chem. Author manuscript; Available in PMC 2025 May 19. (PMC12087459; doi:10.1016/j.ejmech.2024.116934)
Supplement: SI [file NIHMS2077251-supplement-SI.docx]

**Hiding in plain sight: Optimizing topoisomerase IIα inhibitors into Hsp90β selective binders**

Jaka Dernovšek^a^, Tjaša Goričan^b^, Marius Gedgaudas^c^, Živa Zajec^a^, Dunja Urbančič^a^, Ana Jug^a^, Žiga Skok^a^, Caterina Sturtzel^d^, Martin Distel^d^, Simona Golič Grdadolnik^b^, Kesavan Babu^e^, Ashna Panchamatia^e^, Timothy R Stachowski^e^, Marcus Fischer^e^, Janez Ilaš^a^, Asta Zubrienė^c^, Daumantas Matulis^c^, Nace Zidar^a,*^, Tihomir Tomašič^a,*^

*^a^ Faculty of Pharmacy, University of Ljubljana, Aškerčeva cesta 7, 1000 Ljubljana, Slovenia*

*^b^ Laboratory for Molecular Structural Dynamics, Theory Department, National Institute of Chemistry, Hajdrihova 19, 1001 Ljubljana, Slovenia*

*^c^Department of Biothermodynamics and Drug Design, Institute of Biotechnology, Vilnius University, Saulėtekio al. 7 (C319), LT-10257 Vilnius, Lithuania*

*^d^* *St. Anna Children's Cancer Research Institute, Zimmermannplatz 10, 1090 Vienna, Austria*

*^e^ Department of Chemical Biology and Therapeutics, St. Jude Children’s Research Hospital, 262 Danny Thomas Place, Memphis, TN 38105-3678, USA*

Table contents

[1. Molecular dynamics simulations 3](#_Toc176187378)

[2. Fluorescence Thermal Shift Assay (FTSA) 13](#_Toc176187379)

[3. Isothermal Titration Calorimetry (ITC) of 24e 15](#_Toc176187380)

[4. Evaluation of 24e on the kinase panel – supporting data 16](#_Toc176187381)

[5. NCI60 screening results and additional evaluation of 24e 18](#_Toc176187382)

[6. Western blot images used for quantification 28](#_Toc176187383)

[7. STD NMR study – supporting data 30](#_Toc176187384)

[8. Compound similarity analysis 33](#_Toc176187385)

[9. TopoIIα relaxation assay – curves for IC_50_ determination 34](#_Toc176187386)

[10. Representative ^1^H and ^13^C NMR spectra of the intermediates 35](#_Toc176187387)

# Molecular dynamics simulations


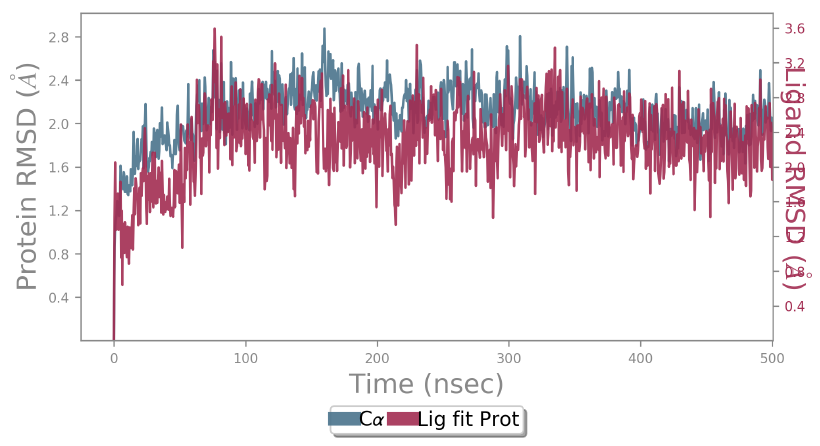


**Figure S1.** The protein and ligand root mean square deviation (RMSD) analysis of 500 ns molecular dynamics simulation of compound **11** in the ATP-binding site of Hsp90β.


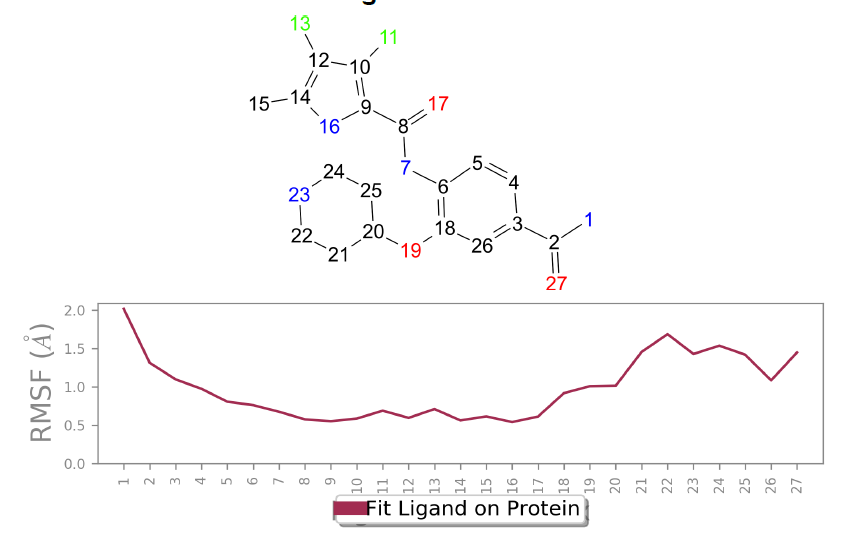


**Figure S2.** The ligand root mean square fluctuation (RMSF) analysis of 500 ns molecular dynamics simulation of compound **11** in the ATP-binding site of Hsp90β. Ligand RMSF shows the fluctuations of the ligand broken down by atom, which corresponds to the structure in the top panel. The bottom panel shows the ligand fluctuations with respect to the protein.


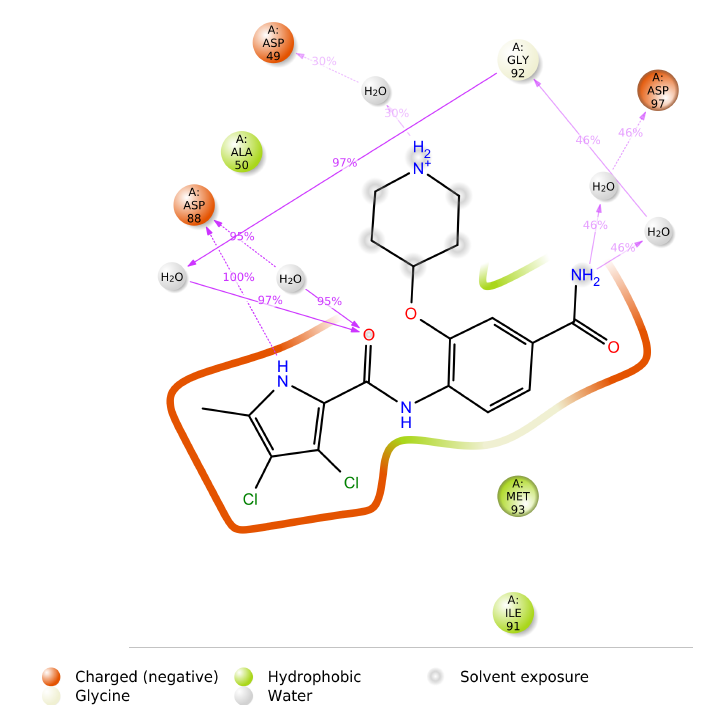


**Figure S3.** A schematic representation of ligand atom interactions with the protein residues. Interactions that occur more than 10.0% of the MD simulation time in the trajectory of compound **11** in the ATP-binding site of Hsp90β, are shown.


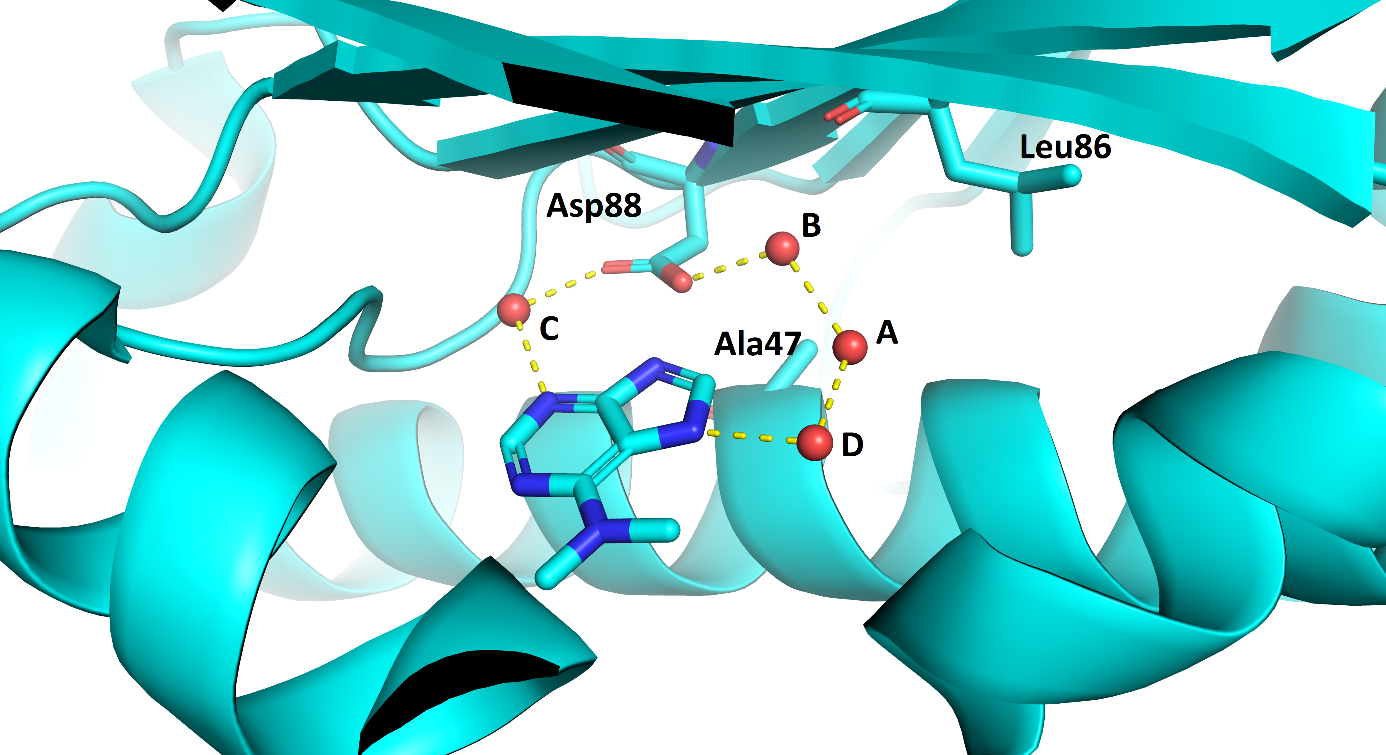


**Figure S4.** Conserved water molecule network in Hsp90β structure (PDB entry: 7ULJ).


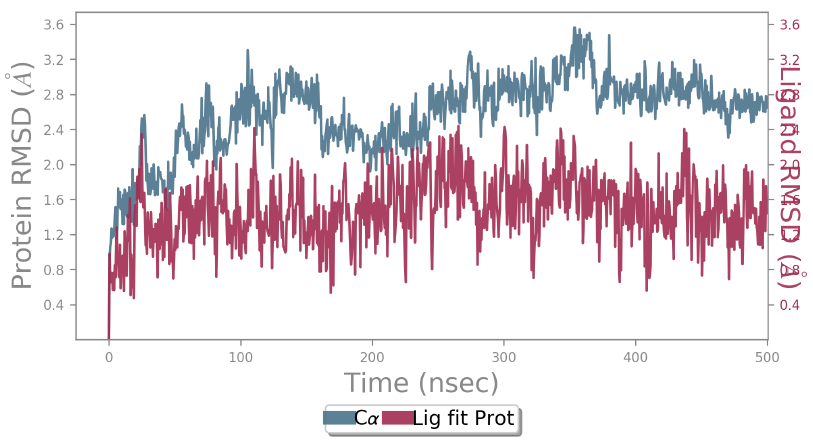


**Figure S5.** The protein and ligand root mean square deviation (RMSD) analysis of 500 ns molecular dynamics simulation of compound **(R)-24e** in the ATP-binding site of Hsp90β.

**
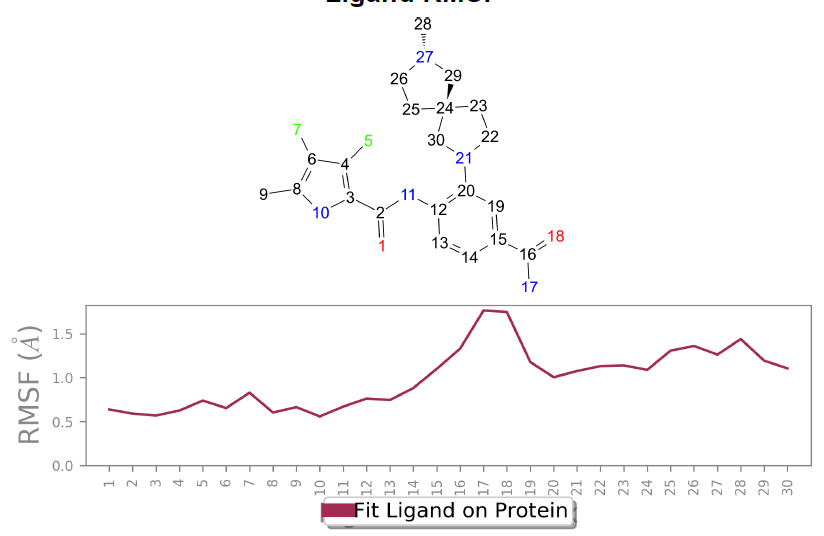
**

**Figure S6.** The ligand root mean square fluctuation (RMSF) analysis of 500 ns molecular dynamics simulation of compound **(R)-24e** in the ATP-binding site of Hsp90β. Ligand RMSF shows the fluctuations of the ligand broken down by atom, which corresponds to the structure in the top panel. The bottom panel shows the ligand fluctuations with respect to the protein.

**
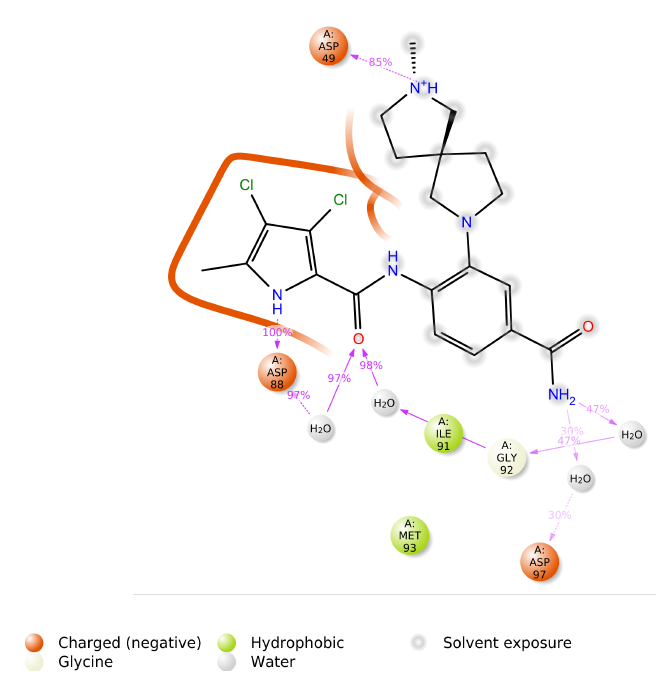
**

**Figure S7.** A schematic representation of ligand atom interactions with the protein residues. Interactions that occur more than 10.0% of the MD simulation time in the trajectory of compound **(R)-24e** in the ATP-binding site of Hsp90β, are shown.

**
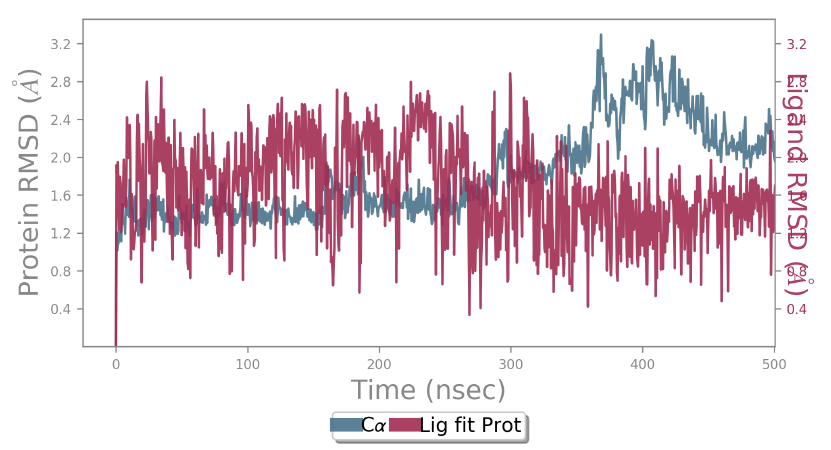
**

**Figure S8.** The protein and ligand root mean square deviation (RMSD) analysis of 500 ns molecular dynamics simulation of compound **(S)-24e** in the ATP-binding site of Hsp90β.


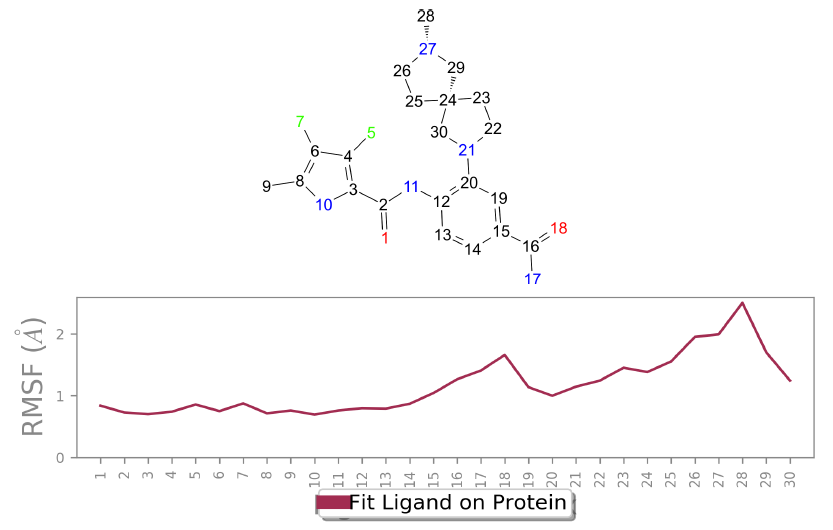


**Figure S9.** The ligand root mean square fluctuation (RMSF) analysis of 500 ns molecular dynamics simulation of compound **(S)-24e** in the ATP-binding site of Hsp90β. Ligand RMSF shows the fluctuations of the ligand broken down by atom, which corresponds to the structure in the top panel. The bottom panel shows the ligand fluctuations with respect to the protein.


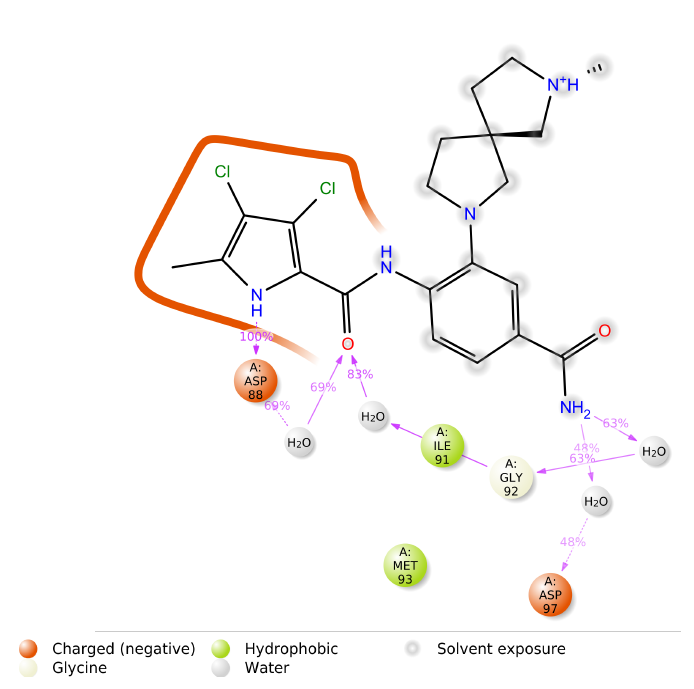


**Figure S10.** A schematic representation of ligand atom interactions with the protein residues. Interactions that occur more than 30.0% of the MD simulation time in the trajectory of compound **(S)-24e** in the ATP-binding site of Hsp90β, are shown.


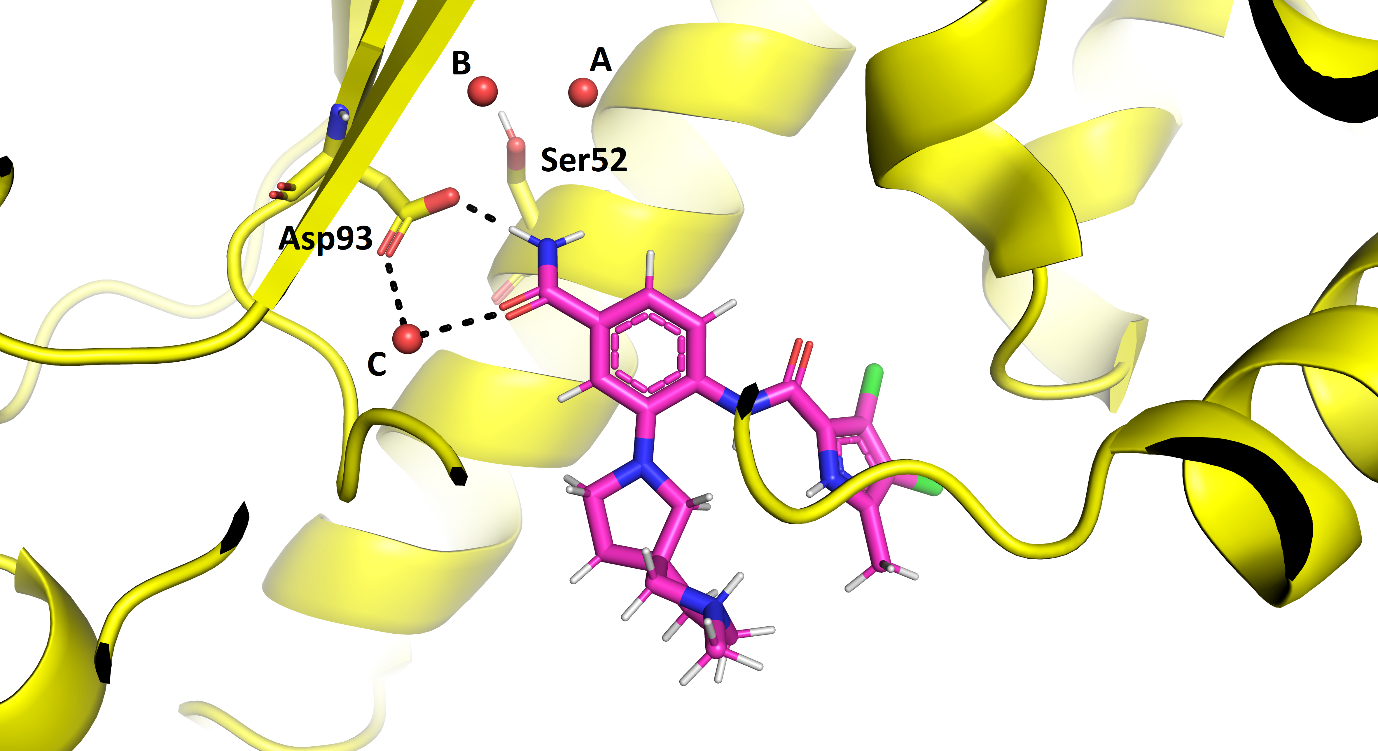


**Figure S11.** Docking binding mode of compound **(R)-24e** (in magenta sticks) in the ATP-binding site of Hsp90α (in yellow cartoon, PDB entry: 2XAB). Structural water molecules are shown as red spheres.

**
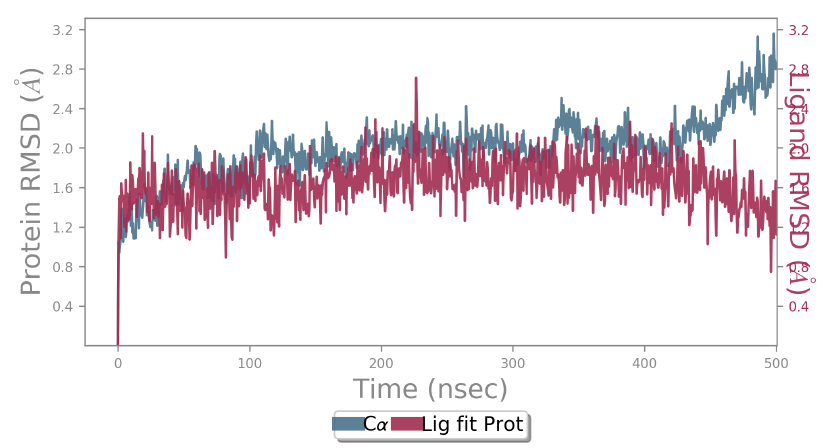
**

**Figure S12.** The protein and ligand root mean square deviation (RMSD) analysis of 500 ns molecular dynamics simulation of compound **(R)-24e** in the ATP-binding site of Hsp90α.


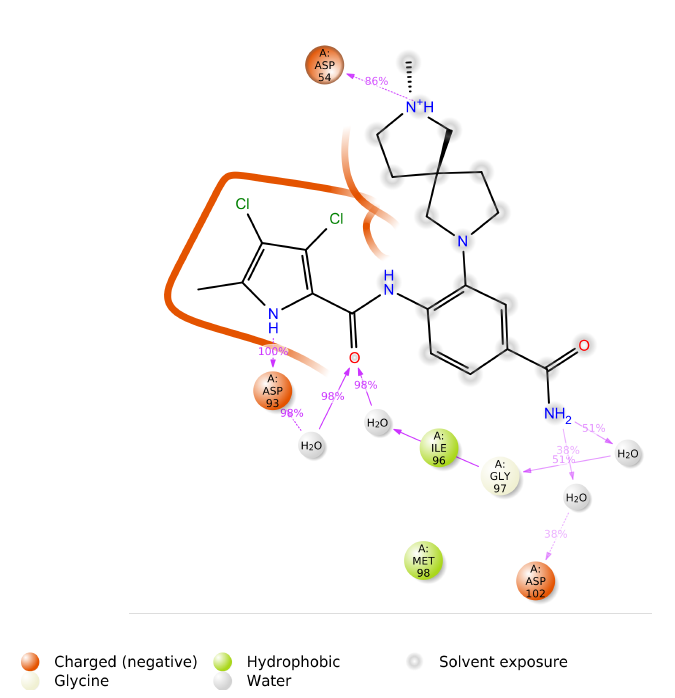


**Figure S13.** A schematic representation of ligand atom interactions with the protein residues. Interactions that occur more than 30.0% of the MD simulation time in the trajectory of compound **(R)-24e** in the ATP-binding site of Hsp90α, are shown.


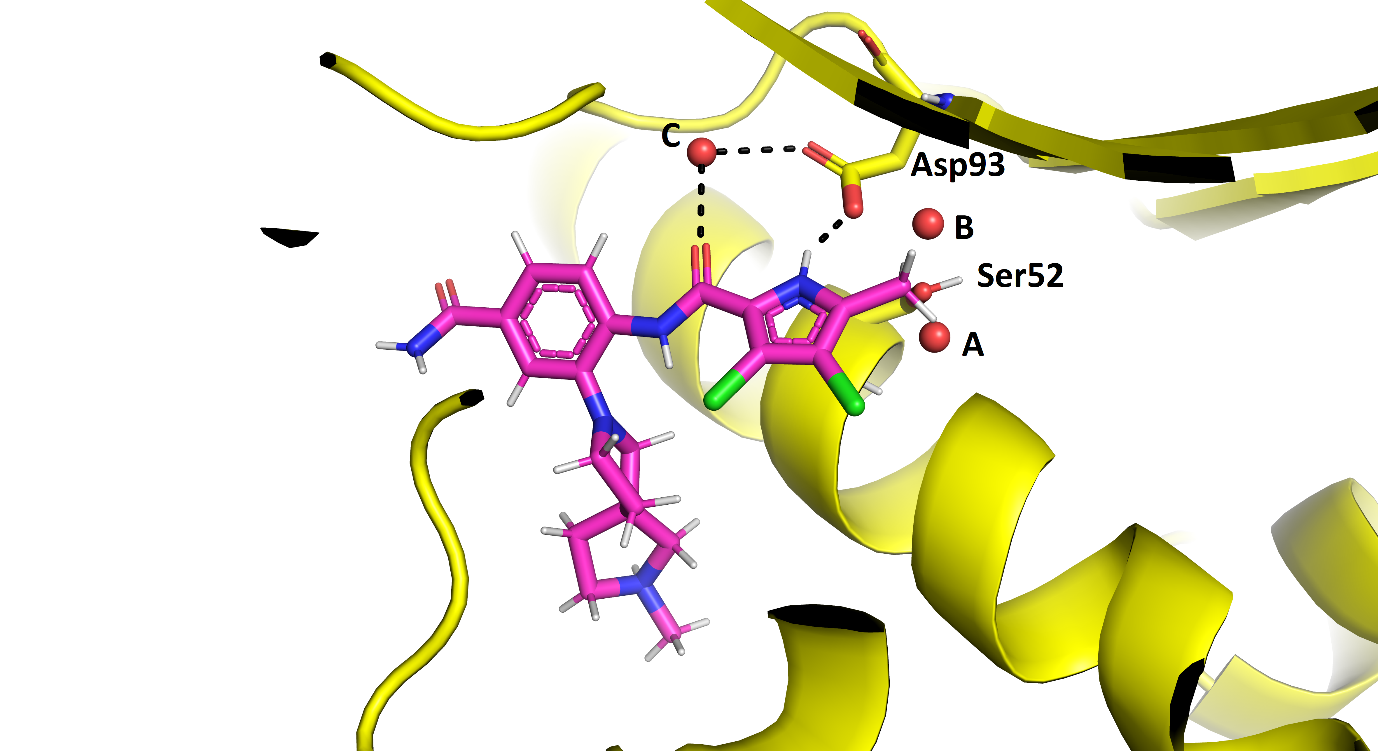


**Figure S14.** Overlap of the 5-methyl group of the pyrrole moiety of **(R)-24e** (in magenta sticks) with the waters A and B (red spheres) in the binding site of Hsp90α (in yellow cartoon, PDB entry: 2XAB).

# Fluorescence Thermal Shift Assay (FTSA)


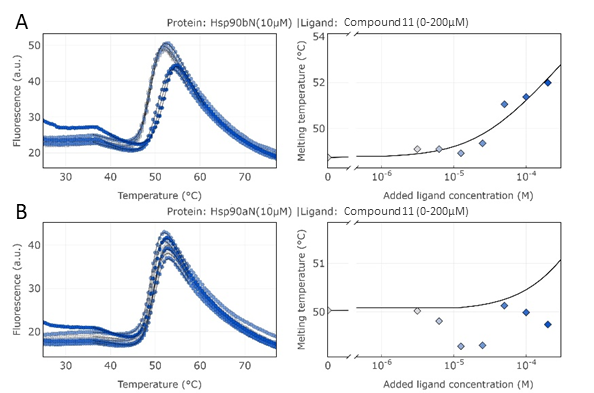


**Figure S15.** Representative FTSA curves and K_d_ calculation graphs for compound **11** with Hsp90β (K_d_ = 17 µM, **A**) and Hsp90α (K_d_ > 200 µM, **B**)


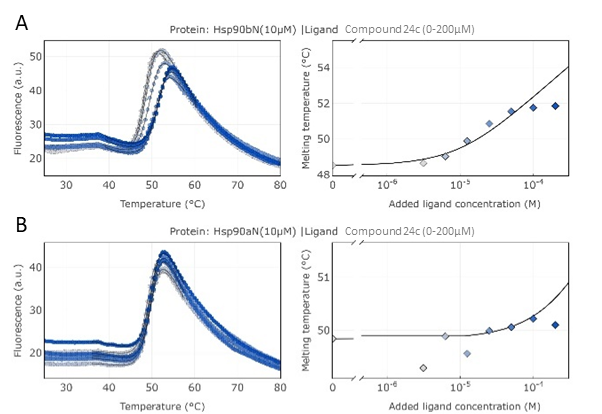


**Figure S16.** Representative FTSA curves and K_d_ calculation graphs for compound **24c** with Hsp90β (K_d_ = 6.3 µM, **A**) and Hsp90α (K_d_ > 200 µM, **B**)


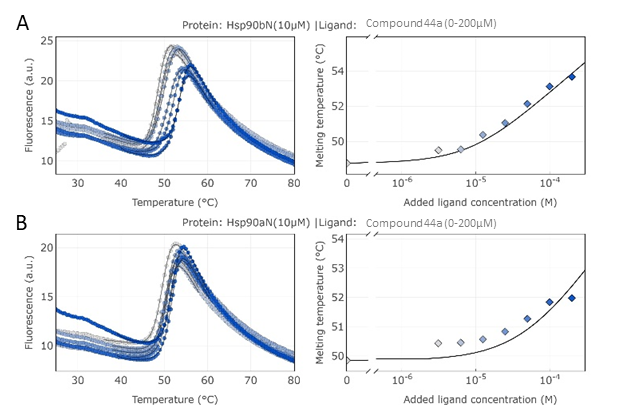


**Figure S17.** Representative FTSA curves and K_d_ calculation graphs for compound **44a** with Hsp90β (K_d_ = 5.5 µM, **A**) and Hsp90α (K_d_ = 32 µM, **B**)


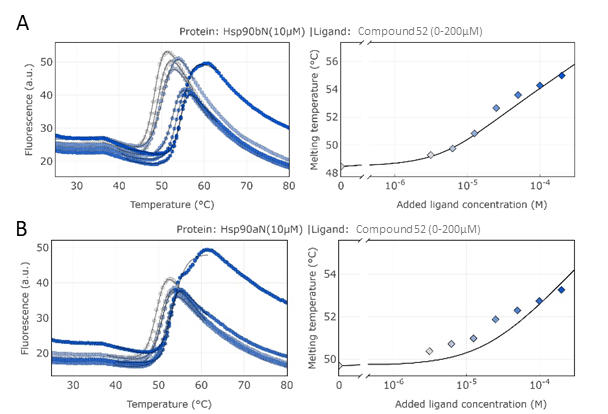


**Figure S18.** Representative FTSA curves and K_d_ calculation graphs for compound **44a** with Hsp90β (K_d_ = 2.0 µM, **A**) and Hsp90α (K_d_ = 12 µM, **B**)

**Table S1.** The dissociation constants K_d_ (µM) for compound interaction with Hsp90αN, Hsp90αN S52A and Hsp90βN isoforms determined using FTSA assay at 37 ◦C and pH 7.5.

| Ligand | *K*_d_, µM | | |
| --- | --- | --- | --- |
|  | Hsp90αN | Hsp90αN S52A | Hsp90βN |
| 17-AAG | 1.6 [1.4, 1.7] | 1.8 [2.1; 1.6] | 1.3 [1.0, 1.7] |
| KUNB31 | 0.61 [0.50; 0.74] | 0.25 [0.29; 0.21] | 0.026 [0.019 ;0.034] |
| **17e** | 0.81 [0.76;0.86] | 3.2 [4.2; 2.6] | 2.7 [2.5;2.8] |
| **18a** | 21 [14;32] | 5.1 [7.6; 3.5] | 3 [2.2;4.2] |
| **24e** | ≥200 | 9.5 [10.7, 8.5] | 7.3 [4.8;11] |
| **24g** | ≥200 | 6.7 [12; 3.6] | 21 [16; 26] |
| **44e** | 60 [51;71] | 3.9 [4.0 ; 3.8] | 4.7 [3.5;6.2] |

# Isothermal Titration Calorimetry (ITC) of 24e


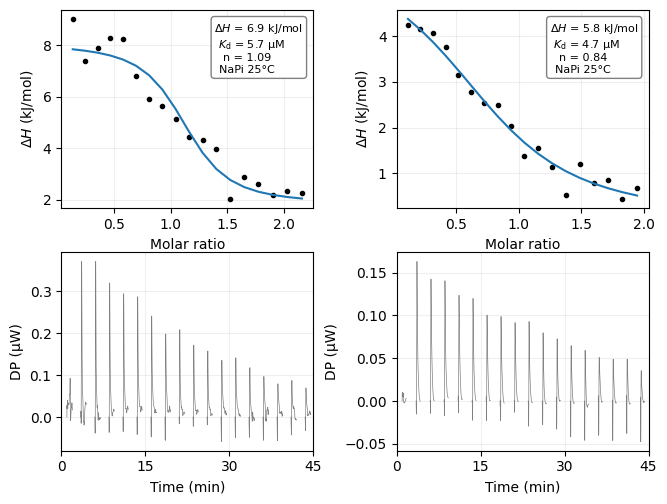


**Figure S19.** ITC curves for the determination of K_d_ value for **24e** with Hsp90βN.

# Evaluation of 24e on the kinase panel – supporting data

**Table S2.** Percentage of non-inhibited kinase activity when exposed to sorafenib and **24e**

|  | % of residual kinase activity when exposed to: | |
| --- | --- | --- |
| Kinase | sorafenib at 10 µM | **24e** at 10 µM |
| FGFR1 | 0.1 | 139.1 |
| JAK3 | 67.3 | 101.2 |
| LCK | 18.9 | 106.4 |
| SYK | 48.4 | 94.2 |
| MINK1 | 17.2 | 79.0 |
| PAK1 | 74.9 | 62.6 |
| IRAK4 | 99.7 | 71.5 |
| TAK1 | 2.4 | 83.0 |
| CDK2/cyclinE1 | 97.5 | 78.0 |
| GSK3β | 47.0 | 85.1 |
| p38 | 0.9 | 94.1 |
| AMPK A1/B1/G2 | 39.4 | 154.5 |
| CAMK4 | 82.0 | 97.0 |
| CHK1 | 91.3 | 101.3 |
| DAPK1 | 86.9 | 95.7 |
| AKT1 | 88.8 | 74.2 |
| PKCα | 87.3 | 97.1 |
| ROCK1 | 77.8 | 88.7 |
| Aurora A | 53.7 | 93.4 |
| CK2α1 | 103.4 | 105.5 |
| IKKβ | 95.0 | 170.7 |
| CK11 | 151.7 | 117.5 |

**Figure S20.** Graph representing residual kinase activity of known kinase inhibitor sorafenib when screened for inhibition at 10 µM.

**Kinase profiling – Method description**

Kinase profiling system – General Panel was purchased from Promega (Promega, Madison, WI, USA) and was carried out according to manufacturer’s protocol. General panel consists of 24 different kinases with substrate pairs. Each kinase had no-enzyme control wells, test wells with compound and no compound control wells. Firstly, 1 µL of compound solution was added to the wells. Then 2 µL of kinase solution and 2 µL of ATP/Substrate solutions were added to each well. Mixture was incubated ar room temperature for 60 minutes, then 5 µL of ADP-Glo™ Reagent (Promega, Madison, WI, USA) was added to all reactions in the plate. After 40 minutes incubation at room temperature 10 µL of Kinase Detection Reagent (Promega, Madison, WI, USA) was added to each well and incubated for another 30 minutes at room temperature. Luminescence was measured using Tecan's Spark Multimode Microplate reader (Tecan Trading AG, Switzerland). The kinase activity of 22 kinases was calculated as a percentage of luminescence of test wells divided by luminescence of no compound control wells.

# NCI60 screening results and additional evaluation of 24e


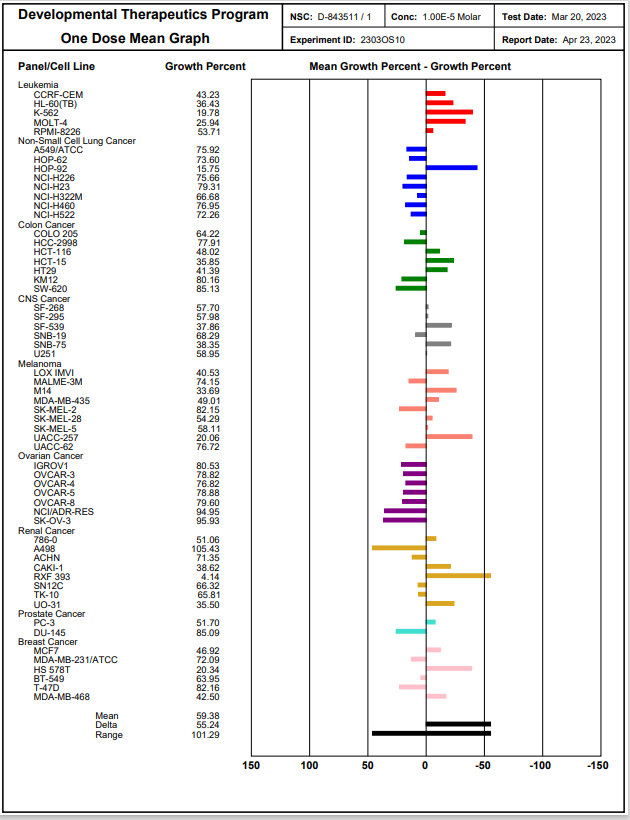


**Figure S21.** NCI60 screening results for **24e.**


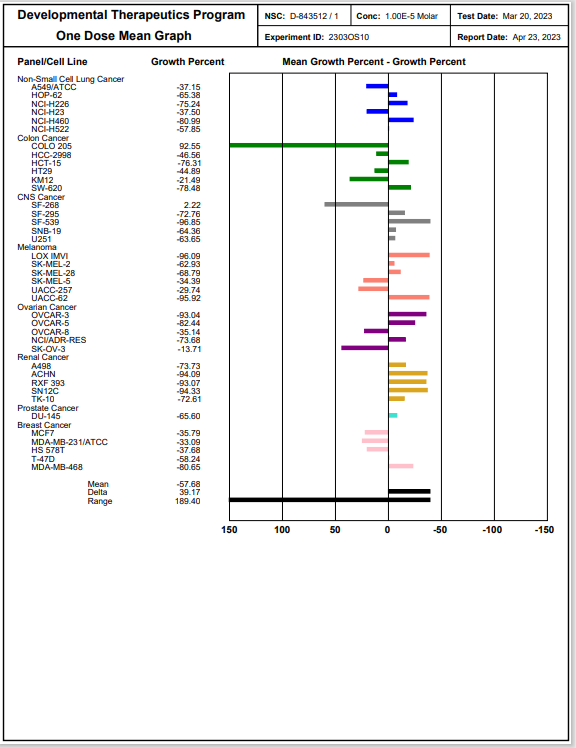


**Figure S22.** NCI60 screening results for **35a**


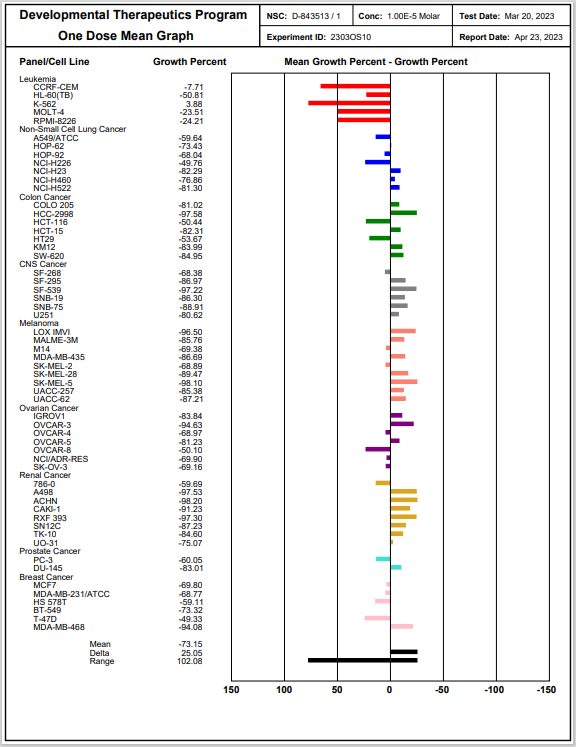


**Figure S23.** NCI60 screening results for **35b**


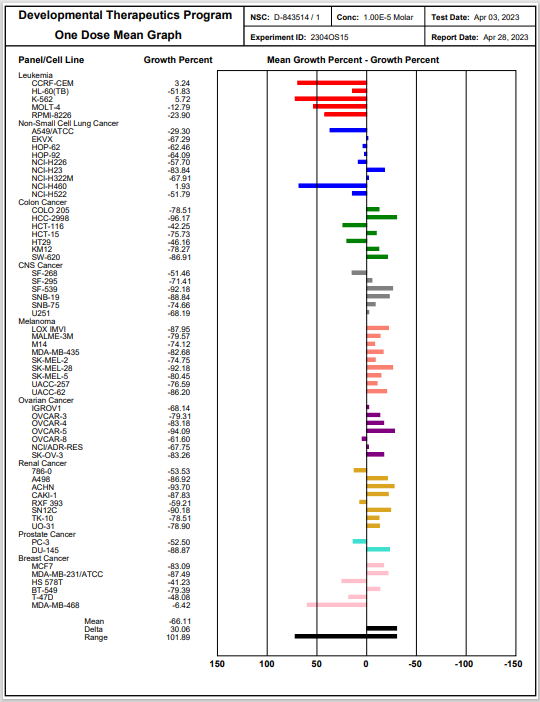


**Figure S24.** NCI60 screening results for **39.**


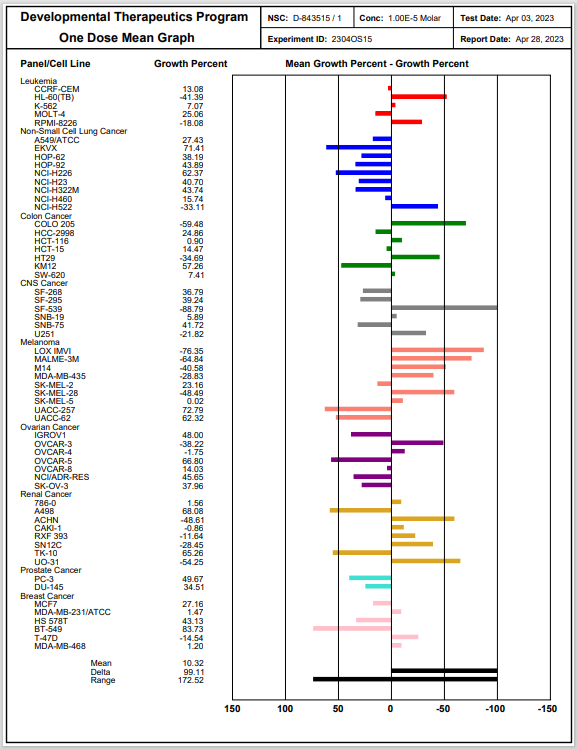


**Figure S25.** NCI60 screening results for **44a.**


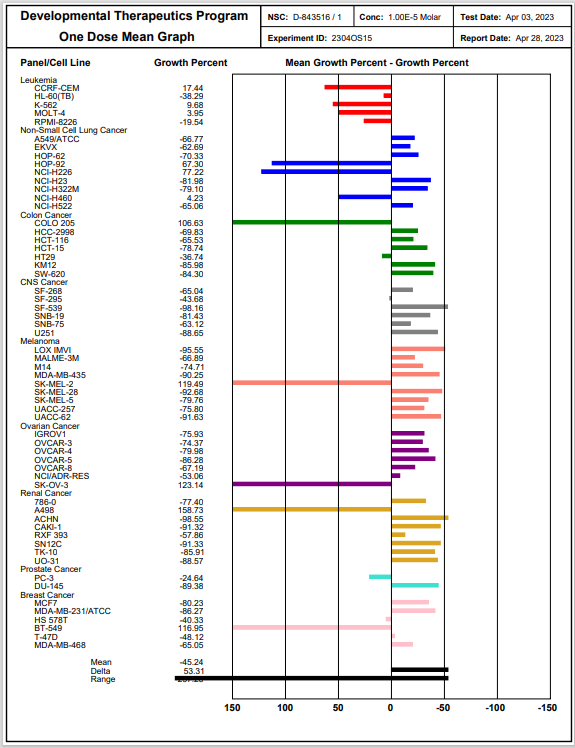


**Figure S26.** NCI60 screening results for **44b.**


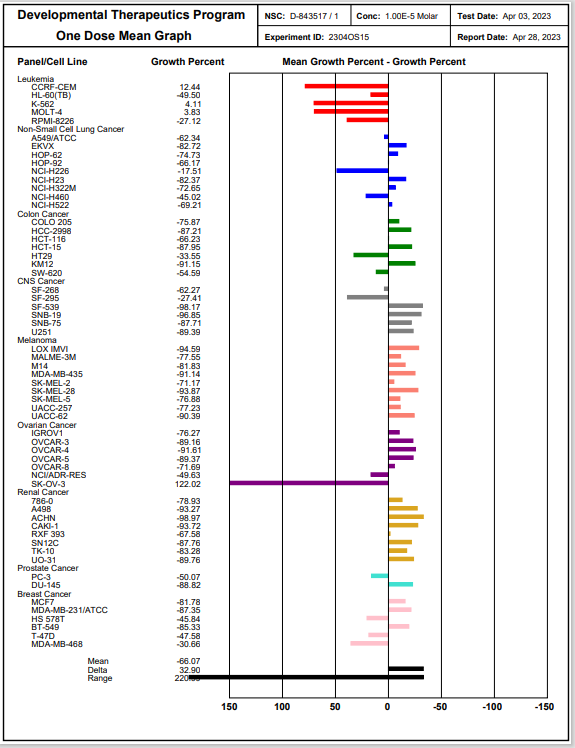


**Figure S27.** NCI60 screening results for **44d.**


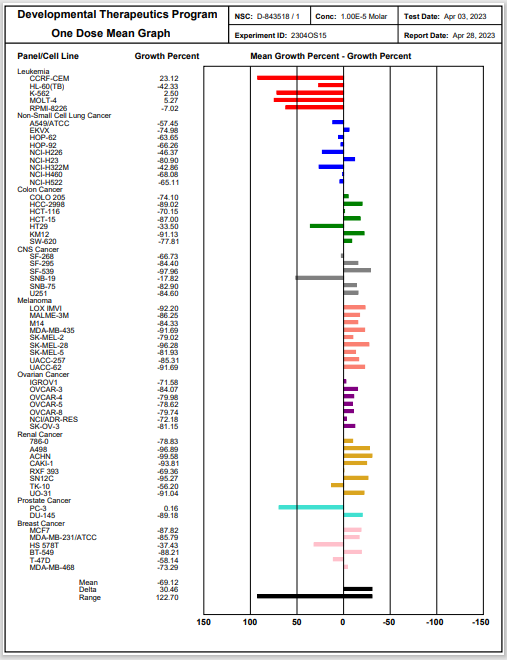


**Figure S28.** NCI60 screening results for **44e.**

**Table S3.** Table displaying the results of the NCI-60 GI_50_ determination for compounds **35a-b**, **39**, **44a-b** and **44d-e** in leukemia, non-small cell lung cancer, colon cancer, CNS cancer and melanoma cell lines.

| Cell line | Compound tag and respective GI_50_ value (µM) | | | | | | |
| --- | --- | --- | --- | --- | --- | --- | --- |
|  | **35a** | **35b** | **39** | **44a** | **44b** | **44d** | **44e** |
| Leukemia | | | | | | | |
| CCRF-CEM | 2.30 | 1.92 | 0.280 | 2.32 | 2.79 | 1.67 | 1.01 |
| HL-60(TB) | 0.217 | 0.309 | 0.219 | 1.58 | 1.88 | 1.23 | 1.99 |
| K-562 | 1.33 | 1.80 | 0.349 | 2.34 | 0.576 | 0.526 | 2.07 |
| MOLT-4 | 2.11 | 1.83 | 0.314 | 3.35 | 2.19 | 1.52 | 2.48 |
| RPMI-8226 | 1.88 | 2.25 | n.t.* | 2.19 | 2.00 | 1.28 | 2.15 |
| SR | n.t.* | n.t.* | /** | 2.01 | 1.63 | 0.975 | 0.233 |
| Non-Small Cell Lung Cancer | | | | | | | |
| A549/ATCC | 1.96 | 1.04 | 3.29 | 4.94 | 1.94 | 1.38 | 1.87 |
| EKVX | 3.80 | 3.41 | 1.05 | 9.85 | 1.71 | 1.51 | 1.79 |
| HOP-62 | 2.08 | 2.54 | 0.290 | 7.10 | 2.07 | 1.55 | 2.84 |
| HOP-92 | 1.75 | 1.81 | 0.486 | 4.63 | 15.3 | 1.24 | 1.81 |
| NCI-H226 | 1.82 | 13.4 | 1.75 | 5.62 | 19.7 | 13.0 | 1.95 |
| NCI-H23 | 2-58 | 1.66 | 0.385 | 4.90 | 1.76 | 1.37 | 1.70 |
| NCI-H322M | 2-38 | 2.14 | 4.47 | 3.18 | 1.93 | 1.18 | 2.03 |
| NCI-H460 | 1.57 | 1.63 | 1.67 | 2.44 | 2.27 | 1.32 | 2.05 |
| NCI-H522 | /** | /** | 1.57 | 2.87 | 1.81 | 1.34 | 1.86 |
| Colon Cancer | | | | | | | |
| Colo 205 | 1.68 | 1.88 | 1.77 | 2.14 | 1.71 | 1.44 | 1.74 |
| HCC-2998 | 1.94 | 1.62 | 0.473 | 3.97 | 1.94 | 1.53 | 1.87 |
| HCT-116 | 1.53 | 1.18 | 0.201 | 2.96 | 2.68 | 1.97 | 2.48 |
| HCT-15 | 1.48 | 1.53 | 0.182 | 3.48 | 1.67 | 1.22 | 1.37 |
| HT29 | /** | 0.079 | 0.182 | 1.79 | 2.09 | 1.46 | 1.80 |
| KM12 | 1.84 | 1.84 | 0.216 | 14.0 | 1.79 | 1.32 | 1.78 |
| SW-620 | 1.75 | 1.40 | 0.244 | 2.28 | 2.46 | 1.31 | 2.45 |
| CNS Cancer | | | | | | | |
| SF-268 | 3.10 | 1.72 | 1.59 | 6.81 | 1.72 | 1.24 | 1.82 |
| SF-295 | 1.81 | 1.75 | 0.184 | 10.6 | 18.7 | 1.31 | 1.73 |
| SF-539 | 1.77 | 1.71 | 0.173 | 1.63 | 1.56 | 1.28 | 1.71 |
| SNB-19 | 3.12 | 1.71 | 0.408 | 4.00 | 1.91 | 1.50 | 2.39 |
| SNB-75 | 1.17 | 1.36 | 0.230 | 1.55 | 1.61 | 1.11 | 1.52 |
| U251 | 1.98 | 1.87 | 0.222 | 2.73 | 2.05 | 1.38 | 1.61 |
| Melanoma | | | | | | | |
| LOX IMVI | 1.64 | 1.70 | 0.171 | 1.63 | 1.64 | 1.23 | 1.70 |
| MALME-3M | 1.66 | 1.54 | 0.170 | 1.95 | 1.82 | 1.21 | 1.77 |
| M14 | 1.69 | 1.79 | 0.205 | 2.69 | 1.92 | 1.28 | 1.85 |
| MDA-MB-435 | 2.27 | 1.65 | 0.184 | 3.37 | 1.73 | 1.27 | 1.69 |
| SK-MEL-2 | 1.85 | 0.055 | 1.73 | 4.27 | 19.4 | 1.20 | 1.81 |
| SK-MEL-28 | 1.83 | 1.58 | 0.184 | 2.29 | 1.75 | 1.39 | 1.77 |
| SK-MEL-5 | 1.75 | 1.54 | 1.72 | 2.25 | 1.51 | 1.15 | 1.44 |
| UACC-257 | 1.82 | 1.80 | 1.73 | 14.0 | 1.93 | 1.45 | 1.75 |
| UACC-62 | 1.58 | 1.64 | 1.66 | 14.1 | 1.73 | 1.32 | 1.67 |

n.t.* - not tested

/** - no GI_50_ was determined for this cell line

**Table S4.** Table displaying the results of the NCI-60 GI_50_ determination for compounds **35a-b**, **39**, **44a-b** and **44d-e** in ovarian, renal, prostate and breast cancer cell lines.

| Cell line | Compound tag and respective GI_50_ value (µM) | | | | | | |
| --- | --- | --- | --- | --- | --- | --- | --- |
|  | **35a** | **35b** | **39** | **44a** | **44b** | **44d** | **44e** |
| Ovarian Cancer | | | | | | | |
| IGROV1 | 1.80 | 1.92 | 1.02 | 3.94 | 2.40 | 1.24 | 1.84 |
| OVCAR-3 | 1.84 | 1.83 | 1.79 | 8.14 | 17.2 | 1.34 | 1.70 |
| OVCAR-4 | 1.93 | 1.79 | 0.209 | 3.21 | 1.93 | 1.44 | 1.83 |
| OVCAR-5 | 1.81 | 1.90 | 1.78 | 11.8 | 2.06 | 1.39 | 2.00 |
| OVCAR-8 | 1.80 | 1.79 | 0.187 | 4.87 | 2.00 | 1.49 | 1.86 |
| NCI/ADR-RES | 1.89 | 1.70 | 0.309 | 5.18 | 2.12 | 1.53 | 1.83 |
| SK-OV-3 | 3.53 | 2.59 | 2.14 | 10.5 | 23.7 | 13.1 | 2.38 |
| Renal Cancer | | | | | | | |
| 786-0 | 2.49 | 1.89 | 0.270 | 3.28 | 1.79 | 1.44 | 1.59 |
| A498 | 2.79 | 1.93 | 1.79 | 3.68 | 20.1 | 11.4 | 1.76 |
| ACHN | 1.65 | 1.47 | 1.74 | 2.85 | 10.6 | 1.40 | 1.71 |
| CAKI-1 | 1.61 | 1.60 | 0.201 | 1.40 | 1.59 | 1.18 | 1.59 |
| RXF 393 | 1.68 | 1.63 | 1.61 | 1.63 | 1.55 | 1.13 | 1.50 |
| SN12C | 1.56 | 1.75 | 1.36 | 1.86 | 1.59 | 1.26 | 1.65 |
| TK-10 | 2.52 | n.t.* | 0.290 | 12.5 | 1.84 | 1.39 | 2.32 |
| UO-31 | 1.42 | 1.68 | 1.42 | 1.43 | 1.59 | 1.16 | 1.39 |
| Prostate Cancer | | | | | | | |
| PC-3 | 2.63 | 2.93 | 0.637 | 7.29 | 2.53 | 1.52 | 3.05 |
| DU-145 | 1.99 | 1.94 | 0.196 | 5.70 | 1.87 | 1.38 | 1.98 |
| Breast Cancer | | | | | | | |
| MCF-7 | 1.85 | 1.62 | 0.185 | 4.79 | 1.75 | 1.35 | 1.55 |
| MDA-MB-231 | 1.72 | 1.72 | 0.203 | 2.34 | 1.74 | 1.35 | 1.80 |
| HS 578T | 1.86 | 1.87 | 0.260 | 1.99 | 2.19 | 1.43 | 2.63 |
| BT-549 | 1.45 | 1.90 | 1.72 | 11.6 | 20.4 | 1.33 | 2.01 |
| T-47D | 1.89 | 2.07 | 1.94 | 3.79 | 1.82 | 1.47 | 1.88 |
| MDA-MB-468 | 1.80 | 1.73 | 0.470 | 1.91 | 14.7 | 1.34 | 1.65 |

n.t.* - not tested

**Table S5.** Additional evaluation of compound **24e** in Ewing sarcoma (SK-N-MC) and leukemia (K562) cell lines.

| Compound | IC_50_ in additional cell lines (µM) | |
| --- | --- | --- |
|  | SK-N-MC | K562 |
| **24e** | 28.5 ± 0.6 | 21.8 ± 5.5 |
| 17-DMAG | 0.01 ± 0.007 | 0.02 ± 0.005 |

# Western blot images used for quantification


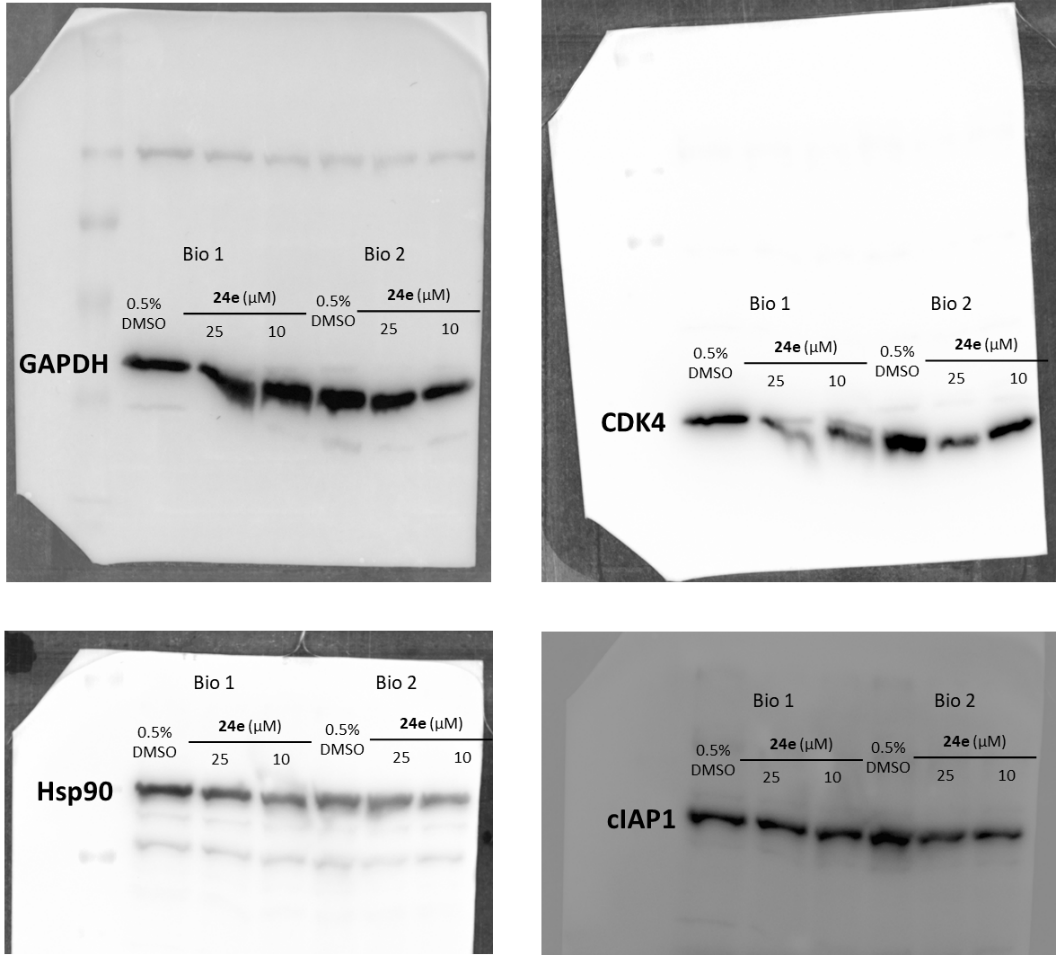


**Figure S29.** Western blot pictures used for quantification of first and second biological repetitions of CDK-4, Hsp90 and cIAP1 along with GAPDH which was used as a standard.


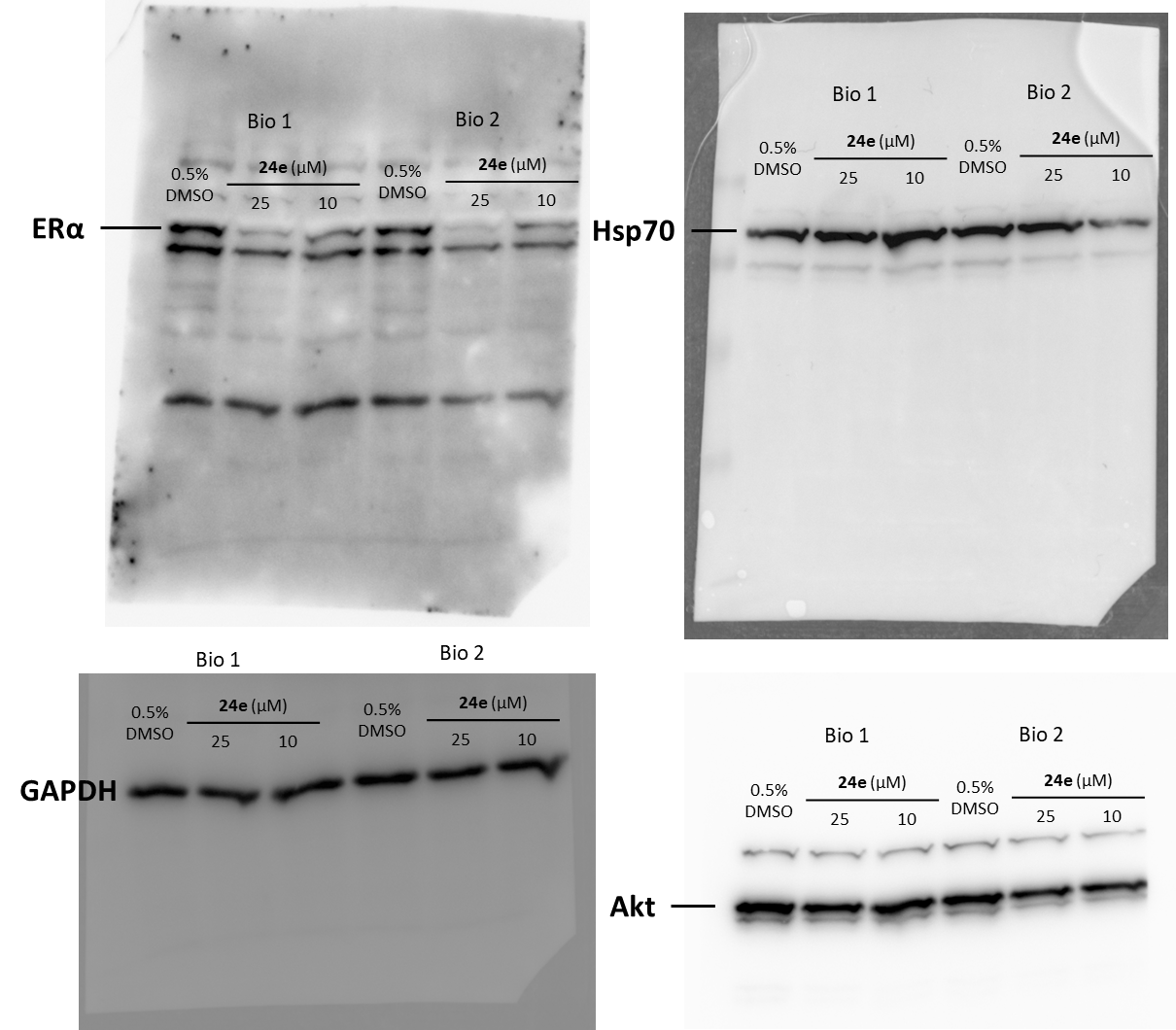


**Figure S30.** Western blot pictures used for quantification of first and second biological repetitions of ERα, Hsp70 and Akt along with GAPDH which was used as a standard.

# STD NMR study – supporting data


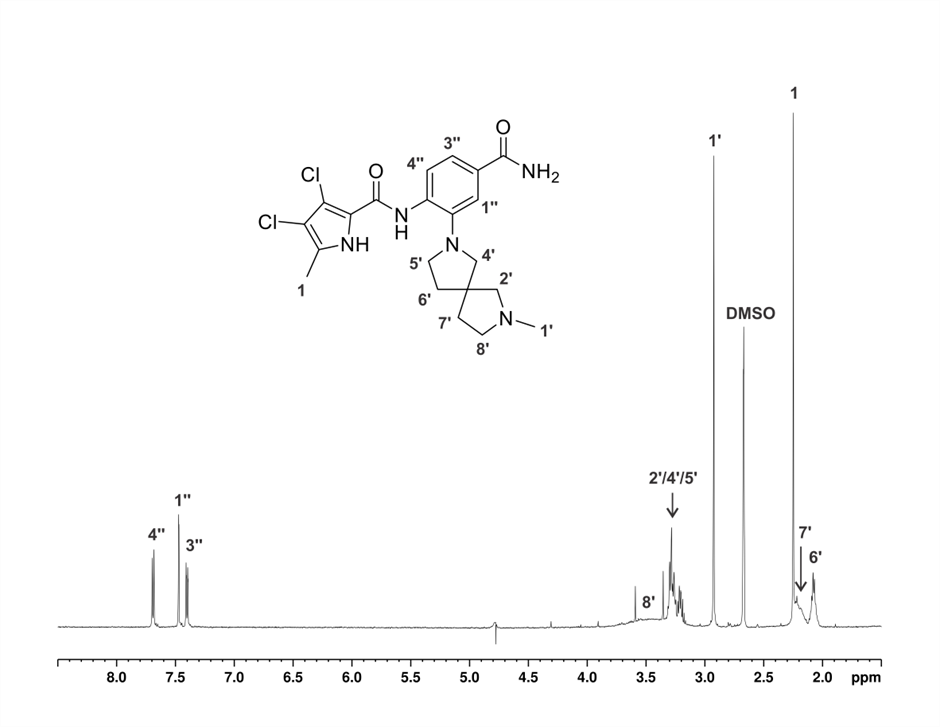


**Figure S31.** ^1^H NMR spectrum for the compound **24e** recorded at a concentration of 0.3 mM in 50 mM K-phosphate buffer (pD 7.5), 100 mM KCl, 2 % DMSO-d_6_ in D_2_O. The proton signals were calibrated to the DSS signal at 0.0 ppm.


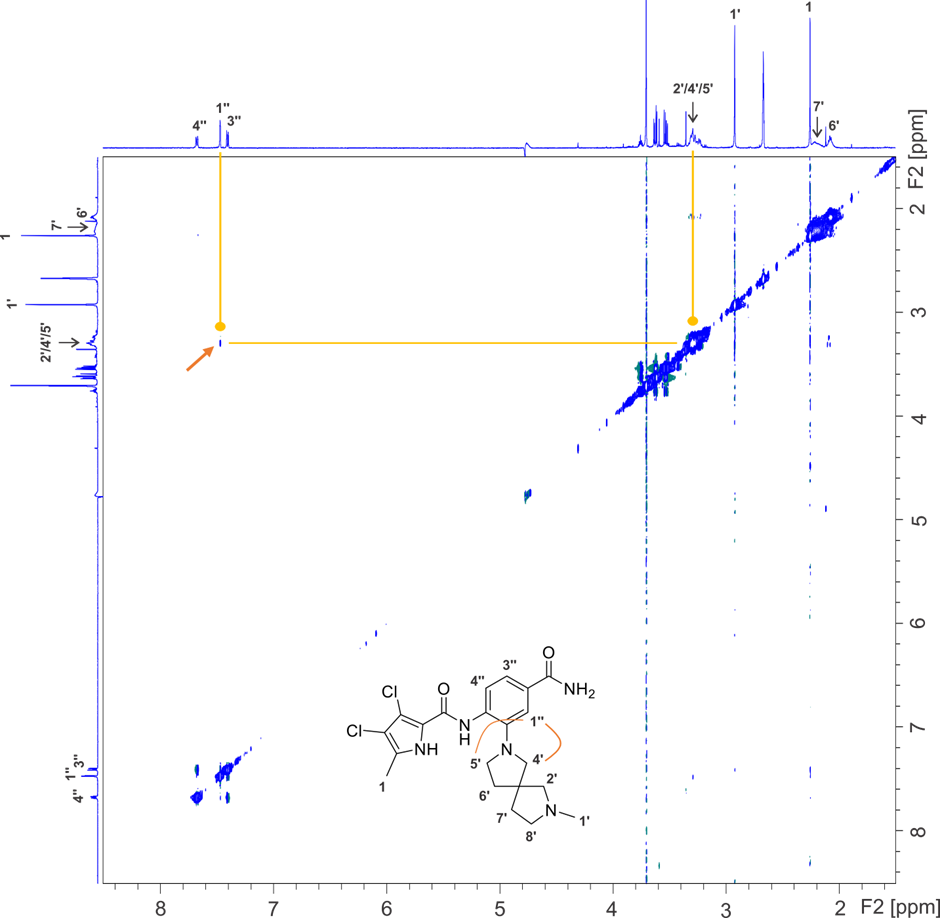


**Figure S32.** The trNOESY spectrum of **24e** in the presence of Hsp90β with the molecular structure illustrating the atom nomenclature and the NOE connectivities between the protons of the different molecular segments. The corresponding NOE and its assignment are shown with an arrow and lines.

**Table S6.** Chemical shifts in ppm of the assigned protons of compound **24e** in phosphate buffer (pD 7.5), referenced to DSS-d_6_.

| **Proton** | **^1^H chemical shift [ppm]** |
| --- | --- |
| 3'' | 7.697 |
| 1'' | 7.479 |
| 4'' | 7.409 |
| 2'/4'/5' | 3.533 |
| 2'/4'/5' | 3.283 |
| 2'/4'/5' | 3.199 |
| 1' | 2.931 |
| 1 | 2.269 |
| 7' | 2.181 |
| 6' | 2.061 |

**Table S7.** STD amplification factors (AMP) and their absolute and relative errors of the assigned protons of compound **24e**. The STD signal of 7’ is partially overlapped and has an insufficient signal-to-noise ratio.

| **Proton** | **AMP** | **AMP absolute error** | **AMP relative error** |
| --- | --- | --- | --- |
| 4'' | 3.45 | 0.04 | 1.2 |
| 1'' | 1.54 | 0.03 | 2.2 |
| 3'' | 2.03 | 0.04 | 1.8 |
| 2'/4'/5' | 1.05 | 0.04 | 3.6 |
| 1' | 0.85 | 0.01 | 1.0 |
| 1 | 2.40 | 0.01 | 0.4 |
| 7' | / | / | / |
| 6' | 1.85 | 0.06 | 3.4 |

# Compound similarity analysis


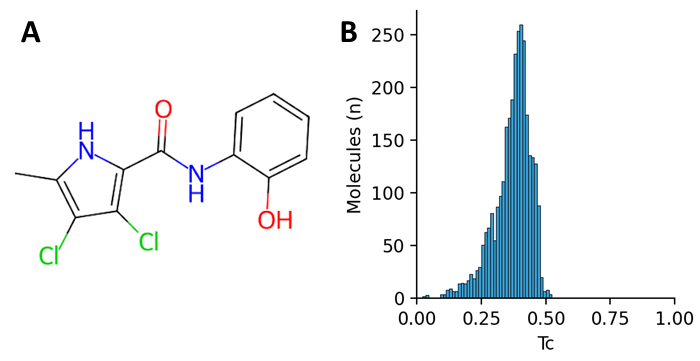


**Figure S33.** The pyrrolamide scaffold (**A**) is dissimilar to other Hsp90α and β binders (**B**). Tc is the Tanimoto coefficient similarity metric where values closer to 1 are more similar and values closer to zero are less similar.

# TopoIIα relaxation assay – curves for IC_50_ determination


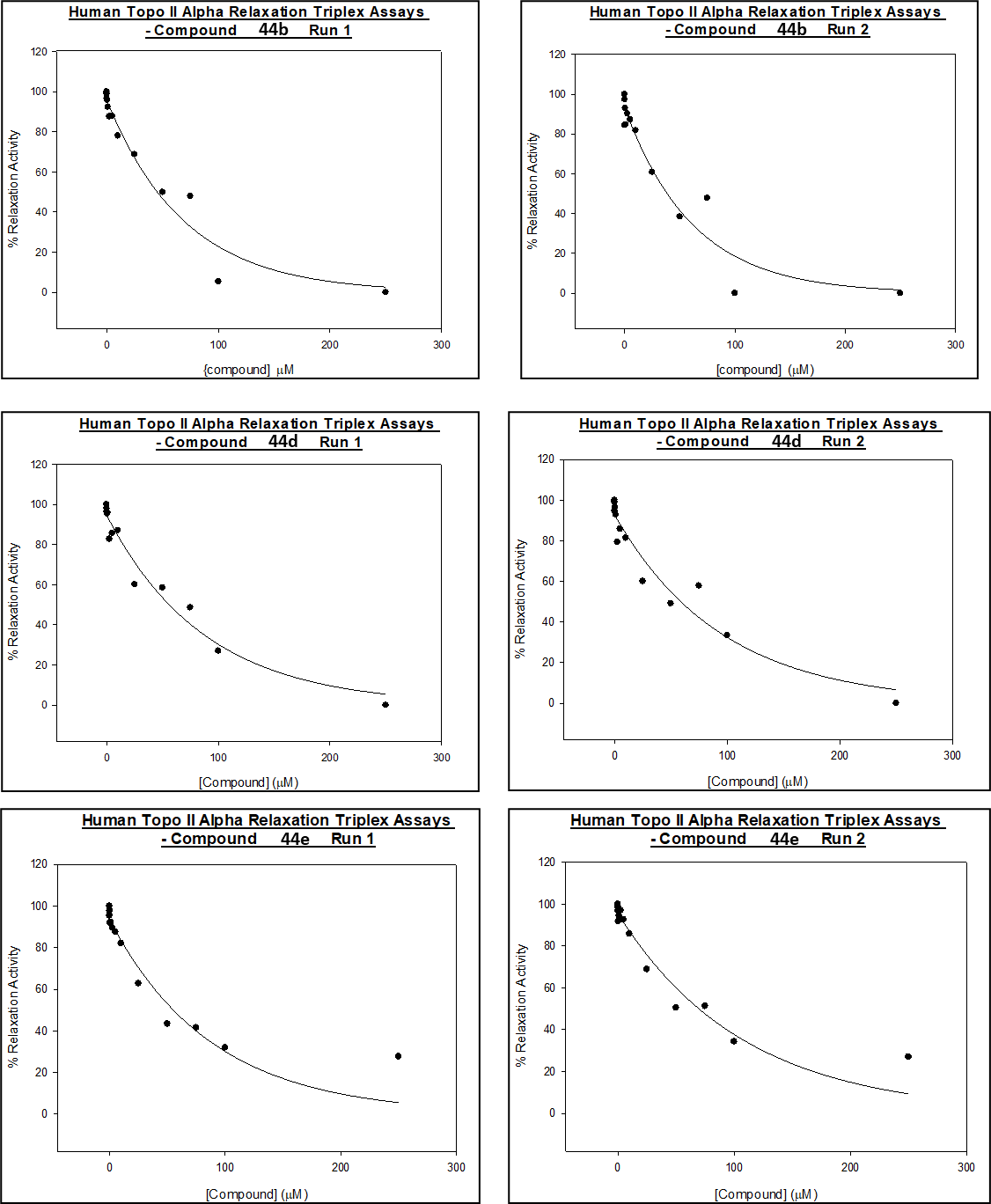


**Figure S34.** TopoIIα relaxation assay IC_50_ determination curves of compounds **44b**, **44d** and **44e** plotted as % of activity against compound concentration.

# Representative ^1^H and ^13^C NMR spectra of the intermediates

Compound **12** ^1^H NMR (400 MHz, DMSO-d_6_)

Compound **12** ^13^C NMR (101 MHz, DMSO-d_6_)

Compound **13** ^1^H NMR (400 MHz, CDCl_3_)

Compound **13** ^13^C NMR (101 MHz, DMSO-d_6_)

Compound **36** ^1^H NMR (400 MHz, CDCl_3_)

Compound **36** ^13^C NMR (101 MHz, DMSO-d_6_)

Compound **37** ^1^H NMR (400 MHz, DMSO-d_6_)

Compound **37** ^13^C NMR (101 MHz, DMSO-d_6_)

Compound **41** ^1^H NMR (400 MHz, DMSO-d_6_)

Compound **41** ^13^C NMR (101 MHz, DMSO-d_6_)

Compound **42d** ^1^H NMR (400 MHz, CDCl_3_)

Compound **42d** ^13^C NMR (101 MHz, DMSO-d_6_)

Compound **43d** ^1^H NMR (400 MHz, DMSO-d_6_)

Compound **43d** ^13^C NMR (101 MHz, DMSO-d_6_)

Compound **43d** ^19^F NMR (376 MHz, DMSO-d_6_)

Compound **19e** ^1^H NMR (400 MHz, CDCl_3_)

Compound **19e** ^13^C NMR (101 MHz, DMSO-d_6_)

Compound **20e** ^1^H NMR (400 MHz, CDCl_3_)

Compound **20e** ^13^C NMR (101 MHz, DMSO-d_6_)

Compound **21e** ^1^H NMR (400 MHz, CDCl_3_)

Compound **21e** ^13^C NMR (101 MHz, DMSO-d_6_)

Compound **22e** ^1^H NMR (400 MHz, DMSO-d_6_)

Compound **22e** ^13^C NMR (101 MHz, DMSO-d_6_)

Compound **23e** ^1^H NMR (400 MHz, DMSO-d_6_)

Compound **23e** ^13^C NMR (101 MHz, DMSO-d_6_)

Compound **23e** ^19^F NMR (376 MHz, DMSO-d_6_)

^1^H and ^13^C NMR spectra of the final biochemically evaluated compounds

Compound **17e** ^1^H NMR (400 MHz, DMSO-d_6_)

Compound **17e** ^13^C NMR (101 MHz, MeOD)

Compound **18a** ^1^H NMR (400 MHz, DMSO-d_6_)

Compound **18a** ^13^C NMR (101 MHz, DMSO-d_6_)

Compound **18b** ^1^H NMR (400 MHz, DMSO-d_6_)

Compound **18b** ^13^C NMR (101 MHz, DMSO-d_6_)

Compound **18c** ^1^H NMR (400 MHz, DMSO-d_6_)

Compound **18c** ^13^C NMR (101 MHz, DMSO-d_6_)

Compound **18d** ^1^H NMR (400 MHz, DMSO-d_6_)

Compound **18d** ^13^C NMR (101 MHz, DMSO-d_6_)

Compound **22i** ^1^H NMR (400 MHz, DMSO-d_6_)

Compound **22i** ^13^C NMR (101 MHz, DMSO-d_6_)

Compound **22j** ^1^H NMR (400 MHz, DMSO-d_6_)

Compound **22j** ^13^C NMR (101 MHz, DMSO-d_6_)

Compound **22k** ^1^H NMR (400 MHz, DMSO-d_6_)

Compound **22k** ^13^C NMR (101 MHz, DMSO-d_6_)

Compound **22l** ^1^H NMR (400 MHz, DMSO-d_6_)

Compound **22l** ^13^C NMR (101 MHz, DMSO-d_6_)

Compound **24a** ^1^H NMR (400 MHz, DMSO-d_6_)

Compound **24a** ^13^C NMR (101 MHz, DMSO-d_6_)

Compound **24b** ^1^H NMR (400 MHz, DMSO-d_6_)

Compound **24b** ^13^C NMR (101 MHz, DMSO-d_6_)

Compound **24c** ^1^H NMR (400 MHz, DMSO-d_6_)

Compound **24c** ^13^C NMR (101 MHz, DMSO-d_6_)

Compound **24d** ^1^H NMR (400 MHz, DMSO-d_6_)

Compound **24d** ^13^C NMR (101 MHz, DMSO-d_6_)

Compound **24e** ^1^H NMR (400 MHz, DMSO-d_6_)

Compound **24e** ^13^C NMR (101 MHz, DMSO-d_6_)

Compound **24f** ^1^H NMR (400 MHz, DMSO-d_6_)

Compound **24f** ^13^C NMR (101 MHz, DMSO-d_6_)

Compound **24g** ^1^H NMR (400 MHz, DMSO-d_6_)

Compound **24g** ^13^C NMR (101 MHz, DMSO-d_6_)

Compound **24h** ^1^H NMR (400 MHz, DMSO-d_6_)

Compound **24h** ^13^C NMR (101 MHz, DMSO-d_6_)

Compound **30** ^1^H NMR (400 MHz, DMSO-d_6_)

Compound **30** ^13^C NMR (101 MHz, DMSO-d_6_)

Compound **35a** ^1^H NMR (400 MHz, DMSO-d_6_)

Compound **35a** ^13^C NMR (101 MHz, DMSO-d_6_)

Compound **35b** ^1^H NMR (400 MHz, DMSO-d_6_)

Compound **35b** ^13^C NMR (101 MHz, DMSO-d_6_)

Compound **39** ^1^H NMR (400 MHz, DMSO-d_6_)

Compound **39** ^13^C NMR (101 MHz, DMSO-d_6_)

Compound **40** ^1^H NMR (400 MHz, DMSO-d_6_)

Compound **40** ^13^C NMR (101 MHz, DMSO-d_6_)

Compound **44a** ^1^H NMR (400 MHz, DMSO-d_6_)

Compound **44a** ^13^C NMR (101 MHz, DMSO-d_6_) ^1^H NMR (400 MHz, DMSO-d_6_)

Compound **44b** ^1^H NMR (400 MHz, DMSO-d_6_)

Compound **44b** ^13^C NMR (101 MHz, DMSO-d_6_)

Compound **44c** ^1^H NMR (400 MHz, DMSO-d_6_)

Compound **44c** ^13^C NMR (101 MHz, DMSO-d_6_) ^1^H NMR (400 MHz, DMSO-d_6_)

Compound **44d** ^1^H NMR (400 MHz, DMSO-d_6_)

Compound **44d** ^13^C NMR (101 MHz, DMSO-d_6_)

Compound **44e** ^1^H NMR (400 MHz, DMSO-d_6_)

Compound **44e** ^13^C NMR (101 MHz, DMSO-d_6_)

Compound **52** ^1^H NMR (400 MHz, DMSO-d_6_)

Compound **52** ^13^C NMR (101 MHz, DMSO-d_6_)

Compound **58** ^1^H NMR (400 MHz, DMSO-d_6_)

Compound **58** ^13^C NMR (101 MHz, DMSO-d_6_)
